# The Immune Cell Infiltration Patterns and Characterization Score in Bladder Cancer to Identify Prognosis

**DOI:** 10.3389/fgene.2022.852708

**Published:** 2022-06-21

**Authors:** Yongsheng Zhang, Yunlong Wang, Jichuang Wang, Kaixiang Zhang

**Affiliations:** ^1^ The First Affiliated Hospital of Zhengzhou University, Zhengzhou, China; ^2^ Academy of Medical Science, Zhengzhou University, Zhengzhou, China; ^3^ Henan Bioengineering Research Center, Zhengzhou, China; ^4^ School of Pharmaceutical Sciences, Zhengzhou University, Zhengzhou, China

**Keywords:** bladder cancer, common gene expression samples data, immune cell infiltration, prognosis, overall survival rate

## Abstract

**Background:** Bladder cancer (BLCA) is among the most frequent types of cancer. Patients with BLCA have a significant recurrence rate and a poor post-surgery survival rate. Recent research has found a link between tumor immune cell infiltration (ICI) and the prognosis of BLCA patients. However, the ICI’s picture of BLCA remains unclear.

**Methods:** Common gene expression data were obtained by combining the Cancer Genome Atlas (TCGA) and Gene Expression Omnibus (GEO) expression databases. Two computational algorithms were proposed to unravel the ICI landscape of BLCA patients. The R package “limma” was applied to find differentially expressed genes (DEGs). ICI patterns were defined by the unsupervised clustering method. Principal-component analysis (PCA) was used to calculate the ICI score. In addition, the combined ICI score and tumor burden mutation (TMB) were utilized to assess BLCA patients’ prognosis. The predictive value of ICI scores was verified by different clinical characteristics.

**Results:** A total of 569 common gene expression data were retrieved from TCGA and GEO cohorts. CD8^+^ T cells were found to have a substantial positive connection with activated memory CD4^+^ T cells and immune score. On the contrary, CD8^+^ T cells were found to have a substantial negative connection with macrophages M0. Thirty-eight DEGs were selected. Two ICI patterns were defined by the unsupervised clustering method. Patients of BLCA were separated into two groups. The high ICI score group exhibited a better outcome than the low ICI score one (*p* < 0.001). Finally, the group with a high tumor mutation burden (TMB) as well as a high ICI score had the best outcome. (*p* < 0.001).

**Conclusions:** Combining TMB and ICI scores resulted in a more accurate survival prediction, suggesting that ICI scores could be used as a prognostic marker for BLCA patients.

## Introduction

Bladder cancer (BLCA) is the world’s 10th most prevalent cancer, accounting for around 549,000 new cases and 200,000 deaths in 2018 (Bray et al., 2018). As a highly heterogeneous tumor (Knowles and Hurst, 2015; Babjuk et al., 2017; Li et al., 2020), BLCA has a high recurrence rate (around 50%) and the five-year survival rate was around 60% after trimodal therapy (Cambier et al., 2016; Kamat et al., 2016; Sanli et al., 2017). Despite the rapid development of clinical imaging after chemotherapy and surgery, the method for evaluating the therapeutic effect of BLCA is not satisfactory. As a result, developing new diagnostic, therapeutic, and prognostic biomarkers for BLCA is critical.

Immune checkpoint inhibitor (ICI_S_), a type of immunotherapy, could kill tumor cells. However, it only works for a few patients with advanced cancer (Johnson et al., 2017; D’Aiello et al., 2021; Topalian et al., 2015; Tøndell et al., 2021; Zou et al., 2016; Dermani et al., 2019). TMB is a predictive biomarker of immunotherapy because it reflects the overall load of new antigens (Rizvi et al., 2015; Hellmann et al., 2019; Zhao et al., 2021). However, the breakpoint between TMB-high and TMB-low is difficult to define (Samstein et al., 2019). Therefore, it is critical to find novel biomarkers that could predict the response of the tumor to immunotherapy.

Extensive research has established the crucial involvement of immune cell infiltration (ICI) in cancer proliferation, recurrence, and metastasis (Jiang et al., 2018; Zeng et al., 2018). The higher the proportion of immune score in the tumor microenvironment, the better prognosis in most patients (Na and Choi, 2018).In addition, tumor-infiltrating lymphocytes (TLSs), including CD4 and CD8 T cells, have been linked to increased survival rates (Vassilakopoulou et al., 2016; Zeng et al., 2019). In contrast, TAM exerts its tumor-promoting effect mainly through the following three ways (Noy and Pollard, 2014; Chen et al., 2019; Liang et al., 2020): 1) TAM can promote tumor growth, invasion, metastasis, and angiogenesis by secreting a variety of cytokines; 2) TAM is also immunosuppressive, inhibiting adaptive immune response and promoting tumor immune escape; 3) TAM can also induce drug resistance of tumor cells by promoting abnormal angiogenesis, affecting the transport of drugs in the blood, and weakening the signal of tumor cell apoptosis. Nevertheless, recognizing TLS cells is insufficient to characterize the complicated tumor microenvironment. TLSs and TAMs interact with each other, indicating that the link between the two sets of TME cells is more important than any single component (Sanli et al., 2017).

In this study, two approaches “CIBERSORT” and “ESTIMATE” were employed to unveil the patient’s ICI picture. In addition, based on the ICI and DEGs, BLCA patients were classified into two subgroups. The ICI score was acquired by principal-component analysis (PCA). Finally, the ICI score was developed to describe distinct immune cell landscapes, which could exactly predict patient outcomes. “As a result, we discovered that ICI score could serve as a prospective prognostic marker that is different from TMB.”

## Methods

### BLCA Data Collection

TCGA and GEO databases were used to gather transcriptome and clinical data. In general, we collected two groups of cohort samples of BLCA: GSE13507 and TCGA-BLCA. The exclusion criteria were as follows: 1) Not tumor tissue sample. 2) The transcriptome sequencing data or clinical information of the samples were incomplete. 3) Not common gene expression sample data. Finally, 569 samples were included. We converted the fragments per kilobase million (FPKM) values to the transcripts per million (TPM) values by using the “limma” R package for the TCGA-BLCA database. We combined TCGA and GEO expression data to get new common gene expression samples’ data for later analysis.

### The Proportion of ICI Was Used to Categorize BLCA Patients

The “CIBERSORT” R package, the LM22 signature, and 1,000 permutations were used to evaluate infiltration levels for various immune cells in BLCA. ESTIMATE calculated the immune score and stromal score in BLCA patients. In addition, we acquired the correlation between different immune cells by using the “corrplot” R package. The hierarchical agglomerative clustering of BLCA was implemented by different ICI patterns of each sample. The number of clusters was determined by the consensus clustering algorithm. We performed the “ConsensusClusterPlus” R package and repeated it 1,000 times to ensure the stability of classification.

### Acquisition of Differentially Expressed Genes Related to ICI Phenotype

In order to find genes linked with ICI patterns, we classified patients into distinct groups based on ICI. DEGs among different groups were screened by means of the R package “limma.” The significant criteria of |log FC| > 1 and *p* (adjust) < 0.05 were used to determine DEGs.

### Generation of ICI Score

In order to further analysis, an unsupervised clustering method for DEG analysis was applied to divide the patients into different groups. Positive and negative DEG correlations with cluster signatures were classified as ICI gene signatures A and B, and the “Boruta” algorithm was applied to reduce their dimensionality. Using the PCA, the gene signature score of patients was derived. Finally, we used a procedure analogous to the grading index gene expression to determine the ICI score. ICI score = ∑PC1A-∑PC1B.

### Collection of Somatic Structural Variation Data

The correlation mutation information of patients in the TCGA-BLCA cohort was obtained from the TCGA data portal (https://portal.gdc.cancer.gov/repository). In order to determine tumor mutation burden, we calculated the total number of non-synonymous mutations in BLCA. We got 20 driver genes through the R package “maftool,” which had the highest mutation frequency in BLCA patients. Finally, we evaluated whether differences in the mutation frequency of genes between two ICI score groups.

### Gene Ontology and Gene Set Enrichment Analysis

The “clusterprofiler” package was utilized for gene annotation and enrichment analysis of ICI distinctive genes. Gene ontology (GO) terms were screened by a stringent cut-off (*p* < 0.05). Furthermore, we used a gene set enrichment analysis (GSEA) to find pathways that were up- and down-regulated between two ICI score groups. The parameter settings were Gene sets database = “Kyoto Encyclopedia of Genes and Genomes (KEGG),” n Perm = 1,000, and *p* < 0.05.

### Analysis of Clinical Features in Two ICI Score Groups

The corresponding clinical data from the TCGA and GEO databases were retrieved and manually organized. We verified the predictive value of the ICI score with distinct clinical features (such as age and gender) by the R package” survival.”

### Statistical Analysis

All data were analyzed by R software (version 4.0.4). The proportion of 22 types of immune cells in BLCA was calculated by the “CIBERSORT” algorithm. The “ConsensusClusterPlus” R package was used to divide BLCA patients into two types. DEGs between two ICI phenotypes were filtered through the R package “limma.” The ICI score was calculated by the PCA algorithm. Further analysis, TMB was obtained in TCGA-BLCA by “TMB.pl.” The prognosis of BLCA patients was evaluated by the R package “survival.” The “clusterprofiler” package was used for gene annotation and enrichment analysis of ICI distinctive genes. The predictive value of ICI scores with different clinical characteristics (such as age and gender) was verified by the R package “survival.” *p* < 0.05 was considered statistically significant.

## Results

### The Pattern e of ICI in the TME of BLCA

**FIGURE 1:**
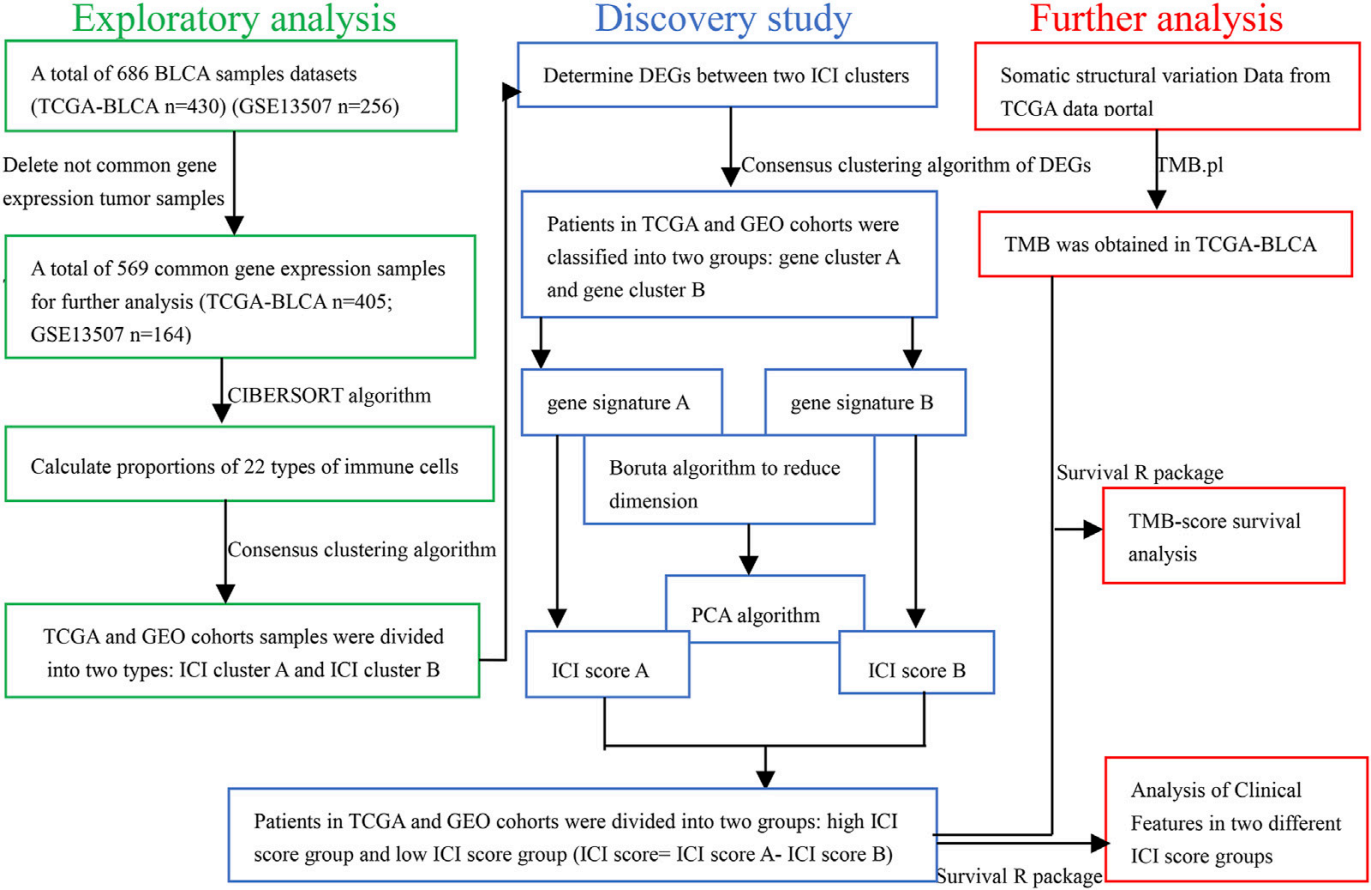
Schematic diagram of research design.

**FIGURE 2:**
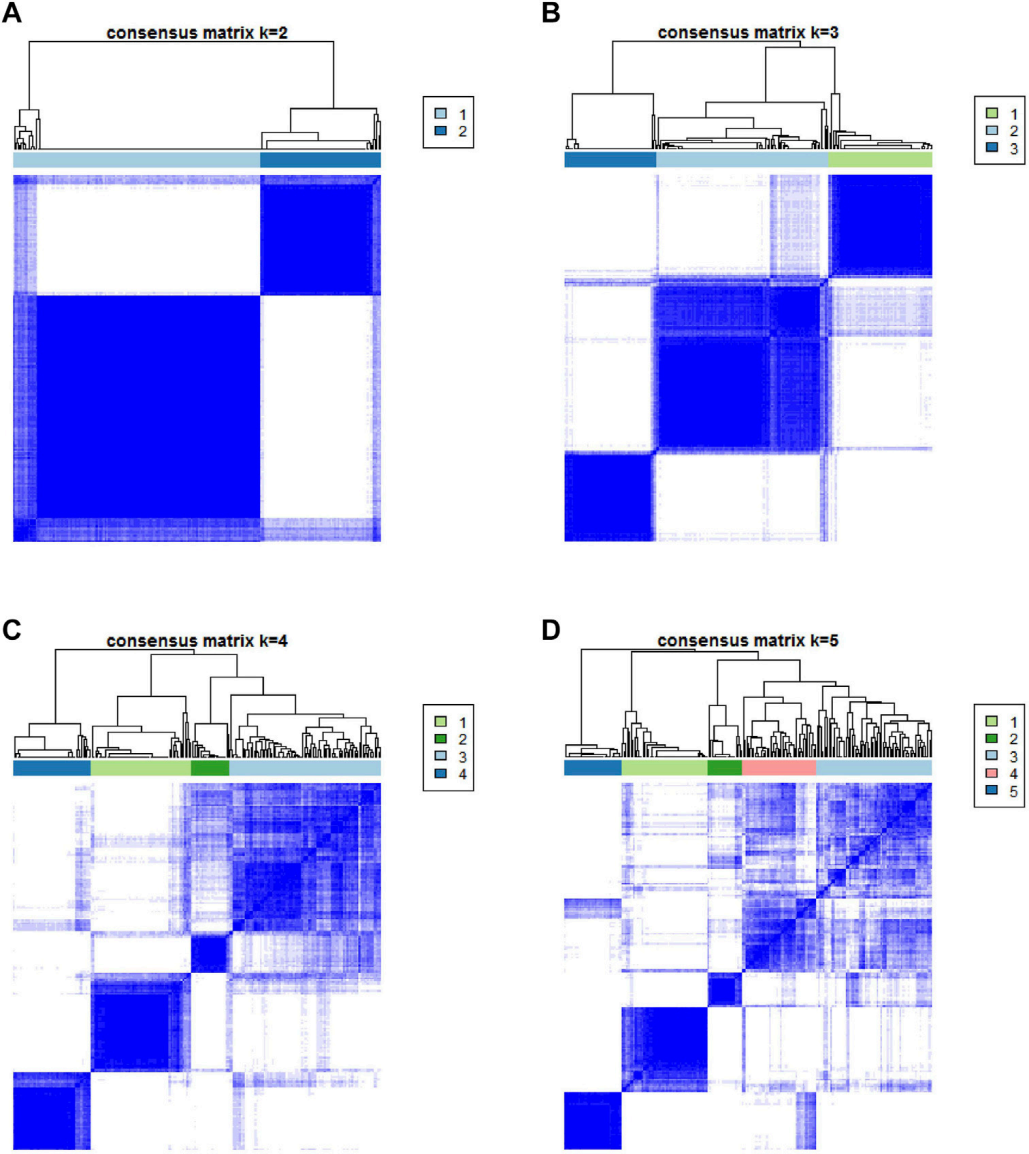
Based on the proportion of infiltrating immune cells, consensus matrixes of all BLCA samples. **(A–D)** In consensus matrixes of all BLCA samples for each k (k = 2–5), the consensus matrix was the crispest when K = 2.

**FIGURE 3:**
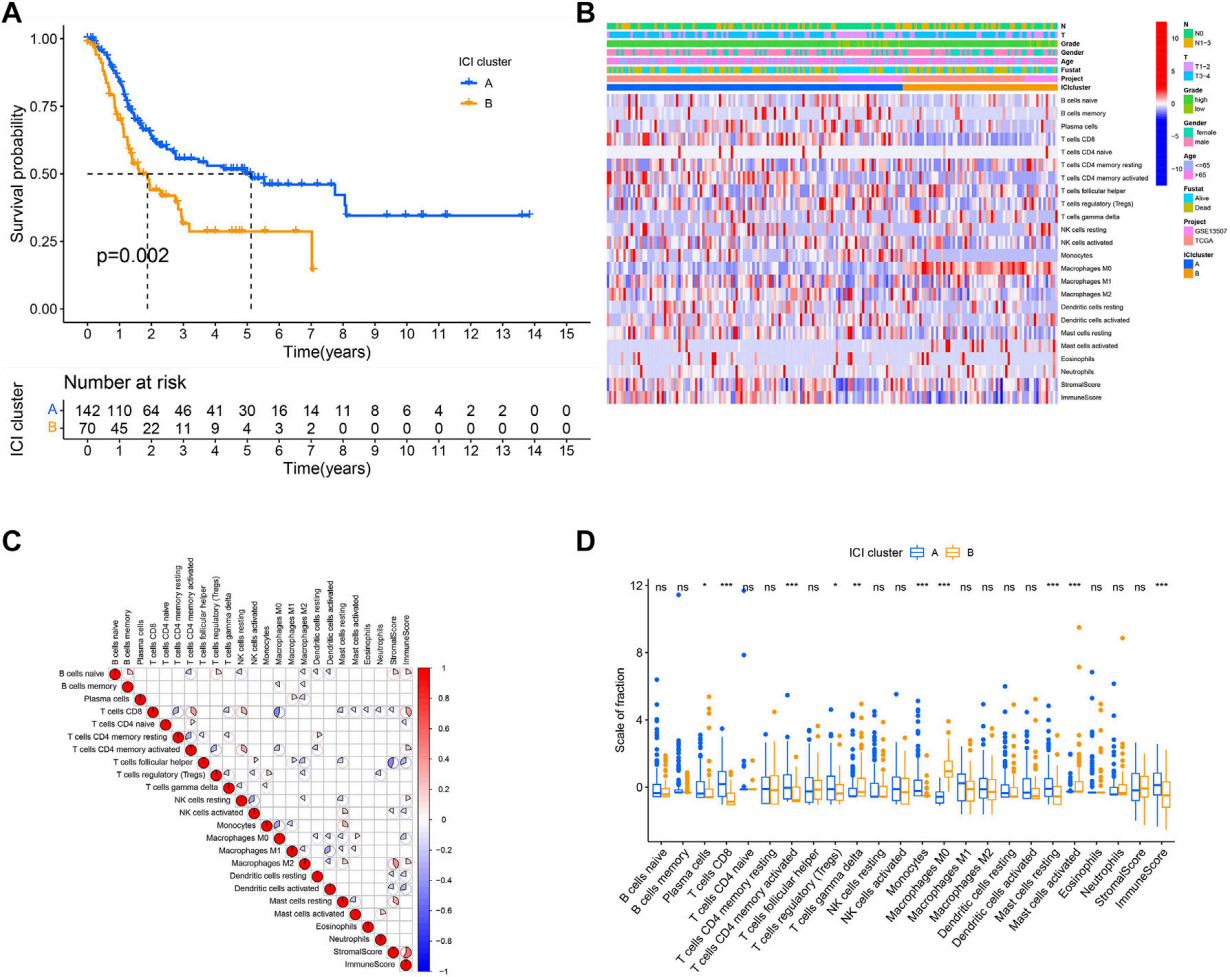
The pattern of ICI in the TME of BLCA. **(A)** Survival time of patients in two independent ICI subtypes. **(B)** Visualized the expression levels of immune cells of distinct ICI clusters. **(C)** The correlation among different immune cells. **(D)** The proportion of tumor-infiltrating immune cells in two ICI clusters. We also depicted the immune score and stromal score of two ICI clusters. *p < 0.05; **p < 0.01; ***p < 0.001.

The workflow is displayed in Figure 1. First, a total of 569 common gene expression data were extracted from TCGA and GEO cohorts. Then, the CIBERSORT and ESTIMATE algorithms were applied to evaluate the levels of immune cells (filter conditions: *p* < 0.05) in BLCA patients. (Supplementary Tables 1, 2). The correlation coefficient heatmap shows the significant positive correlation of CD8 T cells with activated memory CD4 T cells and immune score. On the contrary, there was a significant negative correlation between CD8 T cells with macrophages M0 (Figure 3C). Based on the results of immune cell infiltration, unsupervised clustering was performed by using the “ConsesusClusterPlus” package of R software to divide BLCA patients into two distinct ICI subtypes. The consensus matrix was the crispest when K = 2 (Figures 2A–D), namely, ICI clusters A and B (Supplementary Table 3). The heatmap enables visualization of the expression levels of immune cells of distinct ICI clusters (Figure 3B). Moreover, two independent ICI subtypes showed a significant difference in the overall survival rate (*p* = 0.002; Figure 3A).

To further describe and understand the biological and clinical distinctions among these inherent features, we analyzed the immune cell composition of two ICI subtypes. Between two ICI subtypes, ICI cluster A had a better outcome with a median duration of roughly five years. Meanwhile, it was marked by increased infiltration of CD8 T cells, activated memory CD4 T cells, resting mast cells, etc. In addition, the ICI cluster A has a higher immune score than the ICI cluster B. On the contrary, the ICI cluster B confirmed a poor prognosis (median survival duration of roughly three years) and showed a large rise in the number of macrophages M0 (Figure 3D).

### Identified the Subtypes of Immune-Related Gene

**FIGURE 4:**
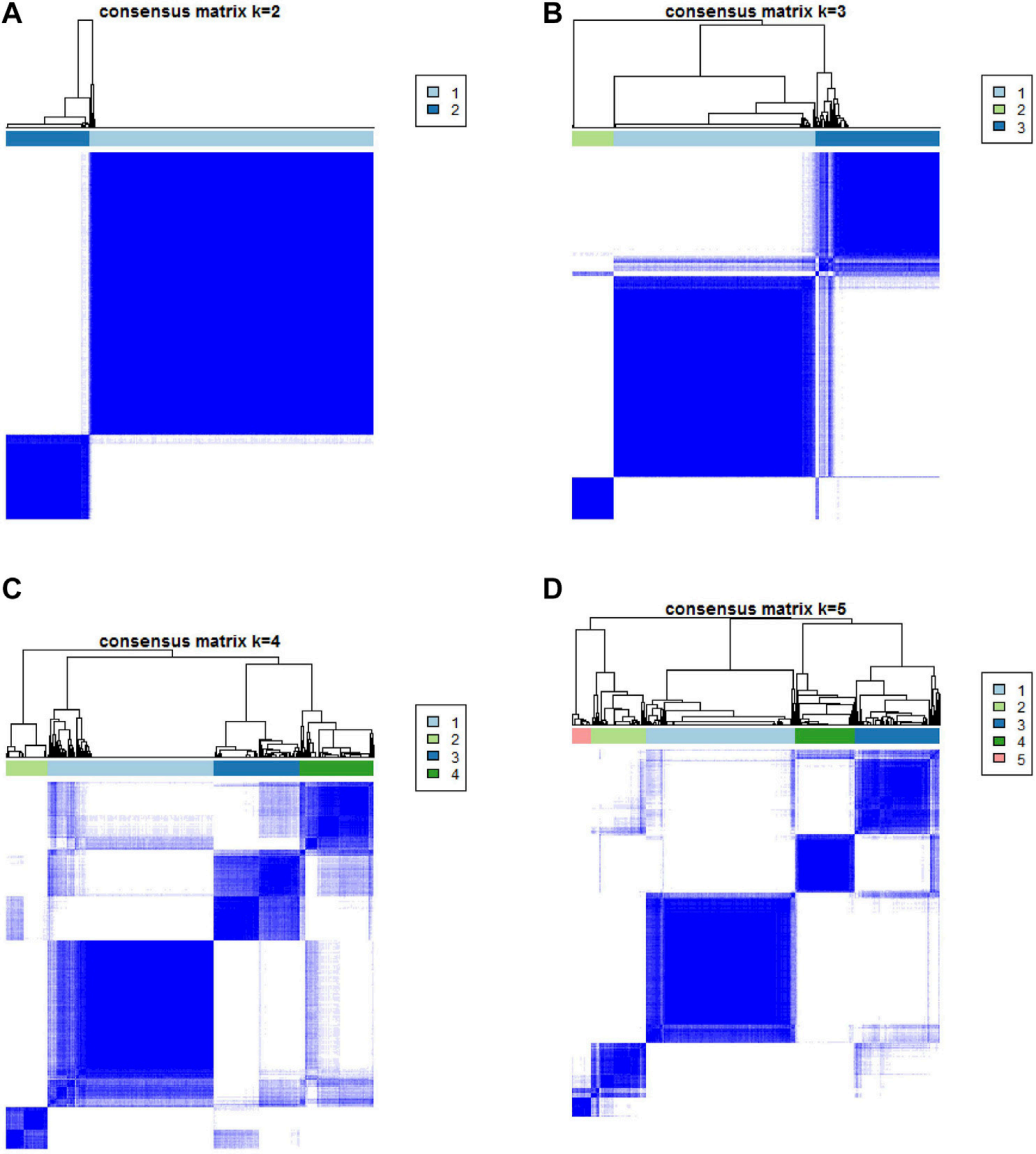
Based on DEGs, consensus matrixes of all BLCA samples. **(A–D)** In consensus matrixes of all BLCA samples for each k (k = 2–5), the consensus matrix was the crispest when K = 2.

**FIGURE 5:**
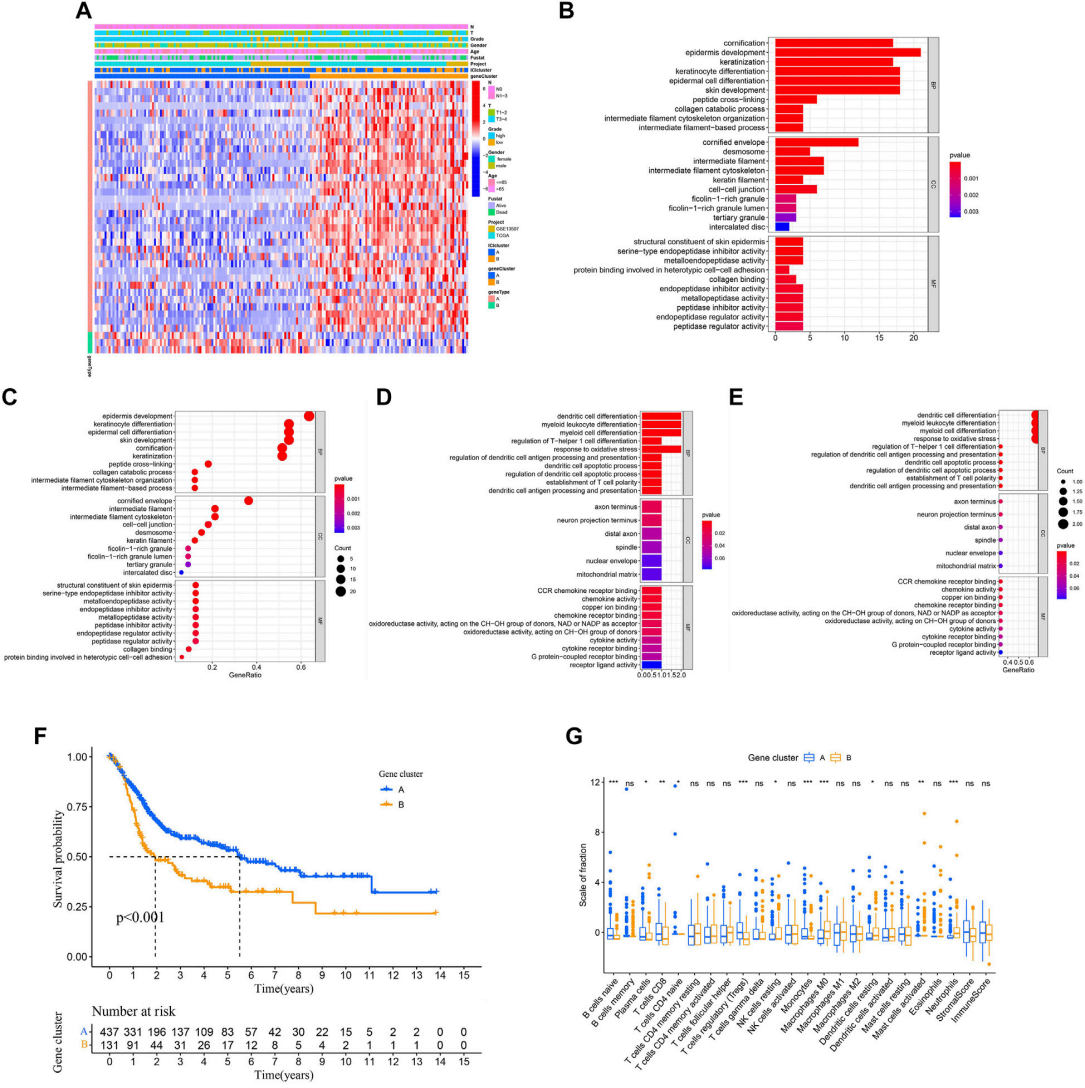
Identification of the subtype of the immune gene. **(A)** Description of the transcriptomic profile of DEGs identified across the genomic clusters. **(B–E)** Functional and pathway enrichment analyses (Gene Ontology-Biological process) of ICI gene signatures A and B: ICI signature genes **A (B–C)** and **B (D–E)**. **(F)** Two independent gene clusters had a significant difference in the overall survival rate (log-rank test, *p* < 0.001). **(G)** The proportion of tumor-infiltrating immune cells in the two gene clusters. We also depicted the immune score and stromal score of two gene clusters. *p < 0.05; **p < 0.01; ***p < 0.001.

In order to reveal the underlying biological properties of distinct immunophenotypes, we used the R package “limma” to carry out differential analysis to identify the transcriptome differences between two subtypes. Unsupervised clustering was implemented using the “limma” package of R software to obtain the differentially expressed genes (DEGs) (Supplementary Table 4). We classified BLCA patients into gene clusters A–B by DEGs (Figures 4A–D; Supplementary Table 5). The positive correlation of DEG values with the clusters signature was coded as ICI gene signature A, and the rest of the DEGs were coded as ICI gene signature B. At the same time, to reduce noise or redundant genes, we employed the “Boruta” method to reduce the dimension of gene signatures A and B. The transcriptome properties of DEGs are shown in a heatmap created with the R package “pheatmap (Figure 5A).” The R package “clusterProfiler” was applied to execute GO enrichment analysis on the signature genes. The significantly enriched biological processes are summarized in Figures 5B–E, and a detailed description is provided in Supplementary Table 6.

Next, we evaluated the prognosis of gene clusters A–B combined with survival information. It showed that two independent gene clusters had a significant difference in overall survival (*p* < 0.001; Figure 5F). The gene cluster A was characterized by a better outcome (median survival duration of roughly 5.5 years), whereas the gene cluster B had unfavorable outcomes (median survival duration of roughly two years). As displayed in Figure 5G, the gene cluster A showed an obvious increase in the infiltration of some immune cells, such as CD8 T cells and naive B cells. Meanwhile, the gene cluster B performed a higher macrophages M0 infiltration. Finally, some differentially expressed target genes were analyzed in two gene clusters by the “limma” package.

### Generation of ICI Score

**FIGURE 6:**
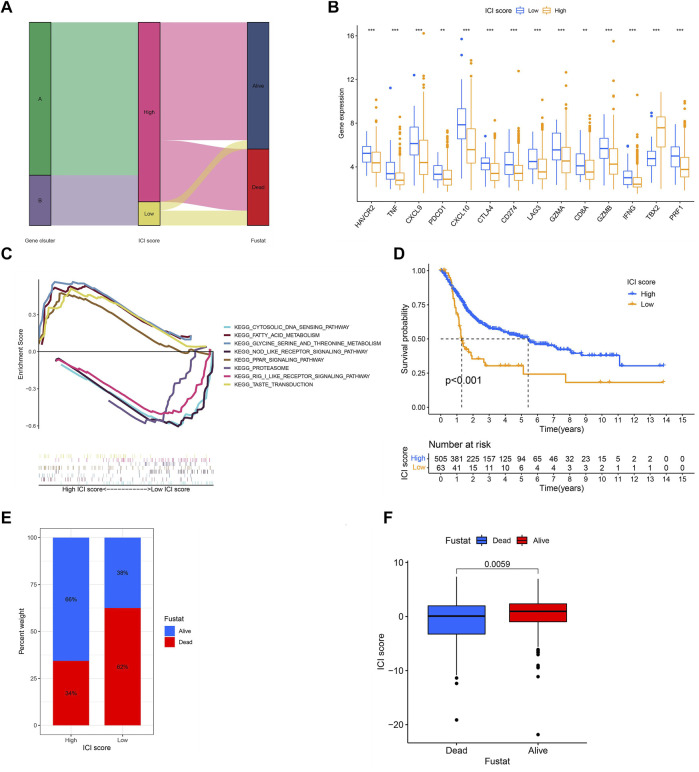
Generation of ICI score. **(A)** The alluvial diagram described the correlation among the gene clusters, the ICI score, and survival outcomes. **(B)** The expression levels of immune-checkpoint–relevant signatures (CD274, CTLA4, HAVCR2, LAG3, and PDCD1) and immune-activity–related signatures (CD8A, CXCL10, CXCL9, GZMA, GZMB, IFNG, PRF1, TBX2, and TNF) in high and low ICI score groups. *p < 0.05; **p < 0.01, ***p < 0.001; **(C)** GSEA analysis results exhibited that some significantly enriched functions or pathways in high and low ICI score groups. **(D)** Survival time of patients in high and low ICI score groups (log-rank test, *p* < 0.0010. **(E,F)** Most of the patients in the high ICI score group were alive; on the contrary, most of the patients in the low ICI score group were dead (log-rank test, *p* = 0.0059).

In order to obtain the quantitative index of the ICI landscape of BLCA patients, we computed two aggregate scores by the PCA algorithm: the ICI score A from ICI signature gene A and the ICI score B from ICI signature gene B. We obtained the sum of individual scores using ICI scores A and B of each sample in the study. Finally, we obtained the ICI score, which is a predictive signature score. The TCGA-BLCA and GSE13507 patients were separated into high and low ICI score groups using the “survival” package (Supplementary Table 7). The alluvial diagram described the correlation among the gene clusters, the ICI score, and survival outcomes (Figure 6A). CD274, CTLA4, HAVCR2, LAG3, and PDCD1 were chosen as immune-checkpoint–relevant signatures, and CD8A, CXCL10, CXCL9, GZMA, GZMB, IFNG, PRF1, TBX2, and TNF were selected as immune-activity–related signatures, to investigate the immunological activation and tolerant state of the TCGA-BLAC and GSE13507 cohorts. With the exception of TBX2, the ICI score was shown to have a substantial negative correlation with the expression quantity of immune-checkpoint–relevant and immune-activity–relevant genes (Figure 6B). Moreover, GSEA analysis results revealed that fatty acid metabolism and PPAR signaling pathways were considerably enriched in the high ICI score group, whereas proteasome and NOD-like receptor signaling pathways were substantially enriched in the low score one (Figure 6C). Detailed enriched information and description is provided in Supplementary Table 8. Next, we evaluated the impact of the ICI score on the prognosis of patients. It showed that two independent ICI score groups had remarkable difference in the overall survival rate (*p* < 0.001; Figure 6D). The high ICI score group showed a good prognosis (median survival duration of roughly 5.3 years), whereas the low one had an unfavorable outcome (median survival duration of roughly 1.2 years). Lastly, the “ggplot2” package was applied to evaluate the relation between ICI score and survival status. We found that two independent ICI score groups had a significant difference in survival status. The majority of BLCA in the high ICI score group were alive; on the contrary, the majority of BLCA in the low one was dead (*p* = 0.0059; Figures 6E,F).

### TMB and ICI Score Were Applied to Evaluate the Prognosis of TCGA-BCLA Cohort Patients

**FIGURE 7:**
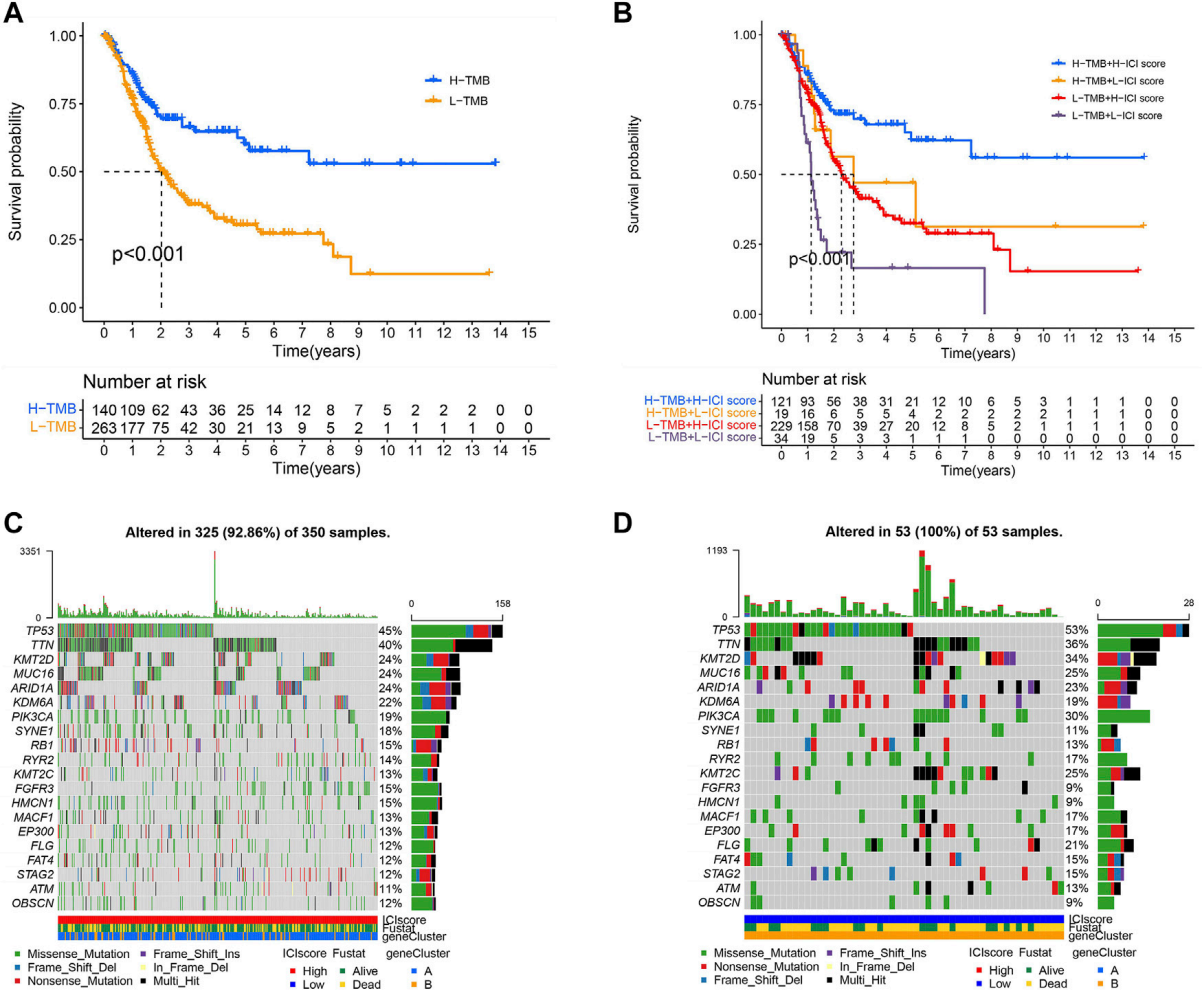
TMB and ICI scores were used to evaluate the prognosis of TOGA-BOLA cohort patients. **(A)** Survival time of patients in high- and low-TMB groups (log-rank test, *p* < 0.0010. **(B)** The high-TMB group combined with the high ICI score group showed the best prognosis in BLCA patients (log-rank test, *p* < 0.001; **(C,D)** The distribution of driver genes in high and low ICI score groups: high ICI score group **(C)** and low ICI score group **(D)**.

Since BLCA was reported to have a high degree of somatic changes, subsequently, we determined the distribution of somatic mutations and combined it with the ICI score to evaluate the prognosis of patients. First, the total mutation burden and mutation distribution of TCGA-BCLA were obtained by analyzing mutation annotation files. Meanwhile, we divided the patients into high- and low-TMB groups. As demonstrated in Figure 7A, we discovered that the high-TMB group was related to a better outcome than the low one (*p* < 0.001). Considering the contraindication value of TMB and ICI score for prognosis, we subsequently studied the synergistic effect of ICI score in the prognostic classification of BLCA. The results reveal that there was a substantial difference in survival between the high- and low-TMB groups depending on ICI score subtypes. Among them, the high TMB combined with a high ICI score had the best prognosis in BLCA (*p* < 0.001; Figure 7B). In conclusion, the ICI score could be utilized as a possible predictor different from TMB, which could effectively predict the response to immunotherapy. In addition, we screened out 20 driver genes with the highest mutation frequency for further analysis. We analyzed the distribution of driver genes in high and low ICI score groups. The result showed that the alteration frequency of TP53, KMT2D, PIK3CA, KMT2C, and FLG was considerably different between the two ICI score groups (Figures 7C,D). Moreover, we discovered that the TP53 mutation frequency was higher in the low ICI score group. The result proved once again that the group with the low ICI had a poor prognosis. These results may provide new ideas for the study of the mechanism of ICI in tumors (Şenbabaoğlu et al., 2016; Chen and Mellman, 2017).

### Analysis of Clinical Features in Two ICI Score Groups

**FIGURE 8:**
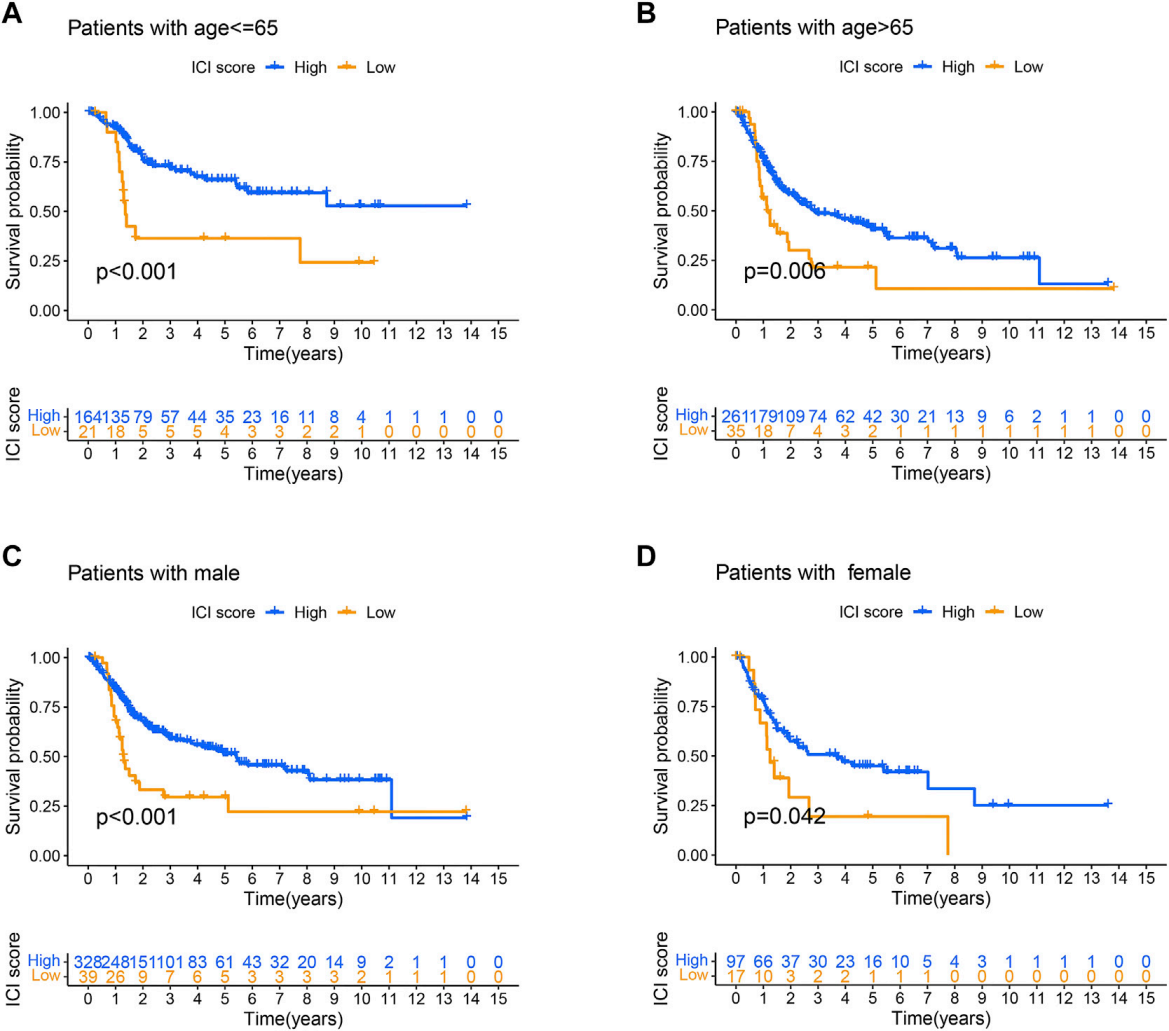
Analysis of clinical features in high and low ICI score groups. **(A–D)** Survival time of patients with high and low ICI score groups in distinct clinical features: **(A)** age ≤ 65; **(B)** age > 65; **(C)** male patients; **(D)** female patients.

To be able to clarify the role of ICI score in BLCA, the relationship between ICI score and clinical characteristics was researched. The stratified survival analysis was used to observe whether ICI scores could be applied to different clinicopathological features. Next, we analyzed patients’ age and gender. Results showed that the ICI score could effectively forecast OS in all groups from the age and gender clinical features (Figures 8A–D).

## Discussion

At present, radical resection is still the main treatment method for localized BLCA, followed by intracavitary chemotherapy or immunotherapy (Cao et al., 2020). However, BLCA has the characteristic of a high recurrence rate and low survival rate (Cambier et al., 2016; Kamat et al., 2016; Sanli et al., 2017). Despite the fact that ICIs are effective against advanced urothelial malignancies, including BLCA, tumor reaction to ICIs is often poor and difficult to predict (Zou et al., 2016; Dermani et al., 2019). Besides, TMB is considered an important marker for predicting ICI response in a variety of tumor types. Nevertheless, the boundary between high and low TMB is yet to be properly defined (Samstein et al., 2019). As a result, finding a novel prognostic marker is critical. Instead of tumor cells, more and more attention has been paid to immune cell infiltration (ICI) recently. In this research, we combined TCGA-BLCA and GSE13507 to get common gene expression data, which contain 569 BLCA patient samples. Subsequently, based on the unsupervised clustering method, a total of 569 patient samples were divided into two different immune subtypes according to the proportion of ICI. Consensus clustering has been widely used in genome research (Șenbabaoğlu et al., 2014). Based on the DEGs between ICI cluster A and ICI cluster B, we classified the BLCA patients into two genomic clusters, namely, gene cluster A and gene cluster B, respectively. Anti-tumor cells and pro-tumor cells are two kinds of immune cells engaged in cancer local immune response (Yu and Ho, 2019; Riera -Domingo et al., 2020). Different immune cells may play different roles in different tumors (Foley et al., 2016). Our analysis results showed that the expression levels of CD8 T cells and naive B cells were up-regulated in gene cluster A, indicating a good outcome. Meanwhile, macrophage M0 was shown to be positively correlated with gene cluster B, which showed a poor prognosis. We obtained two gene signatures by different expression levels of DEGs in different gene clusters. Considering the individual heterogeneity of the immune environment, it is urgent to quantify the ICI model for individual tumors (Callari et al., 2016). In some cancers, individual-based models have been fully established to improve outcome forecasting (Bramsen et al., 2017; Komor et al., 2018). In this study, the PCA algorithm was used to separate the TCGA-BLCA and GSE13507 cohorts into two ICI score groups. The high score group has a better prognosis than the low one. Through GSEA, we found that the genes implicated in the immune activation pathway, such as fatty acid metabolism and PPAR signaling pathways, were significantly abundant in the high ICI score group. In various ICI score groups, we analyzed the levels of immune activation–related signal and immune-checkpoint–related signal. In the low ICI score group, the expression levels of CD274, CTLA4, HAVCR2, LAG3, PDCD1, CD8A, CXCL10, CXCL9, GZMA, GZMB, IFNG, PRF1, and TNF were up-regulated, except TBX2. Besides, we explored the mutation frequency of some driving genes in different ICI score groups. The results revealed that in the low ICI score group, the frequency of TP53 mutations was increased. All of these studies revealed that the ICI score was adversely linked with tumor malignancy from different perspectives. Since the neoantigen load could be easily detected and evaluated by TMB, it has been proved to be an indicator of clinical benefit and a prognostic factor for predicting ICI response. Our analysis shows that high TMB has better OS performance in BLCA. Finally, we found that the high TMB combined with the high ICI score has a higher survival rate than others. ICI score can effectively predict the OS of age and gender groups. However, all results of this study were obtained retrospectively based on public databases, which requires further prospective validation.

## Conclusion

We comprehensively analyzed the BLCA ICI landscape, providing a clear picture of the anti-/pro-tumor immune response regulation in BLCA. The variation of ICI patterns is related to tumor heterogeneity. As a result, this discovery has significant clinical implications for the systematic evaluation of tumor ICI patterns. Our results revealed that the ICI score could be served as an efficient predictive marker which is different from TMB. These findings may provide a new method to predict the prognosis of BLCA.

## Data Availability

The original contributions presented in the study are included in the article/Supplementary Material; further inquiries can be directed to the corresponding author.
