# Supplementary material for: The Immune Cell Infiltration Patterns and Characterization Score in Bladder Cancer to Identify Prognosis
Source: Front Genet. 2022 Jun 21;13:852708. doi: 10.3389/fgene.2022.852708 (PMC9255635; doi:10.3389/fgene.2022.852708)
Supplement: Supplementary file 1 [file Table4.DOC]

**Supplementary Table 5:** Results of gene clusters according to DEGs.

| **ID** | **Gene clusters** | TCGA_TCGA-GU-AATP | A |
| --- | --- | --- | --- |
| TCGA_TCGA-ZF-A9R7 | A | TCGA_TCGA-DK-AA6X | A |
| TCGA_TCGA-FD-A3SO | B | TCGA_TCGA-XF-A9T3 | A |
| TCGA_TCGA-E7-A97P | B | TCGA_TCGA-PQ-A6FN | B |
| TCGA_TCGA-GU-A767 | A | TCGA_TCGA-CF-A1HR | A |
| TCGA_TCGA-4Z-AA81 | B | TCGA_TCGA-E7-A3X6 | B |
| TCGA_TCGA-E7-A85H | A | TCGA_TCGA-DK-A3WW | B |
| TCGA_TCGA-G2-AA3C | A | TCGA_TCGA-XF-AAMY | A |
| TCGA_TCGA-E7-A8O8 | A | TCGA_TCGA-K4-A3WS | A |
| TCGA_TCGA-XF-A9SM | B | TCGA_TCGA-GC-A3YS | B |
| TCGA_TCGA-DK-A3IN | B | TCGA_TCGA-DK-A1AF | B |
| TCGA_TCGA-2F-A9KR | B | TCGA_TCGA-LT-A5Z6 | A |
| TCGA_TCGA-E5-A4U1 | A | TCGA_TCGA-GC-A4ZW | A |
| TCGA_TCGA-E7-A3Y1 | A | TCGA_TCGA-K4-A4AB | A |
| TCGA_TCGA-FD-A6TC | A | TCGA_TCGA-DK-AA74 | B |
| TCGA_TCGA-FD-A5BV | A | TCGA_TCGA-G2-A2EO | A |
| TCGA_TCGA-DK-A2I4 | A | TCGA_TCGA-DK-A1AG | A |
| TCGA_TCGA-UY-A78M | A | TCGA_TCGA-GV-A3JZ | A |
| TCGA_TCGA-4Z-AA7R | A | TCGA_TCGA-XF-A9SV | A |
| TCGA_TCGA-HQ-A5NE | B | TCGA_TCGA-UY-A78K | A |
| TCGA_TCGA-XF-AAMT | B | TCGA_TCGA-FD-A6TH | A |
| TCGA_TCGA-XF-A9SU | A | TCGA_TCGA-DK-AA6W | A |
| TCGA_TCGA-CF-A47S | A | TCGA_TCGA-CF-A3MG | A |
| TCGA_TCGA-FD-A6TK | A | TCGA_TCGA-DK-A3X1 | A |
| TCGA_TCGA-E7-A5KF | A | TCGA_TCGA-DK-A1A6 | A |
| TCGA_TCGA-UY-A78L | B | TCGA_TCGA-XF-AAMG | A |
| TCGA_TCGA-S5-A6DX | A | TCGA_TCGA-E7-A541 | A |
| TCGA_TCGA-XF-A8HI | A | TCGA_TCGA-XF-A8HE | B |
| TCGA_TCGA-XF-A9T5 | A | TCGA_TCGA-DK-AA6Q | B |
| TCGA_TCGA-XF-A9SX | A | TCGA_TCGA-UY-A78N | A |
| TCGA_TCGA-G2-A2EK | A | TCGA_TCGA-DK-AA75 | A |
| TCGA_TCGA-ZF-A9RE | B | TCGA_TCGA-ZF-AA5P | A |
| TCGA_TCGA-E7-A7DV | A | TCGA_TCGA-GC-A3WC | B |
| TCGA_TCGA-XF-AAN1 | A | TCGA_TCGA-K4-A4AC | B |
| TCGA_TCGA-DK-AA6P | A | TCGA_TCGA-FD-A3SL | A |
| TCGA_TCGA-CF-A47Y | A | TCGA_TCGA-K4-A5RH | A |
| TCGA_TCGA-CF-A3MI | A | TCGA_TCGA-FD-A5C1 | B |
| TCGA_TCGA-G2-A2EL | A | TCGA_TCGA-XF-AAN7 | A |
| TCGA_TCGA-XF-AAMZ | A | TCGA_TCGA-2F-A9KO | A |
| TCGA_TCGA-DK-AA6L | B | TCGA_TCGA-DK-A3IM | B |
| TCGA_TCGA-XF-A9T4 | B | TCGA_TCGA-FD-A3SS | B |
| TCGA_TCGA-K4-A6MB | B | TCGA_TCGA-UY-A9PE | A |
| TCGA_TCGA-UY-A8OB | B | TCGA_TCGA-GV-A40E | A |
| TCGA_TCGA-DK-A1A5 | A | TCGA_TCGA-BT-A2LD | A |
| TCGA_TCGA-2F-A9KQ | A | TCGA_TCGA-UY-A9PD | A |
| TCGA_TCGA-SY-A9G0 | A | TCGA_TCGA-DK-A3IQ | A |
| TCGA_TCGA-GV-A40G | A | TCGA_TCGA-E7-A4XJ | A |
| TCGA_TCGA-FD-A5BX | A | TCGA_TCGA-XF-A9T6 | A |
| TCGA_TCGA-BL-A13J | B | TCGA_TCGA-FD-A43Y | B |
| TCGA_TCGA-ZF-AA54 | A | TCGA_TCGA-S5-AA26 | A |
| TCGA_TCGA-GU-A766 | B | TCGA_TCGA-YC-A9TC | A |
| TCGA_TCGA-LT-A8JT | A | TCGA_TCGA-GV-A3QG | A |
| TCGA_TCGA-FJ-A3Z9 | A | TCGA_TCGA-K4-A6FZ | B |
| TCGA_TCGA-DK-A1AC | A | TCGA_TCGA-KQ-A41R | A |
| TCGA_TCGA-C4-A0F1 | B | TCGA_TCGA-DK-AA6M | B |
| TCGA_TCGA-G2-A2ES | B | TCGA_TCGA-ZF-AA52 | A |
| TCGA_TCGA-XF-AAN4 | A | TCGA_TCGA-K4-A5RJ | A |
| TCGA_TCGA-4Z-AA83 | A | TCGA_TCGA-HQ-A5ND | B |
| TCGA_TCGA-4Z-AA7N | A | TCGA_TCGA-BT-A3PK | B |
| TCGA_TCGA-HQ-A2OE | A | TCGA_TCGA-FD-A3N6 | B |
| TCGA_TCGA-UY-A8OC | B | TCGA_TCGA-UY-A78P | B |
| TCGA_TCGA-GV-A3JV | A | TCGA_TCGA-BT-A20Q | A |
| TCGA_TCGA-XF-A9T8 | B | TCGA_TCGA-CU-A0YR | A |
| TCGA_TCGA-KQ-A41S | A | TCGA_TCGA-SY-A9G5 | A |
| TCGA_TCGA-CU-A3YL | A | TCGA_TCGA-4Z-AA84 | A |
| TCGA_TCGA-E7-A7XN | B | TCGA_TCGA-4Z-AA7Q | B |
| TCGA_TCGA-DK-A2HX | A | TCGA_TCGA-FD-A5BT | A |
| TCGA_TCGA-CF-A8HY | A | TCGA_TCGA-CF-A27C | A |
| TCGA_TCGA-4Z-AA7W | A | TCGA_TCGA-E7-A6MF | A |
| TCGA_TCGA-FD-A43U | A | TCGA_TCGA-FD-A6TF | A |
| TCGA_TCGA-BL-A0C8 | A | TCGA_TCGA-XF-AAMR | A |
| TCGA_TCGA-BT-A0S7 | B | TCGA_TCGA-XF-A8HF | A |
| TCGA_TCGA-ZF-A9R3 | A | TCGA_TCGA-ZF-AA4U | A |
| TCGA_TCGA-4Z-AA89 | A | TCGA_TCGA-E7-A97Q | A |
| TCGA_TCGA-GU-A42P | A | TCGA_TCGA-ZF-A9R0 | A |
| TCGA_TCGA-FD-A3NA | B | TCGA_TCGA-FD-A43S | A |
| TCGA_TCGA-E7-A5KE | A | TCGA_TCGA-XF-A8HG | A |
| TCGA_TCGA-ZF-AA4V | A | TCGA_TCGA-K4-A83P | A |
| TCGA_TCGA-ZF-AA53 | B | TCGA_TCGA-GD-A2C5 | A |
| TCGA_TCGA-ZF-A9RM | A | TCGA_TCGA-XF-AAMX | A |
| TCGA_TCGA-BL-A13I | A | TCGA_TCGA-DK-A1AE | A |
| TCGA_TCGA-CU-A72E | B | TCGA_TCGA-FJ-A3ZE | A |
| TCGA_TCGA-CF-A47W | A | TCGA_TCGA-DK-A2I1 | A |
| TCGA_TCGA-GC-A3I6 | B | TCGA_TCGA-DK-A6B0 | A |
| TCGA_TCGA-FD-A6TA | A | TCGA_TCGA-FD-A5BR | A |
| TCGA_TCGA-CF-A47X | A | TCGA_TCGA-GD-A3OP | A |
| TCGA_TCGA-ZF-AA51 | A | TCGA_TCGA-KQ-A41P | A |
| TCGA_TCGA-FD-A62O | A | TCGA_TCGA-UY-A9PH | A |
| TCGA_TCGA-BL-A3JM | A | TCGA_TCGA-FD-A5BY | B |
| TCGA_TCGA-FJ-A3ZF | A | TCGA_TCGA-K4-A3WU | A |
| TCGA_TCGA-CU-A0YO | A | TCGA_TCGA-FD-A3SJ | A |
| TCGA_TCGA-CF-A5UA | A | TCGA_TCGA-E7-A519 | A |
| TCGA_TCGA-K4-AAQO | A | TCGA_TCGA-DK-A3IU | B |
| TCGA_TCGA-FD-A3SM | A | TCGA_TCGA-XF-A9T0 | A |
| TCGA_TCGA-BL-A5ZZ | B | TCGA_TCGA-YF-AA3M | A |
| TCGA_TCGA-FD-A62N | A | TCGA_TCGA-4Z-AA7Y | A |
| TCGA_TCGA-BT-A20J | A | TCGA_TCGA-KQ-A41Q | A |
| TCGA_TCGA-FT-A61P | A | TCGA_TCGA-FD-A3SP | A |
| TCGA_TCGA-DK-AA77 | A | TCGA_TCGA-GC-A6I3 | A |
| TCGA_TCGA-ZF-AA5N | A | TCGA_TCGA-4Z-AA80 | A |
| TCGA_TCGA-DK-AA6U | A | TCGA_TCGA-G2-A3IE | A |
| TCGA_TCGA-BT-A20O | A | TCGA_TCGA-CF-A9FF | A |
| TCGA_TCGA-GD-A3OS | B | TCGA_TCGA-XF-A9ST | A |
| TCGA_TCGA-XF-A9SZ | A | TCGA_TCGA-DK-A6B5 | B |
| TCGA_TCGA-4Z-AA82 | B | TCGA_TCGA-GC-A3RC | B |
| TCGA_TCGA-DK-AA76 | A | TCGA_TCGA-GV-A3QK | A |
| TCGA_TCGA-DK-A3IL | A | TCGA_TCGA-DK-AA71 | B |
| TCGA_TCGA-E7-A678 | A | TCGA_TCGA-KQ-A41O | A |
| TCGA_TCGA-ZF-A9R1 | A | TCGA_TCGA-E7-A677 | A |
| TCGA_TCGA-XF-A9SH | A | TCGA_TCGA-DK-A6B1 | A |
| TCGA_TCGA-FD-A5BU | B | TCGA_TCGA-CF-A5U8 | A |
| TCGA_TCGA-C4-A0F7 | B | TCGA_TCGA-GU-AATO | A |
| TCGA_TCGA-C4-A0F0 | B | TCGA_TCGA-CF-A9FH | A |
| TCGA_TCGA-FD-A3N5 | B | TCGA_TCGA-FD-A6TG | A |
| TCGA_TCGA-ZF-A9R5 | A | TCGA_TCGA-4Z-AA7O | B |
| TCGA_TCGA-CU-A3KJ | B | TCGA_TCGA-GV-A3QI | A |
| TCGA_TCGA-DK-AA6S | A | TCGA_TCGA-DK-A6AV | A |
| TCGA_TCGA-BT-A42E | B | TCGA_TCGA-DK-A1A3 | A |
| TCGA_TCGA-FD-A43X | A | TCGA_TCGA-GD-A6C6 | A |
| TCGA_TCGA-ZF-AA4R | B | TCGA_TCGA-GC-A3RD | A |
| TCGA_TCGA-BT-A20T | B | TCGA_TCGA-BT-A20R | A |
| TCGA_TCGA-E7-A7DU | A | GSE13507_GSM340606 | A |
| TCGA_TCGA-DK-A3WY | A | GSE13507_GSM340607 | A |
| TCGA_TCGA-G2-A2EF | B | GSE13507_GSM340608 | A |
| TCGA_TCGA-BT-A20V | A | GSE13507_GSM340609 | A |
| TCGA_TCGA-K4-A3WV | B | GSE13507_GSM340610 | A |
| TCGA_TCGA-GU-AATQ | B | GSE13507_GSM340611 | B |
| TCGA_TCGA-CF-A3MF | A | GSE13507_GSM340612 | A |
| TCGA_TCGA-CF-A47T | A | GSE13507_GSM340613 | A |
| TCGA_TCGA-YC-A8S6 | A | GSE13507_GSM340614 | A |
| TCGA_TCGA-DK-A3IS | A | GSE13507_GSM340615 | A |
| TCGA_TCGA-XF-A9SJ | A | GSE13507_GSM340616 | A |
| TCGA_TCGA-DK-AA6T | A | GSE13507_GSM340617 | A |
| TCGA_TCGA-BT-A20N | A | GSE13507_GSM340618 | A |
| TCGA_TCGA-XF-AAMW | B | GSE13507_GSM340619 | A |
| TCGA_TCGA-BT-A3PJ | B | GSE13507_GSM340620 | A |
| TCGA_TCGA-GC-A3OO | A | GSE13507_GSM340621 | A |
| TCGA_TCGA-E7-A6ME | A | GSE13507_GSM340622 | A |
| TCGA_TCGA-XF-AAN5 | B | GSE13507_GSM340623 | A |
| TCGA_TCGA-FD-A3SN | A | GSE13507_GSM340624 | A |
| TCGA_TCGA-ZF-A9R2 | A | GSE13507_GSM340625 | A |
| TCGA_TCGA-ZF-AA4T | A | GSE13507_GSM340626 | A |
| TCGA_TCGA-E7-A4IJ | A | GSE13507_GSM340627 | A |
| TCGA_TCGA-FD-A6TE | A | GSE13507_GSM340628 | B |
| TCGA_TCGA-H4-A2HO | A | GSE13507_GSM340629 | B |
| TCGA_TCGA-FD-A6TB | A | GSE13507_GSM340630 | A |
| TCGA_TCGA-XF-AAN3 | B | GSE13507_GSM340631 | A |
| TCGA_TCGA-XF-AAMH | B | GSE13507_GSM340632 | A |
| TCGA_TCGA-FD-A3B8 | B | GSE13507_GSM340633 | A |
| TCGA_TCGA-XF-A9SY | A | GSE13507_GSM340634 | B |
| TCGA_TCGA-CU-A0YN | B | GSE13507_GSM340635 | A |
| TCGA_TCGA-FD-A5BZ | A | GSE13507_GSM340636 | A |
| TCGA_TCGA-BT-A20P | A | GSE13507_GSM340637 | A |
| TCGA_TCGA-XF-A8HD | A | GSE13507_GSM340638 | A |
| TCGA_TCGA-GV-A3JX | A | GSE13507_GSM340639 | A |
| TCGA_TCGA-H4-A2HQ | A | GSE13507_GSM340640 | A |
| TCGA_TCGA-XF-A9SP | A | GSE13507_GSM340641 | A |
| TCGA_TCGA-DK-A3IK | A | GSE13507_GSM340642 | A |
| TCGA_TCGA-C4-A0F6 | A | GSE13507_GSM340643 | A |
| TCGA_TCGA-CF-A7I0 | A | GSE13507_GSM340644 | A |
| TCGA_TCGA-FD-A3B6 | B | GSE13507_GSM340645 | B |
| TCGA_TCGA-GU-A764 | A | GSE13507_GSM340646 | A |
| TCGA_TCGA-GU-A42R | B | GSE13507_GSM340647 | A |
| TCGA_TCGA-DK-A6B6 | A | GSE13507_GSM340648 | A |
| TCGA_TCGA-R3-A69X | A | GSE13507_GSM340649 | A |
| TCGA_TCGA-5N-A9KM | A | GSE13507_GSM340650 | B |
| TCGA_TCGA-FD-A6TD | B | GSE13507_GSM340651 | A |
| TCGA_TCGA-4Z-AA7M | A | GSE13507_GSM340652 | A |
| TCGA_TCGA-ZF-AA56 | B | GSE13507_GSM340653 | A |
| TCGA_TCGA-XF-A8HH | A | GSE13507_GSM340654 | A |
| TCGA_TCGA-C4-A0EZ | A | GSE13507_GSM340655 | A |
| TCGA_TCGA-XF-AAML | A | GSE13507_GSM340656 | A |
| TCGA_TCGA-ZF-AA58 | A | GSE13507_GSM340657 | A |
| TCGA_TCGA-UY-A9PA | A | GSE13507_GSM340658 | A |
| TCGA_TCGA-G2-AA3F | A | GSE13507_GSM340659 | A |
| TCGA_TCGA-FJ-A871 | A | GSE13507_GSM340660 | A |
| TCGA_TCGA-ZF-A9RN | A | GSE13507_GSM340661 | A |
| TCGA_TCGA-GC-A3RB | A | GSE13507_GSM340662 | A |
| TCGA_TCGA-UY-A9PF | A | GSE13507_GSM340663 | B |
| TCGA_TCGA-YC-A89H | B | GSE13507_GSM340664 | A |
| TCGA_TCGA-XF-AAMQ | A | GSE13507_GSM340665 | A |
| TCGA_TCGA-FD-A3B4 | B | GSE13507_GSM340666 | A |
| TCGA_TCGA-DK-A1AB | B | GSE13507_GSM340667 | A |
| TCGA_TCGA-BT-A20X | B | GSE13507_GSM340668 | A |
| TCGA_TCGA-GV-A3JW | A | GSE13507_GSM340669 | A |
| TCGA_TCGA-UY-A8OD | A | GSE13507_GSM340670 | A |
| TCGA_TCGA-2F-A9KT | B | GSE13507_GSM340671 | A |
| TCGA_TCGA-ZF-AA5H | A | GSE13507_GSM340672 | B |
| TCGA_TCGA-XF-A9T2 | A | GSE13507_GSM340673 | A |
| TCGA_TCGA-DK-A3WX | B | GSE13507_GSM340674 | A |
| TCGA_TCGA-YF-AA3L | A | GSE13507_GSM340675 | A |
| TCGA_TCGA-BT-A2LA | A | GSE13507_GSM340676 | A |
| TCGA_TCGA-4Z-AA87 | A | GSE13507_GSM340677 | A |
| TCGA_TCGA-BT-A2LB | A | GSE13507_GSM340678 | A |
| TCGA_TCGA-XF-A8HB | A | GSE13507_GSM340679 | A |
| TCGA_TCGA-DK-A2I6 | A | GSE13507_GSM340680 | A |
| TCGA_TCGA-ZF-AA4X | A | GSE13507_GSM340681 | A |
| TCGA_TCGA-E7-A7PW | A | GSE13507_GSM340682 | A |
| TCGA_TCGA-4Z-AA7S | A | GSE13507_GSM340683 | A |
| TCGA_TCGA-CF-A3MH | A | GSE13507_GSM340684 | B |
| TCGA_TCGA-DK-A6B2 | A | GSE13507_GSM340685 | A |
| TCGA_TCGA-4Z-AA86 | B | GSE13507_GSM340686 | A |
| TCGA_TCGA-5N-A9KI | A | GSE13507_GSM340687 | B |
| TCGA_TCGA-XF-A9SL | A | GSE13507_GSM340688 | A |
| TCGA_TCGA-XF-AAN2 | B | GSE13507_GSM340689 | A |
| TCGA_TCGA-CF-A9FL | A | GSE13507_GSM340690 | A |
| TCGA_TCGA-CF-A9FM | A | GSE13507_GSM340691 | A |
| TCGA_TCGA-ZF-A9RD | B | GSE13507_GSM340692 | A |
| TCGA_TCGA-G2-AA3D | A | GSE13507_GSM340693 | A |
| TCGA_TCGA-DK-A2I2 | B | GSE13507_GSM340694 | A |
| TCGA_TCGA-FD-A62S | B | GSE13507_GSM340695 | A |
| TCGA_TCGA-MV-A51V | A | GSE13507_GSM340696 | B |
| TCGA_TCGA-E5-A2PC | A | GSE13507_GSM340697 | A |
| TCGA_TCGA-DK-A3X2 | B | GSE13507_GSM340698 | A |
| TCGA_TCGA-GD-A76B | A | GSE13507_GSM340699 | A |
| TCGA_TCGA-GC-A6I1 | B | GSE13507_GSM340700 | A |
| TCGA_TCGA-XF-A9SK | A | GSE13507_GSM340701 | A |
| TCGA_TCGA-DK-A1AD | A | GSE13507_GSM340702 | B |
| TCGA_TCGA-K4-A5RI | B | GSE13507_GSM340703 | A |
| TCGA_TCGA-FD-A3SQ | A | GSE13507_GSM340704 | A |
| TCGA_TCGA-GV-A3QF | A | GSE13507_GSM340705 | A |
| TCGA_TCGA-G2-A3VY | A | GSE13507_GSM340706 | B |
| TCGA_TCGA-XF-AAN8 | A | GSE13507_GSM340707 | A |
| TCGA_TCGA-CU-A3QU | A | GSE13507_GSM340708 | A |
| TCGA_TCGA-DK-A6AW | A | GSE13507_GSM340709 | A |
| TCGA_TCGA-GC-A3BM | A | GSE13507_GSM340710 | A |
| TCGA_TCGA-GV-A6ZA | A | GSE13507_GSM340711 | A |
| TCGA_TCGA-G2-A2EJ | B | GSE13507_GSM340712 | A |
| TCGA_TCGA-FD-A43N | A | GSE13507_GSM340713 | A |
| TCGA_TCGA-XF-AAMJ | A | GSE13507_GSM340714 | A |
| TCGA_TCGA-GU-A763 | A | GSE13507_GSM340715 | A |
| TCGA_TCGA-G2-A2EC | A | GSE13507_GSM340716 | A |
| TCGA_TCGA-FD-A3B7 | B | GSE13507_GSM340717 | A |
| TCGA_TCGA-FD-A5C0 | A | GSE13507_GSM340718 | A |
| TCGA_TCGA-FD-A5BS | A | GSE13507_GSM340719 | A |
| TCGA_TCGA-BT-A20W | A | GSE13507_GSM340720 | A |
| TCGA_TCGA-UY-A78O | A | GSE13507_GSM340721 | A |
| TCGA_TCGA-FD-A3B5 | B | GSE13507_GSM340722 | B |
| TCGA_TCGA-BT-A42C | A | GSE13507_GSM340723 | A |
| TCGA_TCGA-CF-A8HX | A | GSE13507_GSM340724 | A |
| TCGA_TCGA-PQ-A6FI | B | GSE13507_GSM340725 | B |
| TCGA_TCGA-FJ-A3Z7 | A | GSE13507_GSM340726 | A |
| TCGA_TCGA-XF-A8HC | A | GSE13507_GSM340727 | B |
| TCGA_TCGA-ZF-AA4W | B | GSE13507_GSM340728 | B |
| TCGA_TCGA-LC-A66R | B | GSE13507_GSM340729 | A |
| TCGA_TCGA-E7-A8O7 | A | GSE13507_GSM340730 | A |
| TCGA_TCGA-UY-A9PB | A | GSE13507_GSM340731 | B |
| TCGA_TCGA-FD-A6TI | A | GSE13507_GSM340732 | B |
| TCGA_TCGA-G2-AA3B | A | GSE13507_GSM340733 | B |
| TCGA_TCGA-K4-A54R | A | GSE13507_GSM340734 | A |
| TCGA_TCGA-DK-A1AA | A | GSE13507_GSM340735 | A |
| TCGA_TCGA-CU-A5W6 | A | GSE13507_GSM340736 | B |
| TCGA_TCGA-DK-AA6R | B | GSE13507_GSM340737 | A |
| TCGA_TCGA-BT-A3PH | A | GSE13507_GSM340738 | A |
| TCGA_TCGA-G2-A3IB | B | GSE13507_GSM340739 | A |
| TCGA_TCGA-2F-A9KP | A | GSE13507_GSM340740 | A |
| TCGA_TCGA-DK-A3IT | A | GSE13507_GSM340741 | A |
| TCGA_TCGA-FD-A43P | A | GSE13507_GSM340742 | A |
| TCGA_TCGA-DK-A3IV | A | GSE13507_GSM340743 | A |
| TCGA_TCGA-2F-A9KW | A | GSE13507_GSM340744 | A |
| TCGA_TCGA-GU-A762 | A | GSE13507_GSM340745 | A |
| TCGA_TCGA-XF-AAME | B | GSE13507_GSM340746 | A |
| TCGA_TCGA-XF-AAN0 | A | GSE13507_GSM340747 | A |
| TCGA_TCGA-FD-A62P | B | GSE13507_GSM340748 | A |
| TCGA_TCGA-CF-A47V | A | GSE13507_GSM340749 | A |
| TCGA_TCGA-XF-A9SI | A | GSE13507_GSM340750 | A |
| TCGA_TCGA-GU-A42Q | B | GSE13507_GSM340751 | A |
| TCGA_TCGA-BT-A42F | B | GSE13507_GSM340752 | A |
| TCGA_TCGA-ZF-A9RF | A | GSE13507_GSM340753 | A |
| TCGA_TCGA-KQ-A41N | A | GSE13507_GSM340754 | A |
| TCGA_TCGA-GD-A3OQ | B | GSE13507_GSM340755 | A |
| TCGA_TCGA-BT-A20U | B | GSE13507_GSM340756 | A |
| TCGA_TCGA-XF-A9SW | A | GSE13507_GSM340757 | A |
| TCGA_TCGA-FD-A3B3 | B | GSE13507_GSM340758 | A |
| TCGA_TCGA-ZF-A9RC | A | GSE13507_GSM340759 | A |
| TCGA_TCGA-E7-A6MD | A | GSE13507_GSM340760 | A |
| TCGA_TCGA-E5-A4TZ | B | GSE13507_GSM340761 | A |
| TCGA_TCGA-ZF-A9R4 | A | GSE13507_GSM340762 | A |
| TCGA_TCGA-GV-A3QH | A | GSE13507_GSM340763 | A |
| TCGA_TCGA-BT-A0YX | B | GSE13507_GSM340764 | A |
| TCGA_TCGA-FT-A3EE | A | GSE13507_GSM340765 | A |
| TCGA_TCGA-ZF-A9RL | A | GSE13507_GSM340766 | A |
| TCGA_TCGA-ZF-A9R9 | A | GSE13507_GSM340767 | A |
| TCGA_TCGA-DK-A1A7 | A | GSE13507_GSM340768 | A |
| TCGA_TCGA-FD-A3SR | A | GSE13507_GSM340769 | B |
