# Supplementary material for: The Immune Cell Infiltration Patterns and Characterization Score in Bladder Cancer to Identify Prognosis
Source: Front Genet. 2022 Jun 21;13:852708. doi: 10.3389/fgene.2022.852708 (PMC9255635; doi:10.3389/fgene.2022.852708)
Supplement: Supplementary file 2 [file Table6.DOC]

**Supplementary Table 7:** Results of high and low ICI score groups of BLCA patients.

| id | futime | fustat | score | sampleType | group |
| --- | --- | --- | --- | --- | --- |
| TCGA-ZF-A9R7 | 1.821917808 | 0 | 0.141098499 | TCGA | High |
| TCGA-FD-A3SO | 0.460273973 | 1 | -2.405915629 | TCGA | High |
| TCGA-E7-A97P | 1.197260274 | 1 | -6.019003684 | TCGA | Low |
| TCGA-GU-A767 | 0.394520548 | 1 | 1.026730346 | TCGA | High |
| TCGA-4Z-AA81 | 3.479452055 | 1 | -0.824266405 | TCGA | High |
| TCGA-E7-A85H | 1.079452055 | 0 | 2.403088123 | TCGA | High |
| TCGA-G2-AA3C | 0.578082192 | 1 | 4.865791272 | TCGA | High |
| TCGA-E7-A8O8 | 0.035616438 | 0 | 1.603897956 | TCGA | High |
| TCGA-XF-A9SM | 2.871232877 | 0 | -2.888224545 | TCGA | High |
| TCGA-DK-A3IN | 0.684931507 | 1 | -2.342355226 | TCGA | High |
| TCGA-2F-A9KR | 8.720547945 | 1 | -4.367525889 | TCGA | High |
| TCGA-E5-A4U1 | 3.235616438 | 0 | 1.215576608 | TCGA | High |
| TCGA-E7-A3Y1 | 0.446575342 | 0 | 2.848184664 | TCGA | High |
| TCGA-FD-A6TC | 0.512328767 | 0 | 0.807855683 | TCGA | High |
| TCGA-FD-A5BV | 0.446575342 | 1 | 1.157920198 | TCGA | High |
| TCGA-DK-A2I4 | 10.50684932 | 0 | 3.70833122 | TCGA | High |
| TCGA-UY-A78M | 1.890410959 | 1 | -1.833454837 | TCGA | High |
| TCGA-4Z-AA7R | 1.430136986 | 1 | 4.817124709 | TCGA | High |
| TCGA-HQ-A5NE | 1.01369863 | 1 | -5.84825669 | TCGA | Low |
| TCGA-XF-AAMT | 0.246575342 | 1 | -3.977241538 | TCGA | High |
| TCGA-XF-A9SU | 0.498630137 | 1 | 1.591157969 | TCGA | High |
| TCGA-CF-A47S | 0.912328767 | 0 | 1.709756159 | TCGA | High |
| TCGA-FD-A6TK | 0.904109589 | 0 | -0.454129405 | TCGA | High |
| TCGA-E7-A5KF | 0.054794521 | 0 | 2.799999673 | TCGA | High |
| TCGA-UY-A78L | 3.087671233 | 0 | -3.76157389 | TCGA | High |
| TCGA-S5-A6DX | 0.153424658 | 1 | -1.781821797 | TCGA | High |
| TCGA-XF-A8HI | 1.490410959 | 1 | 0.981320037 | TCGA | High |
| TCGA-XF-A9T5 | 5.553424658 | 0 | 2.847994914 | TCGA | High |
| TCGA-XF-A9SX | 1.969863014 | 1 | 4.625947591 | TCGA | High |
| TCGA-G2-A2EK | 1.328767123 | 0 | 1.691628526 | TCGA | High |
| TCGA-ZF-A9RE | 0.290410959 | 1 | -6.039148593 | TCGA | Low |
| TCGA-E7-A7DV | 0.101369863 | 0 | 3.865347758 | TCGA | High |
| TCGA-XF-AAN1 | 2.578082192 | 1 | 2.156890142 | TCGA | High |
| TCGA-DK-AA6P | 1.252054795 | 0 | 3.163488756 | TCGA | High |
| TCGA-CF-A47Y | 1.021917808 | 0 | 4.34419414 | TCGA | High |
| TCGA-CF-A3MI | 1.01369863 | 0 | 2.2352896 | TCGA | High |
| TCGA-G2-A2EL | 2.243835616 | 1 | 2.593209727 | TCGA | High |
| TCGA-XF-AAMZ | 3.693150685 | 1 | 1.967344863 | TCGA | High |
| TCGA-DK-AA6L | 3.18630137 | 1 | -3.25489697 | TCGA | High |
| TCGA-XF-A9T4 | 1.356164384 | 1 | -6.508522821 | TCGA | Low |
| TCGA-K4-A6MB | 1.284931507 | 0 | -5.504438088 | TCGA | Low |
| TCGA-UY-A8OB | 5.778082192 | 0 | -2.618940262 | TCGA | High |
| TCGA-DK-A1A5 | 0.178082192 | 1 | -0.171762569 | TCGA | High |
| TCGA-2F-A9KQ | 7.906849315 | 0 | 1.397152753 | TCGA | High |
| TCGA-SY-A9G0 | 2.761643836 | 1 | 2.743016301 | TCGA | High |
| TCGA-GV-A40G | 1.589041096 | 0 | 2.291850419 | TCGA | High |
| TCGA-FD-A5BX | 0.473972603 | 1 | 3.928569068 | TCGA | High |
| TCGA-BL-A13J | 0.221917808 | 1 | -3.45353803 | TCGA | High |
| TCGA-ZF-AA54 | 1.616438356 | 1 | 5.692101368 | TCGA | High |
| TCGA-GU-A766 | 1.315068493 | 0 | -3.691430899 | TCGA | High |
| TCGA-LT-A8JT | 1.756164384 | 0 | 2.471608156 | TCGA | High |
| TCGA-FJ-A3Z9 | 1.054794521 | 1 | -1.273408753 | TCGA | High |
| TCGA-DK-A1AC | 10.90684932 | 0 | 6.53005765 | TCGA | High |
| TCGA-C4-A0F1 | 0.243835616 | 0 | -6.374735642 | TCGA | Low |
| TCGA-G2-A2ES | 2.750684932 | 1 | -5.910915864 | TCGA | Low |
| TCGA-XF-AAN4 | 2.254794521 | 1 | 5.021775896 | TCGA | High |
| TCGA-4Z-AA83 | 5.545205479 | 0 | 0.779888848 | TCGA | High |
| TCGA-4Z-AA7N | 3.745205479 | 1 | 1.01405132 | TCGA | High |
| TCGA-HQ-A2OE | 3.216438356 | 0 | 2.242121919 | TCGA | High |
| TCGA-UY-A8OC | 0 | 1 | -2.20962686 | TCGA | High |
| TCGA-GV-A3JV | 1.189041096 | 1 | 2.92022961 | TCGA | High |
| TCGA-XF-A9T8 | 1.145205479 | 1 | -4.67955623 | TCGA | Low |
| TCGA-KQ-A41S | 0.095890411 | 0 | 1.704227554 | TCGA | High |
| TCGA-CU-A3YL | 2.482191781 | 0 | 1.859348513 | TCGA | High |
| TCGA-E7-A7XN | 1.17260274 | 0 | -6.133015793 | TCGA | Low |
| TCGA-DK-A2HX | 3.890410959 | 1 | 3.31844879 | TCGA | High |
| TCGA-CF-A8HY | 0.945205479 | 0 | 0.863655781 | TCGA | High |
| TCGA-4Z-AA7W | 2.301369863 | 0 | 0.265992609 | TCGA | High |
| TCGA-FD-A43U | 1.742465753 | 0 | 2.68231303 | TCGA | High |
| TCGA-BL-A0C8 | 3.339726027 | 0 | 2.683515953 | TCGA | High |
| TCGA-BT-A0S7 | 0.547945205 | 1 | -3.534336731 | TCGA | High |
| TCGA-ZF-A9R3 | 2.6 | 1 | 1.696268947 | TCGA | High |
| TCGA-4Z-AA89 | 2.819178082 | 0 | 0.048109983 | TCGA | High |
| TCGA-GU-A42P | 0.909589041 | 1 | 3.241813529 | TCGA | High |
| TCGA-FD-A3NA | 5.054794521 | 0 | -4.18981353 | TCGA | High |
| TCGA-E7-A5KE | 0.046575342 | 0 | 0.804413776 | TCGA | High |
| TCGA-ZF-AA4V | 4.947945205 | 0 | -1.935249075 | TCGA | High |
| TCGA-ZF-AA53 | 4.824657534 | 0 | -6.153149558 | TCGA | Low |
| TCGA-ZF-A9RM | 3.98630137 | 0 | 2.330469756 | TCGA | High |
| TCGA-BL-A13I | 0.610958904 | 1 | 5.640403724 | TCGA | High |
| TCGA-CU-A72E | 1.131506849 | 1 | -5.687600008 | TCGA | Low |
| TCGA-CF-A47W | 1.008219178 | 0 | 3.599943486 | TCGA | High |
| TCGA-GC-A3I6 | 1.726027397 | 1 | -6.299741421 | TCGA | Low |
| TCGA-FD-A6TA | 5.238356164 | 0 | 1.10107027 | TCGA | High |
| TCGA-CF-A47X | 1.052054795 | 0 | 3.150846931 | TCGA | High |
| TCGA-ZF-AA51 | 4.695890411 | 0 | 3.97833871 | TCGA | High |
| TCGA-FD-A62O | 0.591780822 | 1 | 0.960335249 | TCGA | High |
| TCGA-BL-A3JM | 0.561643836 | 1 | 4.531922203 | TCGA | High |
| TCGA-FJ-A3ZF | 1.435616438 | 0 | 3.949559457 | TCGA | High |
| TCGA-CU-A0YO | 0.408219178 | 1 | -0.457146552 | TCGA | High |
| TCGA-CF-A5UA | 1 | 0 | 2.10204462 | TCGA | High |
| TCGA-K4-AAQO | 0.983561644 | 0 | 0.903321176 | TCGA | High |
| TCGA-FD-A3SM | 1.498630137 | 1 | 1.512383392 | TCGA | High |
| TCGA-BL-A5ZZ | 1.032876712 | 0 | -2.234924205 | TCGA | High |
| TCGA-FD-A62N | 0.224657534 | 0 | 1.570850633 | TCGA | High |
| TCGA-BT-A20J | 1.58630137 | 1 | 1.819977606 | TCGA | High |
| TCGA-FT-A61P | 0.923287671 | 0 | 3.410835636 | TCGA | High |
| TCGA-DK-AA77 | 1.693150685 | 0 | -1.422225521 | TCGA | High |
| TCGA-ZF-AA5N | 0.460273973 | 1 | -2.101624699 | TCGA | High |
| TCGA-DK-AA6U | 1.583561644 | 0 | -0.905661853 | TCGA | High |
| TCGA-BT-A20O | 1.01369863 | 1 | 7.348981794 | TCGA | High |
| TCGA-GD-A3OS | 1.747945205 | 0 | 0.4813242 | TCGA | High |
| TCGA-XF-A9SZ | 2.353424658 | 1 | 0.251055951 | TCGA | High |
| TCGA-4Z-AA82 | 4.263013699 | 1 | -3.251839255 | TCGA | High |
| TCGA-DK-AA76 | 1.002739726 | 0 | -1.145318684 | TCGA | High |
| TCGA-DK-A3IL | 1.131506849 | 1 | 1.475301012 | TCGA | High |
| TCGA-E7-A678 | 2.18630137 | 0 | 2.954140422 | TCGA | High |
| TCGA-ZF-A9R1 | 2.117808219 | 0 | 0.701821185 | TCGA | High |
| TCGA-XF-A9SH | 5.4 | 1 | 2.085338347 | TCGA | High |
| TCGA-FD-A5BU | 1.610958904 | 0 | -6.316376962 | TCGA | Low |
| TCGA-C4-A0F7 | 0.169863014 | 1 | -2.753074407 | TCGA | High |
| TCGA-C4-A0F0 | 0.161643836 | 0 | -3.073494601 | TCGA | High |
| TCGA-FD-A3N5 | 1.876712329 | 1 | -10.85343875 | TCGA | Low |
| TCGA-ZF-A9R5 | 2.98630137 | 0 | 3.590703501 | TCGA | High |
| TCGA-CU-A3KJ | 1.539726027 | 0 | -4.20977622 | TCGA | High |
| TCGA-DK-AA6S | 13.83561644 | 0 | 0.270353323 | TCGA | High |
| TCGA-BT-A42E | 3.035616438 | 0 | -4.499872774 | TCGA | High |
| TCGA-FD-A43X | 0.301369863 | 0 | 1.639055508 | TCGA | High |
| TCGA-ZF-AA4R | 2.838356164 | 1 | -2.889046591 | TCGA | High |
| TCGA-BT-A20T | 1.24109589 | 1 | -5.106953788 | TCGA | Low |
| TCGA-E7-A7DU | 0.076712329 | 0 | 1.745574112 | TCGA | High |
| TCGA-DK-A3WY | 13.60821918 | 0 | 5.525627951 | TCGA | High |
| TCGA-G2-A2EF | 5.161643836 | 0 | -1.878582724 | TCGA | High |
| TCGA-BT-A20V | 0.421917808 | 1 | -0.347349266 | TCGA | High |
| TCGA-K4-A3WV | 1.769863014 | 0 | -0.981339219 | TCGA | High |
| TCGA-GU-AATQ | 0.583561644 | 1 | -1.121999691 | TCGA | High |
| TCGA-CF-A3MF | 1.049315068 | 0 | 3.92556977 | TCGA | High |
| TCGA-CF-A47T | 1.054794521 | 1 | -0.59119471 | TCGA | High |
| TCGA-YC-A8S6 | 0.802739726 | 0 | -1.504207353 | TCGA | High |
| TCGA-DK-A3IS | 4.189041096 | 0 | 0.201934557 | TCGA | High |
| TCGA-XF-A9SJ | 0.268493151 | 1 | 0.672092207 | TCGA | High |
| TCGA-DK-AA6T | 1.567123288 | 0 | 6.440049293 | TCGA | High |
| TCGA-BT-A20N | 2.178082192 | 1 | -1.129305102 | TCGA | High |
| TCGA-XF-AAMW | 0.693150685 | 1 | -4.651616248 | TCGA | Low |
| TCGA-BT-A3PJ | 2.161643836 | 0 | -2.768151218 | TCGA | High |
| TCGA-GC-A3OO | 1.317808219 | 0 | -1.696261907 | TCGA | High |
| TCGA-E7-A6ME | 2.002739726 | 0 | 2.098379848 | TCGA | High |
| TCGA-XF-AAN5 | 6.282191781 | 0 | -0.41331665 | TCGA | High |
| TCGA-FD-A3SN | 2.430136986 | 0 | -1.976315308 | TCGA | High |
| TCGA-ZF-A9R2 | 1.75890411 | 0 | -0.916744326 | TCGA | High |
| TCGA-ZF-AA4T | 1.64109589 | 1 | 2.963038844 | TCGA | High |
| TCGA-E7-A4IJ | 1.846575342 | 1 | 3.27372324 | TCGA | High |
| TCGA-FD-A6TE | 1.030136986 | 0 | -0.013612847 | TCGA | High |
| TCGA-H4-A2HO | 0.126027397 | 0 | 3.057031677 | TCGA | High |
| TCGA-FD-A6TB | 1.567123288 | 0 | 2.750156984 | TCGA | High |
| TCGA-XF-AAN3 | 7.191780822 | 0 | -1.510661689 | TCGA | High |
| TCGA-XF-AAMH | 0.942465753 | 1 | -2.658948337 | TCGA | High |
| TCGA-FD-A3B8 | 1.052054795 | 0 | -0.798227258 | TCGA | High |
| TCGA-XF-A9SY | 1.753424658 | 0 | -0.378761977 | TCGA | High |
| TCGA-CU-A0YN | 1.076712329 | 1 | -11.37736306 | TCGA | Low |
| TCGA-FD-A5BZ | 2.287671233 | 1 | -3.285362348 | TCGA | High |
| TCGA-BT-A20P | 1.490410959 | 1 | 2.962368084 | TCGA | High |
| TCGA-XF-A8HD | 8.120547945 | 0 | 2.770095444 | TCGA | High |
| TCGA-GV-A3JX | 1.591780822 | 0 | -2.00443647 | TCGA | High |
| TCGA-H4-A2HQ | 1.616438356 | 0 | 0.833732755 | TCGA | High |
| TCGA-XF-A9SP | 1.243835616 | 1 | 2.692487415 | TCGA | High |
| TCGA-DK-A3IK | 0.4 | 1 | 1.743065848 | TCGA | High |
| TCGA-C4-A0F6 | 1.917808219 | 0 | -2.725768347 | TCGA | High |
| TCGA-CF-A7I0 | 1.008219178 | 0 | 2.454206671 | TCGA | High |
| TCGA-FD-A3B6 | 2.753424658 | 1 | -1.056282211 | TCGA | High |
| TCGA-GU-A764 | 1.671232877 | 0 | -0.828142995 | TCGA | High |
| TCGA-GU-A42R | 1.580821918 | 1 | -2.063009616 | TCGA | High |
| TCGA-DK-A6B6 | 3.054794521 | 0 | 3.105072421 | TCGA | High |
| TCGA-R3-A69X | 1.18630137 | 0 | 2.558485754 | TCGA | High |
| TCGA-5N-A9KM | 1.452054795 | 1 | 3.890804282 | TCGA | High |
| TCGA-FD-A6TD | 1.057534247 | 1 | -1.389477646 | TCGA | High |
| TCGA-4Z-AA7M | 1.356164384 | 0 | 1.792265937 | TCGA | High |
| TCGA-ZF-AA56 | 0.709589041 | 1 | -7.178232401 | TCGA | Low |
| TCGA-XF-A8HH | 0.156164384 | 1 | 1.640700785 | TCGA | High |
| TCGA-C4-A0EZ | 0.747945205 | 1 | 0.375589217 | TCGA | High |
| TCGA-XF-AAML | 0.635616438 | 1 | 1.434162325 | TCGA | High |
| TCGA-ZF-AA58 | 4.517808219 | 0 | 2.549898094 | TCGA | High |
| TCGA-UY-A9PA | 2.936986301 | 0 | -2.764661692 | TCGA | High |
| TCGA-G2-AA3F | 2.446575342 | 0 | 1.763887346 | TCGA | High |
| TCGA-FJ-A871 | 0.745205479 | 1 | 4.860511495 | TCGA | High |
| TCGA-ZF-A9RN | 1.684931507 | 1 | 1.785051155 | TCGA | High |
| TCGA-GC-A3RB | 1.594520548 | 0 | 0.894611903 | TCGA | High |
| TCGA-UY-A9PF | 0.320547945 | 0 | 2.441683222 | TCGA | High |
| TCGA-YC-A89H | 1.569863014 | 0 | -6.808379318 | TCGA | Low |
| TCGA-XF-AAMQ | 5.964383562 | 0 | 0.526986325 | TCGA | High |
| TCGA-FD-A3B4 | 1.397260274 | 1 | -7.784983153 | TCGA | Low |
| TCGA-DK-A1AB | 1.391780822 | 1 | -3.808027133 | TCGA | High |
| TCGA-BT-A20X | 0.687671233 | 1 | -7.280497115 | TCGA | Low |
| TCGA-GV-A3JW | 1.778082192 | 0 | -0.895179169 | TCGA | High |
| TCGA-UY-A8OD | 9.402739726 | 0 | 3.601025119 | TCGA | High |
| TCGA-2F-A9KT | 6.443835616 | 0 | -3.616541874 | TCGA | High |
| TCGA-ZF-AA5H | 2.457534247 | 0 | 0.957905536 | TCGA | High |
| TCGA-XF-A9T2 | 1.575342466 | 1 | 1.681125069 | TCGA | High |
| TCGA-DK-A3WX | 0.879452055 | 1 | -7.227119827 | TCGA | Low |
| TCGA-YF-AA3L | 0.997260274 | 0 | 2.632723856 | TCGA | High |
| TCGA-BT-A2LA | 1.430136986 | 0 | 6.704130553 | TCGA | High |
| TCGA-4Z-AA87 | 3.983561644 | 0 | 2.319874665 | TCGA | High |
| TCGA-BT-A2LB | 1.347945205 | 1 | -1.710025288 | TCGA | High |
| TCGA-XF-A8HB | 3.753424658 | 0 | 0.177599365 | TCGA | High |
| TCGA-DK-A2I6 | 7.276712329 | 0 | 2.189973731 | TCGA | High |
| TCGA-ZF-AA4X | 5.6 | 0 | 0.870367996 | TCGA | High |
| TCGA-E7-A7PW | 1.139726027 | 0 | 1.631672212 | TCGA | High |
| TCGA-4Z-AA7S | 2.915068493 | 1 | 1.91729225 | TCGA | High |
| TCGA-CF-A3MH | 1.090410959 | 0 | 0.134654254 | TCGA | High |
| TCGA-DK-A6B2 | 1.306849315 | 0 | 2.074144387 | TCGA | High |
| TCGA-4Z-AA86 | 0.852054795 | 1 | -5.163418922 | TCGA | Low |
| TCGA-5N-A9KI | 0.208219178 | 1 | 2.447918777 | TCGA | High |
| TCGA-XF-A9SL | 5.534246575 | 1 | 4.145170528 | TCGA | High |
| TCGA-XF-AAN2 | 5.120547945 | 1 | -6.345662279 | TCGA | Low |
| TCGA-CF-A9FL | 1.547945205 | 1 | 0.608765408 | TCGA | High |
| TCGA-CF-A9FM | 1.090410959 | 0 | 1.004061993 | TCGA | High |
| TCGA-ZF-A9RD | 1.117808219 | 1 | -9.4062266 | TCGA | Low |
| TCGA-G2-AA3D | 5.860273973 | 0 | 1.024754449 | TCGA | High |
| TCGA-DK-A2I2 | 0.649315068 | 1 | -5.577372078 | TCGA | Low |
| TCGA-FD-A62S | 1.112328767 | 1 | -3.305583437 | TCGA | High |
| TCGA-MV-A51V | 1.123287671 | 0 | 3.070448345 | TCGA | High |
| TCGA-E5-A2PC | 3.632876712 | 0 | 1.268349736 | TCGA | High |
| TCGA-DK-A3X2 | 1.498630137 | 1 | -4.934563054 | TCGA | Low |
| TCGA-GD-A76B | 0.61369863 | 0 | 2.160130623 | TCGA | High |
| TCGA-GC-A6I1 | 0 | 0 | -4.859477256 | TCGA | Low |
| TCGA-XF-A9SK | 1.331506849 | 1 | -0.186620894 | TCGA | High |
| TCGA-DK-A1AD | 9.369863014 | 0 | 1.027585746 | TCGA | High |
| TCGA-K4-A5RI | 0.975342466 | 1 | -2.517415322 | TCGA | High |
| TCGA-FD-A3SQ | 3.898630137 | 1 | 1.062741685 | TCGA | High |
| TCGA-GV-A3QF | 1.690410959 | 1 | 3.145722178 | TCGA | High |
| TCGA-G2-A3VY | 1.468493151 | 0 | 2.42228085 | TCGA | High |
| TCGA-XF-AAN8 | 0.323287671 | 1 | 5.786379265 | TCGA | High |
| TCGA-CU-A3QU | 0.432876712 | 0 | 2.214934124 | TCGA | High |
| TCGA-DK-A6AW | 4.44109589 | 0 | 3.070480916 | TCGA | High |
| TCGA-GC-A3BM | 1.783561644 | 1 | -1.469730596 | TCGA | High |
| TCGA-GV-A6ZA | 1.893150685 | 0 | 3.099835871 | TCGA | High |
| TCGA-G2-A2EJ | 4 | 0 | -11.14870704 | TCGA | Low |
| TCGA-FD-A43N | 1.915068493 | 0 | -0.727638276 | TCGA | High |
| TCGA-XF-AAMJ | 4.575342466 | 1 | 2.900576021 | TCGA | High |
| TCGA-GU-A763 | 2.731506849 | 0 | 2.579097698 | TCGA | High |
| TCGA-G2-A2EC | 1.906849315 | 1 | 2.647235719 | TCGA | High |
| TCGA-FD-A3B7 | 0.334246575 | 1 | -0.419187521 | TCGA | High |
| TCGA-FD-A5C0 | 1.506849315 | 1 | -1.471618972 | TCGA | High |
| TCGA-FD-A5BS | 4.490410959 | 0 | 4.892110384 | TCGA | High |
| TCGA-BT-A20W | 0.695890411 | 1 | 2.250786993 | TCGA | High |
| TCGA-UY-A78O | 6.334246575 | 0 | 1.905235592 | TCGA | High |
| TCGA-FD-A3B5 | 0.745205479 | 1 | -12.38485121 | TCGA | Low |
| TCGA-BT-A42C | 2.391780822 | 0 | -1.293578395 | TCGA | High |
| TCGA-CF-A8HX | 0.945205479 | 0 | 3.148148257 | TCGA | High |
| TCGA-PQ-A6FI | 1.019178082 | 0 | -6.624846131 | TCGA | Low |
| TCGA-FJ-A3Z7 | 2.589041096 | 0 | 4.999093566 | TCGA | High |
| TCGA-XF-A8HC | 0.547945205 | 1 | 3.120489458 | TCGA | High |
| TCGA-ZF-AA4W | 5.01369863 | 0 | -7.052666264 | TCGA | Low |
| TCGA-LC-A66R | 1.276712329 | 0 | -3.113631389 | TCGA | High |
| TCGA-E7-A8O7 | 1.276712329 | 0 | 1.173000822 | TCGA | High |
| TCGA-UY-A9PB | 2.463013699 | 0 | -0.074093523 | TCGA | High |
| TCGA-FD-A6TI | 0.805479452 | 1 | 0.268176394 | TCGA | High |
| TCGA-G2-AA3B | 5.501369863 | 0 | 0.257674893 | TCGA | High |
| TCGA-K4-A54R | 2.306849315 | 0 | 0.870893605 | TCGA | High |
| TCGA-DK-A1AA | 1.583561644 | 0 | -1.66462336 | TCGA | High |
| TCGA-CU-A5W6 | 0.153424658 | 1 | -2.507809447 | TCGA | High |
| TCGA-DK-AA6R | 13.8109589 | 0 | -9.036703626 | TCGA | Low |
| TCGA-BT-A3PH | 0.389041096 | 1 | -0.20435813 | TCGA | High |
| TCGA-G2-A3IB | 0.602739726 | 1 | -10.86402672 | TCGA | Low |
| TCGA-2F-A9KP | 0.997260274 | 1 | 1.65436955 | TCGA | High |
| TCGA-DK-A3IT | 1.775342466 | 0 | 0.531639705 | TCGA | High |
| TCGA-FD-A43P | 2.279452055 | 0 | 2.016320179 | TCGA | High |
| TCGA-DK-A3IV | 0.805479452 | 1 | 1.855368944 | TCGA | High |
| TCGA-2F-A9KW | 0.695890411 | 1 | 4.501522347 | TCGA | High |
| TCGA-GU-A762 | 0.635616438 | 1 | 1.328698407 | TCGA | High |
| TCGA-XF-AAME | 7.747945205 | 1 | -6.572444144 | TCGA | Low |
| TCGA-XF-AAN0 | 4.706849315 | 1 | 0.305275178 | TCGA | High |
| TCGA-FD-A62P | 0.523287671 | 1 | -4.600696599 | TCGA | Low |
| TCGA-CF-A47V | 1.038356164 | 0 | 2.954114125 | TCGA | High |
| TCGA-XF-A9SI | 6.638356164 | 0 | 4.04729035 | TCGA | High |
| TCGA-GU-A42Q | 0.942465753 | 1 | -5.674468721 | TCGA | Low |
| TCGA-BT-A42F | 2.367123288 | 0 | -3.048886722 | TCGA | High |
| TCGA-ZF-A9RF | 5.339726027 | 0 | 4.55665326 | TCGA | High |
| TCGA-KQ-A41N | 4.394520548 | 0 | 4.413970557 | TCGA | High |
| TCGA-GD-A3OQ | 0.260273973 | 0 | -5.272192582 | TCGA | Low |
| TCGA-BT-A20U | 1.246575342 | 1 | -7.75153722 | TCGA | Low |
| TCGA-XF-A9SW | 0.991780822 | 1 | 4.188076422 | TCGA | High |
| TCGA-FD-A3B3 | 2.668493151 | 1 | -7.727839505 | TCGA | Low |
| TCGA-ZF-A9RC | 7.857534247 | 0 | 1.043034497 | TCGA | High |
| TCGA-E7-A6MD | 0.353424658 | 0 | 1.869898321 | TCGA | High |
| TCGA-E5-A4TZ | 1.279452055 | 1 | -8.259032584 | TCGA | Low |
| TCGA-ZF-A9R4 | 2.523287671 | 0 | 2.373823526 | TCGA | High |
| TCGA-GV-A3QH | 0.706849315 | 1 | 2.86847311 | TCGA | High |
| TCGA-BT-A0YX | 1.095890411 | 1 | -3.887911864 | TCGA | High |
| TCGA-FT-A3EE | 0.271232877 | 1 | -0.246407492 | TCGA | High |
| TCGA-ZF-A9RL | 7.405479452 | 0 | 1.854079498 | TCGA | High |
| TCGA-ZF-A9R9 | 2.367123288 | 1 | 3.774857871 | TCGA | High |
| TCGA-DK-A1A7 | 1.534246575 | 0 | 3.255793582 | TCGA | High |
| TCGA-FD-A3SR | 1.649315068 | 1 | 3.012311103 | TCGA | High |
| TCGA-GU-AATP | 2.747945205 | 0 | 2.088897152 | TCGA | High |
| TCGA-DK-AA6X | 1.279452055 | 0 | 1.397122272 | TCGA | High |
| TCGA-XF-A9T3 | 0.18630137 | 0 | 2.746644147 | TCGA | High |
| TCGA-PQ-A6FN | 1.389041096 | 0 | -6.702996 | TCGA | Low |
| TCGA-CF-A1HR | 1.065753425 | 0 | 1.947491715 | TCGA | High |
| TCGA-E7-A3X6 | 2.476712329 | 1 | -3.025716476 | TCGA | High |
| TCGA-DK-A3WW | 1.734246575 | 0 | -5.619637746 | TCGA | Low |
| TCGA-XF-AAMY | 8.249315068 | 0 | 0.845101584 | TCGA | High |
| TCGA-K4-A3WS | 2.084931507 | 0 | 1.113788783 | TCGA | High |
| TCGA-GC-A3YS | 2.076712329 | 0 | -1.54106728 | TCGA | High |
| TCGA-DK-A1AF | 1.468493151 | 0 | -3.416263024 | TCGA | High |
| TCGA-LT-A5Z6 | 1.298630137 | 0 | 0.181882868 | TCGA | High |
| TCGA-GC-A4ZW | 0.04109589 | 0 | 4.570452863 | TCGA | High |
| TCGA-K4-A4AB | 0.208219178 | 0 | -2.441029872 | TCGA | High |
| TCGA-DK-AA74 | 4.679452055 | 0 | -2.376697995 | TCGA | High |
| TCGA-G2-A2EO | 4.942465753 | 1 | 1.218539491 | TCGA | High |
| TCGA-DK-A1AG | 1.301369863 | 0 | -1.226376381 | TCGA | High |
| TCGA-GV-A3JZ | 1.652054795 | 0 | 4.590308639 | TCGA | High |
| TCGA-XF-A9SV | 1.063013699 | 1 | 0.449120102 | TCGA | High |
| TCGA-UY-A78K | 1.468493151 | 1 | 3.627145548 | TCGA | High |
| TCGA-FD-A6TH | 0.35890411 | 1 | 1.921348116 | TCGA | High |
| TCGA-DK-AA6W | 1.136986301 | 1 | -0.456005951 | TCGA | High |
| TCGA-CF-A3MG | 1.010958904 | 0 | 0.910276058 | TCGA | High |
| TCGA-DK-A3X1 | 5.504109589 | 0 | 0.596994549 | TCGA | High |
| TCGA-DK-A1A6 | 5.534246575 | 0 | 1.592353172 | TCGA | High |
| TCGA-XF-AAMG | 9.216438356 | 0 | 5.780041857 | TCGA | High |
| TCGA-E7-A541 | 2.131506849 | 1 | 2.221758545 | TCGA | High |
| TCGA-XF-A8HE | 10.45753425 | 0 | -6.616415249 | TCGA | Low |
| TCGA-DK-AA6Q | 1.131506849 | 1 | -4.947429691 | TCGA | Low |
| TCGA-UY-A78N | 7.235616438 | 1 | 1.146565027 | TCGA | High |
| TCGA-DK-AA75 | 0.931506849 | 1 | 0.008773982 | TCGA | High |
| TCGA-ZF-AA5P | 1.019178082 | 0 | 3.540063797 | TCGA | High |
| TCGA-GC-A3WC | 1.479452055 | 0 | -1.475353085 | TCGA | High |
| TCGA-K4-A4AC | 0.761643836 | 1 | -6.195254068 | TCGA | Low |
| TCGA-FD-A3SL | 1.950684932 | 1 | 3.168674138 | TCGA | High |
| TCGA-K4-A5RH | 0.756164384 | 0 | 6.96297475 | TCGA | High |
| TCGA-FD-A5C1 | 4.909589041 | 0 | -3.579842595 | TCGA | High |
| TCGA-XF-AAN7 | 1.547945205 | 1 | 6.810002114 | TCGA | High |
| TCGA-2F-A9KO | 2.010958904 | 1 | -1.09025176 | TCGA | High |
| TCGA-DK-A3IM | 0.679452055 | 1 | -8.676500383 | TCGA | Low |
| TCGA-FD-A3SS | 1.071232877 | 1 | -2.7697461 | TCGA | High |
| TCGA-UY-A9PE | 0.517808219 | 0 | 1.025284823 | TCGA | High |
| TCGA-GV-A40E | 0.715068493 | 1 | -0.580164654 | TCGA | High |
| TCGA-BT-A2LD | 1.706849315 | 1 | 1.668168905 | TCGA | High |
| TCGA-UY-A9PD | 1.484931507 | 0 | 1.146699212 | TCGA | High |
| TCGA-DK-A3IQ | 1.476712329 | 1 | 0.996760725 | TCGA | High |
| TCGA-E7-A4XJ | 0.18630137 | 1 | 0.690894351 | TCGA | High |
| TCGA-XF-A9T6 | 0.175342466 | 0 | 0.014918624 | TCGA | High |
| TCGA-FD-A43Y | 1.298630137 | 1 | -6.572856832 | TCGA | Low |
| TCGA-S5-AA26 | 1.378082192 | 0 | -1.233138084 | TCGA | High |
| TCGA-YC-A9TC | 0.054794521 | 1 | 3.991199365 | TCGA | High |
| TCGA-K4-A6FZ | 0.150684932 | 0 | -9.34418132 | TCGA | Low |
| TCGA-KQ-A41R | 3.698630137 | 0 | 2.685639895 | TCGA | High |
| TCGA-DK-AA6M | 4.334246575 | 0 | -0.710228745 | TCGA | High |
| TCGA-ZF-AA52 | 2.950684932 | 1 | 1.775891571 | TCGA | High |
| TCGA-K4-A5RJ | 1.476712329 | 0 | 2.905010536 | TCGA | High |
| TCGA-HQ-A5ND | 0.750684932 | 1 | -2.060218784 | TCGA | High |
| TCGA-BT-A3PK | 0.830136986 | 1 | -5.655210821 | TCGA | Low |
| TCGA-FD-A3N6 | 2.331506849 | 0 | -11.84808478 | TCGA | Low |
| TCGA-UY-A78P | 6.520547945 | 0 | -4.554275524 | TCGA | High |
| TCGA-BT-A20Q | 1.624657534 | 1 | 0.912380866 | TCGA | High |
| TCGA-CU-A0YR | 1.260273973 | 1 | 0.341780295 | TCGA | High |
| TCGA-SY-A9G5 | 3.249315068 | 0 | -1.283732154 | TCGA | High |
| TCGA-4Z-AA84 | 1.260273973 | 0 | 3.813140079 | TCGA | High |
| TCGA-4Z-AA7Q | 1.397260274 | 1 | 0.323429609 | TCGA | High |
| TCGA-FD-A5BT | 0.898630137 | 1 | 4.349566631 | TCGA | High |
| TCGA-CF-A27C | 1.164383562 | 0 | 2.300297654 | TCGA | High |
| TCGA-E7-A6MF | 2.054794521 | 0 | 2.165305848 | TCGA | High |
| TCGA-FD-A6TF | 0.189041096 | 1 | -0.470052138 | TCGA | High |
| TCGA-XF-AAMR | 7.643835616 | 0 | 1.22654001 | TCGA | High |
| TCGA-XF-A8HF | 8.093150685 | 1 | 1.605758719 | TCGA | High |
| TCGA-ZF-AA4U | 0.717808219 | 1 | -0.944077973 | TCGA | High |
| TCGA-E7-A97Q | 0.673972603 | 1 | 2.293243817 | TCGA | High |
| TCGA-ZF-A9R0 | 1.863013699 | 1 | 2.504036039 | TCGA | High |
| TCGA-FD-A43S | 1.246575342 | 0 | 1.559768047 | TCGA | High |
| TCGA-XF-A8HG | 1.279452055 | 1 | 3.08812415 | TCGA | High |
| TCGA-K4-A83P | 1.356164384 | 0 | 3.68872789 | TCGA | High |
| TCGA-GD-A2C5 | 2.224657534 | 0 | 2.616796919 | TCGA | High |
| TCGA-XF-AAMX | 0.564383562 | 1 | 1.861502232 | TCGA | High |
| TCGA-DK-A1AE | 1.345205479 | 0 | 0.344161424 | TCGA | High |
| TCGA-FJ-A3ZE | 0.887671233 | 1 | -0.127693183 | TCGA | High |
| TCGA-DK-A2I1 | 1.495890411 | 0 | 2.377247756 | TCGA | High |
| TCGA-DK-A6B0 | 6.383561644 | 0 | 2.402974221 | TCGA | High |
| TCGA-FD-A5BR | 2.22739726 | 0 | -2.275078539 | TCGA | High |
| TCGA-GD-A3OP | 0.175342466 | 0 | 2.598977374 | TCGA | High |
| TCGA-KQ-A41P | 2.997260274 | 0 | -0.786694645 | TCGA | High |
| TCGA-UY-A9PH | 4.276712329 | 0 | 0.217334997 | TCGA | High |
| TCGA-FD-A5BY | 0.687671233 | 0 | -4.592029432 | TCGA | High |
| TCGA-K4-A3WU | 0.287671233 | 0 | -0.511874196 | TCGA | High |
| TCGA-FD-A3SJ | 2.024657534 | 1 | 3.316103481 | TCGA | High |
| TCGA-E7-A519 | 1.391780822 | 0 | 2.24736113 | TCGA | High |
| TCGA-DK-A3IU | 1.934246575 | 1 | -0.534207428 | TCGA | High |
| TCGA-XF-A9T0 | 2.189041096 | 0 | -0.260494124 | TCGA | High |
| TCGA-YF-AA3M | 1.136986301 | 0 | -1.669816576 | TCGA | High |
| TCGA-4Z-AA7Y | 4.169863014 | 0 | -0.97182601 | TCGA | High |
| TCGA-KQ-A41Q | 0.989041096 | 0 | 2.40452728 | TCGA | High |
| TCGA-FD-A3SP | 2.145205479 | 0 | 2.488287952 | TCGA | High |
| TCGA-GC-A6I3 | 0 | 0 | 2.925428542 | TCGA | High |
| TCGA-4Z-AA80 | 0.052054795 | 1 | 1.671174989 | TCGA | High |
| TCGA-G2-A3IE | 1.676712329 | 1 | 2.034286301 | TCGA | High |
| TCGA-CF-A9FF | 0.989041096 | 0 | 2.513912132 | TCGA | High |
| TCGA-XF-A9ST | 0.350684932 | 1 | 4.227127373 | TCGA | High |
| TCGA-DK-A6B5 | 4.224657534 | 0 | -4.703733668 | TCGA | Low |
| TCGA-GC-A3RC | 1.326027397 | 0 | -9.476561898 | TCGA | Low |
| TCGA-GV-A3QK | 2.279452055 | 0 | 2.36344465 | TCGA | High |
| TCGA-DK-AA71 | 1.136986301 | 0 | -3.348740444 | TCGA | High |
| TCGA-KQ-A41O | 4.21369863 | 0 | 3.790279895 | TCGA | High |
| TCGA-E7-A677 | 2.246575342 | 0 | -0.175104376 | TCGA | High |
| TCGA-DK-A6B1 | 5.61369863 | 0 | 3.861594237 | TCGA | High |
| TCGA-CF-A5U8 | 1.093150685 | 0 | 2.85122743 | TCGA | High |
| TCGA-GU-AATO | 0.887671233 | 1 | 2.056831395 | TCGA | High |
| TCGA-CF-A9FH | 0.175342466 | 0 | 1.997595918 | TCGA | High |
| TCGA-FD-A6TG | 0.254794521 | 1 | 1.2068512 | TCGA | High |
| TCGA-4Z-AA7O | 1.402739726 | 0 | -3.806011249 | TCGA | High |
| TCGA-GV-A3QI | 3.04109589 | 0 | 2.849619545 | TCGA | High |
| TCGA-DK-A6AV | 5.347945205 | 0 | 2.361080594 | TCGA | High |
| TCGA-DK-A1A3 | 1.821917808 | 1 | 3.608313884 | TCGA | High |
| TCGA-GD-A6C6 | 0.183561644 | 0 | 2.477315298 | TCGA | High |
| TCGA-GC-A3RD | 1.17260274 | 0 | -3.982866993 | TCGA | High |
| TCGA-BT-A20R | 0.421917808 | 1 | 2.736250281 | TCGA | High |
| GSM340606 | 11.25780822 | 0 | 2.961902002 | GSE13507 | High |
| GSM340607 | 11.20849315 | 0 | 0.266267719 | GSE13507 | High |
| GSM340608 | 2.210958904 | 1 | 0.719428041 | GSE13507 | High |
| GSM340609 | 7.156438356 | 1 | 0.953498947 | GSE13507 | High |
| GSM340610 | 1.257534247 | 1 | -0.216167695 | GSE13507 | High |
| GSM340611 | 1.523013699 | 1 | -2.307620237 | GSE13507 | High |
| GSM340612 | 10.67917808 | 0 | 2.493850374 | GSE13507 | High |
| GSM340613 | 1.033150685 | 1 | -1.780377334 | GSE13507 | High |
| GSM340614 | 10.60027397 | 0 | -0.201169212 | GSE13507 | High |
| GSM340615 | 10.27643836 | 0 | 0.379846823 | GSE13507 | High |
| GSM340616 | 10.29616438 | 0 | -1.170799064 | GSE13507 | High |
| GSM340617 | 10.73424658 | 0 | 1.299718003 | GSE13507 | High |
| GSM340618 | 0.084657534 | 1 | 0.849942343 | GSE13507 | High |
| GSM340619 | 10.65780822 | 0 | 0.83406874 | GSE13507 | High |
| GSM340620 | 1.370136986 | 1 | 1.80887541 | GSE13507 | High |
| GSM340621 | 10.49013699 | 0 | 0.849714701 | GSE13507 | High |
| GSM340622 | 10.23534247 | 0 | 0.949806412 | GSE13507 | High |
| GSM340623 | 0.257260274 | 1 | 0.074371128 | GSE13507 | High |
| GSM340624 | 5.41890411 | 1 | 0.654329987 | GSE13507 | High |
| GSM340625 | 0.265479452 | 1 | 1.047333414 | GSE13507 | High |
| GSM340626 | 9.912328767 | 0 | 1.736004791 | GSE13507 | High |
| GSM340627 | 1.863287671 | 1 | 1.518015296 | GSE13507 | High |
| GSM340628 | 9.904109589 | 0 | -11.133512 | GSE13507 | Low |
| GSM340629 | 4.144931507 | 1 | -2.434392595 | GSE13507 | High |
| GSM340630 | 5.449315068 | 1 | -1.050036402 | GSE13507 | High |
| GSM340631 | 9.876986301 | 0 | 1.646609981 | GSE13507 | High |
| GSM340632 | 9.912328767 | 0 | -0.138494677 | GSE13507 | High |
| GSM340633 | 1.197534247 | 1 | -3.72958209 | GSE13507 | High |
| GSM340634 | 9.520273973 | 0 | -1.569133354 | GSE13507 | High |
| GSM340635 | 8.761643836 | 0 | 1.311131173 | GSE13507 | High |
| GSM340636 | 9.961643836 | 0 | 1.585071073 | GSE13507 | High |
| GSM340637 | 8.054794521 | 1 | 2.09464992 | GSE13507 | High |
| GSM340638 | 5.509315068 | 1 | 1.927187793 | GSE13507 | High |
| GSM340639 | 8.196986301 | 0 | 0.03861529 | GSE13507 | High |
| GSM340640 | 4.134246575 | 1 | 0.452108065 | GSE13507 | High |
| GSM340641 | 8.07369863 | 0 | 0.506020583 | GSE13507 | High |
| GSM340642 | 8.714794521 | 0 | 1.003984543 | GSE13507 | High |
| GSM340643 | 7.832876712 | 0 | 2.072007065 | GSE13507 | High |
| GSM340644 | 6.610684932 | 1 | 2.676388898 | GSE13507 | High |
| GSM340645 | 2.934246575 | 1 | -4.520469519 | GSE13507 | High |
| GSM340646 | 4.255068493 | 1 | 1.31157467 | GSE13507 | High |
| GSM340647 | 7.629863014 | 0 | 1.129671894 | GSE13507 | High |
| GSM340648 | 7.457260274 | 0 | 0.738280486 | GSE13507 | High |
| GSM340649 | 7.435890411 | 0 | 0.393157534 | GSE13507 | High |
| GSM340650 | 7.074246575 | 0 | -3.482154769 | GSE13507 | High |
| GSM340651 | 11.09342466 | 1 | 0.435626413 | GSE13507 | High |
| GSM340652 | 5.41890411 | 1 | 1.070110489 | GSE13507 | High |
| GSM340653 | 2.123013699 | 1 | -0.600064353 | GSE13507 | High |
| GSM340654 | 0.844109589 | 1 | 2.917117721 | GSE13507 | High |
| GSM340655 | 7.021643836 | 1 | 0.198794463 | GSE13507 | High |
| GSM340656 | 0.260547945 | 1 | -0.017608535 | GSE13507 | High |
| GSM340657 | 6.87369863 | 0 | 1.91135319 | GSE13507 | High |
| GSM340658 | 6.87369863 | 0 | 1.169699382 | GSE13507 | High |
| GSM340659 | 5.829863014 | 1 | 0.37067846 | GSE13507 | High |
| GSM340660 | 6.709315068 | 0 | 6.492336624 | GSE13507 | High |
| GSM340661 | 6.591780822 | 0 | 0.069564577 | GSE13507 | High |
| GSM340662 | 6.479178082 | 0 | 2.674791429 | GSE13507 | High |
| GSM340663 | 6.470958904 | 0 | -4.504755465 | GSE13507 | High |
| GSM340664 | 5.813424658 | 1 | 0.812947286 | GSE13507 | High |
| GSM340665 | 0.715068493 | 1 | 0.090590429 | GSE13507 | High |
| GSM340666 | 6.191506849 | 0 | 0.68764317 | GSE13507 | High |
| GSM340667 | 6.221917808 | 0 | 1.342913905 | GSE13507 | High |
| GSM340668 | 6.175068493 | 0 | 1.875749183 | GSE13507 | High |
| GSM340669 | 6.175068493 | 0 | 0.675969308 | GSE13507 | High |
| GSM340670 | 6.096164384 | 0 | -1.072084493 | GSE13507 | High |
| GSM340671 | 1.333972603 | 1 | -0.316939354 | GSE13507 | High |
| GSM340672 | 1.24109589 | 1 | -6.785045353 | GSE13507 | Low |
| GSM340673 | 0.923013699 | 1 | 2.11257278 | GSE13507 | High |
| GSM340674 | 5.706575342 | 0 | 6.27473144 | GSE13507 | High |
| GSM340675 | 2.057260274 | 1 | -1.315377231 | GSE13507 | High |
| GSM340676 | 5.51260274 | 0 | 1.283578624 | GSE13507 | High |
| GSM340677 | 5.361369863 | 0 | 1.882571358 | GSE13507 | High |
| GSM340678 | 0.983835616 | 1 | 0.126822296 | GSE13507 | High |
| GSM340679 | 5.095890411 | 0 | 1.080386979 | GSE13507 | High |
| GSM340680 | 5.040821918 | 0 | 0.929333333 | GSE13507 | High |
| GSM340681 | 5.005479452 | 0 | 0.659638767 | GSE13507 | High |
| GSM340682 | 4.947945205 | 0 | -1.45367408 | GSE13507 | High |
| GSM340683 | 4.898630137 | 0 | -0.039346537 | GSE13507 | High |
| GSM340684 | 0.854794521 | 1 | -4.937161636 | GSE13507 | Low |
| GSM340685 | 4.920821918 | 1 | 1.144417298 | GSE13507 | High |
| GSM340686 | 4.849315068 | 0 | -0.376610283 | GSE13507 | High |
| GSM340687 | 4.827123288 | 0 | -6.618734663 | GSE13507 | Low |
| GSM340688 | 3.794794521 | 1 | 1.177556699 | GSE13507 | High |
| GSM340689 | 4.802465753 | 0 | 0.24270883 | GSE13507 | High |
| GSM340690 | 4.772876712 | 0 | 2.000979702 | GSE13507 | High |
| GSM340691 | 4.736712329 | 0 | 1.692555902 | GSE13507 | High |
| GSM340692 | 2.983561644 | 1 | 1.228376356 | GSE13507 | High |
| GSM340693 | 4.641369863 | 0 | 4.205344473 | GSE13507 | High |
| GSM340694 | 4.561643836 | 0 | 1.782572227 | GSE13507 | High |
| GSM340695 | 4.35369863 | 0 | -2.437886197 | GSE13507 | High |
| GSM340696 | 0.945205479 | 1 | -19.13358418 | GSE13507 | Low |
| GSM340697 | 1.468767123 | 1 | 2.701664639 | GSE13507 | High |
| GSM340698 | 2.344931507 | 1 | -1.387374953 | GSE13507 | High |
| GSM340699 | 1.449041096 | 1 | 5.506843183 | GSE13507 | High |
| GSM340700 | 1.265753425 | 1 | 0.128130955 | GSE13507 | High |
| GSM340701 | 3.980547945 | 0 | 2.630450891 | GSE13507 | High |
| GSM340702 | 0.369863014 | 1 | -1.413055672 | GSE13507 | High |
| GSM340703 | 3.904109589 | 0 | 1.432156548 | GSE13507 | High |
| GSM340704 | 0.429863014 | 1 | 2.724672373 | GSE13507 | High |
| GSM340705 | 3.747945205 | 0 | 3.121766143 | GSE13507 | High |
| GSM340706 | 3.709315068 | 0 | -9.348138494 | GSE13507 | Low |
| GSM340707 | 3.575342466 | 0 | 0.927196496 | GSE13507 | High |
| GSM340708 | 3.624657534 | 0 | -0.994569128 | GSE13507 | High |
| GSM340709 | 0.48739726 | 1 | 1.038332447 | GSE13507 | High |
| GSM340710 | 2.591506849 | 1 | -0.991425606 | GSE13507 | High |
| GSM340711 | 3.435616438 | 0 | 2.329918508 | GSE13507 | High |
| GSM340712 | 0.534246575 | 1 | 2.040549074 | GSE13507 | High |
| GSM340713 | 3.375616438 | 0 | 0.990879538 | GSE13507 | High |
| GSM340714 | 3.268767123 | 0 | 3.262195081 | GSE13507 | High |
| GSM340715 | 3.203013699 | 0 | -2.270229861 | GSE13507 | High |
| GSM340716 | 2.627671233 | 1 | -0.558752686 | GSE13507 | High |
| GSM340717 | 3.065753425 | 0 | 1.968551502 | GSE13507 | High |
| GSM340718 | 3.027123288 | 0 | 2.856394678 | GSE13507 | High |
| GSM340719 | 3.005753425 | 0 | 0.94380792 | GSE13507 | High |
| GSM340720 | 2.309589041 | 0 | 2.945214395 | GSE13507 | High |
| GSM340721 | 0.323013699 | 1 | 0.070554353 | GSE13507 | High |
| GSM340722 | 1.933972603 | 1 | -6.118875157 | GSE13507 | Low |
| GSM340723 | 1.24109589 | 1 | -1.706966764 | GSE13507 | High |
| GSM340724 | 2.866027397 | 0 | 0.212595993 | GSE13507 | High |
| GSM340725 | 2.813424658 | 0 | -6.626539988 | GSE13507 | Low |
| GSM340726 | 2.769863014 | 0 | -1.358383596 | GSE13507 | High |
| GSM340727 | 0.909863014 | 1 | -2.16637329 | GSE13507 | High |
| GSM340728 | 0.474246575 | 1 | -10.05530905 | GSE13507 | Low |
| GSM340729 | 2.718082192 | 0 | 0.1885872 | GSE13507 | High |
| GSM340730 | 2.701643836 | 0 | 0.072417588 | GSE13507 | High |
| GSM340731 | 0.758630137 | 1 | -21.84676434 | GSE13507 | Low |
| GSM340732 | 0.583561644 | 1 | -2.932019929 | GSE13507 | High |
| GSM340733 | 1.407945205 | 1 | -2.368140268 | GSE13507 | High |
| GSM340734 | 1.937260274 | 1 | 0.134441116 | GSE13507 | High |
| GSM340735 | 2.523287671 | 0 | 0.136815128 | GSE13507 | High |
| GSM340736 | 2.473972603 | 0 | -3.901223776 | GSE13507 | High |
| GSM340737 | 2.443561644 | 0 | 1.529922496 | GSE13507 | High |
| GSM340738 | 1.268219178 | 1 | 1.448299999 | GSE13507 | High |
| GSM340739 | 2.413972603 | 0 | 0.979410447 | GSE13507 | High |
| GSM340740 | 1.452328767 | 1 | 1.11190902 | GSE13507 | High |
| GSM340741 | 2.34 | 0 | 1.670907502 | GSE13507 | High |
| GSM340742 | 1.964383562 | 0 | 0.496348865 | GSE13507 | High |
| GSM340743 | 1.937260274 | 0 | 0.656950412 | GSE13507 | High |
| GSM340744 | 1.144931507 | 1 | -0.167407761 | GSE13507 | High |
| GSM340745 | 0.175068493 | 1 | 0.296377006 | GSE13507 | High |
| GSM340746 | 1.830410959 | 0 | 5.844181827 | GSE13507 | High |
| GSM340747 | 0.564657534 | 1 | 1.033970502 | GSE13507 | High |
| GSM340748 | 1.712054795 | 0 | 0.991591219 | GSE13507 | High |
| GSM340749 | 1.777808219 | 0 | 1.471601803 | GSE13507 | High |
| GSM340750 | 1.75890411 | 0 | 3.946848932 | GSE13507 | High |
| GSM340751 | 4.756438356 | 0 | 0.338751352 | GSE13507 | High |
| GSM340752 | 1.744931507 | 0 | -0.258015991 | GSE13507 | High |
| GSM340753 | 1.744931507 | 0 | 1.64487034 | GSE13507 | High |
| GSM340754 | 1.682465753 | 0 | 0.504470632 | GSE13507 | High |
| GSM340755 | 1.676712329 | 0 | -0.716229874 | GSE13507 | High |
| GSM340756 | 1.561643836 | 0 | -1.362094792 | GSE13507 | High |
| GSM340757 | 6.517808219 | 0 | 1.864678337 | GSE13507 | High |
| GSM340758 | 1.271506849 | 1 | -1.01936275 | GSE13507 | High |
| GSM340759 | 2.172328767 | 1 | 3.186306708 | GSE13507 | High |
| GSM340760 | 1.090684932 | 1 | 1.287348037 | GSE13507 | High |
| GSM340761 | 0.526027397 | 1 | -0.301363082 | GSE13507 | High |
| GSM340762 | 0.876986301 | 1 | -0.040200635 | GSE13507 | High |
| GSM340763 | 1.490136986 | 0 | 2.484926954 | GSE13507 | High |
| GSM340764 | 1.263287671 | 0 | 0.722490639 | GSE13507 | High |
| GSM340765 | 0.969863014 | 0 | 0.732241329 | GSE13507 | High |
| GSM340766 | 0.893424658 | 0 | 5.562330709 | GSE13507 | High |
| GSM340767 | 0.824383562 | 0 | -1.068181241 | GSE13507 | High |
| GSM340768 | 0.553150685 | 0 | -0.754679559 | GSE13507 | High |
| GSM340769 | 0.435616438 | 0 | -1.622231622 | GSE13507 | High |
