# Supplementary material for: The Immune Cell Infiltration Patterns and Characterization Score in Bladder Cancer to Identify Prognosis
Source: Front Genet. 2022 Jun 21;13:852708. doi: 10.3389/fgene.2022.852708 (PMC9255635; doi:10.3389/fgene.2022.852708)
Supplement: Supplementary file 3 [file Table8.DOC]

**Supplementary Table 1:** Relative proportions of tumor-infiltrating immune cells of BLCA patients (Filter conditions：p<0.05)

| **ID** | | **B cells naive** | | **B cells memory** | | **Plasma cells** | **T cells CD8** |
| --- | --- | --- | --- | --- | --- | --- | --- |
| TCGA_TCGA-ZF-A9R7 | | 0.033477481 | | 0.002766434 | | 0 | 0.36249071 |
| TCGA_TCGA-E7-A97P | | 0 | | 0 | | 0.025566071 | 0.3112954 |
| TCGA_TCGA-4Z-AA81 | | 0 | | 0 | | 0.045087314 | 0.261274156 |
| TCGA_TCGA-XF-A9SM | | 0.027421514 | | 0 | | 0 | 0.243546991 |
| TCGA_TCGA-DK-A3IN | | 0 | | 0 | | 0.019982466 | 0.084449586 |
| TCGA_TCGA-DK-A2I4 | | 0.013582937 | | 0 | | 0 | 0.209723448 |
| TCGA_TCGA-XF-AAMT | | 0.001362866 | | 0 | | 0.010462266 | 0 |
| TCGA_TCGA-XF-A9SU | | 0.065024108 | | 0 | | 0.13433917 | 0.088659327 |
| TCGA_TCGA-FD-A6TK | | 0.008418644 | | 0 | | 0 | 0.064356191 |
| TCGA_TCGA-XF-A9T5 | | 0.000876409 | | 0 | | 0.038228423 | 0.191273851 |
| TCGA_TCGA-XF-A9SX | | 0.01289341 | | 0 | | 0.000105715 | 0.089113747 |
| TCGA_TCGA-E7-A7DV | | 0 | | 0.024764502 | | 0 | 0.015990711 |
| TCGA_TCGA-DK-AA6L | | 0 | | 0 | | 0 | 0.068303081 |
| TCGA_TCGA-XF-A9T4 | | 0.019089092 | | 0 | | 0 | 0 |
| TCGA_TCGA-UY-A8OB | | 0 | | 0 | | 0 | 0.206515202 |
| TCGA_TCGA-DK-A1A5 | | 0 | | 0.023099835 | | 0.014107364 | 0.063450918 |
| TCGA_TCGA-SY-A9G0 | | 0.157724861 | | 0.117853627 | | 0 | 0.024530581 |
| TCGA_TCGA-FD-A5BX | | 0.005286404 | | 0 | | 0 | 0 |
| TCGA_TCGA-ZF-AA54 | | 0.005769307 | | 0 | | 0 | 0 |
| TCGA_TCGA-GU-A766 | | 0.002481795 | | 0 | | 0.018670388 | 0.146153196 |
| TCGA_TCGA-C4-A0F1 | | 0 | | 0 | | 0.221777032 | 0 |
| TCGA_TCGA-G2-A2ES | | 0 | | 0.03804289 | | 0 | 0 |
| TCGA_TCGA-XF-AAN4 | | 0.000783211 | | 0.008966923 | | 0 | 0.046578391 |
| TCGA_TCGA-4Z-AA7N | | 0.308056362 | | 0.058780386 | | 0 | 0.072367683 |
| TCGA_TCGA-UY-A8OC | | 0.006571554 | | 0 | | 0 | 0 |
| TCGA_TCGA-GV-A3JV | | 0.041498965 | | 0 | | 0.034123049 | 0.22282481 |
| TCGA_TCGA-XF-A9T8 | | 0 | | 0.083971847 | | 0 | 0.090810926 |
| TCGA_TCGA-E7-A7XN | | 0 | | 0 | | 0.017347451 | 0.299558675 |
| TCGA_TCGA-4Z-AA7W | | 0 | | 0.002895095 | | 0.017784269 | 0.296720082 |
| TCGA_TCGA-FD-A43U | | 0 | | 0.004807971 | | 0.044011159 | 0.082240174 |
| TCGA_TCGA-ZF-AA4V | | 0.012810456 | | 0 | | 0.016603339 | 0.285571599 |
| TCGA_TCGA-ZF-AA53 | | 0 | | 0.112452365 | | 0 | 0.012380485 |
| TCGA_TCGA-BL-A13I | | 0.001721083 | | 0 | | 0 | 0.011473189 |
| TCGA_TCGA-CU-A72E | | 0 | | 0 | | 0 | 0 |
| TCGA_TCGA-GC-A3I6 | | 0 | | 0 | | 0.035424202 | 0.034379221 |
| TCGA_TCGA-FD-A6TA | | 0.009586392 | | 0.022423819 | | 0.164923259 | 0.06545057 |
| TCGA_TCGA-BL-A3JM | | 0.010185339 | | 0 | | 0 | 0.009027383 |
| TCGA_TCGA-BL-A5ZZ | | 0.02761674 | | 0 | | 0 | 0.008389789 |
| TCGA_TCGA-FD-A62N | | 0.030665843 | | 0 | | 0.016935186 | 0.210360027 |
| TCGA_TCGA-BT-A20J | | 0.017266853 | | 0.003578227 | | 0 | 0.0877674 |
| TCGA_TCGA-FT-A61P | | 0 | | 0.006128335 | | 0.010437159 | 0 |
| TCGA_TCGA-BT-A20O | | 0.019370779 | | 0 | | 0 | 0.318784878 |
| TCGA_TCGA-4Z-AA82 | | 0.000729927 | | 0.008200247 | | 0.006928067 | 0 |
| TCGA_TCGA-FD-A5BU | | 0.061922669 | | 0 | | 0.046717665 | 0.037202193 |
| TCGA_TCGA-C4-A0F0 | | 0.00043956 | | 0 | | 0.001581725 | 0.151070615 |
| TCGA_TCGA-FD-A3N5 | | 0 | | 0 | | 0.071535344 | 0.008495561 |
| TCGA_TCGA-DK-AA6S | | 0 | | 0.006939097 | | 0.002370725 | 0.076740629 |
| TCGA_TCGA-BT-A42E | | 0.012482519 | | 0 | | 0 | 0.157089459 |
| TCGA_TCGA-ZF-AA4R | | 0 | | 0 | | 0.007779703 | 0.026984696 |
| TCGA_TCGA-DK-A3WY | | 0.391127541 | | 0 | | 0.064155547 | 0.140166177 |
| TCGA_TCGA-G2-A2EF | | 0.00355298 | | 0 | | 0 | 0.173521879 |
| TCGA_TCGA-GU-AATQ | | 0 | | 0.006458267 | | 0 | 0.037311275 |
| TCGA_TCGA-YC-A8S6 | | 0 | | 0 | | 0 | 0.028347981 |
| TCGA_TCGA-XF-A9SJ | | 0 | | 0.005275539 | | 0.0333527 | 0.195313784 |
| TCGA_TCGA-DK-AA6T | | 0.20457877 | | 0.476244536 | | 0.006065358 | 0.047438631 |
| TCGA_TCGA-XF-AAMW | | 0.008950046 | | 0 | | 0.027238031 | 0 |
| TCGA_TCGA-BT-A3PJ | | 0.015367777 | | 0 | | 0.003789493 | 0.100551584 |
| TCGA_TCGA-GC-A3OO | | 0.021564754 | | 0 | | 0.010558057 | 0 |
| TCGA_TCGA-XF-AAN5 | | 0 | | 0.06621806 | | 0 | 0.008672176 |
| TCGA_TCGA-FD-A6TB | | 0.225288849 | | 0 | | 0.012438325 | 0.174004426 |
| TCGA_TCGA-XF-AAN3 | | 0 | | 0.000665453 | | 0.002314184 | 0.033471832 |
| TCGA_TCGA-FD-A3B8 | | 0.040936271 | | 0 | | 0 | 0.143842273 |
| TCGA_TCGA-XF-A9SY | | 0.008281012 | | 0 | | 0.025452095 | 0.106495393 |
| TCGA_TCGA-CU-A0YN | | 0.004338901 | | 0 | | 0.013216119 | 0.008856827 |
| TCGA_TCGA-FD-A5BZ | | 0.088342685 | | 0 | | 0.02562978 | 0 |
| TCGA_TCGA-XF-A8HD | | 0.010917143 | | 0 | | 0.0539944 | 0.216563987 |
| TCGA_TCGA-GV-A3JX | | 0 | | 0 | | 0.008141657 | 0.14739091 |
| TCGA_TCGA-FD-A3B6 | | 0.004702283 | | 0 | | 0.00091898 | 0.203205808 |
| TCGA_TCGA-GU-A764 | | 0.001628587 | | 0 | | 0.10822026 | 0.063172491 |
| TCGA_TCGA-5N-A9KM | | 0 | | 0 | | 0.069729388 | 0.161408901 |
| TCGA_TCGA-FD-A6TD | | 0.021450008 | | 0 | | 0.043735589 | 0.119843472 |
| TCGA_TCGA-ZF-AA56 | | 0.031687384 | | 0.02048455 | | 0.065487994 | 0.01850998 |
| TCGA_TCGA-ZF-AA58 | | 0.010894589 | | 0 | | 0 | 0.109295616 |
| TCGA_TCGA-FJ-A871 | | 0.002094153 | | 0 | | 0.051855048 | 0 |
| TCGA_TCGA-ZF-A9RN | | 0.027638421 | | 0 | | 0 | 0.276329212 |
| TCGA_TCGA-YC-A89H | | 0.049197698 | | 0 | | 0.035588156 | 0.061920551 |
| TCGA_TCGA-XF-AAMQ | | 0.011305713 | | 0.008313978 | | 0 | 0.070940697 |
| TCGA_TCGA-FD-A3B4 | | 0.105588041 | | 0.043010911 | | 0.007593261 | 0.077422978 |
| TCGA_TCGA-DK-A1AB | | 0 | | 0.048027134 | | 0.040616358 | 0 |
| TCGA_TCGA-BT-A20X | | 0 | | 0.012088132 | | 0.265943569 | 0 |
| TCGA_TCGA-ZF-AA5H | | 0.035357459 | | 0 | | 0 | 0.045013469 |
| TCGA_TCGA-XF-A9T2 | | 0.00670303 | | 0 | | 0.01363105 | 0 |
| TCGA_TCGA-DK-A3WX | | 0 | | 0 | | 0 | 0 |
| TCGA_TCGA-DK-A6B2 | | 0.021837494 | | 0 | | 0.069231712 | 0.077177961 |
| TCGA_TCGA-4Z-AA86 | | 0.005038375 | | 0 | | 0 | 0.138267851 |
| TCGA_TCGA-XF-A9SL | | 0.258281864 | | 0 | | 0.016157195 | 0.024879311 |
| TCGA_TCGA-XF-AAN2 | | 0.006918405 | | 0 | | 0.04608048 | 0.144575212 |
| TCGA_TCGA-ZF-A9RD | | 0.062053892 | | 0 | | 0.000536848 | 0.15443163 |
| TCGA_TCGA-DK-A2I2 | | 0 | | 0 | | 0 | 0.017072966 |
| TCGA_TCGA-FD-A62S | | 0.013553871 | | 0 | | 0.00686789 | 0.023761071 |
| TCGA_TCGA-GD-A76B | | 0.021981751 | | 0 | | 0.131072356 | 0.123092474 |
| TCGA_TCGA-GC-A6I1 | | 0.008113692 | | 0 | | 0.017692134 | 0.175451368 |
| TCGA_TCGA-XF-A9SK | | 0 | | 0.010584256 | | 0.042161166 | 0.04777716 |
| TCGA_TCGA-DK-A1AD | | 0.055319371 | | 0 | | 0.110578518 | 0 |
| TCGA_TCGA-K4-A5RI | | 0 | | 0 | | 0.075588975 | 0 |
| TCGA_TCGA-XF-AAN8 | | 0.03428104 | | 0 | | 0 | 0.040021546 |
| TCGA_TCGA-G2-A2EJ | | 0 | | 0.031741393 | | 0 | 0 |
| TCGA_TCGA-FD-A3B7 | | 0.014302506 | | 0 | | 0 | 0.184888899 |
| TCGA_TCGA-FD-A5BS | | 0 | | 0.003900496 | | 0.152363422 | 0.216236419 |
| TCGA_TCGA-LC-A66R | | 0.010778478 | | 0 | | 0.020394212 | 0.094020544 |
| TCGA_TCGA-UY-A9PB | | 0.012514169 | | 0 | | 0.072334544 | 0.056548966 |
| TCGA_TCGA-K4-A54R | | 0 | | 0 | | 0.009748646 | 0.197679106 |
| TCGA_TCGA-FD-A43P | | 0.084509693 | | 0 | | 0.005999111 | 0.240845561 |
| TCGA_TCGA-DK-A3IV | | 0.050113997 | | 0.103656477 | | 0 | 0.054698464 |
| TCGA_TCGA-GU-A762 | | 0.001336328 | | 0 | | 0.024864831 | 0.204487713 |
| TCGA_TCGA-XF-AAME | | 0.02238078 | | 0.065280791 | | 0.038488191 | 0.041755162 |
| TCGA_TCGA-FD-A62P | | 0.016060057 | | 0 | | 0 | 0 |
| TCGA_TCGA-BT-A42F | | 0.038934263 | | 0 | | 0.005449877 | 0.144456837 |
| TCGA_TCGA-ZF-A9RF | | 0.014145818 | | 0 | | 0.009132272 | 0.346275112 |
| TCGA_TCGA-BT-A20U | | 0.009920778 | | 0 | | 0.008469792 | 0.095506886 |
| TCGA_TCGA-XF-A9SW | | 0.06694291 | | 0 | | 0.027079081 | 0.016685342 |
| TCGA_TCGA-FD-A3B3 | | 0.00345659 | | 0 | | 0.04138614 | 0.149444086 |
| TCGA_TCGA-E7-A6MD | | 0.049387147 | | 0.094618337 | | 0 | 0.074034013 |
| TCGA_TCGA-BT-A0YX | | 0 | | 0.055325919 | | 0.003624851 | 0.078418498 |
| TCGA_TCGA-XF-A9T3 | | 0.040247285 | | 0 | | 0.026848003 | 0 |
| TCGA_TCGA-PQ-A6FN | | 0.086932106 | | 0 | | 0.095886101 | 0.088264782 |
| TCGA_TCGA-E7-A3X6 | | 0 | | 0 | | 0.016524468 | 0.178773819 |
| TCGA_TCGA-DK-A3WW | | 0.001277094 | | 0 | | 0 | 0.288684958 |
| TCGA_TCGA-K4-A3WS | | 0.113054322 | | 0 | | 0.026851751 | 0.104040393 |
| TCGA_TCGA-GC-A3YS | | 0.000651417 | | 0 | | 0.017815084 | 0 |
| TCGA_TCGA-DK-A1AF | | 0.011444223 | | 0 | | 0 | 0 |
| TCGA_TCGA-DK-AA74 | | 0 | | 0.076781233 | | 0 | 0.14474941 |
| TCGA_TCGA-G2-A2EO | | 0.004834657 | | 0 | | 0.025456536 | 0.192744864 |
| TCGA_TCGA-UY-A78K | | 0.043765654 | | 0.105240169 | | 0.06462325 | 0.154765996 |
| TCGA_TCGA-FD-A6TH | | 0.127353654 | | 0 | | 0.0225788 | 0 |
| TCGA_TCGA-DK-AA6W | | 0.011722317 | | 0 | | 0 | 0 |
| TCGA_TCGA-DK-A1A6 | | 0 | | 0 | | 0 | 0.239546511 |
| TCGA_TCGA-E7-A541 | | 0 | | 0.006398498 | | 0.012365104 | 0.359535075 |
| TCGA_TCGA-XF-A8HE | | 0 | | 0.10144859 | | 0 | 0.081864615 |
| TCGA_TCGA-DK-AA6Q | | 0.014451109 | | 0 | | 0 | 0.339449432 |
| TCGA_TCGA-GC-A3WC | | 0.034041977 | | 0.009625366 | | 0 | 0.123464362 |
| TCGA_TCGA-K4-A4AC | | 0.001020182 | | 0 | | 0 | 0.051278584 |
| TCGA_TCGA-FD-A3SL | | 0.068984789 | | 0 | | 0.050345465 | 0.032871145 |
| TCGA_TCGA-K4-A5RH | | 0.00357718 | | 0 | | 0.005452039 | 0.39702673 |
| TCGA_TCGA-FD-A5C1 | | 0 | | 0.059123214 | | 0.0290253 | 0.101273083 |
| TCGA_TCGA-2F-A9KO | | 0 | | 0.018574047 | | 0.017582356 | 0.18838064 |
| TCGA_TCGA-DK-A3IM | | 0 | | 0 | | 0 | 0 |
| TCGA_TCGA-XF-A9T6 | | 0 | | 0 | | 0 | 0.157037803 |
| TCGA_TCGA-FD-A43Y | | 0 | | 0.029691519 | | 0 | 0.025706661 |
| TCGA_TCGA-GV-A3QG | | 0.214581213 | | 0 | | 0 | 0.12533589 |
| TCGA_TCGA-DK-AA6M | | 0.021131539 | | 0 | | 0 | 0.034627282 |
| TCGA_TCGA-ZF-AA52 | | 0.028568354 | | 0 | | 0 | 0.060267483 |
| TCGA_TCGA-K4-A5RJ | | 0.008867315 | | 0 | | 0.011304909 | 0.126265923 |
| TCGA_TCGA-BT-A3PK | | 0.027361516 | | 0 | | 0 | 0 |
| TCGA_TCGA-FD-A3N6 | | 0 | | 0 | | 0.234478528 | 0.019944641 |
| TCGA_TCGA-UY-A78P | | 0.010361866 | | 0 | | 0 | 0.008889813 |
| TCGA_TCGA-BT-A20Q | | 0 | | 0 | | 0.11051035 | 0.115076741 |
| TCGA_TCGA-CU-A0YR | | 0.055758625 | | 0 | | 0.04533352 | 0.04425187 |
| TCGA_TCGA-SY-A9G5 | | 0 | | 0 | | 0.010443054 | 0.200720252 |
| TCGA_TCGA-4Z-AA7Q | | 0.019531895 | | 0 | | 0 | 0.27475907 |
| TCGA_TCGA-FD-A5BT | | 0.090724275 | | 0 | | 0 | 0.18736333 |
| TCGA_TCGA-FD-A6TF | | 0.121140182 | | 0 | | 0 | 0.069934273 |
| TCGA_TCGA-XF-A8HF | | 0.026845354 | | 0 | | 0.035959121 | 0 |
| TCGA_TCGA-FD-A43S | | 0.106750406 | | 0.06428443 | | 0.057360314 | 0.241038167 |
| TCGA_TCGA-K4-A83P | | 0.253099916 | | 0.020297008 | | 0.009547875 | 0.120732681 |
| TCGA_TCGA-DK-A2I1 | | 0.087049865 | | 0 | | 0.111552273 | 0.036432495 |
| TCGA_TCGA-KQ-A41P | | 0.007773142 | | 0.009735816 | | 0 | 0.023991438 |
| TCGA_TCGA-UY-A9PH | | 0.00731621 | | 0 | | 0.052353711 | 0.142295896 |
| TCGA_TCGA-FD-A5BY | | 6.57E-05 | | 0 | | 0 | 0 |
| TCGA_TCGA-E7-A519 | | 0 | | 0.075491394 | | 0 | 0.039163095 |
| TCGA_TCGA-DK-A3IU | | 0.111605924 | | 0 | | 0.009899923 | 0.204685407 |
| TCGA_TCGA-FD-A3SP | | 0 | | 0 | | 0.011026914 | 0.079026812 |
| TCGA_TCGA-GC-A6I3 | | 0.001955111 | | 0.013336817 | | 0.109540893 | 0.138629876 |
| TCGA_TCGA-GC-A3RC | | 0 | | 0 | | 0 | 0 |
| TCGA_TCGA-GU-AATO | | 0 | | 0.001004415 | | 0.155964286 | 0.128049103 |
| TCGA_TCGA-DK-A6AV | | 0.140210953 | | 0 | | 0 | 0.117703885 |
| TCGA_TCGA-BT-A20R | | 0.01966594 | | 0 | | 0 | 0.038135998 |
| GSE13507_GSM340606 | | 0 | | 0.05306699 | | 0 | 0 |
| GSE13507_GSM340607 | | 0 | | 0 | | 0.078671108 | 0.339829259 |
| GSE13507_GSM340610 | | 0 | | 0.05460369 | | 0.02142691 | 0.119999204 |
| GSE13507_GSM340623 | | 0.089083933 | | 0 | | 0 | 0.015897659 |
| GSE13507_GSM340636 | | 0.040454233 | | 0 | | 0.006034457 | 0.445307839 |
| GSE13507_GSM340637 | | 0.046276387 | | 0 | | 0 | 0.180389259 |
| GSE13507_GSM340645 | | 0 | | 0.005249152 | | 0.010488036 | 0.010231229 |
| GSE13507_GSM340647 | | 0.051983867 | | 0 | | 0.144161803 | 0.003251688 |
| GSE13507_GSM340650 | | 0.018038218 | | 0 | | 0 | 0.015638944 |
| GSE13507_GSM340654 | | 0.027666533 | | 0 | | 0 | 0.100853559 |
| GSE13507_GSM340655 | | 0.026395485 | | 0 | | 0.020080925 | 0.057554543 |
| GSE13507_GSM340660 | | 0.026568408 | | 0 | | 0 | 0.048646709 |
| GSE13507_GSM340673 | | 0.118759509 | | 0 | | 0 | 0.050110358 |
| GSE13507_GSM340674 | | 0.038534115 | | 0 | | 0 | 0.047975438 |
| GSE13507_GSM340675 | | 0.015585657 | | 0.030087857 | | 0 | 0.159142342 |
| GSE13507_GSM340676 | | 0.0111609 | | 0.002370823 | | 0 | 0.044851428 |
| GSE13507_GSM340677 | | 0.030261259 | | 0 | | 0.101487708 | 0.0923975 |
| GSE13507_GSM340679 | | 0.075365549 | | 0 | | 0.109513677 | 0.124488206 |
| GSE13507_GSM340681 | | 0.044410721 | | 0 | | 0.087890675 | 0.009060389 |
| GSE13507_GSM340686 | | 0.036517629 | | 0 | | 0 | 0.025334557 |
| GSE13507_GSM340687 | | 0.024481097 | | 0 | | 0.026810041 | 0.085104557 |
| GSE13507_GSM340693 | | 0 | | 0 | | 0.183710369 | 0.134335246 |
| GSE13507_GSM340694 | | 0.064523617 | | 0 | | 0.032723294 | 0.191205043 |
| GSE13507_GSM340696 | | 0.020634952 | | 0 | | 0 | 0.090746218 |
| GSE13507_GSM340697 | | 0.196413845 | | 0.124861598 | | 0 | 0.026711668 |
| GSE13507_GSM340700 | | 0 | | 0.052651193 | | 0.034475286 | 0.063022668 |
| GSE13507_GSM340702 | | 0 | | 0 | | 0.008357561 | 0 |
| GSE13507_GSM340705 | | 0.137241962 | | 0.047900464 | | 0 | 0.028422937 |
| GSE13507_GSM340708 | | 0.120217075 | | 0 | | 0.061790416 | 0.113555658 |
| GSE13507_GSM340722 | | 0.005552896 | | 0.000742185 | | 0 | 0 |
| GSE13507_GSM340723 | | 0.037656464 | | 0 | | 0.08677388 | 0.164442679 |
| GSE13507_GSM340725 | | 0.029304426 | | 0 | | 0.05946432 | 0.076183174 |
| GSE13507_GSM340727 | | 0 | | 0.064196261 | | 0.001535647 | 0.076572712 |
| GSE13507_GSM340728 | | 0 | | 0.020097115 | | 0.000656859 | 0.008868728 |
| GSE13507_GSM340730 | | 0.045488502 | | 0 | | 0.007711793 | 0.151402788 |
| GSE13507_GSM340732 | | 0 | | 0 | | 0.024246708 | 0.217952178 |
| GSE13507_GSM340741 | | 0.005632057 | | 0 | | 0.034811714 | 0.330298902 |
| GSE13507_GSM340742 | | 0 | | 0.005545394 | | 0 | 0.054788612 |
| GSE13507_GSM340744 | | 0.017604675 | | 0.054347304 | | 0.034408309 | 0.364436387 |
| GSE13507_GSM340745 | | 0.004007107 | | 0.000658997 | | 0.046292944 | 0.340624062 |
| GSE13507_GSM340746 | | 0.04602014 | | 0 | | 0.001447381 | 0.027225277 |
| GSE13507_GSM340751 | | 0.035309924 | | 0 | | 0.019615738 | 0.078933149 |
| GSE13507_GSM340752 | | 0.007874601 | | 0 | | 0.029580431 | 0.103634314 |
| GSE13507_GSM340758 | | 0.025062056 | | 0 | | 0 | 0.125951842 |
| GSE13507_GSM340763 | | 0.01449477 | | 0 | | 0.051655249 | 0.168909027 |
| GSE13507_GSM340769 | | 0.095710662 | | 0 | | 0.080300685 | 0.050737319 |
| id | | T cells CD4 naive | | T cells CD4 memory resting | | T cells CD4 memory activated | T cells follicular helper |
| TCGA_TCGA-ZF-A9R7 | | 0 | | 0 | | 0.10754957 | 0.183503189 |
| TCGA_TCGA-E7-A97P | | 0 | | 0 | | 0.223968088 | 0.050196142 |
| TCGA_TCGA-4Z-AA81 | | 0 | | 0 | | 0.228281656 | 0.103118726 |
| TCGA_TCGA-XF-A9SM | | 0 | | 0 | | 0.19033815 | 0.015532288 |
| TCGA_TCGA-DK-A3IN | | 0 | | 0.108517209 | | 0.064989622 | 0.029084175 |
| TCGA_TCGA-DK-A2I4 | | 0 | | 0.088894289 | | 0.114875137 | 0.020080333 |
| TCGA_TCGA-XF-AAMT | | 0 | | 0.058357542 | | 0.002774755 | 0 |
| TCGA_TCGA-XF-A9SU | | 0 | | 0 | | 0 | 0.008721626 |
| TCGA_TCGA-FD-A6TK | | 0 | | 0.161574059 | | 0.128481173 | 0.094054019 |
| TCGA_TCGA-XF-A9T5 | | 0 | | 0 | | 0.076327677 | 0.036621137 |
| TCGA_TCGA-XF-A9SX | | 0 | | 0.117506433 | | 0.034807998 | 0.016972294 |
| TCGA_TCGA-E7-A7DV | | 0 | | 0.153678807 | | 0.023359919 | 0 |
| TCGA_TCGA-DK-AA6L | | 0 | | 0.120617067 | | 0 | 0.042710291 |
| TCGA_TCGA-XF-A9T4 | | 0 | | 0.169461534 | | 0.084182615 | 0.120302468 |
| TCGA_TCGA-UY-A8OB | | 0 | | 0 | | 0.255649983 | 0.021344427 |
| TCGA_TCGA-DK-A1A5 | | 0 | | 0.033401631 | | 0 | 0.092269704 |
| TCGA_TCGA-SY-A9G0 | | 0 | | 0.119743573 | | 0 | 0 |
| TCGA_TCGA-FD-A5BX | | 0 | | 0.019442362 | | 0.003342593 | 0.001845626 |
| TCGA_TCGA-ZF-AA54 | | 0 | | 0.081177672 | | 0 | 0.031376777 |
| TCGA_TCGA-GU-A766 | | 0 | | 0.010370496 | | 0.234620887 | 0.045763305 |
| TCGA_TCGA-C4-A0F1 | | 0 | | 0.094265141 | | 0 | 0.057953967 |
| TCGA_TCGA-G2-A2ES | | 0 | | 0 | | 0.176679581 | 0.057719813 |
| TCGA_TCGA-XF-AAN4 | | 0 | | 0.039314054 | | 0.048876711 | 0 |
| TCGA_TCGA-4Z-AA7N | | 0 | | 0.136890859 | | 0.033332261 | 0.040991979 |
| TCGA_TCGA-UY-A8OC | | 0 | | 0.039176416 | | 0 | 0.065894677 |
| TCGA_TCGA-GV-A3JV | | 0 | | 0 | | 0.013040839 | 0.126343845 |
| TCGA_TCGA-XF-A9T8 | | 0 | | 0 | | 0.121002371 | 0.046408027 |
| TCGA_TCGA-E7-A7XN | | 0 | | 0 | | 0.206748274 | 0.024398436 |
| TCGA_TCGA-4Z-AA7W | | 0 | | 0 | | 0.183215016 | 0.096247207 |
| TCGA_TCGA-FD-A43U | | 0 | | 0 | | 0.008936824 | 0.028200275 |
| TCGA_TCGA-ZF-AA4V | | 0 | | 0 | | 0.056875224 | 0.111517613 |
| TCGA_TCGA-ZF-AA53 | | 0 | | 0 | | 0.035175743 | 0.062869737 |
| TCGA_TCGA-BL-A13I | | 0 | | 0.130456449 | | 0.014040339 | 0.008286028 |
| TCGA_TCGA-CU-A72E | | 0 | | 0.085308937 | | 0 | 0.038105566 |
| TCGA_TCGA-GC-A3I6 | | 0 | | 0.06074154 | | 0.094558288 | 0.055795338 |
| TCGA_TCGA-FD-A6TA | | 0 | | 0.095976824 | | 0.001715525 | 0.020614054 |
| TCGA_TCGA-BL-A3JM | | 0 | | 0 | | 0.021113578 | 0.193764026 |
| TCGA_TCGA-BL-A5ZZ | | 0 | | 0.062481656 | | 0.039877828 | 0.009492756 |
| TCGA_TCGA-FD-A62N | | 0 | | 0.11563283 | | 0.112935758 | 0.003102418 |
| TCGA_TCGA-BT-A20J | | 0 | | 0.20277633 | | 0.02760516 | 0.045391669 |
| TCGA_TCGA-FT-A61P | | 0 | | 0 | | 0.061022691 | 0.168381088 |
| TCGA_TCGA-BT-A20O | | 0 | | 0 | | 0.137708714 | 0.009514219 |
| TCGA_TCGA-4Z-AA82 | | 0 | | 0.092709603 | | 0 | 0.052626215 |
| TCGA_TCGA-FD-A5BU | | 0 | | 0.05535296 | | 0.013812725 | 0.031250915 |
| TCGA_TCGA-C4-A0F0 | | 0 | | 0.058140277 | | 0.065087335 | 0 |
| TCGA_TCGA-FD-A3N5 | | 0 | | 0 | | 0.062638332 | 0.097178545 |
| TCGA_TCGA-DK-AA6S | | 0 | | 0.193174257 | | 0.074294564 | 0.032729921 |
| TCGA_TCGA-BT-A42E | | 0 | | 0.001167552 | | 0.028840558 | 0.094783954 |
| TCGA_TCGA-ZF-AA4R | | 0 | | 0 | | 0 | 0.100978468 |
| TCGA_TCGA-DK-A3WY | | 0 | | 0.010241048 | | 0.0096788 | 0.052911167 |
| TCGA_TCGA-G2-A2EF | | 0 | | 0.031582785 | | 0.197277997 | 0.023476039 |
| TCGA_TCGA-GU-AATQ | | 0 | | 0.086478643 | | 0 | 0.099709025 |
| TCGA_TCGA-YC-A8S6 | | 0.021333407 | | 0.077027821 | | 0.041607793 | 0 |
| TCGA_TCGA-XF-A9SJ | | 0 | | 0.028278672 | | 0.111606589 | 0.009933828 |
| TCGA_TCGA-DK-AA6T | | 0 | | 0 | | 0.02257655 | 0.020713385 |
| TCGA_TCGA-XF-AAMW | | 0 | | 0.177283937 | | 0.026892897 | 0.021120764 |
| TCGA_TCGA-BT-A3PJ | | 0 | | 0.060200163 | | 0.094994475 | 0.071550249 |
| TCGA_TCGA-GC-A3OO | | 0.008638386 | | 0 | | 0.002255681 | 0.024137423 |
| TCGA_TCGA-XF-AAN5 | | 0 | | 0.06672343 | | 0.221428862 | 0.035802204 |
| TCGA_TCGA-FD-A6TB | | 0 | | 0.02710049 | | 0.045780727 | 0.034070116 |
| TCGA_TCGA-XF-AAN3 | | 0 | | 0.147723439 | | 0.00519798 | 0.006188919 |
| TCGA_TCGA-FD-A3B8 | | 0 | | 0.094565532 | | 0.076270938 | 0 |
| TCGA_TCGA-XF-A9SY | | 0 | | 0.047205967 | | 0.133160511 | 0 |
| TCGA_TCGA-CU-A0YN | | 0 | | 0.24797815 | | 0.005541619 | 0 |
| TCGA_TCGA-FD-A5BZ | | 0 | | 0.003925964 | | 0 | 0.02718944 |
| TCGA_TCGA-XF-A8HD | | 0 | | 0 | | 0.198993774 | 0.0371228 |
| TCGA_TCGA-GV-A3JX | | 0 | | 0 | | 0.061471684 | 0.151246123 |
| TCGA_TCGA-FD-A3B6 | | 0 | | 0.078505014 | | 0.141886175 | 0.024099175 |
| TCGA_TCGA-GU-A764 | | 0 | | 0.104750299 | | 0.098810624 | 0.037590626 |
| TCGA_TCGA-5N-A9KM | | 0 | | 0.100558546 | | 0.055795524 | 0.038552444 |
| TCGA_TCGA-FD-A6TD | | 0 | | 0.0437774 | | 0.09490716 | 0.019464343 |
| TCGA_TCGA-ZF-AA56 | | 0 | | 0.138785291 | | 0.05245905 | 0 |
| TCGA_TCGA-ZF-AA58 | | 0 | | 0 | | 0.113996042 | 0.023861996 |
| TCGA_TCGA-FJ-A871 | | 0.183978131 | | 0.232907465 | | 0.239309433 | 0 |
| TCGA_TCGA-ZF-A9RN | | 0 | | 0.135557954 | | 0.091974551 | 0.081594781 |
| TCGA_TCGA-YC-A89H | | 0 | | 0 | | 0.07104576 | 0.011136041 |
| TCGA_TCGA-XF-AAMQ | | 0 | | 0.114299115 | | 0.060432341 | 0.160583611 |
| TCGA_TCGA-FD-A3B4 | | 0 | | 0.057609618 | | 0.028923884 | 0.044302047 |
| TCGA_TCGA-DK-A1AB | | 0 | | 0.142329726 | | 0.001086602 | 0.064795804 |
| TCGA_TCGA-BT-A20X | | 0 | | 0 | | 0.119279344 | 0.043746485 |
| TCGA_TCGA-ZF-AA5H | | 0 | | 0.114452465 | | 0.087133775 | 0.013403883 |
| TCGA_TCGA-XF-A9T2 | | 0 | | 0.042689878 | | 0 | 0.03024642 |
| TCGA_TCGA-DK-A3WX | | 0 | | 0.163974162 | | 0.046913313 | 0 |
| TCGA_TCGA-DK-A6B2 | | 0 | | 0.085068003 | | 0 | 0.021430753 |
| TCGA_TCGA-4Z-AA86 | | 0 | | 0.076316015 | | 0.089425716 | 0.052295359 |
| TCGA_TCGA-XF-A9SL | | 0.022119547 | | 0.059115212 | | 0.032333067 | 0 |
| TCGA_TCGA-XF-AAN2 | | 0 | | 0.120851554 | | 0.095762745 | 0.092994315 |
| TCGA_TCGA-ZF-A9RD | | 0 | | 0.025971004 | | 0.121376767 | 0.034448574 |
| TCGA_TCGA-DK-A2I2 | | 0 | | 0.131590781 | | 0 | 0 |
| TCGA_TCGA-FD-A62S | | 0 | | 0.152758939 | | 0.012156922 | 0.001476522 |
| TCGA_TCGA-GD-A76B | | 0 | | 0.067158644 | | 0.090934353 | 0.034111261 |
| TCGA_TCGA-GC-A6I1 | | 0 | | 0 | | 0.073256956 | 0.014922047 |
| TCGA_TCGA-XF-A9SK | | 0 | | 0.150116122 | | 0 | 0.005521819 |
| TCGA_TCGA-DK-A1AD | | 0.124437158 | | 0 | | 0.062459405 | 0 |
| TCGA_TCGA-K4-A5RI | | 0 | | 0.083282682 | | 0 | 0.005248276 |
| TCGA_TCGA-XF-AAN8 | | 0 | | 0.109618922 | | 0.046734522 | 0 |
| TCGA_TCGA-G2-A2EJ | | 0 | | 0 | | 0.083150209 | 0.054110491 |
| TCGA_TCGA-FD-A3B7 | | 0 | | 0.083016856 | | 0.150014211 | 0 |
| TCGA_TCGA-FD-A5BS | | 0 | | 0 | | 0.054883077 | 0.064745895 |
| TCGA_TCGA-LC-A66R | | 0 | | 0.08490224 | | 0.151910981 | 0 |
| TCGA_TCGA-UY-A9PB | | 0 | | 0.022610197 | | 0.109930979 | 0.028469958 |
| TCGA_TCGA-K4-A54R | | 0 | | 0.003327224 | | 0.166857143 | 0.01233068 |
| TCGA_TCGA-FD-A43P | | 0 | | 0 | | 0.098936687 | 0.02201834 |
| TCGA_TCGA-DK-A3IV | | 0 | | 0.067164695 | | 0 | 0.062888007 |
| TCGA_TCGA-GU-A762 | | 0 | | 0.009507189 | | 0.188817312 | 0.030823962 |
| TCGA_TCGA-XF-AAME | | 0 | | 0.09550414 | | 0.030741848 | 0 |
| TCGA_TCGA-FD-A62P | | 0 | | 0 | | 0.138734197 | 0.060049199 |
| TCGA_TCGA-BT-A42F | | 0 | | 0 | | 0.083388067 | 0.055887892 |
| TCGA_TCGA-ZF-A9RF | | 0 | | 0.07444243 | | 0.075029963 | 0 |
| TCGA_TCGA-BT-A20U | | 0 | | 0.030585083 | | 0.018692671 | 0.030308096 |
| TCGA_TCGA-XF-A9SW | | 0 | | 0.086314592 | | 0 | 0.006157623 |
| TCGA_TCGA-FD-A3B3 | | 0 | | 0.085429086 | | 0.093308185 | 0.010362712 |
| TCGA_TCGA-E7-A6MD | | 0 | | 0.242259202 | | 0 | 0.016060153 |
| TCGA_TCGA-BT-A0YX | | 0 | | 0.04060305 | | 0.11847285 | 0.123536184 |
| TCGA_TCGA-XF-A9T3 | | 0 | | 0.011919932 | | 0.036484112 | 0.055233374 |
| TCGA_TCGA-PQ-A6FN | | 0 | | 0 | | 0 | 0.053363818 |
| TCGA_TCGA-E7-A3X6 | | 0 | | 0 | | 0.122742237 | 0.075519886 |
| TCGA_TCGA-DK-A3WW | | 0 | | 0 | | 0.201767091 | 0.041263075 |
| TCGA_TCGA-K4-A3WS | | 0 | | 0.026049374 | | 0.023387952 | 0 |
| TCGA_TCGA-GC-A3YS | | 0 | | 0.198147086 | | 0.03458039 | 0 |
| TCGA_TCGA-DK-A1AF | | 0 | | 0.138736556 | | 0.018741617 | 0 |
| TCGA_TCGA-DK-AA74 | | 0 | | 0.026947434 | | 0.030858019 | 0.044658018 |
| TCGA_TCGA-G2-A2EO | | 0 | | 0.008532674 | | 0.126600304 | 0.080477674 |
| TCGA_TCGA-UY-A78K | | 0 | | 0 | | 0.104995003 | 0 |
| TCGA_TCGA-FD-A6TH | | 0 | | 0.128826809 | | 0 | 0.081185261 |
| TCGA_TCGA-DK-AA6W | | 0 | | 0.141913371 | | 0 | 0.060582221 |
| TCGA_TCGA-DK-A1A6 | | 0 | | 0 | | 0.090122759 | 0.0769846 |
| TCGA_TCGA-E7-A541 | | 0 | | 0 | | 0.086420116 | 0.041318894 |
| TCGA_TCGA-XF-A8HE | | 0 | | 0 | | 0.146527152 | 0.106081121 |
| TCGA_TCGA-DK-AA6Q | | 0 | | 0 | | 0.111758172 | 0.060587438 |
| TCGA_TCGA-GC-A3WC | | 0 | | 0.075768122 | | 0.057312234 | 0.044860764 |
| TCGA_TCGA-K4-A4AC | | 0 | | 0.188583714 | | 0.072671903 | 0 |
| TCGA_TCGA-FD-A3SL | | 0 | | 0.049875727 | | 0 | 0.025568214 |
| TCGA_TCGA-K4-A5RH | | 0 | | 0.016168672 | | 0.135833962 | 0.015894094 |
| TCGA_TCGA-FD-A5C1 | | 0 | | 0.035999639 | | 0.100136718 | 0.071106545 |
| TCGA_TCGA-2F-A9KO | | 0 | | 0.061220845 | | 0.097237288 | 0.067793105 |
| TCGA_TCGA-DK-A3IM | | 0 | | 0.096053831 | | 0 | 0.12870222 |
| TCGA_TCGA-XF-A9T6 | | 0 | | 0 | | 0.04688858 | 0 |
| TCGA_TCGA-FD-A43Y | | 0 | | 0.024417674 | | 0 | 0.060170089 |
| TCGA_TCGA-GV-A3QG | | 0.026340553 | | 0.008198314 | | 0.052881439 | 0 |
| TCGA_TCGA-DK-AA6M | | 0 | | 0.155153415 | | 0 | 0 |
| TCGA_TCGA-ZF-AA52 | | 0 | | 0.079553811 | | 0 | 0.012065295 |
| TCGA_TCGA-K4-A5RJ | | 0 | | 0.09116177 | | 0.021916643 | 0 |
| TCGA_TCGA-BT-A3PK | | 0 | | 0.055316323 | | 0.008425524 | 0.055879947 |
| TCGA_TCGA-FD-A3N6 | | 0 | | 0.013775527 | | 0.005792563 | 0.067721322 |
| TCGA_TCGA-UY-A78P | | 0 | | 0.015883491 | | 0.087343504 | 0.043832129 |
| TCGA_TCGA-BT-A20Q | | 0 | | 0.054004558 | | 0.077718821 | 0.041172281 |
| TCGA_TCGA-CU-A0YR | | 0 | | 0 | | 0.066269405 | 0.040602428 |
| TCGA_TCGA-SY-A9G5 | | 0 | | 0 | | 0.062248166 | 0.098569247 |
| TCGA_TCGA-4Z-AA7Q | | 0 | | 0 | | 0.029060976 | 0.103358453 |
| TCGA_TCGA-FD-A5BT | | 0 | | 0.002874956 | | 0.064145729 | 0.030553989 |
| TCGA_TCGA-FD-A6TF | | 0 | | 0.080075033 | | 0 | 0.003109617 |
| TCGA_TCGA-XF-A8HF | | 0.022391667 | | 0 | | 0 | 0.026806671 |
| TCGA_TCGA-FD-A43S | | 0 | | 0 | | 0.001490726 | 0.021282845 |
| TCGA_TCGA-K4-A83P | | 0 | | 0.110718622 | | 0.047848759 | 0 |
| TCGA_TCGA-DK-A2I1 | | 0 | | 0.006987975 | | 0 | 0.002315557 |
| TCGA_TCGA-KQ-A41P | | 0 | | 0.010704509 | | 0 | 0.028901046 |
| TCGA_TCGA-UY-A9PH | | 0 | | 0.091897599 | | 0.030796639 | 0.033200952 |
| TCGA_TCGA-FD-A5BY | | 0 | | 0.055443636 | | 0 | 0.036497779 |
| TCGA_TCGA-E7-A519 | | 0 | | 0.162574409 | | 0.017003804 | 0.019077027 |
| TCGA_TCGA-DK-A3IU | | 0 | | 0.06390244 | | 0.057148747 | 0.01549425 |
| TCGA_TCGA-FD-A3SP | | 0 | | 0.140255842 | | 0.006663047 | 0 |
| TCGA_TCGA-GC-A6I3 | | 0 | | 0 | | 0.056936274 | 0.055697559 |
| TCGA_TCGA-GC-A3RC | | 0 | | 0.120605193 | | 0.005718896 | 0 |
| TCGA_TCGA-GU-AATO | | 0 | | 0.04579424 | | 0.019072246 | 0.094797338 |
| TCGA_TCGA-DK-A6AV | | 0 | | 0.078480767 | | 0 | 0.181399989 |
| TCGA_TCGA-BT-A20R | | 0 | | 0 | | 0 | 0.009317734 |
| GSE13507_GSM340606 | | 0.002666091 | | 0 | | 0.420981128 | 0 |
| GSE13507_GSM340607 | | 0 | | 0.126931738 | | 0 | 0.020951272 |
| GSE13507_GSM340610 | | 0 | | 0.153027404 | | 0.00261602 | 0.064501224 |
| GSE13507_GSM340623 | | 0 | | 0.011379963 | | 0 | 0.025382898 |
| GSE13507_GSM340636 | | 0 | | 0.023393703 | | 0.011795307 | 0.066542852 |
| GSE13507_GSM340637 | | 0 | | 0.145202341 | | 0 | 0.059256887 |
| GSE13507_GSM340645 | | 0 | | 0.221857922 | | 0 | 0.114669251 |
| GSE13507_GSM340647 | | 0 | | 0.137722343 | | 0 | 0.091067801 |
| GSE13507_GSM340650 | | 0 | | 0.364098197 | | 0 | 0 |
| GSE13507_GSM340654 | | 0 | | 0.123039172 | | 0 | 0 |
| GSE13507_GSM340655 | | 0 | | 0 | | 0 | 0.085253104 |
| GSE13507_GSM340660 | | 0 | | 0.058146891 | | 0.086748619 | 0 |
| GSE13507_GSM340673 | | 0 | | 0.105949951 | | 0.010177543 | 0.094999518 |
| GSE13507_GSM340674 | | 0 | | 0.029480863 | | 0.004699222 | 0.017627888 |
| GSE13507_GSM340675 | | 0 | | 0.032638308 | | 0.113283535 | 0.035661766 |
| GSE13507_GSM340676 | | 0 | | 0.031113394 | | 0.084503086 | 0.042289155 |
| GSE13507_GSM340677 | | 0 | | 0.049821358 | | 0 | 0.07364388 |
| GSE13507_GSM340679 | | 0 | | 0.102679453 | | 0 | 0.071069944 |
| GSE13507_GSM340681 | | 0 | | 0.123857056 | | 0 | 0.099336043 |
| GSE13507_GSM340686 | | 0 | | 0.243137595 | | 0.012417361 | 0.003155221 |
| GSE13507_GSM340687 | | 0 | | 0.136158364 | | 0.016604858 | 0.031634518 |
| GSE13507_GSM340693 | | 0 | | 0.012871729 | | 0.080024239 | 0.033228811 |
| GSE13507_GSM340694 | | 0 | | 0.112798702 | | 0 | 0.047176791 |
| GSE13507_GSM340696 | | 0 | | 0.092516497 | | 0.026506576 | 0.042284854 |
| GSE13507_GSM340697 | | 0 | | 0.08234094 | | 0.038141516 | 0.102179532 |
| GSE13507_GSM340700 | | 0 | | 0.009991307 | | 0 | 0.037390771 |
| GSE13507_GSM340702 | | 0 | | 0.275523104 | | 0 | 0.140750627 |
| GSE13507_GSM340705 | | 0.026832248 | | 0.093163502 | | 0.044309991 | 0 |
| GSE13507_GSM340708 | | 0 | | 0 | | 0.003144876 | 0.070525836 |
| GSE13507_GSM340722 | | 0 | | 0.061719497 | | 0.005935413 | 0.053797851 |
| GSE13507_GSM340723 | | 0 | | 0.070219601 | | 0.058283691 | 0.142121576 |
| GSE13507_GSM340725 | | 0 | | 0.063811839 | | 0 | 0.014294995 |
| GSE13507_GSM340727 | | 0 | | 0.109062187 | | 0 | 0.067670841 |
| GSE13507_GSM340728 | | 0 | | 0.145681023 | | 0 | 0.024689961 |
| GSE13507_GSM340730 | | 0 | | 0.087955243 | | 0 | 0.035963723 |
| GSE13507_GSM340732 | | 0 | | 0.103903109 | | 0 | 0.077944702 |
| GSE13507_GSM340741 | | 0 | | 0.10469393 | | 0.093915163 | 0.084982388 |
| GSE13507_GSM340742 | | 0 | | 0.0190882 | | 0.232325096 | 0 |
| GSE13507_GSM340744 | | 0 | | 0.003745285 | | 0 | 0.030259446 |
| GSE13507_GSM340745 | | 0 | | 0.023609114 | | 0.127615959 | 0.072344357 |
| GSE13507_GSM340746 | | 0 | | 0.072616145 | | 0.037953198 | 0.028100099 |
| GSE13507_GSM340751 | | 0 | | 0.09398882 | | 0.025751363 | 0.112436571 |
| GSE13507_GSM340752 | | 0 | | 0 | | 0.05017272 | 0.065597826 |
| GSE13507_GSM340758 | | 0 | | 0.033463627 | | 0 | 0.09554307 |
| GSE13507_GSM340763 | | 0 | | 0.210546261 | | 0.024290632 | 0 |
| GSE13507_GSM340769 | | 0 | | 0.136686278 | | 0 | 0 |
| id | | T cells regulatory (Tregs) | | T cells gamma delta | | NK cells resting | NK cells activated |
| TCGA_TCGA-ZF-A9R7 | | 0.049412166 | | 0 | | 0 | 0.048294653 |
| TCGA_TCGA-E7-A97P | | 0.003002222 | | 0 | | 0.078804347 | 0 |
| TCGA_TCGA-4Z-AA81 | | 0 | | 0 | | 0.01300816 | 0.067399288 |
| TCGA_TCGA-XF-A9SM | | 0.040585641 | | 0 | | 0.01613715 | 0.005207849 |
| TCGA_TCGA-DK-A3IN | | 0.051234313 | | 0 | | 0.002709595 | 0.02549161 |
| TCGA_TCGA-DK-A2I4 | | 0 | | 0 | | 0 | 0.057643281 |
| TCGA_TCGA-XF-AAMT | | 0.058661175 | | 0.022332079 | | 0 | 0.016879552 |
| TCGA_TCGA-XF-A9SU | | 0.123706759 | | 0.032018426 | | 0 | 0.013394883 |
| TCGA_TCGA-FD-A6TK | | 0 | | 0 | | 0 | 0.063632315 |
| TCGA_TCGA-XF-A9T5 | | 0.085016286 | | 0.010085411 | | 0 | 0.011599825 |
| TCGA_TCGA-XF-A9SX | | 0.156449655 | | 0 | | 0 | 0.041805448 |
| TCGA_TCGA-E7-A7DV | | 0 | | 0 | | 0.014120195 | 0.003037333 |
| TCGA_TCGA-DK-AA6L | | 0.079361969 | | 0 | | 0 | 0.056144495 |
| TCGA_TCGA-XF-A9T4 | | 0 | | 0.030223092 | | 0 | 0.003023156 |
| TCGA_TCGA-UY-A8OB | | 0 | | 0 | | 0.026917546 | 0.108066429 |
| TCGA_TCGA-DK-A1A5 | | 0 | | 0.040935493 | | 0 | 0.093233098 |
| TCGA_TCGA-SY-A9G0 | | 0.057685535 | | 0.016223394 | | 0 | 0.015878594 |
| TCGA_TCGA-FD-A5BX | | 0 | | 0.155657107 | | 0 | 0.019407846 |
| TCGA_TCGA-ZF-AA54 | | 0.053272102 | | 0.024261077 | | 0 | 0.012411242 |
| TCGA_TCGA-GU-A766 | | 0 | | 0 | | 0.08372615 | 0.027039739 |
| TCGA_TCGA-C4-A0F1 | | 0 | | 0.074112336 | | 0 | 0 |
| TCGA_TCGA-G2-A2ES | | 0.01033166 | | 0.043752122 | | 0.018480497 | 0 |
| TCGA_TCGA-XF-AAN4 | | 0.024977023 | | 0.004036015 | | 0 | 0.027361074 |
| TCGA_TCGA-4Z-AA7N | | 0.140933529 | | 0 | | 0 | 0 |
| TCGA_TCGA-UY-A8OC | | 0.017246953 | | 0 | | 0 | 0.015865986 |
| TCGA_TCGA-GV-A3JV | | 0.087275207 | | 0 | | 0 | 0.012749014 |
| TCGA_TCGA-XF-A9T8 | | 0 | | 0 | | 0.017933455 | 0.069202975 |
| TCGA_TCGA-E7-A7XN | | 0.043579065 | | 0 | | 0.099472318 | 0 |
| TCGA_TCGA-4Z-AA7W | | 0.050076203 | | 0.003512245 | | 0.092710222 | 0 |
| TCGA_TCGA-FD-A43U | | 0.101110466 | | 0 | | 0.022890134 | 0 |
| TCGA_TCGA-ZF-AA4V | | 0.03484905 | | 0.014078083 | | 0 | 0.058195528 |
| TCGA_TCGA-ZF-AA53 | | 0.010250692 | | 0.050412043 | | 0 | 0.05720585 |
| TCGA_TCGA-BL-A13I | | 0.036109142 | | 0 | | 0.006161738 | 0 |
| TCGA_TCGA-CU-A72E | | 0.045322789 | | 0 | | 0 | 0.050236017 |
| TCGA_TCGA-GC-A3I6 | | 0.003799564 | | 0 | | 0.027089836 | 0.058080102 |
| TCGA_TCGA-FD-A6TA | | 0.145635281 | | 0 | | 0 | 0.007438356 |
| TCGA_TCGA-BL-A3JM | | 0 | | 0.100051681 | | 0 | 0.063557625 |
| TCGA_TCGA-BL-A5ZZ | | 0.024642005 | | 0.010030909 | | 0.023841961 | 0.032355443 |
| TCGA_TCGA-FD-A62N | | 0.032447754 | | 0.003059368 | | 0 | 0 |
| TCGA_TCGA-BT-A20J | | 0.046222553 | | 0 | | 0 | 0.069426409 |
| TCGA_TCGA-FT-A61P | | 0 | | 0.064264648 | | 0 | 0.05327611 |
| TCGA_TCGA-BT-A20O | | 0.057286792 | | 0.02608299 | | 0.06507579 | 0 |
| TCGA_TCGA-4Z-AA82 | | 0.045377333 | | 0 | | 0 | 0.088036513 |
| TCGA_TCGA-FD-A5BU | | 0.063089887 | | 0 | | 0 | 0.060365638 |
| TCGA_TCGA-C4-A0F0 | | 0.004486539 | | 0 | | 0 | 0.01403672 |
| TCGA_TCGA-FD-A3N5 | | 0.011639437 | | 0.012491553 | | 0 | 0.076681354 |
| TCGA_TCGA-DK-AA6S | | 0.081232152 | | 0.014978991 | | 0 | 0.023572745 |
| TCGA_TCGA-BT-A42E | | 0.115424925 | | 0 | | 0.008222591 | 0.036083652 |
| TCGA_TCGA-ZF-AA4R | | 0.070213198 | | 0.013761445 | | 0 | 0.078011847 |
| TCGA_TCGA-DK-A3WY | | 0.091748437 | | 0 | | 0 | 0.03554547 |
| TCGA_TCGA-G2-A2EF | | 0 | | 0 | | 0.076767585 | 0 |
| TCGA_TCGA-GU-AATQ | | 0.054843221 | | 0 | | 0 | 0.059015108 |
| TCGA_TCGA-YC-A8S6 | | 0.13736322 | | 0 | | 0 | 0.012759965 |
| TCGA_TCGA-XF-A9SJ | | 0.029144555 | | 0 | | 0.053682335 | 0.017294465 |
| TCGA_TCGA-DK-AA6T | | 0 | | 0.039223132 | | 0 | 0.005002229 |
| TCGA_TCGA-XF-AAMW | | 0.030512471 | | 0 | | 0 | 0.025366205 |
| TCGA_TCGA-BT-A3PJ | | 0.036318094 | | 0 | | 0.01940841 | 0.02679228 |
| TCGA_TCGA-GC-A3OO | | 0.060863264 | | 0 | | 0.004202796 | 0 |
| TCGA_TCGA-XF-AAN5 | | 0 | | 0.008979231 | | 0 | 0.084420138 |
| TCGA_TCGA-FD-A6TB | | 0.088993305 | | 0.03487154 | | 0 | 0 |
| TCGA_TCGA-XF-AAN3 | | 0.07817103 | | 0 | | 0 | 0.008441856 |
| TCGA_TCGA-FD-A3B8 | | 0.039206876 | | 0 | | 0.011326434 | 0 |
| TCGA_TCGA-XF-A9SY | | 0.024861316 | | 0 | | 0.065303717 | 0 |
| TCGA_TCGA-CU-A0YN | | 0 | | 0.014749963 | | 0.047562963 | 0 |
| TCGA_TCGA-FD-A5BZ | | 0.047807015 | | 0.025590068 | | 0 | 0.013346577 |
| TCGA_TCGA-XF-A8HD | | 0 | | 0 | | 0.001240826 | 0.097086885 |
| TCGA_TCGA-GV-A3JX | | 0.041402787 | | 0.01495536 | | 0.010628906 | 0.037981136 |
| TCGA_TCGA-FD-A3B6 | | 0.022295435 | | 0 | | 0.064809827 | 0 |
| TCGA_TCGA-GU-A764 | | 0.007794097 | | 0 | | 0 | 0.069145259 |
| TCGA_TCGA-5N-A9KM | | 0 | | 0.046342141 | | 0 | 0.053793922 |
| TCGA_TCGA-FD-A6TD | | 0.010115451 | | 0 | | 0 | 0.079509936 |
| TCGA_TCGA-ZF-AA56 | | 0.039808895 | | 0 | | 0 | 0.024243486 |
| TCGA_TCGA-ZF-AA58 | | 0.049058213 | | 0 | | 0.040348141 | 0 |
| TCGA_TCGA-FJ-A871 | | 0 | | 0 | | 0.029984501 | 0 |
| TCGA_TCGA-ZF-A9RN | | 0.066831652 | | 0 | | 0.01056735 | 0.015229481 |
| TCGA_TCGA-YC-A89H | | 0.040819758 | | 0 | | 0.015476013 | 0.029006814 |
| TCGA_TCGA-XF-AAMQ | | 0.032395597 | | 0.050379625 | | 0 | 0.049650146 |
| TCGA_TCGA-FD-A3B4 | | 0.014318963 | | 0.031643703 | | 0.001045303 | 0.027435881 |
| TCGA_TCGA-DK-A1AB | | 0.048788973 | | 0.01280626 | | 0.010574161 | 0 |
| TCGA_TCGA-BT-A20X | | 0 | | 0.086489014 | | 0.015789195 | 0 |
| TCGA_TCGA-ZF-AA5H | | 0 | | 0 | | 0.042205009 | 0.052848796 |
| TCGA_TCGA-XF-A9T2 | | 0.013173191 | | 0.030096282 | | 0 | 0.11614911 |
| TCGA_TCGA-DK-A3WX | | 0 | | 0.00483207 | | 0.040906144 | 0.052184041 |
| TCGA_TCGA-DK-A6B2 | | 0.078104501 | | 0.023834138 | | 0 | 0.025443425 |
| TCGA_TCGA-4Z-AA86 | | 0.022879151 | | 0 | | 0.074096818 | 0 |
| TCGA_TCGA-XF-A9SL | | 0.123447094 | | 0.009229065 | | 0 | 0 |
| TCGA_TCGA-XF-AAN2 | | 0.013393309 | | 0 | | 0.055756839 | 0.037181223 |
| TCGA_TCGA-ZF-A9RD | | 0.015262354 | | 0.025069849 | | 0 | 0.029331236 |
| TCGA_TCGA-DK-A2I2 | | 0.034579736 | | 0 | | 0 | 0.00269005 |
| TCGA_TCGA-FD-A62S | | 0.066941505 | | 0 | | 0 | 0.005608739 |
| TCGA_TCGA-GD-A76B | | 0.150989576 | | 0 | | 0.01716624 | 0.01939285 |
| TCGA_TCGA-GC-A6I1 | | 0.047943886 | | 0.005260654 | | 0 | 0.041873074 |
| TCGA_TCGA-XF-A9SK | | 0.097349652 | | 0 | | 0 | 0.062211835 |
| TCGA_TCGA-DK-A1AD | | 0.032710699 | | 0.138293428 | | 0.05832021 | 0 |
| TCGA_TCGA-K4-A5RI | | 0.057647589 | | 0 | | 0.080357058 | 0.074627761 |
| TCGA_TCGA-XF-AAN8 | | 0.004705653 | | 0.002905493 | | 0 | 0.009128553 |
| TCGA_TCGA-G2-A2EJ | | 0 | | 0.132177081 | | 0 | 0.034302518 |
| TCGA_TCGA-FD-A3B7 | | 0 | | 0.045659034 | | 0.030841809 | 0 |
| TCGA_TCGA-FD-A5BS | | 0.10269082 | | 0 | | 0.020452634 | 0.033263367 |
| TCGA_TCGA-LC-A66R | | 0 | | 0.110359494 | | 0.010718546 | 0.019625991 |
| TCGA_TCGA-UY-A9PB | | 0.03818479 | | 0.027401208 | | 0.004332268 | 0.046672857 |
| TCGA_TCGA-K4-A54R | | 0 | | 0 | | 0.143453048 | 0.009191397 |
| TCGA_TCGA-FD-A43P | | 0.13098246 | | 0 | | 0 | 0.039578085 |
| TCGA_TCGA-DK-A3IV | | 0.202254887 | | 0 | | 0 | 0.061537314 |
| TCGA_TCGA-GU-A762 | | 0.039507168 | | 0 | | 0.113080199 | 0 |
| TCGA_TCGA-XF-AAME | | 0.099321863 | | 0.028464501 | | 0 | 0 |
| TCGA_TCGA-FD-A62P | | 0 | | 0.064666476 | | 0.020874921 | 0 |
| TCGA_TCGA-BT-A42F | | 0.026249385 | | 0 | | 0 | 0.045169392 |
| TCGA_TCGA-ZF-A9RF | | 0.046500491 | | 0 | | 0 | 0.029757744 |
| TCGA_TCGA-BT-A20U | | 0.111666629 | | 0 | | 0.01465171 | 0.010161318 |
| TCGA_TCGA-XF-A9SW | | 0.015541102 | | 0.025688046 | | 0 | 0.029260896 |
| TCGA_TCGA-FD-A3B3 | | 0.012689237 | | 0 | | 0.045426382 | 0.01319717 |
| TCGA_TCGA-E7-A6MD | | 0.149428838 | | 0 | | 0 | 0.030233331 |
| TCGA_TCGA-BT-A0YX | | 0 | | 0.022258251 | | 0 | 0.065110136 |
| TCGA_TCGA-XF-A9T3 | | 0.030493553 | | 0.041332106 | | 0 | 0.070931554 |
| TCGA_TCGA-PQ-A6FN | | 0.070741184 | | 0.034207031 | | 0 | 0.026147646 |
| TCGA_TCGA-E7-A3X6 | | 0.087634478 | | 0 | | 0.025565773 | 0.021064241 |
| TCGA_TCGA-DK-A3WW | | 0.044463761 | | 0.002991647 | | 0.071903733 | 0 |
| TCGA_TCGA-K4-A3WS | | 0.081702026 | | 0 | | 0 | 0.031995226 |
| TCGA_TCGA-GC-A3YS | | 0.029296784 | | 0.012557543 | | 0.048177736 | 0.037032434 |
| TCGA_TCGA-DK-A1AF | | 0.020023772 | | 0.106534401 | | 0 | 0.024883434 |
| TCGA_TCGA-DK-AA74 | | 0.014960449 | | 0.002729223 | | 0 | 0.033767408 |
| TCGA_TCGA-G2-A2EO | | 0.076871712 | | 0 | | 0.064543429 | 0 |
| TCGA_TCGA-UY-A78K | | 0.058924796 | | 0.040766097 | | 0 | 0.024384264 |
| TCGA_TCGA-FD-A6TH | | 0.014262229 | | 0.040457799 | | 0 | 0.037453196 |
| TCGA_TCGA-DK-AA6W | | 0.054916419 | | 0 | | 0 | 0.008149149 |
| TCGA_TCGA-DK-A1A6 | | 0.040426082 | | 0.024476417 | | 0 | 0.009970337 |
| TCGA_TCGA-E7-A541 | | 0.082103167 | | 0 | | 0 | 0.051246335 |
| TCGA_TCGA-XF-A8HE | | 0 | | 0 | | 0.103750163 | 0 |
| TCGA_TCGA-DK-AA6Q | | 0.058207694 | | 0 | | 0.034221131 | 0.002830921 |
| TCGA_TCGA-GC-A3WC | | 0.044031319 | | 0 | | 0 | 0.023141074 |
| TCGA_TCGA-K4-A4AC | | 0 | | 0 | | 0.000947315 | 0.043026917 |
| TCGA_TCGA-FD-A3SL | | 0.037459136 | | 0 | | 0 | 0.024719806 |
| TCGA_TCGA-K4-A5RH | | 0.03713378 | | 0.006762478 | | 0.034633046 | 0 |
| TCGA_TCGA-FD-A5C1 | | 0.012880682 | | 0.010486795 | | 0 | 0.027612264 |
| TCGA_TCGA-2F-A9KO | | 0.087508636 | | 0 | | 0 | 0.045566874 |
| TCGA_TCGA-DK-A3IM | | 0.018118075 | | 0 | | 0 | 0.016091463 |
| TCGA_TCGA-XF-A9T6 | | 0.068933835 | | 0 | | 0 | 0.027641862 |
| TCGA_TCGA-FD-A43Y | | 0.015445134 | | 0.020719659 | | 0 | 0.037717663 |
| TCGA_TCGA-GV-A3QG | | 0.064063915 | | 0.00495442 | | 0 | 0.03284924 |
| TCGA_TCGA-DK-AA6M | | 0.070767875 | | 0 | | 0 | 0.093973657 |
| TCGA_TCGA-ZF-AA52 | | 0.034355393 | | 0 | | 0 | 0.06086178 |
| TCGA_TCGA-K4-A5RJ | | 0.023253752 | | 0.134711611 | | 0 | 0.056290841 |
| TCGA_TCGA-BT-A3PK | | 0.065146961 | | 0.00583453 | | 0.000926079 | 0.014500294 |
| TCGA_TCGA-FD-A3N6 | | 0.021075643 | | 0.016158657 | | 0.010045492 | 0.003703717 |
| TCGA_TCGA-UY-A78P | | 0 | | 0.026179459 | | 0 | 0.035040453 |
| TCGA_TCGA-BT-A20Q | | 0.084241772 | | 0 | | 0.006840993 | 0.007553478 |
| TCGA_TCGA-CU-A0YR | | 0.034731673 | | 0.006448019 | | 0.012801184 | 0.012726649 |
| TCGA_TCGA-SY-A9G5 | | 0.036726229 | | 0 | | 0.039544445 | 0.025932571 |
| TCGA_TCGA-4Z-AA7Q | | 0.025007921 | | 0 | | 0 | 0.083896661 |
| TCGA_TCGA-FD-A5BT | | 0.014179566 | | 0.016122567 | | 0 | 0.107455517 |
| TCGA_TCGA-FD-A6TF | | 0.043034351 | | 0 | | 0 | 0.027285671 |
| TCGA_TCGA-XF-A8HF | | 0.153864837 | | 0.035313111 | | 0 | 0.04850113 |
| TCGA_TCGA-FD-A43S | | 0.197027149 | | 0 | | 0 | 0.024967743 |
| TCGA_TCGA-K4-A83P | | 0.150023732 | | 0 | | 0 | 0.006532768 |
| TCGA_TCGA-DK-A2I1 | | 0.126809549 | | 0.02223802 | | 0 | 0.044515253 |
| TCGA_TCGA-KQ-A41P | | 0.06001363 | | 0.014652241 | | 0 | 0.042658866 |
| TCGA_TCGA-UY-A9PH | | 0.059859062 | | 0 | | 0.052307174 | 0.015398822 |
| TCGA_TCGA-FD-A5BY | | 0.024839626 | | 0.065248655 | | 0 | 0.052505642 |
| TCGA_TCGA-E7-A519 | | 0.27305504 | | 0 | | 0.011261566 | 0 |
| TCGA_TCGA-DK-A3IU | | 0.086408243 | | 0.044168258 | | 0 | 0.004953283 |
| TCGA_TCGA-FD-A3SP | | 0.044061509 | | 0 | | 0 | 0 |
| TCGA_TCGA-GC-A6I3 | | 0.12756612 | | 0.078914439 | | 0 | 0 |
| TCGA_TCGA-GC-A3RC | | 0.06348719 | | 0.040273426 | | 0 | 0.034398541 |
| TCGA_TCGA-GU-AATO | | 0.058679519 | | 0.000855437 | | 0 | 0.025545213 |
| TCGA_TCGA-DK-A6AV | | 0.056177504 | | 0 | | 0 | 0 |
| TCGA_TCGA-BT-A20R | | 0.013894336 | | 0.008140672 | | 0 | 0.026121936 |
| GSE13507_GSM340606 | | 0.021894779 | | 0.020263839 | | 0 | 0.03480705 |
| GSE13507_GSM340607 | | 0.097576155 | | 0 | | 0.105843856 | 0.000173981 |
| GSE13507_GSM340610 | | 0.04019702 | | 0 | | 0.00731577 | 0.02528866 |
| GSE13507_GSM340623 | | 0.172793582 | | 0.007658808 | | 0.037309924 | 0 |
| GSE13507_GSM340636 | | 0.080424374 | | 0 | | 0.06615582 | 0.024758213 |
| GSE13507_GSM340637 | | 0.048971312 | | 0 | | 0.011646276 | 0.016857146 |
| GSE13507_GSM340645 | | 0.032985537 | | 0 | | 0.035415061 | 0 |
| GSE13507_GSM340647 | | 0 | | 0.018505882 | | 0 | 0.192146645 |
| GSE13507_GSM340650 | | 0.1113047 | | 0 | | 0.080767914 | 0.050532564 |
| GSE13507_GSM340654 | | 0.197731392 | | 0 | | 0 | 0.081884745 |
| GSE13507_GSM340655 | | 0.075020044 | | 0 | | 0.018204302 | 0 |
| GSE13507_GSM340660 | | 0 | | 0 | | 0 | 0.042003441 |
| GSE13507_GSM340673 | | 0.057286875 | | 0.02296671 | | 0 | 0.022752168 |
| GSE13507_GSM340674 | | 0 | | 0.047758634 | | 0 | 0.028286021 |
| GSE13507_GSM340675 | | 0.177498884 | | 0 | | 0.040970816 | 0.024016643 |
| GSE13507_GSM340676 | | 0.047390279 | | 0.068175602 | | 0 | 0.019358271 |
| GSE13507_GSM340677 | | 0.044848673 | | 0.020411473 | | 0.007610571 | 0.108663723 |
| GSE13507_GSM340679 | | 0.063055507 | | 0 | | 0 | 0.040511068 |
| GSE13507_GSM340681 | | 0.049486392 | | 0 | | 0 | 0.061937003 |
| GSE13507_GSM340686 | | 0.117791825 | | 0 | | 0.021730833 | 0.004887483 |
| GSE13507_GSM340687 | | 0.000885357 | | 0 | | 0.038119649 | 0.045941106 |
| GSE13507_GSM340693 | | 0.057656433 | | 0 | | 0.13139146 | 0 |
| GSE13507_GSM340694 | | 0.089942509 | | 0 | | 0 | 0.065709455 |
| GSE13507_GSM340696 | | 0 | | 0 | | 0.064893098 | 0.020686881 |
| GSE13507_GSM340697 | | 0 | | 0.104076094 | | 0.006704121 | 0 |
| GSE13507_GSM340700 | | 0.168714764 | | 0.047517935 | | 0 | 0.030087435 |
| GSE13507_GSM340702 | | 0.043257264 | | 0 | | 0.009642195 | 0.044405646 |
| GSE13507_GSM340705 | | 0.033332279 | | 0 | | 0.035464832 | 0 |
| GSE13507_GSM340708 | | 0.075674638 | | 0 | | 0.028537628 | 0.01031846 |
| GSE13507_GSM340722 | | 0.109035455 | | 0 | | 0.101237717 | 0 |
| GSE13507_GSM340723 | | 0.058984753 | | 0 | | 0.035137228 | 0.014041386 |
| GSE13507_GSM340725 | | 0.020111323 | | 0 | | 0.003321941 | 0.045643852 |
| GSE13507_GSM340727 | | 0.064601131 | | 0 | | 0 | 0.050304418 |
| GSE13507_GSM340728 | | 0.002678194 | | 0 | | 0 | 0.023965488 |
| GSE13507_GSM340730 | | 0.089168447 | | 0 | | 0 | 0.047663729 |
| GSE13507_GSM340732 | | 0.020646777 | | 0 | | 0.150526096 | 0.001012102 |
| GSE13507_GSM340741 | | 0.139873454 | | 0 | | 0.045328963 | 0 |
| GSE13507_GSM340742 | | 0 | | 0 | | 0 | 0.085020039 |
| GSE13507_GSM340744 | | 0.096341785 | | 0.064174051 | | 0 | 0.02902725 |
| GSE13507_GSM340745 | | 0.016765586 | | 0 | | 0.053203698 | 0.026596034 |
| GSE13507_GSM340746 | | 0 | | 0 | | 0.028717444 | 0 |
| GSE13507_GSM340751 | | 0.027809405 | | 0.080971078 | | 0 | 0.00341738 |
| GSE13507_GSM340752 | | 0.028577448 | | 0 | | 0 | 0.031812061 |
| GSE13507_GSM340758 | | 0 | | 0 | | 0 | 0.052641224 |
| GSE13507_GSM340763 | | 0.04505094 | | 0.010615919 | | 0 | 0.058848391 |
| GSE13507_GSM340769 | | 0.03525825 | | 0 | | 0 | 0.041256089 |
| id | | Monocytes | | Macrophages M0 | | Macrophages M1 | Macrophages M2 |
| TCGA_TCGA-ZF-A9R7 | | 0.011283505 | | 0.006492852 | | 0.101409926 | 0.043086881 |
| TCGA_TCGA-E7-A97P | | 0.00572419 | | 0.037518901 | | 0.133528255 | 0.063827117 |
| TCGA_TCGA-4Z-AA81 | | 0.008895318 | | 0 | | 0.006509558 | 0.092892783 |
| TCGA_TCGA-XF-A9SM | | 0.003637594 | | 0.052021987 | | 0.092288924 | 0.221839287 |
| TCGA_TCGA-DK-A3IN | | 0 | | 0.161734025 | | 0.059651537 | 0.299327649 |
| TCGA_TCGA-DK-A2I4 | | 0.014629432 | | 0.055285292 | | 0.099806332 | 0.227131176 |
| TCGA_TCGA-XF-AAMT | | 0 | | 0.230552681 | | 0.003824322 | 0.424713728 |
| TCGA_TCGA-XF-A9SU | | 0.042418531 | | 0.101586937 | | 0.17387952 | 0.134150156 |
| TCGA_TCGA-FD-A6TK | | 0.009419798 | | 0 | | 0.124942333 | 0.246536009 |
| TCGA_TCGA-XF-A9T5 | | 0 | | 0.231778152 | | 0.07209599 | 0.146383177 |
| TCGA_TCGA-XF-A9SX | | 0.011856244 | | 0.049674883 | | 0.110842001 | 0.202660791 |
| TCGA_TCGA-E7-A7DV | | 0.039336615 | | 0.139301258 | | 0.05794521 | 0.472786859 |
| TCGA_TCGA-DK-AA6L | | 0.00950208 | | 0.226728473 | | 0.031984685 | 0.145165659 |
| TCGA_TCGA-XF-A9T4 | | 0 | | 0.259788234 | | 0.132042676 | 0.111868768 |
| TCGA_TCGA-UY-A8OB | | 0.013197134 | | 0.118705719 | | 0.018339059 | 0.087793576 |
| TCGA_TCGA-DK-A1A5 | | 0.015649827 | | 0.253026475 | | 0.104650793 | 0.027271443 |
| TCGA_TCGA-SY-A9G0 | | 0 | | 0.030891463 | | 0.011095832 | 0.105032378 |
| TCGA_TCGA-FD-A5BX | | 0.009175115 | | 0.361118271 | | 0.026266545 | 0.315645905 |
| TCGA_TCGA-ZF-AA54 | | 0 | | 0.227894102 | | 0.059561247 | 0.460677464 |
| TCGA_TCGA-GU-A766 | | 0.041994522 | | 0.035624803 | | 0.144929009 | 0.071695526 |
| TCGA_TCGA-C4-A0F1 | | 0 | | 0.208592254 | | 0.15692127 | 0.067705681 |
| TCGA_TCGA-G2-A2ES | | 0 | | 0.227619134 | | 0.087870747 | 0.153275314 |
| TCGA_TCGA-XF-AAN4 | | 0.053483234 | | 0 | | 0.12063442 | 0.203553327 |
| TCGA_TCGA-4Z-AA7N | | 0 | | 0.109526677 | | 0.031846565 | 0.029417787 |
| TCGA_TCGA-UY-A8OC | | 0 | | 0.570847699 | | 0.021432816 | 0.203512002 |
| TCGA_TCGA-GV-A3JV | | 0.02153192 | | 0.080790068 | | 0.17021544 | 0.061628879 |
| TCGA_TCGA-XF-A9T8 | | 0 | | 0.135386242 | | 0.109792989 | 0.1273552 |
| TCGA_TCGA-E7-A7XN | | 0.009207497 | | 0 | | 0.122938995 | 0.081496136 |
| TCGA_TCGA-4Z-AA7W | | 0.009957271 | | 0 | | 0.055555637 | 0.096824831 |
| TCGA_TCGA-FD-A43U | | 0.046899543 | | 0.521356229 | | 0.023916586 | 0.090389485 |
| TCGA_TCGA-ZF-AA4V | | 0 | | 0.091596599 | | 0.112679281 | 0.164347405 |
| TCGA_TCGA-ZF-AA53 | | 0 | | 0.075420531 | | 0.149574575 | 0.205548559 |
| TCGA_TCGA-BL-A13I | | 0.000113296 | | 0.029487927 | | 0.035048561 | 0.591331321 |
| TCGA_TCGA-CU-A72E | | 0 | | 0.400799957 | | 0.031702753 | 0.220755774 |
| TCGA_TCGA-GC-A3I6 | | 0 | | 0.114105667 | | 0.105977141 | 0.134272907 |
| TCGA_TCGA-FD-A6TA | | 0.001431771 | | 0.109002442 | | 0.118525549 | 0.158997018 |
| TCGA_TCGA-BL-A3JM | | 0 | | 0.338576617 | | 0.146894487 | 0.043680387 |
| TCGA_TCGA-BL-A5ZZ | | 0 | | 0.383230849 | | 0 | 0.252407241 |
| TCGA_TCGA-FD-A62N | | 0.001842644 | | 0.053851638 | | 0.144043604 | 0.127678461 |
| TCGA_TCGA-BT-A20J | | 0.0238866 | | 0.092932285 | | 0.148436115 | 0.161116371 |
| TCGA_TCGA-FT-A61P | | 0 | | 0.168722938 | | 0.086111665 | 0.285845677 |
| TCGA_TCGA-BT-A20O | | 0 | | 0 | | 0.123993575 | 0.208494679 |
| TCGA_TCGA-4Z-AA82 | | 0.00838368 | | 0.059110598 | | 0 | 0.068067833 |
| TCGA_TCGA-FD-A5BU | | 0.052215556 | | 0.078703056 | | 0.110799527 | 0.125774394 |
| TCGA_TCGA-C4-A0F0 | | 0 | | 0.109058314 | | 0.084048878 | 0.149389522 |
| TCGA_TCGA-FD-A3N5 | | 0 | | 0.409832996 | | 0.044186816 | 0.086918342 |
| TCGA_TCGA-DK-AA6S | | 0 | | 0.103119504 | | 0.147623431 | 0.131980206 |
| TCGA_TCGA-BT-A42E | | 0 | | 0.0948711 | | 0.118087635 | 0.160955812 |
| TCGA_TCGA-ZF-AA4R | | 0 | | 0.376699389 | | 0.154113034 | 0.027204991 |
| TCGA_TCGA-DK-A3WY | | 0 | | 0.0785752 | | 0.037670124 | 0.047650903 |
| TCGA_TCGA-G2-A2EF | | 0 | | 0.091037176 | | 0.167982884 | 0.140594817 |
| TCGA_TCGA-GU-AATQ | | 0.02358959 | | 0.202860141 | | 0.105182038 | 0.191523752 |
| TCGA_TCGA-YC-A8S6 | | 0.072471972 | | 0 | | 0.006718376 | 0.080258059 |
| TCGA_TCGA-XF-A9SJ | | 0.009781143 | | 0.11254215 | | 0.138305636 | 0.189594835 |
| TCGA_TCGA-DK-AA6T | | 0 | | 0 | | 0 | 0.062081217 |
| TCGA_TCGA-XF-AAMW | | 0.000200725 | | 0.115123711 | | 0.087346957 | 0.191223078 |
| TCGA_TCGA-BT-A3PJ | | 0 | | 0.163495264 | | 0.149423397 | 0.140006581 |
| TCGA_TCGA-GC-A3OO | | 0 | | 0.491607537 | | 0.023097117 | 0.173698712 |
| TCGA_TCGA-XF-AAN5 | | 0 | | 0.068552025 | | 0.089851807 | 0.145956816 |
| TCGA_TCGA-FD-A6TB | | 0 | | 0.104623948 | | 0.095646918 | 0.133805394 |
| TCGA_TCGA-XF-AAN3 | | 0.010564457 | | 0 | | 0.016703446 | 0.209527428 |
| TCGA_TCGA-FD-A3B8 | | 0.00715381 | | 0.106754107 | | 0.168793516 | 0.186213524 |
| TCGA_TCGA-XF-A9SY | | 0 | | 0.22750724 | | 0.134448638 | 0.121588081 |
| TCGA_TCGA-CU-A0YN | | 0 | | 0.269761542 | | 0 | 0.090357743 |
| TCGA_TCGA-FD-A5BZ | | 0 | | 0.481672944 | | 0.093186454 | 0.081883313 |
| TCGA_TCGA-XF-A8HD | | 0.007410687 | | 0 | | 0.013497926 | 0.059533518 |
| TCGA_TCGA-GV-A3JX | | 0 | | 0.019014015 | | 0.119697832 | 0.24939683 |
| TCGA_TCGA-FD-A3B6 | | 0.004325247 | | 0.152802743 | | 0.075417352 | 0.185321073 |
| TCGA_TCGA-GU-A764 | | 0.027773899 | | 0.097576478 | | 0.126678403 | 0.146005462 |
| TCGA_TCGA-5N-A9KM | | 0.086249451 | | 0 | | 0.055719351 | 0.220870279 |
| TCGA_TCGA-FD-A6TD | | 0.007103497 | | 0.074242156 | | 0.102774118 | 0.213885557 |
| TCGA_TCGA-ZF-AA56 | | 0.010458424 | | 0.080030956 | | 0.045358393 | 0.167173335 |
| TCGA_TCGA-ZF-AA58 | | 0 | | 0.252264579 | | 0.128630172 | 0.267993136 |
| TCGA_TCGA-FJ-A871 | | 0 | | 0.141622388 | | 0 | 0.03682996 |
| TCGA_TCGA-ZF-A9RN | | 0.007115793 | | 0.045920137 | | 0.128250381 | 0.073466779 |
| TCGA_TCGA-YC-A89H | | 0 | | 0.335476992 | | 0.087539829 | 0.18733415 |
| TCGA_TCGA-XF-AAMQ | | 0.00796024 | | 0.026283201 | | 0.106857179 | 0.123040796 |
| TCGA_TCGA-FD-A3B4 | | 0 | | 0.187258383 | | 0.118596665 | 0.095102021 |
| TCGA_TCGA-DK-A1AB | | 0 | | 0.220520056 | | 0.227239212 | 0.04228394 |
| TCGA_TCGA-BT-A20X | | 0.00132892 | | 0.17327101 | | 0.136726252 | 0.013948149 |
| TCGA_TCGA-ZF-AA5H | | 0 | | 0.265009111 | | 0.117773522 | 0.201894517 |
| TCGA_TCGA-XF-A9T2 | | 0 | | 0.396510712 | | 0.004086716 | 0.102598326 |
| TCGA_TCGA-DK-A3WX | | 0 | | 0.219232775 | | 0.172435702 | 0 |
| TCGA_TCGA-DK-A6B2 | | 0.003589197 | | 0.050982545 | | 0.119982155 | 0.195093454 |
| TCGA_TCGA-4Z-AA86 | | 0 | | 0.242379551 | | 0.09706621 | 0.146152177 |
| TCGA_TCGA-XF-A9SL | | 0.041617052 | | 0.068899687 | | 0.012785036 | 0.135236402 |
| TCGA_TCGA-XF-AAN2 | | 0.004664372 | | 0.084088902 | | 0.100206845 | 0.135480877 |
| TCGA_TCGA-ZF-A9RD | | 0 | | 0.078052454 | | 0.159128601 | 0.140600389 |
| TCGA_TCGA-DK-A2I2 | | 0 | | 0.173394926 | | 0.053548144 | 0.113847049 |
| TCGA_TCGA-FD-A62S | | 0 | | 0.229274544 | | 0.087848214 | 0.198066213 |
| TCGA_TCGA-GD-A76B | | 0.010699154 | | 0 | | 0.113231883 | 0.081409468 |
| TCGA_TCGA-GC-A6I1 | | 0 | | 0.33028331 | | 0.058948873 | 0.139521034 |
| TCGA_TCGA-XF-A9SK | | 0.075183428 | | 0.009606797 | | 0.05876003 | 0.193473009 |
| TCGA_TCGA-DK-A1AD | | 0.009613744 | | 0.028592685 | | 0.186875765 | 0.082088301 |
| TCGA_TCGA-K4-A5RI | | 0.022823134 | | 0.001047274 | | 0.002679667 | 0.103450202 |
| TCGA_TCGA-XF-AAN8 | | 0.043102754 | | 0.016804636 | | 0.02480078 | 0.345167568 |
| TCGA_TCGA-G2-A2EJ | | 0 | | 0.171139689 | | 0.042680453 | 0.068889942 |
| TCGA_TCGA-FD-A3B7 | | 0 | | 0.216584769 | | 0.099283782 | 0.1396941 |
| TCGA_TCGA-FD-A5BS | | 0.005506619 | | 0.110876304 | | 0.11269858 | 0.089244892 |
| TCGA_TCGA-LC-A66R | | 0 | | 0.104148987 | | 0.087436996 | 0.154867676 |
| TCGA_TCGA-UY-A9PB | | 0 | | 0.175165725 | | 0.118054651 | 0.167341339 |
| TCGA_TCGA-K4-A54R | | 0 | | 0.077747877 | | 0.137033915 | 0.129095307 |
| TCGA_TCGA-FD-A43P | | 0 | | 0.115343853 | | 0.173455412 | 0.040291505 |
| TCGA_TCGA-DK-A3IV | | 0.019354319 | | 0.059747748 | | 0.145304447 | 0.01647071 |
| TCGA_TCGA-GU-A762 | | 0.004377244 | | 0 | | 0.121567011 | 0.196144244 |
| TCGA_TCGA-XF-AAME | | 0.01516728 | | 0.084906854 | | 0.008786012 | 0.257937215 |
| TCGA_TCGA-FD-A62P | | 0 | | 0.356274364 | | 0.067223482 | 0.145008007 |
| TCGA_TCGA-BT-A42F | | 0 | | 0.297037721 | | 0.050856783 | 0.116441223 |
| TCGA_TCGA-ZF-A9RF | | 0.011541924 | | 0 | | 0.121899526 | 0.219507172 |
| TCGA_TCGA-BT-A20U | | 0 | | 0.287379057 | | 0.079719841 | 0.214542803 |
| TCGA_TCGA-XF-A9SW | | 0.018730827 | | 0 | | 0.025907853 | 0.34225156 |
| TCGA_TCGA-FD-A3B3 | | 0 | | 0.154335195 | | 0.174696229 | 0.111285399 |
| TCGA_TCGA-E7-A6MD | | 0.015839881 | | 0.053649997 | | 0.047401902 | 0.093135965 |
| TCGA_TCGA-BT-A0YX | | 0.004893169 | | 0.018096737 | | 0.086526553 | 0.157441991 |
| TCGA_TCGA-XF-A9T3 | | 0 | | 0.304127063 | | 0.112709406 | 0.186751626 |
| TCGA_TCGA-PQ-A6FN | | 0 | | 0.290498265 | | 0.088523675 | 0.078722193 |
| TCGA_TCGA-E7-A3X6 | | 0 | | 0.026366476 | | 0.120958851 | 0.119238406 |
| TCGA_TCGA-DK-A3WW | | 0 | | 0.024641767 | | 0.144554015 | 0.122485593 |
| TCGA_TCGA-K4-A3WS | | 0 | | 0.061672693 | | 0.202858466 | 0.123181622 |
| TCGA_TCGA-GC-A3YS | | 0 | | 0.142790961 | | 0.0014129 | 0.185890001 |
| TCGA_TCGA-DK-A1AF | | 0 | | 0.275176585 | | 0.076888613 | 0.1357263 |
| TCGA_TCGA-DK-AA74 | | 0 | | 0.149634336 | | 0.141680253 | 0.201941169 |
| TCGA_TCGA-G2-A2EO | | 0 | | 0.162555011 | | 0.081275522 | 0.133886352 |
| TCGA_TCGA-UY-A78K | | 0.018990728 | | 0.08521476 | | 0.151147168 | 0.086590482 |
| TCGA_TCGA-FD-A6TH | | 0 | | 0.247298056 | | 0.103913522 | 0.161533964 |
| TCGA_TCGA-DK-AA6W | | 0 | | 0.183087844 | | 0.086567554 | 0.119520409 |
| TCGA_TCGA-DK-A1A6 | | 0 | | 0 | | 0 | 0.176829976 |
| TCGA_TCGA-E7-A541 | | 0.005818106 | | 0.008893718 | | 0.053385406 | 0.114930277 |
| TCGA_TCGA-XF-A8HE | | 0 | | 0.009162734 | | 0.119568917 | 0.123454648 |
| TCGA_TCGA-DK-AA6Q | | 0.044241693 | | 0.000975451 | | 0.116588636 | 0.04781818 |
| TCGA_TCGA-GC-A3WC | | 0 | | 0.090140346 | | 0.173134642 | 0.106529162 |
| TCGA_TCGA-K4-A4AC | | 0 | | 0.15448308 | | 0.110957837 | 0.250076929 |
| TCGA_TCGA-FD-A3SL | | 0 | | 0.181792901 | | 0.156212549 | 0.214157618 |
| TCGA_TCGA-K4-A5RH | | 0.000726533 | | 0 | | 0.077715583 | 0.254812339 |
| TCGA_TCGA-FD-A5C1 | | 0 | | 0.091541984 | | 0.113130673 | 0.239390787 |
| TCGA_TCGA-2F-A9KO | | 0.012744452 | | 0.061988449 | | 0.097271044 | 0.107162612 |
| TCGA_TCGA-DK-A3IM | | 0 | | 0.521142329 | | 0.029669467 | 0.048114082 |
| TCGA_TCGA-XF-A9T6 | | 0.00373128 | | 0.111868255 | | 0 | 0.206384886 |
| TCGA_TCGA-FD-A43Y | | 0 | | 0.260084093 | | 0.173102739 | 0.06711727 |
| TCGA_TCGA-GV-A3QG | | 0 | | 0.19358997 | | 0.143236271 | 0.083309131 |
| TCGA_TCGA-DK-AA6M | | 0.000545222 | | 0 | | 0.058507883 | 0.25484308 |
| TCGA_TCGA-ZF-AA52 | | 0 | | 0.335461799 | | 0.030035943 | 0.233391951 |
| TCGA_TCGA-K4-A5RJ | | 0 | | 0.0022086 | | 0.108529309 | 0.33571003 |
| TCGA_TCGA-BT-A3PK | | 0 | | 0.308249419 | | 0.086372972 | 0.082001735 |
| TCGA_TCGA-FD-A3N6 | | 0.00329488 | | 0.266826281 | | 0.239821425 | 0 |
| TCGA_TCGA-UY-A78P | | 0 | | 0.359627451 | | 0.091949042 | 0.174784596 |
| TCGA_TCGA-BT-A20Q | | 0.014018943 | | 0.017637936 | | 0.132273486 | 0.136198088 |
| TCGA_TCGA-CU-A0YR | | 0 | | 0.347664827 | | 0.071136585 | 0.13278074 |
| TCGA_TCGA-SY-A9G5 | | 0.009451106 | | 0.050771536 | | 0 | 0.173854633 |
| TCGA_TCGA-4Z-AA7Q | | 0.017497341 | | 0.037858073 | | 0.2197175 | 0.09324016 |
| TCGA_TCGA-FD-A5BT | | 0.007441891 | | 0.069841655 | | 0.1146832 | 0.271950578 |
| TCGA_TCGA-FD-A6TF | | 0 | | 0.174159318 | | 0.120757843 | 0.173693964 |
| TCGA_TCGA-XF-A8HF | | 0.006034053 | | 0.128508899 | | 0.036878371 | 0.306548296 |
| TCGA_TCGA-FD-A43S | | 0.013768054 | | 0.013149004 | | 0.011117915 | 0.159089717 |
| TCGA_TCGA-K4-A83P | | 0.031452098 | | 0.085912994 | | 0.066306552 | 0.047768389 |
| TCGA_TCGA-DK-A2I1 | | 0 | | 0.112154351 | | 0.150966774 | 0.147630507 |
| TCGA_TCGA-KQ-A41P | | 0 | | 0.478186382 | | 0.042934128 | 0.139086352 |
| TCGA_TCGA-UY-A9PH | | 0.025034115 | | 0 | | 0.154663228 | 0.174239112 |
| TCGA_TCGA-FD-A5BY | | 0 | | 0.281614889 | | 0.042026024 | 0.133851916 |
| TCGA_TCGA-E7-A519 | | 0.110136517 | | 0.087924375 | | 0.001556014 | 0.077371143 |
| TCGA_TCGA-DK-A3IU | | 0 | | 0.137535976 | | 0.133117709 | 0.070615753 |
| TCGA_TCGA-FD-A3SP | | 0 | | 0.343751156 | | 0.103973787 | 0.156147272 |
| TCGA_TCGA-GC-A6I3 | | 0 | | 0.137669398 | | 0.05925077 | 0.108864085 |
| TCGA_TCGA-GC-A3RC | | 0 | | 0.398416944 | | 0.059289728 | 0.230727604 |
| TCGA_TCGA-GU-AATO | | 0 | | 0.04856862 | | 0.132326233 | 0.171432997 |
| TCGA_TCGA-DK-A6AV | | 0.027654241 | | 0.05343581 | | 0.08804873 | 0.050285411 |
| TCGA_TCGA-BT-A20R | | 0 | | 0.658835178 | | 0.0253262 | 0.089375917 |
| GSE13507_GSM340606 | | 0 | | 0.137344771 | | 0 | 0 |
| GSE13507_GSM340607 | | 0.017955568 | | 0.020106594 | | 0.02885387 | 0.090478 |
| GSE13507_GSM340610 | | 0.011131998 | | 0.141045564 | | 0.14357673 | 0 |
| GSE13507_GSM340623 | | 0 | | 0.325372056 | | 0.083392803 | 0.163919678 |
| GSE13507_GSM340636 | | 0.02365466 | | 0.047210842 | | 0.047067761 | 0.064561979 |
| GSE13507_GSM340637 | | 0.034906092 | | 0 | | 0.073117545 | 0.316996223 |
| GSE13507_GSM340645 | | 0 | | 0.161912613 | | 0.207405576 | 0.052746123 |
| GSE13507_GSM340647 | | 0 | | 0.064885018 | | 0.020842145 | 0.024336186 |
| GSE13507_GSM340650 | | 0.000194546 | | 0.181430554 | | 0.06024528 | 0.094236889 |
| GSE13507_GSM340654 | | 0 | | 0.230759955 | | 0.086771508 | 0.078424994 |
| GSE13507_GSM340655 | | 0.00887036 | | 0.175360391 | | 0.057730835 | 0.066682361 |
| GSE13507_GSM340660 | | 0.099974133 | | 0 | | 0.008379639 | 0.375388191 |
| GSE13507_GSM340673 | | 0 | | 0.327824387 | | 0.024105214 | 0.053785161 |
| GSE13507_GSM340674 | | 0.021776414 | | 0 | | 0.023636395 | 0.344862286 |
| GSE13507_GSM340675 | | 0.02141605 | | 0.076263461 | | 0.027186037 | 0.097581897 |
| GSE13507_GSM340676 | | 0 | | 0.026021943 | | 0.175339127 | 0.173357236 |
| GSE13507_GSM340677 | | 0.001677246 | | 0.071147806 | | 0.080934482 | 0.169650542 |
| GSE13507_GSM340679 | | 0 | | 0.080306583 | | 0.119420708 | 0.11487213 |
| GSE13507_GSM340681 | | 0.00881663 | | 0.031797994 | | 0.072292568 | 0.175425315 |
| GSE13507_GSM340686 | | 0.057942877 | | 0 | | 0.035809977 | 0 |
| GSE13507_GSM340687 | | 0 | | 0.192430057 | | 0.107883968 | 0.201320476 |
| GSE13507_GSM340693 | | 0 | | 0.207018363 | | 0.081900872 | 0.056753519 |
| GSE13507_GSM340694 | | 0.003830949 | | 0.074607466 | | 0.053389366 | 0.245700407 |
| GSE13507_GSM340696 | | 0 | | 0.333742646 | | 0.053131078 | 0.118598741 |
| GSE13507_GSM340697 | | 0 | | 0.139186986 | | 0.103116209 | 0.063540125 |
| GSE13507_GSM340700 | | 0.029539512 | | 0.106352292 | | 0.145919971 | 0.059986722 |
| GSE13507_GSM340702 | | 0.001100254 | | 0.100485493 | | 0.115768231 | 0.102709695 |
| GSE13507_GSM340705 | | 0 | | 0.328170914 | | 0 | 0.075900414 |
| GSE13507_GSM340708 | | 0 | | 0.079289874 | | 0.170247912 | 0.074401594 |
| GSE13507_GSM340722 | | 0 | | 0.476521173 | | 0.064370276 | 0.064718974 |
| GSE13507_GSM340723 | | 0.015420357 | | 0.028634521 | | 0.017183919 | 0.078720907 |
| GSE13507_GSM340725 | | 0 | | 0.226487321 | | 0.159867548 | 0.087793357 |
| GSE13507_GSM340727 | | 0.098276952 | | 0.019951238 | | 0.048955853 | 0.126888614 |
| GSE13507_GSM340728 | | 0 | | 0.104156323 | | 0.035705787 | 0.100293294 |
| GSE13507_GSM340730 | | 0.010566942 | | 0 | | 0.042127042 | 0.099490808 |
| GSE13507_GSM340732 | | 0.046552143 | | 0.067430444 | | 0.060147409 | 0.107730853 |
| GSE13507_GSM340741 | | 0.021279963 | | 0 | | 0.029788763 | 0.061860664 |
| GSE13507_GSM340742 | | 0.018475397 | | 0.034490595 | | 0 | 0.147874218 |
| GSE13507_GSM340744 | | 0 | | 0.09133502 | | 0.055440003 | 0.06965799 |
| GSE13507_GSM340745 | | 0 | | 0.105814585 | | 0.081800498 | 0.071556598 |
| GSE13507_GSM340746 | | 0.093021178 | | 0.067068165 | | 0.020226484 | 0.401748299 |
| GSE13507_GSM340751 | | 0 | | 0.186547179 | | 0.062429288 | 0.19510493 |
| GSE13507_GSM340752 | | 0 | | 0.1643795 | | 0.149809049 | 0.14570121 |
| GSE13507_GSM340758 | | 0.035914419 | | 0.091271773 | | 0.162702664 | 0.132886234 |
| GSE13507_GSM340763 | | 0 | | 0.058754344 | | 0.128539348 | 0.113227903 |
| GSE13507_GSM340769 | | 0.048306923 | | 0.053724687 | | 0.086223838 | 0.208332044 |
| id | | Dendritic cells resting | | Dendritic cells activated | | Mast cells resting | Mast cells activated |
| TCGA_TCGA-ZF-A9R7 | | 0.019825228 | | 0.001435532 | | 0.020513513 | 0 |
| TCGA_TCGA-E7-A97P | | 0.01122561 | | 0.009381615 | | 0.045962041 | 0 |
| TCGA_TCGA-4Z-AA81 | | 0 | | 0.120093073 | | 0.053439968 | 0 |
| TCGA_TCGA-XF-A9SM | | 0.024698235 | | 0 | | 0.059683333 | 0 |
| TCGA_TCGA-DK-A3IN | | 0.019031834 | | 0 | | 0.054885524 | 0 |
| TCGA_TCGA-DK-A2I4 | | 0.012775991 | | 0 | | 0.082836662 | 0 |
| TCGA_TCGA-XF-AAMT | | 0 | | 0.040606953 | | 0.112698777 | 0 |
| TCGA_TCGA-XF-A9SU | | 0.017503754 | | 0.008073791 | | 0.056523012 | 0 |
| TCGA_TCGA-FD-A6TK | | 0.014148759 | | 0.00803605 | | 0.071069164 | 0 |
| TCGA_TCGA-XF-A9T5 | | 0.084303831 | | 0.004551096 | | 0.010858736 | 0 |
| TCGA_TCGA-XF-A9SX | | 0.005064976 | | 0.062630078 | | 0.082451596 | 0 |
| TCGA_TCGA-E7-A7DV | | 0 | | 0.024850279 | | 0.01083305 | 0 |
| TCGA_TCGA-DK-AA6L | | 0.184139095 | | 0 | | 0.034120633 | 0 |
| TCGA_TCGA-XF-A9T4 | | 0 | | 0.039560942 | | 0 | 0 |
| TCGA_TCGA-UY-A8OB | | 0.010681232 | | 0.075185849 | | 0.057603843 | 0 |
| TCGA_TCGA-DK-A1A5 | | 0.103789544 | | 0.072220611 | | 0.059696677 | 0 |
| TCGA_TCGA-SY-A9G0 | | 0.013900128 | | 0.031832703 | | 0.297607332 | 0 |
| TCGA_TCGA-FD-A5BX | | 0.060088488 | | 0 | | 0.022723739 | 0 |
| TCGA_TCGA-ZF-AA54 | | 0 | | 0 | | 0.043599011 | 0 |
| TCGA_TCGA-GU-A766 | | 0 | | 0.111247255 | | 0.018009313 | 0 |
| TCGA_TCGA-C4-A0F1 | | 0.048746562 | | 0.025741331 | | 0.020603717 | 0 |
| TCGA_TCGA-G2-A2ES | | 0 | | 0.17675837 | | 0 | 0 |
| TCGA_TCGA-XF-AAN4 | | 0.00386838 | | 0.061952596 | | 0.347850527 | 0 |
| TCGA_TCGA-4Z-AA7N | | 0 | | 0.0187548 | | 0.019101112 | 0 |
| TCGA_TCGA-UY-A8OC | | 0.01165949 | | 0 | | 0 | 0.023996371 |
| TCGA_TCGA-GV-A3JV | | 0.06024461 | | 0 | | 0.052456851 | 0 |
| TCGA_TCGA-XF-A9T8 | | 0.028053824 | | 0.010951253 | | 0.095665493 | 0 |
| TCGA_TCGA-E7-A7XN | | 0.031496576 | | 0.024740677 | | 0.038919065 | 0 |
| TCGA_TCGA-4Z-AA7W | | 0.010899743 | | 0.068966983 | | 0.014635198 | 0 |
| TCGA_TCGA-FD-A43U | | 0 | | 0.000147157 | | 0.025093997 | 0 |
| TCGA_TCGA-ZF-AA4V | | 0 | | 0.018310303 | | 0.022565521 | 0 |
| TCGA_TCGA-ZF-AA53 | | 0.198014805 | | 0.00084493 | | 0 | 0 |
| TCGA_TCGA-BL-A13I | | 0 | | 0 | | 0.135770926 | 0 |
| TCGA_TCGA-CU-A72E | | 0.007424286 | | 0 | | 0.103645611 | 0 |
| TCGA_TCGA-GC-A3I6 | | 0 | | 0.138222686 | | 0.137553507 | 0 |
| TCGA_TCGA-FD-A6TA | | 0.025887935 | | 0 | | 0.050176311 | 0 |
| TCGA_TCGA-BL-A3JM | | 0.010213483 | | 0 | | 0.062935394 | 0 |
| TCGA_TCGA-BL-A5ZZ | | 0 | | 0.035402176 | | 0 | 0.081332506 |
| TCGA_TCGA-FD-A62N | | 0.08981207 | | 0 | | 0.057229335 | 0 |
| TCGA_TCGA-BT-A20J | | 0.016407183 | | 0.002095746 | | 0.05247651 | 0 |
| TCGA_TCGA-FT-A61P | | 0.013713076 | | 0.024472814 | | 0.057623798 | 0 |
| TCGA_TCGA-BT-A20O | | 0 | | 0 | | 0.033687584 | 0 |
| TCGA_TCGA-4Z-AA82 | | 0.201893218 | | 0.239715185 | | 0.12822158 | 0 |
| TCGA_TCGA-FD-A5BU | | 0.110441941 | | 0.023439316 | | 0.128911558 | 0 |
| TCGA_TCGA-C4-A0F0 | | 0.105034864 | | 0.130128034 | | 0.102212361 | 0 |
| TCGA_TCGA-FD-A3N5 | | 0.020944643 | | 0.019501554 | | 0 | 0.053361973 |
| TCGA_TCGA-DK-AA6S | | 0.052819119 | | 0 | | 0.054054321 | 0 |
| TCGA_TCGA-BT-A42E | | 0.110643627 | | 0.041209083 | | 0 | 0.020137533 |
| TCGA_TCGA-ZF-AA4R | | 0 | | 0.075298868 | | 0.06895436 | 0 |
| TCGA_TCGA-DK-A3WY | | 0 | | 0 | | 0.040529585 | 0 |
| TCGA_TCGA-G2-A2EF | | 0.031324007 | | 0.05027766 | | 0.012604192 | 0 |
| TCGA_TCGA-GU-AATQ | | 0.030519648 | | 0.036361423 | | 0.066147869 | 0 |
| TCGA_TCGA-YC-A8S6 | | 0.051968531 | | 0.1556068 | | 0.192237496 | 0.081982599 |
| TCGA_TCGA-XF-A9SJ | | 0 | | 0 | | 0.06589377 | 0 |
| TCGA_TCGA-DK-AA6T | | 0 | | 0.100546193 | | 0.01553 | 0 |
| TCGA_TCGA-XF-AAMW | | 0.12944448 | | 0.103605743 | | 0.040842438 | 0 |
| TCGA_TCGA-BT-A3PJ | | 0.024143607 | | 0.000812476 | | 0 | 0.080735324 |
| TCGA_TCGA-GC-A3OO | | 0.002596767 | | 0 | | 0.176779508 | 0 |
| TCGA_TCGA-XF-AAN5 | | 0.031411454 | | 0 | | 0.138505262 | 0 |
| TCGA_TCGA-FD-A6TB | | 0 | | 0.001135051 | | 0.022240911 | 0 |
| TCGA_TCGA-XF-AAN3 | | 0.215883612 | | 0.096810443 | | 0.15356469 | 0 |
| TCGA_TCGA-FD-A3B8 | | 0 | | 0.003435963 | | 0.105935367 | 0 |
| TCGA_TCGA-XF-A9SY | | 0 | | 0 | | 0 | 0.104079813 |
| TCGA_TCGA-CU-A0YN | | 0.000284486 | | 0.112365834 | | 0 | 0.122468564 |
| TCGA_TCGA-FD-A5BZ | | 0 | | 0 | | 0.11142576 | 0 |
| TCGA_TCGA-XF-A8HD | | 0 | | 0.190462058 | | 0.10214096 | 0 |
| TCGA_TCGA-GV-A3JX | | 0.021175789 | | 0.086438166 | | 0.024361454 | 0 |
| TCGA_TCGA-FD-A3B6 | | 7.05E-05 | | 0 | | 0.027933453 | 0 |
| TCGA_TCGA-GU-A764 | | 0.043686895 | | 0 | | 0.067166619 | 0 |
| TCGA_TCGA-5N-A9KM | | 0 | | 0.035309309 | | 0.075670743 | 0 |
| TCGA_TCGA-FD-A6TD | | 0.11510284 | | 0.005635718 | | 0.028299424 | 0 |
| TCGA_TCGA-ZF-AA56 | | 0.009445975 | | 0.06247213 | | 0.144861538 | 0 |
| TCGA_TCGA-ZF-AA58 | | 0 | | 0.001457041 | | 0 | 0 |
| TCGA_TCGA-FJ-A871 | | 0 | | 0.023552289 | | 0.054804303 | 0 |
| TCGA_TCGA-ZF-A9RN | | 0 | | 0 | | 0.039523508 | 0 |
| TCGA_TCGA-YC-A89H | | 0.055949314 | | 0 | | 0 | 0.016438037 |
| TCGA_TCGA-XF-AAMQ | | 0.031253694 | | 0.082002168 | | 0.038909944 | 0 |
| TCGA_TCGA-FD-A3B4 | | 0 | | 0.017463073 | | 0.136767025 | 0 |
| TCGA_TCGA-DK-A1AB | | 0.08088172 | | 0 | | 0.060050054 | 0 |
| TCGA_TCGA-BT-A20X | | 0.014486015 | | 0.062981444 | | 0.040828419 | 0 |
| TCGA_TCGA-ZF-AA5H | | 0.001534899 | | 0.009845378 | | 0 | 0 |
| TCGA_TCGA-XF-A9T2 | | 0 | | 0 | | 0.244115286 | 0 |
| TCGA_TCGA-DK-A3WX | | 0.027651537 | | 0 | | 0.06557784 | 0 |
| TCGA_TCGA-DK-A6B2 | | 0.031500985 | | 0.103857769 | | 0.086103137 | 0 |
| TCGA_TCGA-4Z-AA86 | | 0 | | 0.011635038 | | 0 | 0.044447737 |
| TCGA_TCGA-XF-A9SL | | 0 | | 0.020393156 | | 0.175506313 | 0 |
| TCGA_TCGA-XF-AAN2 | | 0 | | 0.013814074 | | 0.040956799 | 0 |
| TCGA_TCGA-ZF-A9RD | | 0.038280145 | | 0.006693009 | | 0.101096099 | 0 |
| TCGA_TCGA-DK-A2I2 | | 0.14916966 | | 0 | | 0 | 0.270143397 |
| TCGA_TCGA-FD-A62S | | 0.122609771 | | 0.008520957 | | 0.063056574 | 0 |
| TCGA_TCGA-GD-A76B | | 0.112562615 | | 0.020042217 | | 0 | 0 |
| TCGA_TCGA-GC-A6I1 | | 0.006778772 | | 0 | | 0 | 0.075019449 |
| TCGA_TCGA-XF-A9SK | | 0.03655291 | | 0.119131882 | | 0.090817208 | 0 |
| TCGA_TCGA-DK-A1AD | | 0.026341796 | | 0.077252334 | | 0 | 0 |
| TCGA_TCGA-K4-A5RI | | 0.229185407 | | 0.03283725 | | 0.228888561 | 0 |
| TCGA_TCGA-XF-AAN8 | | 0 | | 0.00276927 | | 0.216089202 | 0 |
| TCGA_TCGA-G2-A2EJ | | 0 | | 0.294448735 | | 0 | 0.06331963 |
| TCGA_TCGA-FD-A3B7 | | 0.002780463 | | 0 | | 0 | 0.016416235 |
| TCGA_TCGA-FD-A5BS | | 0.006462751 | | 0 | | 0.021334414 | 0 |
| TCGA_TCGA-LC-A66R | | 0.003207851 | | 0.059053723 | | 0.070216723 | 0 |
| TCGA_TCGA-UY-A9PB | | 0.023729054 | | 0 | | 0.092692893 | 0 |
| TCGA_TCGA-K4-A54R | | 0.009841526 | | 0.011857802 | | 0.09052813 | 0 |
| TCGA_TCGA-FD-A43P | | 0 | | 0.005720467 | | 0.042318825 | 0 |
| TCGA_TCGA-DK-A3IV | | 0 | | 0.080972579 | | 0.075836355 | 0 |
| TCGA_TCGA-GU-A762 | | 0.001085464 | | 0.025696573 | | 0.038704762 | 0 |
| TCGA_TCGA-XF-AAME | | 0.057773634 | | 0.074677587 | | 0.055369442 | 0 |
| TCGA_TCGA-FD-A62P | | 0 | | 0.029484373 | | 0.085268004 | 0 |
| TCGA_TCGA-BT-A42F | | 0.001873184 | | 0.016310779 | | 0 | 0.10287457 |
| TCGA_TCGA-ZF-A9RF | | 0.032731789 | | 0 | | 0 | 0.019035756 |
| TCGA_TCGA-BT-A20U | | 0.031700487 | | 0.02416512 | | 0.020489183 | 0 |
| TCGA_TCGA-XF-A9SW | | 0.019988378 | | 0.085562881 | | 0.210519292 | 0 |
| TCGA_TCGA-FD-A3B3 | | 0.003641383 | | 0.009643857 | | 0.08664431 | 0 |
| TCGA_TCGA-E7-A6MD | | 0.04430779 | | 0.018801221 | | 0.070842222 | 0 |
| TCGA_TCGA-BT-A0YX | | 0.042504359 | | 0.051597674 | | 0.102364781 | 0 |
| TCGA_TCGA-XF-A9T3 | | 0 | | 0 | | 0.082921986 | 0 |
| TCGA_TCGA-PQ-A6FN | | 0.025827256 | | 0.009843848 | | 0.048171934 | 0 |
| TCGA_TCGA-E7-A3X6 | | 0.093836171 | | 0.033348908 | | 0.066263393 | 0 |
| TCGA_TCGA-DK-A3WW | | 0.005127795 | | 0.011962434 | | 0.028304768 | 0 |
| TCGA_TCGA-K4-A3WS | | 0.013656787 | | 0.035968458 | | 0.15558093 | 0 |
| TCGA_TCGA-GC-A3YS | | 0.070403053 | | 0.149972047 | | 0.071272564 | 0 |
| TCGA_TCGA-DK-A1AF | | 0.014981749 | | 0.021509422 | | 0.127717043 | 0 |
| TCGA_TCGA-DK-AA74 | | 0.057253082 | | 0 | | 0.050227598 | 0 |
| TCGA_TCGA-G2-A2EO | | 0.013967591 | | 0 | | 0.028253674 | 0 |
| TCGA_TCGA-UY-A78K | | 0.018418197 | | 0 | | 0.042173435 | 0 |
| TCGA_TCGA-FD-A6TH | | 0 | | 0 | | 0.030059741 | 0 |
| TCGA_TCGA-DK-AA6W | | 0.333540716 | | 0 | | 0 | 0 |
| TCGA_TCGA-DK-A1A6 | | 0 | | 0.325557673 | | 0 | 0 |
| TCGA_TCGA-E7-A541 | | 0.090906335 | | 0.017051862 | | 0.063904587 | 0 |
| TCGA_TCGA-XF-A8HE | | 0.00087589 | | 0.052887825 | | 0.008194783 | 0 |
| TCGA_TCGA-DK-AA6Q | | 0.108617326 | | 0 | | 0.060252816 | 0 |
| TCGA_TCGA-GC-A3WC | | 0.122108921 | | 0 | | 0.095841712 | 0 |
| TCGA_TCGA-K4-A4AC | | 0.064083017 | | 0 | | 0.059390558 | 0 |
| TCGA_TCGA-FD-A3SL | | 0.034020405 | | 0 | | 0.123992246 | 0 |
| TCGA_TCGA-K4-A5RH | | 0 | | 0 | | 0.014263565 | 0 |
| TCGA_TCGA-FD-A5C1 | | 0.021468314 | | 0.034197882 | | 0.04438406 | 0 |
| TCGA_TCGA-2F-A9KO | | 0.011866871 | | 0.039815028 | | 0.085287753 | 0 |
| TCGA_TCGA-DK-A3IM | | 0 | | 0.065906043 | | 0 | 0.068494105 |
| TCGA_TCGA-XF-A9T6 | | 0.103575201 | | 0.236329922 | | 0.029379448 | 0 |
| TCGA_TCGA-FD-A43Y | | 0.21956844 | | 0.034907267 | | 0.031351791 | 0 |
| TCGA_TCGA-GV-A3QG | | 0 | | 0 | | 0.050087436 | 0 |
| TCGA_TCGA-DK-AA6M | | 0.180076241 | | 0.005983997 | | 0.107219134 | 0 |
| TCGA_TCGA-ZF-AA52 | | 0.051598362 | | 0.016022225 | | 0 | 0.027362232 |
| TCGA_TCGA-K4-A5RJ | | 0.026580009 | | 0 | | 0.053199289 | 0 |
| TCGA_TCGA-BT-A3PK | | 0 | | 0.121260343 | | 0 | 0.101902744 |
| TCGA_TCGA-FD-A3N6 | | 0.055797239 | | 0 | | 0 | 0.019408936 |
| TCGA_TCGA-UY-A78P | | 0.032619966 | | 0 | | 0.081199969 | 0 |
| TCGA_TCGA-BT-A20Q | | 0.102417159 | | 0.02048007 | | 0.079855325 | 0 |
| TCGA_TCGA-CU-A0YR | | 0.042150566 | | 0.041689853 | | 0.043058658 | 0 |
| TCGA_TCGA-SY-A9G5 | | 0.006783792 | | 0.216456157 | | 0.06542031 | 0 |
| TCGA_TCGA-4Z-AA7Q | | 0.007931332 | | 0.025084881 | | 0.063055736 | 0 |
| TCGA_TCGA-FD-A5BT | | 0.00219573 | | 0 | | 0.020467018 | 0 |
| TCGA_TCGA-FD-A6TF | | 0 | | 0.017006933 | | 0.169802815 | 0 |
| TCGA_TCGA-XF-A8HF | | 0.002899695 | | 0.081179667 | | 0.088269127 | 0 |
| TCGA_TCGA-FD-A43S | | 0 | | 0.012908283 | | 0.075765247 | 0 |
| TCGA_TCGA-K4-A83P | | 0 | | 0 | | 0.04149977 | 0 |
| TCGA_TCGA-DK-A2I1 | | 0.049914072 | | 0 | | 0.096314732 | 0 |
| TCGA_TCGA-KQ-A41P | | 0 | | 0.033334708 | | 0.108027741 | 0 |
| TCGA_TCGA-UY-A9PH | | 0.055430409 | | 0.054448067 | | 0.048891786 | 0 |
| TCGA_TCGA-FD-A5BY | | 0.091706359 | | 0.020673354 | | 0.176280288 | 0 |
| TCGA_TCGA-E7-A519 | | 0 | | 0.094428924 | | 0 | 0.030956693 |
| TCGA_TCGA-DK-A3IU | | 0 | | 0 | | 0.04642511 | 0 |
| TCGA_TCGA-FD-A3SP | | 0.049779568 | | 0 | | 0.060933869 | 0 |
| TCGA_TCGA-GC-A6I3 | | 0.016781615 | | 0.04595077 | | 0.048906273 | 0 |
| TCGA_TCGA-GC-A3RC | | 0.018647049 | | 0.009482227 | | 0.018953202 | 0 |
| TCGA_TCGA-GU-AATO | | 0.000381517 | | 0 | | 0.045104007 | 0 |
| TCGA_TCGA-DK-A6AV | | 0.054861257 | | 0.102274509 | | 0 | 0.039702318 |
| TCGA_TCGA-BT-A20R | | 0 | | 0.047017465 | | 0.064168625 | 0 |
| GSE13507_GSM340606 | | 0 | | 0.214376906 | | 0.056838102 | 0 |
| GSE13507_GSM340607 | | 0.033262711 | | 0.026529898 | | 0.012835989 | 0 |
| GSE13507_GSM340610 | | 0.153017212 | | 0.03158739 | | 0.030665203 | 0 |
| GSE13507_GSM340623 | | 0 | | 0 | | 0 | 0.063878077 |
| GSE13507_GSM340636 | | 0 | | 0.009221341 | | 0.043416617 | 0 |
| GSE13507_GSM340637 | | 0 | | 0.047870539 | | 0.018295514 | 0 |
| GSE13507_GSM340645 | | 0 | | 0.111002324 | | 0.036037177 | 0 |
| GSE13507_GSM340647 | | 0.022139325 | | 0.095410595 | | 0.122936758 | 0 |
| GSE13507_GSM340650 | | 0 | | 0.001795946 | | 0 | 0.021716247 |
| GSE13507_GSM340654 | | 0.007693377 | | 0 | | 0.063101255 | 0 |
| GSE13507_GSM340655 | | 0 | | 0.374464437 | | 0 | 0.021445116 |
| GSE13507_GSM340660 | | 0 | | 0.039781823 | | 0.204966987 | 0 |
| GSE13507_GSM340673 | | 0 | | 0.084554239 | | 0.026728367 | 0 |
| GSE13507_GSM340674 | | 0.075538731 | | 0.031141316 | | 0.258757193 | 0 |
| GSE13507_GSM340675 | | 0 | | 0.119437472 | | 0 | 0 |
| GSE13507_GSM340676 | | 0.163133516 | | 0.003284697 | | 0.107650543 | 0 |
| GSE13507_GSM340677 | | 0.031015887 | | 0.030193826 | | 0.081109849 | 0 |
| GSE13507_GSM340679 | | 0.022620358 | | 0.013920753 | | 0.062176063 | 0 |
| GSE13507_GSM340681 | | 0 | | 0.17944179 | | 0.056247424 | 0 |
| GSE13507_GSM340686 | | 0.398774076 | | 0.027387887 | | 0 | 0.015112679 |
| GSE13507_GSM340687 | | 0.034311364 | | 0 | | 0 | 0.057317113 |
| GSE13507_GSM340693 | | 0 | | 0.003838483 | | 0.017270477 | 0 |
| GSE13507_GSM340694 | | 0 | | 0.003071937 | | 0.015320463 | 0 |
| GSE13507_GSM340696 | | 0.05265114 | | 0.009386991 | | 0.036475273 | 0 |
| GSE13507_GSM340697 | | 0 | | 0.011391134 | | 0 | 0.001336233 |
| GSE13507_GSM340700 | | 0 | | 0.17857655 | | 0 | 0.023929346 |
| GSE13507_GSM340702 | | 0.035848688 | | 0.080362877 | | 0.03981611 | 0 |
| GSE13507_GSM340705 | | 0 | | 0.112341635 | | 0.024217083 | 0 |
| GSE13507_GSM340708 | | 0.112092584 | | 0 | | 0.080203449 | 0 |
| GSE13507_GSM340722 | | 0 | | 0.017236051 | | 0 | 0.039132512 |
| GSE13507_GSM340723 | | 0.014204861 | | 0.150734084 | | 0.019177509 | 0 |
| GSE13507_GSM340725 | | 0 | | 0.156644477 | | 0.055111547 | 0 |
| GSE13507_GSM340727 | | 0.007838029 | | 0.048628424 | | 0.20065765 | 0 |
| GSE13507_GSM340728 | | 0 | | 0.126430514 | | 0 | 0.355835152 |
| GSE13507_GSM340730 | | 0.307142596 | | 0.011440815 | | 0.063877571 | 0 |
| GSE13507_GSM340732 | | 0.001124465 | | 0.076315897 | | 0.036500936 | 0 |
| GSE13507_GSM340741 | | 0.003836684 | | 0.034782041 | | 0.003048376 | 0 |
| GSE13507_GSM340742 | | 0.009209552 | | 0.252471955 | | 0.085747891 | 0 |
| GSE13507_GSM340744 | | 0.018516379 | | 0.022859581 | | 0.047846536 | 0 |
| GSE13507_GSM340745 | | 0.012681989 | | 0.013907266 | | 0 | 0.002521206 |
| GSE13507_GSM340746 | | 0.007363057 | | 0.007748782 | | 0.142211598 | 0 |
| GSE13507_GSM340751 | | 0 | | 0 | | 0.077685178 | 0 |
| GSE13507_GSM340752 | | 0.038362177 | | 0.08724745 | | 0.097251213 | 0 |
| GSE13507_GSM340758 | | 0.084788897 | | 0.038187557 | | 0.121586636 | 0 |
| GSE13507_GSM340763 | | 0.00786356 | | 0.075517211 | | 0.031686446 | 0 |
| GSE13507_GSM340769 | | 0 | | 0 | | 0.159972375 | 0 |
| **id** | **Eosinophils** | | **Neutrophils** | |  | | |
| TCGA_TCGA-ZF-A9R7 | 0.00845836 | | 0 | |  | | |
| TCGA_TCGA-E7-A97P | 0 | | 0 | |  | | |
| TCGA_TCGA-4Z-AA81 | 0 | | 0 | |  | | |
| TCGA_TCGA-XF-A9SM | 0 | | 0.007061058 | |  | | |
| TCGA_TCGA-DK-A3IN | 0 | | 0.018910855 | |  | | |
| TCGA_TCGA-DK-A2I4 | 0 | | 0.00273569 | |  | | |
| TCGA_TCGA-XF-AAMT | 0 | | 0.016773304 | |  | | |
| TCGA_TCGA-XF-A9SU | 0 | | 0 | |  | | |
| TCGA_TCGA-FD-A6TK | 0 | | 0.005331486 | |  | | |
| TCGA_TCGA-XF-A9T5 | 0 | | 0 | |  | | |
| TCGA_TCGA-XF-A9SX | 0.005164732 | | 0 | |  | | |
| TCGA_TCGA-E7-A7DV | 0 | | 0.019995264 | |  | | |
| TCGA_TCGA-DK-AA6L | 0 | | 0.001222471 | |  | | |
| TCGA_TCGA-XF-A9T4 | 0.00182698 | | 0.028630443 | |  | | |
| TCGA_TCGA-UY-A8OB | 0 | | 0 | |  | | |
| TCGA_TCGA-DK-A1A5 | 0.003196587 | | 0 | |  | | |
| TCGA_TCGA-SY-A9G0 | 0 | | 0 | |  | | |
| TCGA_TCGA-FD-A5BX | 0 | | 0 | |  | | |
| TCGA_TCGA-ZF-AA54 | 0 | | 0 | |  | | |
| TCGA_TCGA-GU-A766 | 0 | | 0.007673616 | |  | | |
| TCGA_TCGA-C4-A0F1 | 0.005694086 | | 0.017886622 | |  | | |
| TCGA_TCGA-G2-A2ES | 0 | | 0.009469873 | |  | | |
| TCGA_TCGA-XF-AAN4 | 0.007764114 | | 0 | |  | | |
| TCGA_TCGA-4Z-AA7N | 0 | | 0 | |  | | |
| TCGA_TCGA-UY-A8OC | 0.023796037 | | 0 | |  | | |
| TCGA_TCGA-GV-A3JV | 0.015276503 | | 0 | |  | | |
| TCGA_TCGA-XF-A9T8 | 0 | | 0.0634654 | |  | | |
| TCGA_TCGA-E7-A7XN | 0 | | 9.68E-05 | |  | | |
| TCGA_TCGA-4Z-AA7W | 0 | | 0 | |  | | |
| TCGA_TCGA-FD-A43U | 0 | | 0 | |  | | |
| TCGA_TCGA-ZF-AA4V | 0 | | 0 | |  | | |
| TCGA_TCGA-ZF-AA53 | 0.007952169 | | 0.021897515 | |  | | |
| TCGA_TCGA-BL-A13I | 0 | | 0 | |  | | |
| TCGA_TCGA-CU-A72E | 0.01669831 | | 0 | |  | | |
| TCGA_TCGA-GC-A3I6 | 0 | | 0 | |  | | |
| TCGA_TCGA-FD-A6TA | 0 | | 0.002214893 | |  | | |
| TCGA_TCGA-BL-A3JM | 0 | | 0 | |  | | |
| TCGA_TCGA-BL-A5ZZ | 0 | | 0.008898141 | |  | | |
| TCGA_TCGA-FD-A62N | 0 | | 0.000403063 | |  | | |
| TCGA_TCGA-BT-A20J | 0 | | 0.002614589 | |  | | |
| TCGA_TCGA-FT-A61P | 0 | | 0 | |  | | |
| TCGA_TCGA-BT-A20O | 0 | | 0 | |  | | |
| TCGA_TCGA-4Z-AA82 | 0 | | 0 | |  | | |
| TCGA_TCGA-FD-A5BU | 0 | | 0 | |  | | |
| TCGA_TCGA-C4-A0F0 | 0 | | 0.025285255 | |  | | |
| TCGA_TCGA-FD-A3N5 | 0 | | 0.02459355 | |  | | |
| TCGA_TCGA-DK-AA6S | 0 | | 0.004370337 | |  | | |
| TCGA_TCGA-BT-A42E | 0 | | 0 | |  | | |
| TCGA_TCGA-ZF-AA4R | 0 | | 0 | |  | | |
| TCGA_TCGA-DK-A3WY | 0 | | 0 | |  | | |
| TCGA_TCGA-G2-A2EF | 0 | | 0 | |  | | |
| TCGA_TCGA-GU-AATQ | 0 | | 0 | |  | | |
| TCGA_TCGA-YC-A8S6 | 0 | | 0.040315978 | |  | | |
| TCGA_TCGA-XF-A9SJ | 0 | | 0 | |  | | |
| TCGA_TCGA-DK-AA6T | 0 | | 0 | |  | | |
| TCGA_TCGA-XF-AAMW | 0.001540071 | | 0.013308445 | |  | | |
| TCGA_TCGA-BT-A3PJ | 0 | | 0.012410825 | |  | | |
| TCGA_TCGA-GC-A3OO | 0 | | 0 | |  | | |
| TCGA_TCGA-XF-AAN5 | 0.016335566 | | 0.017142969 | |  | | |
| TCGA_TCGA-FD-A6TB | 0 | | 0 | |  | | |
| TCGA_TCGA-XF-AAN3 | 0 | | 0.01477123 | |  | | |
| TCGA_TCGA-FD-A3B8 | 0 | | 0.015565388 | |  | | |
| TCGA_TCGA-XF-A9SY | 0 | | 0.001616216 | |  | | |
| TCGA_TCGA-CU-A0YN | 0 | | 0.062517289 | |  | | |
| TCGA_TCGA-FD-A5BZ | 0 | | 0 | |  | | |
| TCGA_TCGA-XF-A8HD | 0 | | 0.011035036 | |  | | |
| TCGA_TCGA-GV-A3JX | 0.006133741 | | 0.000563609 | |  | | |
| TCGA_TCGA-FD-A3B6 | 0 | | 0.013706954 | |  | | |
| TCGA_TCGA-GU-A764 | 0 | | 0 | |  | | |
| TCGA_TCGA-5N-A9KM | 0 | | 0 | |  | | |
| TCGA_TCGA-FD-A6TD | 0 | | 0.020153331 | |  | | |
| TCGA_TCGA-ZF-AA56 | 0.032377632 | | 0.056354987 | |  | | |
| TCGA_TCGA-ZF-AA58 | 0 | | 0.002200473 | |  | | |
| TCGA_TCGA-FJ-A871 | 0 | | 0.003062328 | |  | | |
| TCGA_TCGA-ZF-A9RN | 0 | | 0 | |  | | |
| TCGA_TCGA-YC-A89H | 0 | | 0.003070887 | |  | | |
| TCGA_TCGA-XF-AAMQ | 0.025391953 | | 0 | |  | | |
| TCGA_TCGA-FD-A3B4 | 0 | | 0.005918244 | |  | | |
| TCGA_TCGA-DK-A1AB | 0 | | 0 | |  | | |
| TCGA_TCGA-BT-A20X | 0.004188513 | | 0.00890554 | |  | | |
| TCGA_TCGA-ZF-AA5H | 0.011873182 | | 0.001654535 | |  | | |
| TCGA_TCGA-XF-A9T2 | 0 | | 0 | |  | | |
| TCGA_TCGA-DK-A3WX | 0 | | 0.206292417 | |  | | |
| TCGA_TCGA-DK-A6B2 | 0 | | 0.006762771 | |  | | |
| TCGA_TCGA-4Z-AA86 | 0 | | 0 | |  | | |
| TCGA_TCGA-XF-A9SL | 0 | | 0 | |  | | |
| TCGA_TCGA-XF-AAN2 | 0 | | 0.007274049 | |  | | |
| TCGA_TCGA-ZF-A9RD | 0 | | 0.007667147 | |  | | |
| TCGA_TCGA-DK-A2I2 | 0 | | 0.05396329 | |  | | |
| TCGA_TCGA-FD-A62S | 0 | | 0.00749827 | |  | | |
| TCGA_TCGA-GD-A76B | 0.001657803 | | 0.004497355 | |  | | |
| TCGA_TCGA-GC-A6I1 | 0 | | 0.004934751 | |  | | |
| TCGA_TCGA-XF-A9SK | 0 | | 0.000752725 | |  | | |
| TCGA_TCGA-DK-A1AD | 0.007116586 | | 0 | |  | | |
| TCGA_TCGA-K4-A5RI | 0.002336165 | | 0 | |  | | |
| TCGA_TCGA-XF-AAN8 | 0 | | 0.103870058 | |  | | |
| TCGA_TCGA-G2-A2EJ | 0 | | 0.02403986 | |  | | |
| TCGA_TCGA-FD-A3B7 | 0 | | 0.016517338 | |  | | |
| TCGA_TCGA-FD-A5BS | 0 | | 0.005340309 | |  | | |
| TCGA_TCGA-LC-A66R | 0 | | 0.018357557 | |  | | |
| TCGA_TCGA-UY-A9PB | 0 | | 0.004016403 | |  | | |
| TCGA_TCGA-K4-A54R | 0 | | 0.0013082 | |  | | |
| TCGA_TCGA-FD-A43P | 0 | | 0 | |  | | |
| TCGA_TCGA-DK-A3IV | 0 | | 0 | |  | | |
| TCGA_TCGA-GU-A762 | 0 | | 0 | |  | | |
| TCGA_TCGA-XF-AAME | 0.005649342 | | 0.017795357 | |  | | |
| TCGA_TCGA-FD-A62P | 0 | | 0.016356922 | |  | | |
| TCGA_TCGA-BT-A42F | 0 | | 0.015070027 | |  | | |
| TCGA_TCGA-ZF-A9RF | 0 | | 0 | |  | | |
| TCGA_TCGA-BT-A20U | 0 | | 0.012040548 | |  | | |
| TCGA_TCGA-XF-A9SW | 0 | | 0.023369616 | |  | | |
| TCGA_TCGA-FD-A3B3 | 0 | | 0.005054038 | |  | | |
| TCGA_TCGA-E7-A6MD | 0 | | 0 | |  | | |
| TCGA_TCGA-BT-A0YX | 0 | | 0.029224996 | |  | | |
| TCGA_TCGA-XF-A9T3 | 0 | | 0 | |  | | |
| TCGA_TCGA-PQ-A6FN | 0 | | 0.002870161 | |  | | |
| TCGA_TCGA-E7-A3X6 | 0 | | 0.012162892 | |  | | |
| TCGA_TCGA-DK-A3WW | 0 | | 0.010572271 | |  | | |
| TCGA_TCGA-K4-A3WS | 0 | | 0 | |  | | |
| TCGA_TCGA-GC-A3YS | 0 | | 0 | |  | | |
| TCGA_TCGA-DK-A1AF | 0 | | 0.027636283 | |  | | |
| TCGA_TCGA-DK-AA74 | 0 | | 0.023812368 | |  | | |
| TCGA_TCGA-G2-A2EO | 0 | | 0 | |  | | |
| TCGA_TCGA-UY-A78K | 0 | | 0 | |  | | |
| TCGA_TCGA-FD-A6TH | 0 | | 0.00507697 | |  | | |
| TCGA_TCGA-DK-AA6W | 0 | | 0 | |  | | |
| TCGA_TCGA-DK-A1A6 | 0 | | 0.016085644 | |  | | |
| TCGA_TCGA-E7-A541 | 0 | | 0.00572252 | |  | | |
| TCGA_TCGA-XF-A8HE | 0 | | 0.146183561 | |  | | |
| TCGA_TCGA-DK-AA6Q | 0 | | 0 | |  | | |
| TCGA_TCGA-GC-A3WC | 0 | | 0 | |  | | |
| TCGA_TCGA-K4-A4AC | 0 | | 0.003479964 | |  | | |
| TCGA_TCGA-FD-A3SL | 0 | | 0 | |  | | |
| TCGA_TCGA-K4-A5RH | 0 | | 0 | |  | | |
| TCGA_TCGA-FD-A5C1 | 0 | | 0.00824206 | |  | | |
| TCGA_TCGA-2F-A9KO | 0 | | 0 | |  | | |
| TCGA_TCGA-DK-A3IM | 0 | | 0.007708386 | |  | | |
| TCGA_TCGA-XF-A9T6 | 0 | | 0.008228928 | |  | | |
| TCGA_TCGA-FD-A43Y | 0 | | 0 | |  | | |
| TCGA_TCGA-GV-A3QG | 0 | | 0.000572208 | |  | | |
| TCGA_TCGA-DK-AA6M | 0 | | 0.017170671 | |  | | |
| TCGA_TCGA-ZF-AA52 | 0 | | 0.030455372 | |  | | |
| TCGA_TCGA-K4-A5RJ | 0 | | 0 | |  | | |
| TCGA_TCGA-BT-A3PK | 0 | | 0.066821614 | |  | | |
| TCGA_TCGA-FD-A3N6 | 0.022155148 | | 0 | |  | | |
| TCGA_TCGA-UY-A78P | 0.010940342 | | 0.021347917 | |  | | |
| TCGA_TCGA-BT-A20Q | 0 | | 0 | |  | | |
| TCGA_TCGA-CU-A0YR | 0 | | 0.002595395 | |  | | |
| TCGA_TCGA-SY-A9G5 | 0.003078503 | | 0 | |  | | |
| TCGA_TCGA-4Z-AA7Q | 0 | | 0 | |  | | |
| TCGA_TCGA-FD-A5BT | 0 | | 0 | |  | | |
| TCGA_TCGA-FD-A6TF | 0 | | 0 | |  | | |
| TCGA_TCGA-XF-A8HF | 0 | | 0 | |  | | |
| TCGA_TCGA-FD-A43S | 0 | | 0 | |  | | |
| TCGA_TCGA-K4-A83P | 0 | | 0.008258835 | |  | | |
| TCGA_TCGA-DK-A2I1 | 0 | | 0.005118577 | |  | | |
| TCGA_TCGA-KQ-A41P | 0 | | 0 | |  | | |
| TCGA_TCGA-UY-A9PH | 0 | | 0.001867219 | |  | | |
| TCGA_TCGA-FD-A5BY | 0 | | 0.019246103 | |  | | |
| TCGA_TCGA-E7-A519 | 0 | | 0 | |  | | |
| TCGA_TCGA-DK-A3IU | 0 | | 0.014038977 | |  | | |
| TCGA_TCGA-FD-A3SP | 0 | | 0.004380222 | |  | | |
| TCGA_TCGA-GC-A6I3 | 0 | | 0 | |  | | |
| TCGA_TCGA-GC-A3RC | 0 | | 0 | |  | | |
| TCGA_TCGA-GU-AATO | 0 | | 0.072424829 | |  | | |
| TCGA_TCGA-DK-A6AV | 0.009764627 | | 0 | |  | | |
| TCGA_TCGA-BT-A20R | 0 | | 0 | |  | | |
| GSE13507_GSM340606 | 0.006390137 | | 0.031370206 | |  | | |
| GSE13507_GSM340607 | 0 | | 0 | |  | | |
| GSE13507_GSM340610 | 0 | | 0 | |  | | |
| GSE13507_GSM340623 | 0 | | 0.003930619 | |  | | |
| GSE13507_GSM340636 | 0 | | 0 | |  | | |
| GSE13507_GSM340637 | 0 | | 0.000214481 | |  | | |
| GSE13507_GSM340645 | 0 | | 0 | |  | | |
| GSE13507_GSM340647 | 0 | | 0.010609945 | |  | | |
| GSE13507_GSM340650 | 0 | | 0 | |  | | |
| GSE13507_GSM340654 | 0 | | 0.00207351 | |  | | |
| GSE13507_GSM340655 | 0 | | 0.012938098 | |  | | |
| GSE13507_GSM340660 | 0.009359528 | | 3.56E-05 | |  | | |
| GSE13507_GSM340673 | 0 | | 0 | |  | | |
| GSE13507_GSM340674 | 0.015846756 | | 0.014078727 | |  | | |
| GSE13507_GSM340675 | 0 | | 0.029229274 | |  | | |
| GSE13507_GSM340676 | 0 | | 0 | |  | | |
| GSE13507_GSM340677 | 0 | | 0.005124218 | |  | | |
| GSE13507_GSM340679 | 0 | | 0 | |  | | |
| GSE13507_GSM340681 | 0 | | 0 | |  | | |
| GSE13507_GSM340686 | 0 | | 0 | |  | | |
| GSE13507_GSM340687 | 0 | | 0.000997475 | |  | | |
| GSE13507_GSM340693 | 0 | | 0 | |  | | |
| GSE13507_GSM340694 | 0 | | 0 | |  | | |
| GSE13507_GSM340696 | 0 | | 0.037745055 | |  | | |
| GSE13507_GSM340697 | 0 | | 0 | |  | | |
| GSE13507_GSM340700 | 0.00563657 | | 0.006207678 | |  | | |
| GSE13507_GSM340702 | 0 | | 0.001972255 | |  | | |
| GSE13507_GSM340705 | 0 | | 0.012701741 | |  | | |
| GSE13507_GSM340708 | 0 | | 0 | |  | | |
| GSE13507_GSM340722 | 0 | | 0 | |  | | |
| GSE13507_GSM340723 | 0 | | 0.008262584 | |  | | |
| GSE13507_GSM340725 | 0 | | 0.001959879 | |  | | |
| GSE13507_GSM340727 | 0 | | 0.014860043 | |  | | |
| GSE13507_GSM340728 | 0.006137754 | | 0.044803809 | |  | | |
| GSE13507_GSM340730 | 0 | | 0 | |  | | |
| GSE13507_GSM340732 | 0 | | 0.007966183 | |  | | |
| GSE13507_GSM340741 | 0 | | 0.005866937 | |  | | |
| GSE13507_GSM340742 | 0.003036819 | | 0.051926232 | |  | | |
| GSE13507_GSM340744 | 0 | | 0 | |  | | |
| GSE13507_GSM340745 | 0 | | 0 | |  | | |
| GSE13507_GSM340746 | 0 | | 0.018532753 | |  | | |
| GSE13507_GSM340751 | 0 | | 0 | |  | | |
| GSE13507_GSM340752 | 0 | | 0 | |  | | |
| GSE13507_GSM340758 | 0 | | 0 | |  | | |
| GSE13507_GSM340763 | 0 | | 0 | |  | | |
| GSE13507_GSM340769 | 0 | | 0.00349085 | |  | | |
