# Supplementary material for: The Immune Cell Infiltration Patterns and Characterization Score in Bladder Cancer to Identify Prognosis
Source: Front Genet. 2022 Jun 21;13:852708. doi: 10.3389/fgene.2022.852708 (PMC9255635; doi:10.3389/fgene.2022.852708)
Supplement: Supplementary file 4 [file Table7.DOC]

| **Supplementary Table 8:** Enrichment analysis of ICI score (KEGG gene sets). | | | |
| --- | --- | --- | --- |
| Enrichment in phenotype: the high ICI score group | | | |
| NAME | NES | p-value | FDR q-val |
| KEGG_TASTE_TRANSDUCTION | 1.6481768 | 0.022222223 | 0.8732218 |
| KEGG_FATTY_ACID_METABOLISM | 1.5506264 | 0.033898305 | 0.93104136 |
| KEGG_GLYCINE_SERINE_AND_THREONINE_METABOLISM | 1.520006 | 0.030710172 | 0.76811063 |
| KEGG_PENTOSE_AND_GLUCURONATE_INTERCONVERSIONS | 1.5037718 | 0.06839187 | 0.6478636 |
| KEGG_PPAR_SIGNALING_PATHWAY | 1.4752166 | 0.031365313 | 0.62785006 |
| KEGG_DRUG_METABOLISM_CYTOCHROME_P450 | 1.4112062 | 0.077876106 | 0.7617004 |
| KEGG_CARDIAC_MUSCLE_CONTRACTION | 1.3850793 | 0.11363637 | 0.7503405 |
| KEGG_ASCORBATE_AND_ALDARATE_METABOLISM | 1.3788798 | 0.11173184 | 0.6792418 |
| KEGG_PROXIMAL_TUBULE_BICARBONATE_RECLAMATION | 1.3403981 | 0.104779415 | 0.7333873 |
| KEGG_PORPHYRIN_AND_CHLOROPHYLL_METABOLISM | 1.3382336 | 0.15018316 | 0.66639405 |
| KEGG_PEROXISOME | 1.3379828 | 0.13899614 | 0.6063974 |
| KEGG_TYROSINE_METABOLISM | 1.3372258 | 0.091561936 | 0.5575685 |
| KEGG_VALINE_LEUCINE_AND_ISOLEUCINE_DEGRADATION | 1.332811 | 0.15643564 | 0.5269847 |
| KEGG_SELENOAMINO_ACID_METABOLISM | 1.2918527 | 0.16393442 | 0.59484434 |
| KEGG_METABOLISM_OF_XENOBIOTICS_BY_CYTOCHROME_P450 | 1.2828556 | 0.1865942 | 0.5784677 |
| KEGG_RETINOL_METABOLISM | 1.2692173 | 0.1827957 | 0.5732709 |
| KEGG_ALDOSTERONE_REGULATED_SODIUM_REABSORPTION | 1.2465427 | 0.16604477 | 0.5929815 |
| KEGG_GLYCEROPHOSPHOLIPID_METABOLISM | 1.2439104 | 0.12331407 | 0.56660116 |
| KEGG_STEROID_HORMONE_BIOSYNTHESIS | 1.219139 | 0.21272728 | 0.59584105 |
| KEGG_LINOLEIC_ACID_METABOLISM | 1.1764024 | 0.25316456 | 0.6660996 |
| KEGG_TIGHT_JUNCTION | 1.1711702 | 0.2 | 0.64610165 |
| KEGG_ALPHA_LINOLENIC_ACID_METABOLISM | 1.1467726 | 0.2635379 | 0.67690814 |
| KEGG_ABC_TRANSPORTERS | 1.1293912 | 0.3027523 | 0.6868168 |
| KEGG_N_GLYCAN_BIOSYNTHESIS | 1.1142793 | 0.36639675 | 0.6927711 |
| KEGG_GLYCOLYSIS_GLUCONEOGENESIS | 1.0957859 | 0.3296501 | 0.7061008 |
| KEGG_PRIMARY_BILE_ACID_BIOSYNTHESIS | 1.0880252 | 0.36742425 | 0.6952127 |
| KEGG_BUTANOATE_METABOLISM | 1.0840825 | 0.3634538 | 0.6781752 |
| KEGG_CYSTEINE_AND_METHIONINE_METABOLISM | 1.0807046 | 0.375 | 0.66112524 |
| KEGG_ETHER_LIPID_METABOLISM | 1.0246617 | 0.39926064 | 0.75667924 |
| KEGG_BETA_ALANINE_METABOLISM | 1.0144405 | 0.41199225 | 0.75266707 |
| KEGG_ALANINE_ASPARTATE_AND_GLUTAMATE_METABOLISM | 1.0089828 | 0.42805755 | 0.7399299 |
| KEGG_PROPANOATE_METABOLISM | 1.0003017 | 0.45686275 | 0.73515624 |
| KEGG_BIOSYNTHESIS_OF_UNSATURATED_FATTY_ACIDS | 0.9875075 | 0.4880478 | 0.7382526 |
| KEGG_STARCH_AND_SUCROSE_METABOLISM | 0.9811122 | 0.46554935 | 0.7295578 |
| KEGG_MATURITY_ONSET_DIABETES_OF_THE_YOUNG | 0.9481702 | 0.51805055 | 0.7748491 |
| KEGG_GLYCOSYLPHOSPHATIDYLINOSITOL_GPI_ANCHOR_BIOSYNTHESIS | 0.9354395 | 0.5417515 | 0.77937734 |
| KEGG_ARACHIDONIC_ACID_METABOLISM | 0.9337881 | 0.5358423 | 0.7619449 |
| KEGG_PYRUVATE_METABOLISM | 0.9281824 | 0.54296875 | 0.75205314 |
| KEGG_TRYPTOPHAN_METABOLISM | 0.92275435 | 0.571949 | 0.74253845 |
| KEGG_OTHER_GLYCAN_DEGRADATION | 0.8895462 | 0.60816324 | 0.7872961 |
| KEGG_TERPENOID_BACKBONE_BIOSYNTHESIS | 0.88416064 | 0.590535 | 0.77810425 |
| KEGG_NICOTINATE_AND_NICOTINAMIDE_METABOLISM | 0.8714082 | 0.6535581 | 0.78298306 |
| KEGG_INOSITOL_PHOSPHATE_METABOLISM | 0.86411977 | 0.64803314 | 0.77738416 |
| KEGG_TGF_BETA_SIGNALING_PATHWAY | 0.86226505 | 0.6368821 | 0.7631257 |
| KEGG_PURINE_METABOLISM | 0.85525244 | 0.68421054 | 0.75852734 |
| KEGG_GLYCOSAMINOGLYCAN_BIOSYNTHESIS_HEPARAN_SULFATE | 0.8462704 | 0.66467065 | 0.7575654 |
| KEGG_GLYCEROLIPID_METABOLISM | 0.79131156 | 0.8485437 | 0.83624035 |
| KEGG_PHENYLALANINE_METABOLISM | 0.7836243 | 0.8057041 | 0.831932 |
| KEGG_GLYCOSPHINGOLIPID_BIOSYNTHESIS_GANGLIO_SERIES | 0.76283014 | 0.791587 | 0.8476915 |
| KEGG_GLUTATHIONE_METABOLISM | 0.7606255 | 0.790566 | 0.8341737 |
| KEGG_HUNTINGTONS_DISEASE | 0.71935344 | 0.8063241 | 0.8797962 |
| KEGG_NEUROACTIVE_LIGAND_RECEPTOR_INTERACTION | 0.71136296 | 0.93115944 | 0.8740916 |
| KEGG_BASAL_CELL_CARCINOMA | 0.6986372 | 0.8888889 | 0.8742642 |
| KEGG_VASCULAR_SMOOTH_MUSCLE_CONTRACTION | 0.69339794 | 0.8767123 | 0.8644264 |
| KEGG_TYPE_II_DIABETES_MELLITUS | 0.6841741 | 0.947644 | 0.86033666 |
| KEGG_RIBOSOME | 0.4559915 | 0.93465346 | 0.9891679 |
| Enrichment in phenotype: the low ICI score group | | | |
| NAME | NES | p-value | FDR q-val |
| KEGG_CYTOSOLIC_DNA_SENSING_PATHWAY | -1.8962559 | 0.004192872 | 0.29919404 |
| KEGG_RIG_I_LIKE_RECEPTOR_SIGNALING_PATHWAY | -1.715539 | 0.01002004 | 0.6036764 |
| KEGG_PROTEASOME | -1.699994 | 0.029411765 | 0.45415145 |
| KEGG_TOLL_LIKE_RECEPTOR_SIGNALING_PATHWAY | -1.6766762 | 0.02296451 | 0.41126853 |
| KEGG_NOD_LIKE_RECEPTOR_SIGNALING_PATHWAY | -1.6535443 | 0.018828452 | 0.4054588 |
| KEGG_BASAL_TRANSCRIPTION_FACTORS | -1.6094016 | 0.035363458 | 0.47178483 |
| KEGG_GALACTOSE_METABOLISM | -1.580872 | 0.033264033 | 0.49810648 |
| KEGG_EPITHELIAL_CELL_SIGNALING_IN_HELICOBACTER_PYLORI_INFECTION | -1.5796974 | 0.012738854 | 0.43947777 |
| KEGG_PANCREATIC_CANCER | -1.5757637 | 0.019305019 | 0.40162405 |
| KEGG_CHRONIC_MYELOID_LEUKEMIA | -1.5679643 | 0.022132797 | 0.38135713 |
| KEGG_PATHOGENIC_ESCHERICHIA_COLI_INFECTION | -1.5431592 | 0.026262626 | 0.40519455 |
| KEGG_JAK_STAT_SIGNALING_PATHWAY | -1.522999 | 0.0661157 | 0.42566127 |
| KEGG_CELL_CYCLE | -1.5114412 | 0.068 | 0.421217 |
| KEGG_RENAL_CELL_CARCINOMA | -1.507034 | 0.04518664 | 0.40070224 |
| KEGG_MISMATCH_REPAIR | -1.4978023 | 0.06626506 | 0.39783144 |
| KEGG_BLADDER_CANCER | -1.472042 | 0.036960986 | 0.43575785 |
| KEGG_GLIOMA | -1.459525 | 0.02631579 | 0.4415226 |
| KEGG_APOPTOSIS | -1.457982 | 0.07942974 | 0.42189348 |
| KEGG_ERBB_SIGNALING_PATHWAY | -1.4494667 | 0.046413504 | 0.41930607 |
| KEGG_PYRIMIDINE_METABOLISM | -1.4491858 | 0.09467456 | 0.39889383 |
| KEGG_OOCYTE_MEIOSIS | -1.4408424 | 0.08151093 | 0.39769188 |
| KEGG_NUCLEOTIDE_EXCISION_REPAIR | -1.4404687 | 0.12840466 | 0.38053912 |
| KEGG_DNA_REPLICATION | -1.4271195 | 0.12966602 | 0.38970885 |
| KEGG_CYTOKINE_CYTOKINE_RECEPTOR_INTERACTION | -1.4151967 | 0.10147992 | 0.40006772 |
| KEGG_PROGESTERONE_MEDIATED_OOCYTE_MATURATION | -1.4022628 | 0.087128714 | 0.41001385 |
| KEGG_NATURAL_KILLER_CELL_MEDIATED_CYTOTOXICITY | -1.3959166 | 0.12938596 | 0.40695035 |
| KEGG_SMALL_CELL_LUNG_CANCER | -1.393714 | 0.096969694 | 0.396511 |
| KEGG_LEISHMANIA_INFECTION | -1.3818603 | 0.15286624 | 0.4061898 |
| KEGG_P53_SIGNALING_PATHWAY | -1.3604493 | 0.103869654 | 0.4349508 |
| KEGG_FC_GAMMA_R_MEDIATED_PHAGOCYTOSIS | -1.3484442 | 0.124197 | 0.44679862 |
| KEGG_ANTIGEN_PROCESSING_AND_PRESENTATION | -1.3389347 | 0.23236515 | 0.45269275 |
| KEGG_PRION_DISEASES | -1.3161931 | 0.15843621 | 0.4883862 |
| KEGG_SNARE_INTERACTIONS_IN_VESICULAR_TRANSPORT | -1.3083351 | 0.15927419 | 0.4915342 |
| KEGG_ACUTE_MYELOID_LEUKEMIA | -1.3029276 | 0.16075157 | 0.4887 |
| KEGG_T_CELL_RECEPTOR_SIGNALING_PATHWAY | -1.2974261 | 0.20168068 | 0.48678786 |
| KEGG_UBIQUITIN_MEDIATED_PROTEOLYSIS | -1.2907556 | 0.18252428 | 0.4883288 |
| KEGG_COMPLEMENT_AND_COAGULATION_CASCADES | -1.2895083 | 0.1814346 | 0.47756338 |
| KEGG_GRAFT_VERSUS_HOST_DISEASE | -1.2872909 | 0.25571725 | 0.46923757 |
| KEGG_RNA_POLYMERASE | -1.2770157 | 0.21713148 | 0.47937077 |
| KEGG_ECM_RECEPTOR_INTERACTION | -1.2685822 | 0.2275574 | 0.48447302 |
| KEGG_HEMATOPOIETIC_CELL_LINEAGE | -1.2530656 | 0.25213677 | 0.5043578 |
| KEGG_PATHWAYS_IN_CANCER | -1.2427114 | 0.14893617 | 0.51351416 |
| KEGG_SPLICEOSOME | -1.2350943 | 0.30393997 | 0.51702416 |
| KEGG_GNRH_SIGNALING_PATHWAY | -1.2198652 | 0.15869565 | 0.5377627 |
| KEGG_AMINO_SUGAR_AND_NUCLEOTIDE_SUGAR_METABOLISM | -1.2163504 | 0.25833333 | 0.53355217 |
| KEGG_REGULATION_OF_AUTOPHAGY | -1.2126045 | 0.25296444 | 0.5303394 |
| KEGG_GAP_JUNCTION | -1.212144 | 0.23076923 | 0.5199565 |
| KEGG_REGULATION_OF_ACTIN_CYTOSKELETON | -1.2118088 | 0.21132898 | 0.50986195 |
| KEGG_AUTOIMMUNE_THYROID_DISEASE | -1.2086595 | 0.3219616 | 0.50544494 |
| KEGG_CHEMOKINE_SIGNALING_PATHWAY | -1.193289 | 0.26709402 | 0.52590394 |
| KEGG_NEUROTROPHIN_SIGNALING_PATHWAY | -1.1827313 | 0.25462013 | 0.5351197 |
| KEGG_HOMOLOGOUS_RECOMBINATION | -1.1735909 | 0.34516767 | 0.5434963 |
| KEGG_COLORECTAL_CANCER | -1.1728611 | 0.26559356 | 0.5344177 |
| KEGG_FOCAL_ADHESION | -1.144257 | 0.3210634 | 0.58132535 |
| KEGG_AMYOTROPHIC_LATERAL_SCLEROSIS_ALS | -1.139431 | 0.27578476 | 0.5807033 |
| KEGG_ALLOGRAFT_REJECTION | -1.1330886 | 0.3923241 | 0.5826363 |
| KEGG_NON_SMALL_CELL_LUNG_CANCER | -1.1172289 | 0.31013918 | 0.602433 |
| KEGG_ALZHEIMERS_DISEASE | -1.1168498 | 0.29718876 | 0.5928339 |
| KEGG_ADHERENS_JUNCTION | -1.1133115 | 0.3326613 | 0.5903726 |
| KEGG_NOTCH_SIGNALING_PATHWAY | -1.1122456 | 0.32310838 | 0.58259845 |
| KEGG_ARRHYTHMOGENIC_RIGHT_VENTRICULAR_CARDIOMYOPATHY_ARVC | -1.1119187 | 0.31781375 | 0.5735442 |
| KEGG_O_GLYCAN_BIOSYNTHESIS | -1.1055458 | 0.33267716 | 0.5762398 |
| KEGG_ENDOMETRIAL_CANCER | -1.1000165 | 0.36895162 | 0.57776135 |
| KEGG_PRIMARY_IMMUNODEFICIENCY | -1.0964068 | 0.41525424 | 0.57549334 |
| KEGG_LONG_TERM_POTENTIATION | -1.0906585 | 0.36023623 | 0.5770952 |
| KEGG_MELANOGENESIS | -1.0638565 | 0.34567901 | 0.61664563 |
| KEGG_B_CELL_RECEPTOR_SIGNALING_PATHWAY | -1.0561405 | 0.43404254 | 0.6221926 |
| KEGG_VIRAL_MYOCARDITIS | -1.0558531 | 0.42105263 | 0.613489 |
| KEGG_THYROID_CANCER | -1.0431385 | 0.40733197 | 0.62826985 |
| KEGG_GLYCOSAMINOGLYCAN_BIOSYNTHESIS_CHONDROITIN_SULFATE | -1.0246834 | 0.43407708 | 0.65422416 |
| KEGG_RNA_DEGRADATION | -1.0200335 | 0.45366797 | 0.6540419 |
| KEGG_LYSINE_DEGRADATION | -1.0120625 | 0.45708582 | 0.6591452 |
| KEGG_WNT_SIGNALING_PATHWAY | -1.0118542 | 0.42735043 | 0.65048987 |
| KEGG_SPHINGOLIPID_METABOLISM | -1.0111855 | 0.428 | 0.6428339 |
| KEGG_TYPE_I_DIABETES_MELLITUS | -1.0034692 | 0.48590022 | 0.64855176 |
| KEGG_MTOR_SIGNALING_PATHWAY | -1.0032245 | 0.4651163 | 0.64046735 |
| KEGG_GLYCOSPHINGOLIPID_BIOSYNTHESIS_LACTO_AND_NEOLACTO_SERIES | -0.99948364 | 0.4681275 | 0.63882244 |
| KEGG_DORSO_VENTRAL_AXIS_FORMATION | -0.9977085 | 0.45208332 | 0.63407004 |
| KEGG_ARGININE_AND_PROLINE_METABOLISM | -0.9932953 | 0.45755693 | 0.63341767 |
| KEGG_FRUCTOSE_AND_MANNOSE_METABOLISM | -0.99283636 | 0.45147678 | 0.62643176 |
| KEGG_ENDOCYTOSIS | -0.991906 | 0.47474748 | 0.6204601 |
| KEGG_STEROID_BIOSYNTHESIS | -0.9848676 | 0.48902196 | 0.624292 |
| KEGG_CITRATE_CYCLE_TCA_CYCLE | -0.97950226 | 0.47553816 | 0.62616754 |
| KEGG_PROSTATE_CANCER | -0.965175 | 0.5231388 | 0.6435766 |
| KEGG_SYSTEMIC_LUPUS_ERYTHEMATOSUS | -0.96104914 | 0.5351812 | 0.64320445 |
| KEGG_AXON_GUIDANCE | -0.955504 | 0.51304346 | 0.6453341 |
| KEGG_BASE_EXCISION_REPAIR | -0.95277244 | 0.5335968 | 0.64269924 |
| KEGG_PANTOTHENATE_AND_COA_BIOSYNTHESIS | -0.9498931 | 0.53961456 | 0.64057386 |
| KEGG_PHOSPHATIDYLINOSITOL_SIGNALING_SYSTEM | -0.9483807 | 0.5575397 | 0.6357876 |
| KEGG_NITROGEN_METABOLISM | -0.93586993 | 0.52109706 | 0.64901274 |
| KEGG_FC_EPSILON_RI_SIGNALING_PATHWAY | -0.93218803 | 0.53205127 | 0.6479815 |
| KEGG_PROTEIN_EXPORT | -0.92806983 | 0.5551102 | 0.6480395 |
| KEGG_GLYCOSAMINOGLYCAN_DEGRADATION | -0.9235772 | 0.5614754 | 0.64826053 |
| KEGG_MAPK_SIGNALING_PATHWAY | -0.9123508 | 0.5877193 | 0.6598496 |
| KEGG_INTESTINAL_IMMUNE_NETWORK_FOR_IGA_PRODUCTION | -0.91033965 | 0.55932206 | 0.656546 |
| KEGG_LONG_TERM_DEPRESSION | -0.892307 | 0.62173915 | 0.6805366 |
| KEGG_GLYOXYLATE_AND_DICARBOXYLATE_METABOLISM | -0.8870253 | 0.59770113 | 0.682145 |
| KEGG_MELANOMA | -0.8808309 | 0.6587473 | 0.68547314 |
| KEGG_HISTIDINE_METABOLISM | -0.8791338 | 0.6764045 | 0.6810081 |
| KEGG_CALCIUM_SIGNALING_PATHWAY | -0.8542187 | 0.6509636 | 0.7155515 |
| KEGG_ASTHMA | -0.831018 | 0.6539278 | 0.7476781 |
| KEGG_DRUG_METABOLISM_OTHER_ENZYMES | -0.8301259 | 0.68619245 | 0.7420022 |
| KEGG_VEGF_SIGNALING_PATHWAY | -0.80396694 | 0.777042 | 0.7800263 |
| KEGG_INSULIN_SIGNALING_PATHWAY | -0.8019974 | 0.78361344 | 0.77598745 |
| KEGG_ONE_CARBON_POOL_BY_FOLATE | -0.7975457 | 0.7074148 | 0.77596194 |
| KEGG_LEUKOCYTE_TRANSENDOTHELIAL_MIGRATION | -0.79414606 | 0.74626863 | 0.77402765 |
| KEGG_DILATED_CARDIOMYOPATHY | -0.78917116 | 0.73221755 | 0.7752106 |
| KEGG_PENTOSE_PHOSPHATE_PATHWAY | -0.77835673 | 0.75259876 | 0.7852629 |
| KEGG_OLFACTORY_TRANSDUCTION | -0.7717103 | 0.5917969 | 0.7883361 |
| KEGG_HEDGEHOG_SIGNALING_PATHWAY | -0.764218 | 0.8065217 | 0.79283124 |
| KEGG_AMINOACYL_TRNA_BIOSYNTHESIS | -0.74867475 | 0.7207031 | 0.80879587 |
| KEGG_LYSOSOME | -0.7400788 | 0.7576375 | 0.81412923 |
| KEGG_CELL_ADHESION_MOLECULES_CAMS | -0.72707057 | 0.7819383 | 0.8257212 |
| KEGG_HYPERTROPHIC_CARDIOMYOPATHY_HCM | -0.7168467 | 0.8375527 | 0.83301 |
| KEGG_ADIPOCYTOKINE_SIGNALING_PATHWAY | -0.71624446 | 0.9 | 0.8267609 |
| KEGG_VASOPRESSIN_REGULATED_WATER_REABSORPTION | -0.6783392 | 0.86382115 | 0.86979717 |
| KEGG_PARKINSONS_DISEASE | -0.6713922 | 0.77959186 | 0.8709176 |
| KEGG_VIBRIO_CHOLERAE_INFECTION | -0.6317754 | 0.9284211 | 0.9067251 |
| KEGG_RENIN_ANGIOTENSIN_SYSTEM | -0.54989165 | 0.9676724 | 0.96040314 |
| KEGG_OXIDATIVE_PHOSPHORYLATION | -0.54341996 | 0.9117043 | 0.9556781 |
