# Supplementary material for: The Immune Cell Infiltration Patterns and Characterization Score in Bladder Cancer to Identify Prognosis
Source: Front Genet. 2022 Jun 21;13:852708. doi: 10.3389/fgene.2022.852708 (PMC9255635; doi:10.3389/fgene.2022.852708)
Supplement: Supplementary file 5 [file Table3.DOC]

**Supplementary Table 4:** Differentially expressed genes (DEGs) between ICI cluster A and ICI cluster B.

| MMP9 | SERPINB2 | KRT6B | DSC3 |
| --- | --- | --- | --- |
| SBSN | DSG3 | DSP | SNCG |
| PTHLH | GJB2 | PKP1 | KRT4 |
| LCE3D | SPP1 | CALML5 | MMP1 |
| MMP12 | FGFBP1 | TNNT1 | SERPINB3 |
| SPRR2E | SPRR2A | CCL19 | SERPINB13 |
| TGM1 | SPRR2G | LAMC2 | MMP13 |
| SPRR1B | LGALS7 | KRT6C | DHRS2 |
| BARX2 | KRT16 | KRT1 |  |
| DSC2 | PI3 | UBD |  |
