# Supplementary material for: The Immune Cell Infiltration Patterns and Characterization Score in Bladder Cancer to Identify Prognosis
Source: Front Genet. 2022 Jun 21;13:852708. doi: 10.3389/fgene.2022.852708 (PMC9255635; doi:10.3389/fgene.2022.852708)
Supplement: Supplementary file 6 [file Table1.DOC]

**Supplementary Table 2:** Results of immune score and stromal score of 569 BLCA patients.

| **ID** | **StromalScore** | **ImmuneScore** | **ESTIMATEScore** |
| --- | --- | --- | --- |
| TCGA_TCGA-ZF-A9R7 | -1048.983799 | 1841.799321 | 792.8155212 |
| TCGA_TCGA-FD-A3SO | 230.1095297 | 866.4315676 | 1096.541097 |
| TCGA_TCGA-E7-A97P | -44.82876309 | 1568.461157 | 1523.632394 |
| TCGA_TCGA-GU-A767 | -435.4242881 | -230.6899826 | -666.1142708 |
| TCGA_TCGA-4Z-AA81 | -1364.951506 | 659.4255098 | -705.5259961 |
| TCGA_TCGA-E7-A85H | -645.8951876 | 447.402159 | -198.4930287 |
| TCGA_TCGA-G2-AA3C | -73.59416478 | 1901.603459 | 1828.009294 |
| TCGA_TCGA-E7-A8O8 | -1518.58502 | -348.168777 | -1866.753797 |
| TCGA_TCGA-XF-A9SM | 490.9290648 | 2286.323573 | 2777.252638 |
| TCGA_TCGA-DK-A3IN | 322.0038481 | 857.5142275 | 1179.518076 |
| TCGA_TCGA-2F-A9KR | -1560.570177 | -338.7931131 | -1899.36329 |
| TCGA_TCGA-E5-A4U1 | -2102.713634 | -996.6073901 | -3099.321024 |
| TCGA_TCGA-E7-A3Y1 | -1329.170724 | -210.3421455 | -1539.512869 |
| TCGA_TCGA-FD-A6TC | 160.2330805 | 0.991094514 | 161.224175 |
| TCGA_TCGA-FD-A5BV | -1135.714347 | -605.8986028 | -1741.612949 |
| TCGA_TCGA-DK-A2I4 | 1025.959849 | 2585.511255 | 3611.471104 |
| TCGA_TCGA-UY-A78M | -412.8394093 | -343.9918975 | -756.8313068 |
| TCGA_TCGA-4Z-AA7R | -1039.280706 | -781.2136841 | -1820.494391 |
| TCGA_TCGA-HQ-A5NE | -577.3805313 | 209.8334786 | -367.5470527 |
| TCGA_TCGA-XF-AAMT | 682.2361932 | 872.7551155 | 1554.991309 |
| TCGA_TCGA-XF-A9SU | 329.6590704 | 801.7547223 | 1131.413793 |
| TCGA_TCGA-CF-A47S | -1531.72763 | 144.6949237 | -1387.032706 |
| TCGA_TCGA-FD-A6TK | 742.602785 | 1422.84202 | 2165.444805 |
| TCGA_TCGA-E7-A5KF | -2454.54212 | -611.3292426 | -3065.871363 |
| TCGA_TCGA-UY-A78L | -354.927863 | 102.0878741 | -252.8399889 |
| TCGA_TCGA-S5-A6DX | 260.9004422 | 1757.476771 | 2018.377213 |
| TCGA_TCGA-XF-A8HI | -1584.028381 | -82.34128454 | -1666.369666 |
| TCGA_TCGA-XF-A9T5 | -29.22262809 | 1979.506555 | 1950.283927 |
| TCGA_TCGA-XF-A9SX | 832.2997123 | 1740.005321 | 2572.305033 |
| TCGA_TCGA-G2-A2EK | -1006.02003 | 189.7543496 | -816.2656807 |
| TCGA_TCGA-ZF-A9RE | -2122.510721 | -439.1678644 | -2561.678586 |
| TCGA_TCGA-E7-A7DV | 456.4256509 | 1962.213771 | 2418.639422 |
| TCGA_TCGA-XF-AAN1 | -1537.588781 | -252.5727766 | -1790.161558 |
| TCGA_TCGA-DK-AA6P | -1674.372323 | -892.0948664 | -2566.467189 |
| TCGA_TCGA-CF-A47Y | -1705.497763 | -490.1266905 | -2195.624453 |
| TCGA_TCGA-CF-A3MI | -1093.016127 | 228.0521702 | -864.9639567 |
| TCGA_TCGA-G2-A2EL | -2122.428541 | -1699.828619 | -3822.25716 |
| TCGA_TCGA-XF-AAMZ | -1077.158476 | 154.8737007 | -922.2847753 |
| TCGA_TCGA-DK-AA6L | -1042.863922 | 657.3008878 | -385.5630343 |
| TCGA_TCGA-XF-A9T4 | -830.7029109 | 487.2232804 | -343.4796304 |
| TCGA_TCGA-K4-A6MB | -553.7492527 | -327.3249092 | -881.0741619 |
| TCGA_TCGA-UY-A8OB | -1386.284277 | 449.608456 | -936.6758213 |
| TCGA_TCGA-DK-A1A5 | 177.6887183 | 240.9859002 | 418.6746185 |
| TCGA_TCGA-2F-A9KQ | -1275.936628 | -683.7927293 | -1959.729357 |
| TCGA_TCGA-SY-A9G0 | 958.7767209 | 510.6453642 | 1469.422085 |
| TCGA_TCGA-GV-A40G | -1871.184518 | -777.8235935 | -2649.008111 |
| TCGA_TCGA-FD-A5BX | 697.3221127 | 1709.617448 | 2406.93956 |
| TCGA_TCGA-BL-A13J | -130.8452981 | -160.7886429 | -291.633941 |
| TCGA_TCGA-ZF-AA54 | 711.3571072 | 1586.906347 | 2298.263454 |
| TCGA_TCGA-GU-A766 | 116.9071611 | 2279.846261 | 2396.753422 |
| TCGA_TCGA-LT-A8JT | -1529.897844 | 56.27742014 | -1473.620423 |
| TCGA_TCGA-FJ-A3Z9 | -2054.919256 | -820.4074214 | -2875.326678 |
| TCGA_TCGA-DK-A1AC | -121.7782131 | 780.4497399 | 658.6715268 |
| TCGA_TCGA-C4-A0F1 | -481.8129434 | 188.0644778 | -293.7484656 |
| TCGA_TCGA-G2-A2ES | -163.2721994 | 712.808127 | 549.5359276 |
| TCGA_TCGA-XF-AAN4 | 1482.144149 | 1860.380038 | 3342.524187 |
| TCGA_TCGA-4Z-AA83 | -888.1639313 | 342.5590804 | -545.6048509 |
| TCGA_TCGA-4Z-AA7N | 676.4117131 | 2396.622187 | 3073.0339 |
| TCGA_TCGA-HQ-A2OE | -1821.041061 | -530.6602245 | -2351.701286 |
| TCGA_TCGA-UY-A8OC | 235.7483414 | 192.9693651 | 428.7177064 |
| TCGA_TCGA-GV-A3JV | -294.2045705 | 448.031286 | 153.8267155 |
| TCGA_TCGA-XF-A9T8 | 223.6726233 | 1829.970317 | 2053.64294 |
| TCGA_TCGA-KQ-A41S | 234.3384861 | 162.1140009 | 396.452487 |
| TCGA_TCGA-CU-A3YL | -1071.082261 | -243.966664 | -1315.048925 |
| TCGA_TCGA-E7-A7XN | -1009.058339 | 1759.364004 | 750.3056657 |
| TCGA_TCGA-DK-A2HX | 810.6673507 | 131.9496602 | 942.6170109 |
| TCGA_TCGA-CF-A8HY | -1667.459781 | -186.3272273 | -1853.787009 |
| TCGA_TCGA-4Z-AA7W | -181.4811064 | 2359.150897 | 2177.669791 |
| TCGA_TCGA-FD-A43U | 904.4921734 | 2159.386871 | 3063.879045 |
| TCGA_TCGA-BL-A0C8 | -753.8803175 | -533.7982407 | -1287.678558 |
| TCGA_TCGA-BT-A0S7 | -570.6403304 | -197.0001532 | -767.6404836 |
| TCGA_TCGA-ZF-A9R3 | -1261.477258 | 121.5106888 | -1139.966569 |
| TCGA_TCGA-4Z-AA89 | -2239.675467 | -170.1788488 | -2409.854316 |
| TCGA_TCGA-GU-A42P | -1958.583605 | -1086.178233 | -3044.761837 |
| TCGA_TCGA-FD-A3NA | -458.8460197 | 532.766117 | 73.92009725 |
| TCGA_TCGA-E7-A5KE | -2234.992818 | -1074.479415 | -3309.472233 |
| TCGA_TCGA-ZF-AA4V | -583.2681579 | 699.917682 | 116.6495241 |
| TCGA_TCGA-ZF-AA53 | 506.150422 | 1546.397169 | 2052.547591 |
| TCGA_TCGA-ZF-A9RM | -2571.062629 | -955.6715157 | -3526.734144 |
| TCGA_TCGA-BL-A13I | 780.9193334 | 1975.859465 | 2756.778798 |
| TCGA_TCGA-CU-A72E | -278.5256455 | 154.5362202 | -123.9894253 |
| TCGA_TCGA-CF-A47W | -1708.164916 | -472.6836427 | -2180.848559 |
| TCGA_TCGA-GC-A3I6 | -643.814799 | 659.5420144 | 15.72721546 |
| TCGA_TCGA-FD-A6TA | 392.0230165 | 1106.387017 | 1498.410033 |
| TCGA_TCGA-CF-A47X | -1847.200329 | -719.9752773 | -2567.175606 |
| TCGA_TCGA-ZF-AA51 | -50.43134887 | 1300.336568 | 1249.905219 |
| TCGA_TCGA-FD-A62O | -967.5676174 | -531.8826689 | -1499.450286 |
| TCGA_TCGA-BL-A3JM | -693.8884109 | -330.7473756 | -1024.635786 |
| TCGA_TCGA-FJ-A3ZF | -1915.592772 | -1136.946948 | -3052.53972 |
| TCGA_TCGA-CU-A0YO | -439.9266164 | 712.5730658 | 272.6464494 |
| TCGA_TCGA-CF-A5UA | -1593.195394 | -359.5649277 | -1952.760322 |
| TCGA_TCGA-K4-AAQO | 332.1969365 | 646.9161854 | 979.1131219 |
| TCGA_TCGA-FD-A3SM | 25.62425839 | -173.4982629 | -147.8740045 |
| TCGA_TCGA-BL-A5ZZ | 785.1277874 | 1069.247422 | 1854.375209 |
| TCGA_TCGA-FD-A62N | 1208.29357 | 2874.161535 | 4082.455105 |
| TCGA_TCGA-BT-A20J | -464.5910341 | 1614.900387 | 1150.309353 |
| TCGA_TCGA-FT-A61P | 517.6259091 | 1039.807912 | 1557.433821 |
| TCGA_TCGA-DK-AA77 | -1481.062732 | 354.4577779 | -1126.604954 |
| TCGA_TCGA-ZF-AA5N | -1628.71633 | -279.0509372 | -1907.767267 |
| TCGA_TCGA-DK-AA6U | -1931.863146 | -526.0135097 | -2457.876655 |
| TCGA_TCGA-BT-A20O | 892.2479331 | 2910.774779 | 3803.022712 |
| TCGA_TCGA-GD-A3OS | -564.8746659 | -195.2330521 | -760.1077181 |
| TCGA_TCGA-XF-A9SZ | 483.4777777 | 706.5709992 | 1190.048777 |
| TCGA_TCGA-4Z-AA82 | -324.8515836 | 162.6187408 | -162.2328428 |
| TCGA_TCGA-DK-AA76 | -1863.291279 | -1141.541658 | -3004.832937 |
| TCGA_TCGA-DK-A3IL | -880.7133622 | -407.3835434 | -1288.096906 |
| TCGA_TCGA-E7-A678 | -1963.173943 | -97.62632013 | -2060.800263 |
| TCGA_TCGA-ZF-A9R1 | -1315.833435 | -276.8090072 | -1592.642442 |
| TCGA_TCGA-XF-A9SH | -962.8723644 | 17.04137232 | -945.8309921 |
| TCGA_TCGA-FD-A5BU | 327.3684876 | 1145.930799 | 1473.299286 |
| TCGA_TCGA-C4-A0F7 | -1052.430103 | -559.1847562 | -1611.614859 |
| TCGA_TCGA-C4-A0F0 | -293.1117084 | 701.1234743 | 408.0117659 |
| TCGA_TCGA-FD-A3N5 | -1025.583278 | 216.4738553 | -809.1094223 |
| TCGA_TCGA-ZF-A9R5 | -1764.951138 | -194.3387021 | -1959.28984 |
| TCGA_TCGA-CU-A3KJ | -1158.514881 | 302.320764 | -856.1941175 |
| TCGA_TCGA-DK-AA6S | 637.6041258 | 982.2486838 | 1619.85281 |
| TCGA_TCGA-BT-A42E | -615.4661756 | 1559.653232 | 944.1870559 |
| TCGA_TCGA-FD-A43X | -1848.160951 | -614.7476958 | -2462.908647 |
| TCGA_TCGA-ZF-AA4R | 18.55951279 | -282.1502786 | -263.5907658 |
| TCGA_TCGA-BT-A20T | -169.419044 | 623.4829628 | 454.0639188 |
| TCGA_TCGA-E7-A7DU | -1591.61809 | -275.5855376 | -1867.203628 |
| TCGA_TCGA-DK-A3WY | 725.3776499 | 2865.709087 | 3591.086737 |
| TCGA_TCGA-G2-A2EF | -771.9150267 | 1469.183902 | 697.2688749 |
| TCGA_TCGA-BT-A20V | -1767.85758 | 282.7754324 | -1485.082148 |
| TCGA_TCGA-K4-A3WV | -1462.824222 | -948.8545324 | -2411.678754 |
| TCGA_TCGA-GU-AATQ | -1193.048785 | 17.71185151 | -1175.336934 |
| TCGA_TCGA-CF-A3MF | -1360.421653 | -388.6947092 | -1749.116363 |
| TCGA_TCGA-CF-A47T | -1354.570714 | -581.0338887 | -1935.604603 |
| TCGA_TCGA-YC-A8S6 | 489.3231713 | 1136.37398 | 1625.697152 |
| TCGA_TCGA-DK-A3IS | -1883.86031 | -296.9132064 | -2180.773516 |
| TCGA_TCGA-XF-A9SJ | 96.25772988 | 1266.064365 | 1362.322095 |
| TCGA_TCGA-DK-AA6T | -220.3527064 | 1998.048871 | 1777.696165 |
| TCGA_TCGA-BT-A20N | -1124.665163 | -792.2040003 | -1916.869163 |
| TCGA_TCGA-XF-AAMW | -581.4423607 | 659.6040607 | 78.16169992 |
| TCGA_TCGA-BT-A3PJ | -740.6091874 | 876.9479798 | 136.3387924 |
| TCGA_TCGA-GC-A3OO | -162.2703423 | 247.8367505 | 85.56640823 |
| TCGA_TCGA-E7-A6ME | -1703.436912 | 58.21311804 | -1645.223794 |
| TCGA_TCGA-XF-AAN5 | -676.8339189 | 1270.842981 | 594.0090622 |
| TCGA_TCGA-FD-A3SN | -419.2405006 | 159.8836961 | -259.3568045 |
| TCGA_TCGA-ZF-A9R2 | -2569.944887 | -863.1044325 | -3433.049319 |
| TCGA_TCGA-ZF-AA4T | -1869.479273 | -495.7257418 | -2365.205015 |
| TCGA_TCGA-E7-A4IJ | -1435.600727 | 363.3021387 | -1072.298588 |
| TCGA_TCGA-FD-A6TE | -2013.00866 | -219.7766786 | -2232.785339 |
| TCGA_TCGA-H4-A2HO | -833.5710666 | 395.765703 | -437.8053637 |
| TCGA_TCGA-FD-A6TB | 323.9075168 | 1765.586122 | 2089.493639 |
| TCGA_TCGA-XF-AAN3 | 122.2243093 | 591.3353379 | 713.5596472 |
| TCGA_TCGA-XF-AAMH | -957.8232482 | -699.9028766 | -1657.726125 |
| TCGA_TCGA-FD-A3B8 | 1479.013417 | 2509.807323 | 3988.82074 |
| TCGA_TCGA-XF-A9SY | 420.7937853 | 1907.918925 | 2328.71271 |
| TCGA_TCGA-CU-A0YN | -243.4492627 | 746.2860565 | 502.8367938 |
| TCGA_TCGA-FD-A5BZ | 865.2526102 | 11.42040117 | 876.6730113 |
| TCGA_TCGA-BT-A20P | -1546.320959 | -507.8540694 | -2054.175029 |
| TCGA_TCGA-XF-A8HD | -380.0179537 | 1265.626499 | 885.6085451 |
| TCGA_TCGA-GV-A3JX | -1370.55813 | 456.9417155 | -913.616415 |
| TCGA_TCGA-H4-A2HQ | -1793.863689 | -461.4586515 | -2255.32234 |
| TCGA_TCGA-XF-A9SP | 801.7090685 | 675.1541061 | 1476.863175 |
| TCGA_TCGA-DK-A3IK | -511.1765674 | -156.4613686 | -667.6379361 |
| TCGA_TCGA-C4-A0F6 | -497.4179404 | -20.48990947 | -517.9078499 |
| TCGA_TCGA-CF-A7I0 | -550.9386997 | -48.01747716 | -598.9561768 |
| TCGA_TCGA-FD-A3B6 | -327.209944 | 1546.339845 | 1219.129901 |
| TCGA_TCGA-GU-A764 | 142.4342897 | 1279.550219 | 1421.984509 |
| TCGA_TCGA-GU-A42R | -1664.545449 | -743.2623471 | -2407.807796 |
| TCGA_TCGA-DK-A6B6 | -1515.774821 | 634.2195341 | -881.5552865 |
| TCGA_TCGA-R3-A69X | 315.6446625 | 1086.307078 | 1401.95174 |
| TCGA_TCGA-5N-A9KM | -204.5834616 | 740.0528485 | 535.4693869 |
| TCGA_TCGA-FD-A6TD | 130.0911523 | 1457.349603 | 1587.440755 |
| TCGA_TCGA-4Z-AA7M | -1158.347237 | -270.9269449 | -1429.274182 |
| TCGA_TCGA-ZF-AA56 | -314.2191984 | 848.4430569 | 534.2238586 |
| TCGA_TCGA-XF-A8HH | 95.26688181 | 165.9941108 | 261.2609926 |
| TCGA_TCGA-C4-A0EZ | -1413.316227 | -1233.322667 | -2646.638893 |
| TCGA_TCGA-XF-AAML | -2040.545303 | 108.2904263 | -1932.254877 |
| TCGA_TCGA-ZF-AA58 | 665.401598 | 2228.14188 | 2893.543478 |
| TCGA_TCGA-UY-A9PA | -1690.688342 | 665.8014019 | -1024.88694 |
| TCGA_TCGA-G2-AA3F | -1190.252512 | -557.2689017 | -1747.521413 |
| TCGA_TCGA-FJ-A871 | -943.1986164 | -604.0285396 | -1547.227156 |
| TCGA_TCGA-ZF-A9RN | -1233.77137 | 866.9229774 | -366.8483928 |
| TCGA_TCGA-GC-A3RB | -1398.754469 | -302.1683437 | -1700.922813 |
| TCGA_TCGA-UY-A9PF | -536.8511599 | 328.8117871 | -208.0393727 |
| TCGA_TCGA-YC-A89H | -745.8205438 | 209.2431395 | -536.5774044 |
| TCGA_TCGA-XF-AAMQ | -776.3132622 | 958.9272441 | 182.6139819 |
| TCGA_TCGA-FD-A3B4 | -13.59988004 | 1167.837915 | 1154.238035 |
| TCGA_TCGA-DK-A1AB | 728.7473492 | 814.100121 | 1542.84747 |
| TCGA_TCGA-BT-A20X | -361.7495874 | 308.6430173 | -53.10657009 |
| TCGA_TCGA-GV-A3JW | -1593.352893 | -1101.35365 | -2694.706543 |
| TCGA_TCGA-UY-A8OD | 439.510086 | 814.4165849 | 1253.926671 |
| TCGA_TCGA-2F-A9KT | -1043.102675 | -209.1159167 | -1252.218592 |
| TCGA_TCGA-ZF-AA5H | -329.9375505 | 1115.891045 | 785.953494 |
| TCGA_TCGA-XF-A9T2 | -251.5076003 | -218.2798799 | -469.7874802 |
| TCGA_TCGA-DK-A3WX | -34.46597029 | 1253.128844 | 1218.662873 |
| TCGA_TCGA-YF-AA3L | -1369.871368 | -235.9787087 | -1605.850077 |
| TCGA_TCGA-BT-A2LA | -1525.538799 | -1542.413191 | -3067.95199 |
| TCGA_TCGA-4Z-AA87 | -898.4564394 | 553.9052489 | -344.5511905 |
| TCGA_TCGA-BT-A2LB | 80.94026723 | 863.9342133 | 944.8744805 |
| TCGA_TCGA-XF-A8HB | -1986.831338 | -522.9543149 | -2509.785653 |
| TCGA_TCGA-DK-A2I6 | -1052.275652 | -344.7801215 | -1397.055774 |
| TCGA_TCGA-ZF-AA4X | -2512.266832 | -482.7609211 | -2995.027753 |
| TCGA_TCGA-E7-A7PW | -2003.981606 | -264.9668371 | -2268.948443 |
| TCGA_TCGA-4Z-AA7S | -1450.264248 | -724.2678229 | -2174.532071 |
| TCGA_TCGA-CF-A3MH | -2022.404488 | -244.2990955 | -2266.703583 |
| TCGA_TCGA-DK-A6B2 | 471.7248161 | 1117.038867 | 1588.763683 |
| TCGA_TCGA-4Z-AA86 | 476.6461755 | 1667.673427 | 2144.319603 |
| TCGA_TCGA-5N-A9KI | -149.5446064 | 584.4894319 | 434.9448255 |
| TCGA_TCGA-XF-A9SL | 977.5868762 | 1848.14207 | 2825.728946 |
| TCGA_TCGA-XF-AAN2 | -1016.585131 | 781.3397891 | -235.245342 |
| TCGA_TCGA-CF-A9FL | -461.0547264 | -107.9049325 | -568.9596589 |
| TCGA_TCGA-CF-A9FM | -1556.471429 | 100.1180307 | -1456.353399 |
| TCGA_TCGA-ZF-A9RD | -328.7116738 | 1686.99188 | 1358.280206 |
| TCGA_TCGA-G2-AA3D | -2170.668238 | -466.2206986 | -2636.888937 |
| TCGA_TCGA-DK-A2I2 | 103.5345585 | 1437.228648 | 1540.763206 |
| TCGA_TCGA-FD-A62S | 1049.70819 | 1249.435106 | 2299.143296 |
| TCGA_TCGA-MV-A51V | -1319.927302 | -679.7871703 | -1999.714472 |
| TCGA_TCGA-E5-A2PC | -1625.352871 | 313.7019462 | -1311.650924 |
| TCGA_TCGA-DK-A3X2 | -1903.493469 | -1038.46247 | -2941.955939 |
| TCGA_TCGA-GD-A76B | -826.9146378 | 1227.80888 | 400.8942423 |
| TCGA_TCGA-GC-A6I1 | -295.6557744 | 2227.647312 | 1931.991538 |
| TCGA_TCGA-XF-A9SK | 574.4387997 | 1087.609403 | 1662.048203 |
| TCGA_TCGA-DK-A1AD | -437.5510384 | 177.3535416 | -260.1974968 |
| TCGA_TCGA-K4-A5RI | -690.3510563 | 79.34277636 | -611.0082799 |
| TCGA_TCGA-FD-A3SQ | 639.3275932 | 721.021493 | 1360.349086 |
| TCGA_TCGA-GV-A3QF | -1742.779767 | -560.4202137 | -2303.199981 |
| TCGA_TCGA-G2-A3VY | -2115.638776 | -1092.132058 | -3207.770835 |
| TCGA_TCGA-XF-AAN8 | 2018.253629 | 2056.142956 | 4074.396586 |
| TCGA_TCGA-CU-A3QU | -2086.324846 | -485.2544333 | -2571.57928 |
| TCGA_TCGA-DK-A6AW | -1167.078634 | -424.2829749 | -1591.361609 |
| TCGA_TCGA-GC-A3BM | -1586.501063 | -154.0149208 | -1740.515984 |
| TCGA_TCGA-GV-A6ZA | -1417.085176 | 234.2418622 | -1182.843313 |
| TCGA_TCGA-G2-A2EJ | -1393.339936 | 267.3494689 | -1125.990467 |
| TCGA_TCGA-FD-A43N | -161.6224427 | 217.5549945 | 55.93255179 |
| TCGA_TCGA-XF-AAMJ | 1059.57048 | 458.3651971 | 1517.935677 |
| TCGA_TCGA-GU-A763 | -2056.655777 | -149.7629178 | -2206.418694 |
| TCGA_TCGA-G2-A2EC | 236.415006 | 791.6523805 | 1028.067387 |
| TCGA_TCGA-FD-A3B7 | 1131.179723 | 1952.027 | 3083.206723 |
| TCGA_TCGA-FD-A5C0 | -555.049657 | 82.07163353 | -472.9780235 |
| TCGA_TCGA-FD-A5BS | 1548.691961 | 3071.198437 | 4619.890398 |
| TCGA_TCGA-BT-A20W | -715.4583112 | 404.9724506 | -310.4858606 |
| TCGA_TCGA-UY-A78O | -971.807882 | -173.7919054 | -1145.599787 |
| TCGA_TCGA-FD-A3B5 | -888.2131672 | -384.8602756 | -1273.073443 |
| TCGA_TCGA-BT-A42C | -1822.449257 | -554.8096053 | -2377.258863 |
| TCGA_TCGA-CF-A8HX | -1685.01953 | -605.491266 | -2290.510796 |
| TCGA_TCGA-PQ-A6FI | -1307.859573 | 311.3250323 | -996.5345409 |
| TCGA_TCGA-FJ-A3Z7 | 54.04554326 | 240.0369046 | 294.0824479 |
| TCGA_TCGA-XF-A8HC | -2099.55227 | -1081.641 | -3181.193269 |
| TCGA_TCGA-ZF-AA4W | -1401.559515 | -140.8532797 | -1542.412795 |
| TCGA_TCGA-LC-A66R | -413.0023834 | 1353.485246 | 940.4828623 |
| TCGA_TCGA-E7-A8O7 | -1367.013671 | 486.5837389 | -880.4299322 |
| TCGA_TCGA-UY-A9PB | 586.3454984 | 1761.766698 | 2348.112197 |
| TCGA_TCGA-FD-A6TI | -794.6109327 | -145.7992853 | -940.4102181 |
| TCGA_TCGA-G2-AA3B | -1811.283955 | 171.1350486 | -1640.148907 |
| TCGA_TCGA-K4-A54R | 99.65240196 | 1208.055201 | 1307.707603 |
| TCGA_TCGA-DK-A1AA | -1483.27956 | -50.49044572 | -1533.770005 |
| TCGA_TCGA-CU-A5W6 | -1055.77619 | 21.74549913 | -1034.030691 |
| TCGA_TCGA-DK-AA6R | -1008.765395 | 44.28561805 | -964.4797765 |
| TCGA_TCGA-BT-A3PH | -1087.336182 | -494.6601616 | -1581.996344 |
| TCGA_TCGA-G2-A3IB | -1518.107857 | -335.459105 | -1853.566962 |
| TCGA_TCGA-2F-A9KP | -1704.560279 | -797.2816632 | -2501.841942 |
| TCGA_TCGA-DK-A3IT | -192.840651 | 369.3622672 | 176.5216162 |
| TCGA_TCGA-FD-A43P | -400.7179085 | 1765.329208 | 1364.6113 |
| TCGA_TCGA-DK-A3IV | -1594.047025 | 1053.884833 | -540.1621918 |
| TCGA_TCGA-2F-A9KW | -141.3288279 | 212.0030243 | 70.6741964 |
| TCGA_TCGA-GU-A762 | 302.8617312 | 2223.676852 | 2526.538584 |
| TCGA_TCGA-XF-AAME | 1410.155551 | 2192.535051 | 3602.690602 |
| TCGA_TCGA-XF-AAN0 | -546.9028792 | 760.5652941 | 213.662415 |
| TCGA_TCGA-FD-A62P | 9.971391788 | 994.1633457 | 1004.134737 |
| TCGA_TCGA-CF-A47V | -861.6120775 | 271.1473022 | -590.4647753 |
| TCGA_TCGA-XF-A9SI | 551.9773536 | 2619.932294 | 3171.909648 |
| TCGA_TCGA-GU-A42Q | -1531.320081 | -632.8913778 | -2164.211458 |
| TCGA_TCGA-BT-A42F | -305.935914 | 1922.031176 | 1616.095262 |
| TCGA_TCGA-ZF-A9RF | -834.3603923 | 1710.642781 | 876.2823882 |
| TCGA_TCGA-KQ-A41N | -1328.320708 | -867.6298187 | -2195.950526 |
| TCGA_TCGA-GD-A3OQ | -1287.344572 | -231.2710419 | -1518.615614 |
| TCGA_TCGA-BT-A20U | -467.608567 | 46.31178624 | -421.2967808 |
| TCGA_TCGA-XF-A9SW | 1349.791481 | 979.2763618 | 2329.067843 |
| TCGA_TCGA-FD-A3B3 | 556.4855747 | 1800.159153 | 2356.644728 |
| TCGA_TCGA-ZF-A9RC | -113.7837067 | -370.6097388 | -484.3934455 |
| TCGA_TCGA-E7-A6MD | -63.62800688 | 916.9446386 | 853.3166317 |
| TCGA_TCGA-E5-A4TZ | -2084.072426 | -383.9012645 | -2467.973691 |
| TCGA_TCGA-ZF-A9R4 | -1641.338282 | 53.31888324 | -1588.019399 |
| TCGA_TCGA-GV-A3QH | -2296.855533 | -848.3816555 | -3145.237189 |
| TCGA_TCGA-BT-A0YX | -645.1679357 | 806.3310872 | 161.1631515 |
| TCGA_TCGA-FT-A3EE | -1124.224293 | -347.9728571 | -1472.19715 |
| TCGA_TCGA-ZF-A9RL | -2289.767577 | -984.3107166 | -3274.078293 |
| TCGA_TCGA-ZF-A9R9 | 645.396167 | 389.524461 | 1034.920628 |
| TCGA_TCGA-DK-A1A7 | -1121.172123 | -91.31163066 | -1212.483754 |
| TCGA_TCGA-FD-A3SR | 196.9011746 | 815.1852637 | 1012.086438 |
| TCGA_TCGA-GU-AATP | 383.5743423 | 604.3568368 | 987.9311791 |
| TCGA_TCGA-DK-AA6X | -684.1899571 | 707.0492173 | 22.85926014 |
| TCGA_TCGA-XF-A9T3 | 361.608827 | 842.6894856 | 1204.298313 |
| TCGA_TCGA-PQ-A6FN | -626.0834649 | 564.0013567 | -62.08210813 |
| TCGA_TCGA-CF-A1HR | -901.0204294 | 244.2700508 | -656.7503786 |
| TCGA_TCGA-E7-A3X6 | -767.9213887 | 937.0571314 | 169.1357427 |
| TCGA_TCGA-DK-A3WW | -569.5514485 | 1824.402692 | 1254.851244 |
| TCGA_TCGA-XF-AAMY | 36.15708794 | 57.15569473 | 93.31278267 |
| TCGA_TCGA-K4-A3WS | 960.304863 | 1057.502299 | 2017.807162 |
| TCGA_TCGA-GC-A3YS | 332.8230304 | 765.7502265 | 1098.573257 |
| TCGA_TCGA-DK-A1AF | 1063.506864 | 1552.359606 | 2615.86647 |
| TCGA_TCGA-LT-A5Z6 | -1891.354773 | -68.79434378 | -1960.149116 |
| TCGA_TCGA-GC-A4ZW | -1754.231541 | -671.8180275 | -2426.049569 |
| TCGA_TCGA-K4-A4AB | 29.18370014 | 404.1854488 | 433.3691489 |
| TCGA_TCGA-DK-AA74 | 1294.395384 | 2242.364086 | 3536.75947 |
| TCGA_TCGA-G2-A2EO | 340.5482351 | 1247.096804 | 1587.645039 |
| TCGA_TCGA-DK-A1AG | -1788.829672 | 51.47680332 | -1737.352868 |
| TCGA_TCGA-GV-A3JZ | -490.5146305 | -16.83698655 | -507.3516171 |
| TCGA_TCGA-XF-A9SV | 60.66279613 | -201.8821588 | -141.2193627 |
| TCGA_TCGA-UY-A78K | -628.6769256 | 1624.112005 | 995.4350792 |
| TCGA_TCGA-FD-A6TH | -104.4683461 | 9.630056183 | -94.83828993 |
| TCGA_TCGA-DK-AA6W | -1300.390404 | -230.7267299 | -1531.117134 |
| TCGA_TCGA-CF-A3MG | -2204.578084 | -676.0152156 | -2880.5933 |
| TCGA_TCGA-DK-A3X1 | -642.6063091 | -24.26686635 | -666.8731754 |
| TCGA_TCGA-DK-A1A6 | -574.6352398 | 816.7868785 | 242.1516387 |
| TCGA_TCGA-XF-AAMG | -321.1150797 | -163.7453146 | -484.8603942 |
| TCGA_TCGA-E7-A541 | -908.5864829 | 1078.539874 | 169.9533915 |
| TCGA_TCGA-XF-A8HE | -404.1025773 | 1152.544555 | 748.4419778 |
| TCGA_TCGA-DK-AA6Q | -1634.232279 | 906.839612 | -727.3926667 |
| TCGA_TCGA-UY-A78N | -1288.300279 | -1015.503423 | -2303.803701 |
| TCGA_TCGA-DK-AA75 | -1982.264649 | -849.8784002 | -2832.143049 |
| TCGA_TCGA-ZF-AA5P | 616.8161464 | 772.6218448 | 1389.437991 |
| TCGA_TCGA-GC-A3WC | -309.0451523 | 1570.887029 | 1261.841877 |
| TCGA_TCGA-K4-A4AC | -654.1687363 | 818.9380676 | 164.7693313 |
| TCGA_TCGA-FD-A3SL | 987.935381 | 594.5345088 | 1582.46989 |
| TCGA_TCGA-K4-A5RH | 716.4868272 | 2315.685294 | 3032.172122 |
| TCGA_TCGA-FD-A5C1 | 556.4295124 | 1661.492195 | 2217.921708 |
| TCGA_TCGA-XF-AAN7 | -243.7469785 | -97.92094935 | -341.6679278 |
| TCGA_TCGA-2F-A9KO | -257.3561953 | 1305.183875 | 1047.82768 |
| TCGA_TCGA-DK-A3IM | -1859.756519 | -760.3044918 | -2620.061011 |
| TCGA_TCGA-FD-A3SS | -1079.68896 | -544.5040589 | -1624.193018 |
| TCGA_TCGA-UY-A9PE | -618.3020996 | -388.5515663 | -1006.853666 |
| TCGA_TCGA-GV-A40E | -703.9896971 | 264.8458164 | -439.1438807 |
| TCGA_TCGA-BT-A2LD | -713.5399646 | 70.84738527 | -642.6925793 |
| TCGA_TCGA-UY-A9PD | -371.5006873 | -165.0317284 | -536.5324157 |
| TCGA_TCGA-DK-A3IQ | 1616.361772 | 266.9130391 | 1883.274811 |
| TCGA_TCGA-E7-A4XJ | -1412.944524 | -577.2776765 | -1990.2222 |
| TCGA_TCGA-XF-A9T6 | -1314.072565 | 161.9870313 | -1152.085534 |
| TCGA_TCGA-FD-A43Y | -59.74737559 | 674.8524341 | 615.1050585 |
| TCGA_TCGA-S5-AA26 | -1972.561369 | -697.812236 | -2670.373605 |
| TCGA_TCGA-YC-A9TC | -1054.653484 | -307.2715377 | -1361.925022 |
| TCGA_TCGA-GV-A3QG | 1194.53598 | 2521.230373 | 3715.766353 |
| TCGA_TCGA-K4-A6FZ | -1016.024488 | 756.5352481 | -259.4892395 |
| TCGA_TCGA-KQ-A41R | -1367.623937 | -510.9065002 | -1878.530437 |
| TCGA_TCGA-DK-AA6M | -148.2935024 | 1467.816225 | 1319.522722 |
| TCGA_TCGA-ZF-AA52 | 698.5027711 | 906.7470556 | 1605.249827 |
| TCGA_TCGA-K4-A5RJ | 349.7146885 | 2616.319535 | 2966.034224 |
| TCGA_TCGA-HQ-A5ND | -1774.905281 | -281.6338394 | -2056.53912 |
| TCGA_TCGA-BT-A3PK | 169.7183511 | 836.8386836 | 1006.557035 |
| TCGA_TCGA-FD-A3N6 | -1149.757779 | 195.0455101 | -954.7122693 |
| TCGA_TCGA-UY-A78P | 48.55460787 | 1836.970339 | 1885.524947 |
| TCGA_TCGA-BT-A20Q | 253.4298042 | 891.8167354 | 1145.24654 |
| TCGA_TCGA-CU-A0YR | 0.480570921 | 1142.96364 | 1143.444211 |
| TCGA_TCGA-SY-A9G5 | 613.1885674 | 1548.878664 | 2162.067232 |
| TCGA_TCGA-4Z-AA84 | -1111.068067 | -455.1901855 | -1566.258253 |
| TCGA_TCGA-4Z-AA7Q | -615.6740658 | 1652.737936 | 1037.063871 |
| TCGA_TCGA-FD-A5BT | 1359.746205 | 2256.997064 | 3616.743269 |
| TCGA_TCGA-CF-A27C | -2247.719291 | -493.0854522 | -2740.804743 |
| TCGA_TCGA-E7-A6MF | -1896.681861 | -413.5349825 | -2310.216843 |
| TCGA_TCGA-FD-A6TF | 309.3637139 | 285.4784215 | 594.8421354 |
| TCGA_TCGA-XF-AAMR | 414.5509346 | 417.3671951 | 831.9181297 |
| TCGA_TCGA-XF-A8HF | -291.8573827 | 362.3902759 | 70.53289316 |
| TCGA_TCGA-ZF-AA4U | -2367.338302 | -371.086543 | -2738.424845 |
| TCGA_TCGA-E7-A97Q | -263.2527865 | 161.3788214 | -101.8739651 |
| TCGA_TCGA-ZF-A9R0 | -717.6392449 | 258.0224491 | -459.6167959 |
| TCGA_TCGA-FD-A43S | 714.9451205 | 2109.694787 | 2824.639907 |
| TCGA_TCGA-XF-A8HG | -1775.554901 | -595.1317369 | -2370.686638 |
| TCGA_TCGA-K4-A83P | 1004.836299 | 2347.862856 | 3352.699155 |
| TCGA_TCGA-GD-A2C5 | -315.2175432 | -100.2292732 | -415.4468164 |
| TCGA_TCGA-XF-AAMX | -493.5528507 | -378.8197137 | -872.3725644 |
| TCGA_TCGA-DK-A1AE | -1328.695392 | -491.9590539 | -1820.654446 |
| TCGA_TCGA-FJ-A3ZE | -2131.470644 | -1099.737803 | -3231.208447 |
| TCGA_TCGA-DK-A2I1 | 682.3785983 | 1094.20791 | 1776.586509 |
| TCGA_TCGA-DK-A6B0 | -1793.573708 | -320.192388 | -2113.766096 |
| TCGA_TCGA-FD-A5BR | -276.1381628 | 867.8429788 | 591.704816 |
| TCGA_TCGA-GD-A3OP | -114.2544115 | 152.0150276 | 37.76061614 |
| TCGA_TCGA-KQ-A41P | -183.6747651 | 397.5962471 | 213.921482 |
| TCGA_TCGA-UY-A9PH | 110.1271923 | 1869.960693 | 1980.087885 |
| TCGA_TCGA-FD-A5BY | -334.5045614 | 273.7724918 | -60.73206965 |
| TCGA_TCGA-K4-A3WU | 45.39216416 | 1344.687022 | 1390.079187 |
| TCGA_TCGA-FD-A3SJ | -332.1800666 | -134.7937736 | -466.9738402 |
| TCGA_TCGA-E7-A519 | -804.0629194 | 1198.060185 | 393.9972658 |
| TCGA_TCGA-DK-A3IU | 759.8949812 | 2570.848958 | 3330.743939 |
| TCGA_TCGA-XF-A9T0 | -329.2328687 | 495.4747969 | 166.2419282 |
| TCGA_TCGA-YF-AA3M | -1616.422778 | -248.2269446 | -1864.649723 |
| TCGA_TCGA-4Z-AA7Y | -1779.373881 | -584.7436951 | -2364.117576 |
| TCGA_TCGA-KQ-A41Q | -1501.993544 | -525.7265897 | -2027.720134 |
| TCGA_TCGA-FD-A3SP | 1511.182554 | 1727.476952 | 3238.659506 |
| TCGA_TCGA-GC-A6I3 | -315.1157532 | 1500.197676 | 1185.081922 |
| TCGA_TCGA-4Z-AA80 | -1801.525372 | -602.436212 | -2403.961584 |
| TCGA_TCGA-G2-A3IE | -1409.791218 | -236.9270256 | -1646.718244 |
| TCGA_TCGA-CF-A9FF | -710.15766 | 318.2242761 | -391.9333839 |
| TCGA_TCGA-XF-A9ST | -1567.190883 | -1047.789816 | -2614.980699 |
| TCGA_TCGA-DK-A6B5 | -1045.226624 | 636.2189951 | -409.0076288 |
| TCGA_TCGA-GC-A3RC | -534.089085 | 1249.337511 | 715.2484257 |
| TCGA_TCGA-GV-A3QK | -1299.254849 | 759.0625353 | -540.1923134 |
| TCGA_TCGA-DK-AA71 | -1365.005272 | -344.2425555 | -1709.247828 |
| TCGA_TCGA-KQ-A41O | -2700.005765 | -1493.147752 | -4193.153517 |
| TCGA_TCGA-E7-A677 | -1179.30857 | 11.38948639 | -1167.919084 |
| TCGA_TCGA-DK-A6B1 | -1280.352477 | -493.8684728 | -1774.22095 |
| TCGA_TCGA-CF-A5U8 | -1832.733657 | -572.3924222 | -2405.126079 |
| TCGA_TCGA-GU-AATO | -221.8870073 | 1004.101172 | 782.2141651 |
| TCGA_TCGA-CF-A9FH | -1976.18118 | -336.4903199 | -2312.6715 |
| TCGA_TCGA-FD-A6TG | 400.4023482 | 692.5981675 | 1093.000516 |
| TCGA_TCGA-4Z-AA7O | -701.3619457 | 504.2334688 | -197.1284769 |
| TCGA_TCGA-GV-A3QI | -1909.672014 | -1237.854357 | -3147.526371 |
| TCGA_TCGA-DK-A6AV | -1096.025501 | 184.2112001 | -911.8143005 |
| TCGA_TCGA-DK-A1A3 | 107.3135367 | 512.9428991 | 620.2564358 |
| TCGA_TCGA-GD-A6C6 | -1115.177302 | 64.06482474 | -1051.112478 |
| TCGA_TCGA-GC-A3RD | -1263.018481 | -216.9490692 | -1479.96755 |
| TCGA_TCGA-BT-A20R | 696.4147906 | 438.0224962 | 1134.437287 |
| GSE13507_GSM340606 | 809.2438477 | 545.113704 | 1354.357552 |
| GSE13507_GSM340607 | -410.6316003 | 1605.462557 | 1194.830956 |
| GSE13507_GSM340608 | -1840.317591 | -332.7055559 | -2173.023147 |
| GSE13507_GSM340609 | -1674.745563 | -327.9049291 | -2002.650492 |
| GSE13507_GSM340610 | -1268.321529 | 595.3662504 | -672.9552783 |
| GSE13507_GSM340611 | -1439.257266 | -182.1009624 | -1621.358228 |
| GSE13507_GSM340612 | 748.9519085 | 633.7988752 | 1382.750784 |
| GSE13507_GSM340613 | -550.3875992 | 674.9612089 | 124.5736098 |
| GSE13507_GSM340614 | -1159.113133 | -371.8628534 | -1530.975987 |
| GSE13507_GSM340615 | -1076.904858 | 489.4872576 | -587.4176009 |
| GSE13507_GSM340616 | -693.6366615 | 730.7209998 | 37.08433832 |
| GSE13507_GSM340617 | -1362.05824 | 174.746508 | -1187.311732 |
| GSE13507_GSM340618 | -1424.91624 | -302.252634 | -1727.168874 |
| GSE13507_GSM340619 | -1394.871962 | -394.4462332 | -1789.318195 |
| GSE13507_GSM340620 | -534.4094616 | -218.5087142 | -752.9181758 |
| GSE13507_GSM340621 | -1575.123246 | -369.3312051 | -1944.454451 |
| GSE13507_GSM340622 | -1394.385525 | 239.2418951 | -1155.14363 |
| GSE13507_GSM340623 | -995.332034 | 445.7108814 | -549.6211526 |
| GSE13507_GSM340624 | -1274.462033 | -44.19539794 | -1318.657431 |
| GSE13507_GSM340625 | -1784.871299 | -335.185625 | -2120.056924 |
| GSE13507_GSM340626 | -1440.290321 | -85.33005645 | -1525.620377 |
| GSE13507_GSM340627 | -1224.573879 | -200.3306489 | -1424.904528 |
| GSE13507_GSM340628 | -759.3698436 | 529.3448279 | -230.0250157 |
| GSE13507_GSM340629 | -365.8444137 | 1135.906798 | 770.0623841 |
| GSE13507_GSM340630 | -1268.223399 | -410.0288384 | -1678.252237 |
| GSE13507_GSM340631 | -1613.639623 | 19.78351667 | -1593.856106 |
| GSE13507_GSM340632 | -1082.224167 | -157.2990226 | -1239.52319 |
| GSE13507_GSM340633 | -1263.006176 | -61.81146274 | -1324.817639 |
| GSE13507_GSM340634 | -914.3647188 | 130.152303 | -784.2124159 |
| GSE13507_GSM340635 | -561.5534859 | 304.787332 | -256.766154 |
| GSE13507_GSM340636 | -1519.710504 | 936.7804048 | -582.9300987 |
| GSE13507_GSM340637 | -839.2578798 | 716.1326894 | -123.1251904 |
| GSE13507_GSM340638 | -1382.370385 | -12.11735066 | -1394.487736 |
| GSE13507_GSM340639 | -985.2146049 | 93.85918427 | -891.3554207 |
| GSE13507_GSM340640 | -1355.51894 | 334.5025452 | -1021.016395 |
| GSE13507_GSM340641 | -1784.558857 | -659.6967092 | -2444.255566 |
| GSE13507_GSM340642 | -813.6584875 | 31.26287587 | -782.3956116 |
| GSE13507_GSM340643 | -1703.779412 | -19.02055183 | -1722.799963 |
| GSE13507_GSM340644 | -1133.779053 | 253.2161901 | -880.5628624 |
| GSE13507_GSM340645 | -1212.10347 | 479.8883055 | -732.2151645 |
| GSE13507_GSM340646 | -1433.103925 | -190.4566477 | -1623.560573 |
| GSE13507_GSM340647 | -1420.367296 | -118.1735395 | -1538.540835 |
| GSE13507_GSM340648 | -1019.613775 | 92.91229528 | -926.7014792 |
| GSE13507_GSM340649 | -838.5323243 | 563.0015957 | -275.5307286 |
| GSE13507_GSM340650 | -114.8233901 | 1332.838137 | 1218.014746 |
| GSE13507_GSM340651 | -877.3957165 | 100.231334 | -777.1643824 |
| GSE13507_GSM340652 | -1567.685771 | -230.236781 | -1797.922552 |
| GSE13507_GSM340653 | -742.850173 | 66.45395286 | -676.3962201 |
| GSE13507_GSM340654 | -143.7571251 | 1394.903797 | 1251.146672 |
| GSE13507_GSM340655 | -956.8434435 | 231.1391104 | -725.7043331 |
| GSE13507_GSM340656 | -1305.987723 | 225.3723055 | -1080.615418 |
| GSE13507_GSM340657 | -601.7851938 | 1404.614355 | 802.8291608 |
| GSE13507_GSM340658 | -879.0593437 | 101.4956552 | -777.5636886 |
| GSE13507_GSM340659 | -917.0696466 | 763.0265057 | -154.0431408 |
| GSE13507_GSM340660 | 1072.029391 | 929.3953377 | 2001.424729 |
| GSE13507_GSM340661 | -1032.595957 | 137.2984256 | -895.2975312 |
| GSE13507_GSM340662 | -1091.720872 | -204.014087 | -1295.734959 |
| GSE13507_GSM340663 | -975.3402656 | 342.5071901 | -632.8330755 |
| GSE13507_GSM340664 | -1453.575354 | -372.1571284 | -1825.732482 |
| GSE13507_GSM340665 | -92.9360343 | 181.8682188 | 88.9321845 |
| GSE13507_GSM340666 | -1381.082286 | -126.9808488 | -1508.063135 |
| GSE13507_GSM340667 | -1844.913569 | -3.483452983 | -1848.397022 |
| GSE13507_GSM340668 | -1932.683553 | -307.963991 | -2240.647544 |
| GSE13507_GSM340669 | -1081.635259 | -271.4769112 | -1353.11217 |
| GSE13507_GSM340670 | -811.818333 | 533.2247975 | -278.5935355 |
| GSE13507_GSM340671 | -1026.696177 | -72.46171343 | -1099.15789 |
| GSE13507_GSM340672 | -416.4403033 | 531.9462165 | 115.5059132 |
| GSE13507_GSM340673 | -1191.242127 | -260.14327 | -1451.385397 |
| GSE13507_GSM340674 | 1341.555302 | 1273.960602 | 2615.515904 |
| GSE13507_GSM340675 | -1108.445921 | 480.5660915 | -627.8798293 |
| GSE13507_GSM340676 | -811.4289604 | 251.9749302 | -559.4540301 |
| GSE13507_GSM340677 | -628.9044856 | 624.2202956 | -4.684189951 |
| GSE13507_GSM340678 | -106.7906059 | 290.2367779 | 183.446172 |
| GSE13507_GSM340679 | -1090.13591 | 448.1709643 | -641.9649454 |
| GSE13507_GSM340680 | -1474.536162 | -63.0197217 | -1537.555883 |
| GSE13507_GSM340681 | -827.1272331 | 393.9853108 | -433.1419224 |
| GSE13507_GSM340682 | -1320.956612 | -62.53512817 | -1383.49174 |
| GSE13507_GSM340683 | -1092.258818 | -192.4864474 | -1284.745265 |
| GSE13507_GSM340684 | -777.5004217 | 868.8613213 | 91.36089966 |
| GSE13507_GSM340685 | -1428.754144 | 170.6558114 | -1258.098333 |
| GSE13507_GSM340686 | -892.6785684 | 221.2579527 | -671.4206158 |
| GSE13507_GSM340687 | 795.7264797 | 1811.31861 | 2607.04509 |
| GSE13507_GSM340688 | -1498.493382 | -391.0282251 | -1889.521607 |
| GSE13507_GSM340689 | -657.4820139 | 987.8156873 | 330.3336734 |
| GSE13507_GSM340690 | -635.6145493 | 708.2504218 | 72.63587245 |
| GSE13507_GSM340691 | -521.0130218 | 1029.81928 | 508.8062584 |
| GSE13507_GSM340692 | -1234.068209 | -210.1771392 | -1444.245348 |
| GSE13507_GSM340693 | -146.4155221 | 1393.766477 | 1247.350955 |
| GSE13507_GSM340694 | -575.903121 | 1324.468782 | 748.5656606 |
| GSE13507_GSM340695 | -1051.404802 | 296.5455137 | -754.8592883 |
| GSE13507_GSM340696 | 190.6520312 | 1681.589956 | 1872.241987 |
| GSE13507_GSM340697 | 1048.991053 | 1827.420087 | 2876.41114 |
| GSE13507_GSM340698 | -1254.677176 | -137.3341053 | -1392.011281 |
| GSE13507_GSM340699 | 767.0876694 | 1956.725472 | 2723.813141 |
| GSE13507_GSM340700 | -1117.761069 | 209.9107653 | -907.8503033 |
| GSE13507_GSM340701 | -1106.515711 | -839.8401417 | -1946.355853 |
| GSE13507_GSM340702 | -447.4323004 | 881.9983264 | 434.566026 |
| GSE13507_GSM340703 | -943.2533866 | 847.6788791 | -95.57450751 |
| GSE13507_GSM340704 | -1306.05107 | -732.8207834 | -2038.871854 |
| GSE13507_GSM340705 | 1541.983078 | 2810.649857 | 4352.632935 |
| GSE13507_GSM340706 | -701.4656562 | 607.2251249 | -94.24053131 |
| GSE13507_GSM340707 | -1587.724318 | 73.953552 | -1513.770766 |
| GSE13507_GSM340708 | -996.3028021 | 407.8507451 | -588.452057 |
| GSE13507_GSM340709 | -640.1201226 | -231.1581391 | -871.2782617 |
| GSE13507_GSM340710 | -561.2829094 | 122.773535 | -438.5093743 |
| GSE13507_GSM340711 | -809.255358 | 177.4084205 | -631.8469375 |
| GSE13507_GSM340712 | -819.0479777 | -48.66873044 | -867.7167081 |
| GSE13507_GSM340713 | -427.8350056 | -45.89382419 | -473.7288298 |
| GSE13507_GSM340714 | 448.5609151 | 499.9907731 | 948.5516882 |
| GSE13507_GSM340715 | -654.1303335 | 1384.582488 | 730.4521542 |
| GSE13507_GSM340716 | -1143.425081 | 125.9241089 | -1017.500972 |
| GSE13507_GSM340717 | -1442.780079 | -12.66324635 | -1455.443326 |
| GSE13507_GSM340718 | 32.68195119 | 658.6848265 | 691.3667777 |
| GSE13507_GSM340719 | -699.6904119 | 849.5256944 | 149.8352825 |
| GSE13507_GSM340720 | 1607.128752 | 2255.632386 | 3862.761138 |
| GSE13507_GSM340721 | -509.0310714 | -198.7211938 | -707.7522652 |
| GSE13507_GSM340722 | -567.5527793 | 789.6140936 | 222.0613143 |
| GSE13507_GSM340723 | -788.2064251 | 492.9664294 | -295.2399957 |
| GSE13507_GSM340724 | -1066.085843 | -194.1604562 | -1260.246299 |
| GSE13507_GSM340725 | 111.7419306 | 603.1079513 | 714.8498819 |
| GSE13507_GSM340726 | -1083.980651 | 321.7122343 | -762.2684166 |
| GSE13507_GSM340727 | -119.5202791 | 1053.007978 | 933.4876991 |
| GSE13507_GSM340728 | -289.714817 | 1273.664135 | 983.9493184 |
| GSE13507_GSM340729 | -1817.259391 | -642.7149711 | -2459.974362 |
| GSE13507_GSM340730 | -860.6883652 | 339.6103859 | -521.0779793 |
| GSE13507_GSM340731 | -468.8184937 | 640.7657463 | 171.9472526 |
| GSE13507_GSM340732 | 129.1960911 | 1870.774748 | 1999.970839 |
| GSE13507_GSM340733 | 551.0113968 | 2171.901555 | 2722.912952 |
| GSE13507_GSM340734 | -945.9270886 | 226.0554632 | -719.8716254 |
| GSE13507_GSM340735 | -1144.77056 | -81.87837536 | -1226.648935 |
| GSE13507_GSM340736 | -1139.233707 | -99.25478947 | -1238.488496 |
| GSE13507_GSM340737 | -1238.033387 | -227.8967326 | -1465.930119 |
| GSE13507_GSM340738 | -1155.580757 | 239.3673163 | -916.2134408 |
| GSE13507_GSM340739 | -532.1664885 | 1254.212911 | 722.0464223 |
| GSE13507_GSM340740 | -360.8807268 | 641.4138395 | 280.5331127 |
| GSE13507_GSM340741 | -839.7712073 | 965.7214928 | 125.9502855 |
| GSE13507_GSM340742 | 685.1800894 | 1015.175624 | 1700.355714 |
| GSE13507_GSM340743 | -611.7888998 | 706.2589569 | 94.47005712 |
| GSE13507_GSM340744 | -271.9203973 | 1303.261054 | 1031.340657 |
| GSE13507_GSM340745 | -885.8113455 | 1349.180774 | 463.3694288 |
| GSE13507_GSM340746 | 2055.284228 | 1772.365252 | 3827.64948 |
| GSE13507_GSM340747 | -1053.805509 | -190.8857168 | -1244.691226 |
| GSE13507_GSM340748 | -1357.736439 | 86.14402636 | -1271.592412 |
| GSE13507_GSM340749 | 735.859681 | 1437.664958 | 2173.524639 |
| GSE13507_GSM340750 | 111.8948718 | 20.3872601 | 132.2821319 |
| GSE13507_GSM340751 | -671.541118 | 617.6036155 | -53.93750252 |
| GSE13507_GSM340752 | -196.5433427 | 390.992692 | 194.4493494 |
| GSE13507_GSM340753 | -1048.237595 | -76.86046118 | -1125.098056 |
| GSE13507_GSM340754 | -1030.021098 | 37.04429264 | -992.9768056 |
| GSE13507_GSM340755 | -1139.958434 | -11.82033078 | -1151.778765 |
| GSE13507_GSM340756 | -1073.093471 | 168.4421575 | -904.6513132 |
| GSE13507_GSM340757 | -1620.7633 | -353.1916784 | -1973.954978 |
| GSE13507_GSM340758 | 1078.933137 | 1028.484557 | 2107.417694 |
| GSE13507_GSM340759 | 1190.631904 | 1164.03132 | 2354.663224 |
| GSE13507_GSM340760 | -571.6619849 | -170.6063638 | -742.2683487 |
| GSE13507_GSM340761 | 1601.885714 | 1051.690491 | 2653.576206 |
| GSE13507_GSM340762 | -696.0857075 | 545.6162179 | -150.4694896 |
| GSE13507_GSM340763 | -51.8484319 | 1455.612249 | 1403.763817 |
| GSE13507_GSM340764 | -843.1294981 | -187.0158992 | -1030.145397 |
| GSE13507_GSM340765 | -1209.491705 | 21.26450039 | -1188.227205 |
| GSE13507_GSM340766 | 670.6080874 | 2765.318106 | 3435.926193 |
| GSE13507_GSM340767 | -1180.660072 | 390.8279882 | -789.8320837 |
| GSE13507_GSM340768 | -920.0568216 | -127.0036867 | -1047.060508 |
| GSE13507_GSM340769 | 1569.345511 | 2123.644551 | 3692.990062 |
