# Supplementary material for: The Immune Cell Infiltration Patterns and Characterization Score in Bladder Cancer to Identify Prognosis
Source: Front Genet. 2022 Jun 21;13:852708. doi: 10.3389/fgene.2022.852708 (PMC9255635; doi:10.3389/fgene.2022.852708)
Supplement: Supplementary file 7 [file Table5.DOC]

**Supplementary Table 6:** Functions and pathways enrichment analyses of ICI gene signature A and B.

| ICI gene signature A | | | | | |
| --- | --- | --- | --- | --- | --- |
| ONTOLOGY | ID | pvalue | p.adjust | qvalue | Count |
| BP | GO:0070268 | 5.04E-30 | 2.69E-27 | 2.06E-27 | 17 |
| BP | GO:0008544 | 5.00E-26 | 1.33E-23 | 1.02E-23 | 21 |
| BP | GO:0031424 | 1.07E-24 | 1.90E-22 | 1.45E-22 | 17 |
| BP | GO:0030216 | 3.05E-24 | 4.06E-22 | 3.11E-22 | 18 |
| BP | GO:0009913 | 7.56E-23 | 8.06E-21 | 6.18E-21 | 18 |
| BP | GO:0043588 | 1.19E-21 | 1.06E-19 | 8.09E-20 | 18 |
| BP | GO:0018149 | 2.30E-11 | 1.75E-09 | 1.34E-09 | 6 |
| BP | GO:0030574 | 1.31E-06 | 8.74E-05 | 6.70E-05 | 4 |
| BP | GO:0045104 | 1.69E-06 | 9.75E-05 | 7.47E-05 | 4 |
| BP | GO:0045103 | 1.83E-06 | 9.75E-05 | 7.47E-05 | 4 |
| BP | GO:0022617 | 1.23E-05 | 0.000597071 | 0.000457517 | 4 |
| BP | GO:0032963 | 4.37E-05 | 0.00193944 | 0.001486132 | 4 |
| BP | GO:0030198 | 5.56E-05 | 0.002148406 | 0.001646255 | 6 |
| BP | GO:0043062 | 5.64E-05 | 0.002148406 | 0.001646255 | 6 |
| BP | GO:0010951 | 7.98E-05 | 0.002836806 | 0.002173755 | 5 |
| BP | GO:0010466 | 0.000102394 | 0.003410991 | 0.002613735 | 5 |
| BP | GO:1904645 | 0.000126883 | 0.003978155 | 0.003048334 | 3 |
| BP | GO:0043312 | 0.000175669 | 0.005094942 | 0.003904093 | 6 |
| BP | GO:0002283 | 0.000181621 | 0.005094942 | 0.003904093 | 6 |
| BP | GO:0086069 | 0.000266477 | 0.007101601 | 0.005441732 | 2 |
| BP | GO:0007565 | 0.000365143 | 0.009267671 | 0.007101523 | 4 |
| BP | GO:0045861 | 0.000421232 | 0.010205301 | 0.007819999 | 5 |
| BP | GO:0051546 | 0.000498008 | 0.011059938 | 0.008474881 | 2 |
| BP | GO:0098911 | 0.000498008 | 0.011059938 | 0.008474881 | 2 |
| BP | GO:1903035 | 0.000568388 | 0.012118031 | 0.009285664 | 3 |
| BP | GO:0044706 | 0.000624673 | 0.012805798 | 0.009812678 | 4 |
| BP | GO:0045109 | 0.000867983 | 0.015601803 | 0.011955168 | 2 |
| BP | GO:0046697 | 0.000867983 | 0.015601803 | 0.011955168 | 2 |
| BP | GO:0061436 | 0.000867983 | 0.015601803 | 0.011955168 | 2 |
| BP | GO:0052548 | 0.00087815 | 0.015601803 | 0.011955168 | 5 |
| BP | GO:0042730 | 0.000939287 | 0.016149669 | 0.012374981 | 2 |
| BP | GO:0033561 | 0.001090076 | 0.018156576 | 0.013912811 | 2 |
| BP | GO:0052547 | 0.001206383 | 0.019484909 | 0.01493067 | 5 |
| BP | GO:0051346 | 0.001288917 | 0.020205669 | 0.015482965 | 5 |
| BP | GO:0098901 | 0.001607235 | 0.024475896 | 0.018755106 | 2 |
| BP | GO:0001893 | 0.001800977 | 0.026664458 | 0.020432131 | 2 |
| BP | GO:0086005 | 0.002005275 | 0.0288868 | 0.022135042 | 2 |
| BP | GO:0086091 | 0.002111361 | 0.029614614 | 0.022692742 | 2 |
| BP | GO:0086004 | 0.00256174 | 0.035010441 | 0.026827395 | 2 |
| BP | GO:0031214 | 0.002855982 | 0.037127772 | 0.028449838 | 3 |
| BP | GO:0110148 | 0.002855982 | 0.037127772 | 0.028449838 | 3 |
| BP | GO:1903115 | 0.003053351 | 0.038551807 | 0.029541031 | 2 |
| BP | GO:0007156 | 0.003110183 | 0.038551807 | 0.029541031 | 3 |
| BP | GO:0030195 | 0.003448779 | 0.041777256 | 0.03201259 | 2 |
| BP | GO:1900047 | 0.003585632 | 0.042469814 | 0.032543276 | 2 |
| BP | GO:1903034 | 0.003954867 | 0.045824872 | 0.035114151 | 3 |
| BP | GO:0050819 | 0.004158026 | 0.046853176 | 0.035902108 | 2 |
| BP | GO:0086002 | 0.004307328 | 0.046853176 | 0.035902108 | 2 |
| BP | GO:0086065 | 0.004307328 | 0.046853176 | 0.035902108 | 2 |
| BP | GO:0098900 | 0.004459093 | 0.047533934 | 0.036423751 | 2 |
| BP | GO:0007566 | 0.004613314 | 0.048213658 | 0.036944602 | 2 |
| BP | GO:0050678 | 0.004792291 | 0.04885816 | 0.037438463 | 4 |
| BP | GO:0051216 | 0.004858316 | 0.04885816 | 0.037438463 | 3 |
| BP | GO:0034113 | 0.005254584 | 0.051864689 | 0.039742272 | 2 |
| BP | GO:0060135 | 0.005589731 | 0.054169576 | 0.041508434 | 2 |
| BP | GO:0045216 | 0.005800617 | 0.054958104 | 0.042112658 | 3 |
| BP | GO:0050679 | 0.005877321 | 0.054958104 | 0.042112658 | 3 |
| BP | GO:0032102 | 0.006614167 | 0.060781914 | 0.04657526 | 4 |
| BP | GO:0050891 | 0.006837791 | 0.061771912 | 0.047333864 | 2 |
| BP | GO:0086003 | 0.007407912 | 0.065806947 | 0.050425784 | 2 |
| BP | GO:0086001 | 0.007602589 | 0.066429183 | 0.050902583 | 2 |
| BP | GO:0050673 | 0.007736599 | 0.066509793 | 0.050964352 | 4 |
| BP | GO:0061045 | 0.008200422 | 0.069378175 | 0.053162302 | 2 |
| BP | GO:0030104 | 0.008404272 | 0.06999183 | 0.053632527 | 2 |
| BP | GO:0030193 | 0.009029427 | 0.073524725 | 0.056339673 | 2 |
| BP | GO:0055117 | 0.00924232 | 0.073524725 | 0.056339673 | 2 |
| BP | GO:1900046 | 0.00924232 | 0.073524725 | 0.056339673 | 2 |
| BP | GO:0034109 | 0.00989441 | 0.077554717 | 0.059427729 | 2 |
| BP | GO:0050818 | 0.010340234 | 0.079874562 | 0.061205352 | 2 |
| BP | GO:0061448 | 0.010623453 | 0.080890006 | 0.061983455 | 3 |
| BP | GO:0098742 | 0.012341947 | 0.092197699 | 0.070648183 | 3 |
| BP | GO:1901655 | 0.012454473 | 0.092197699 | 0.070648183 | 2 |
| BP | GO:0006942 | 0.012700145 | 0.092728455 | 0.071054884 | 2 |
| BP | GO:0002027 | 0.014218481 | 0.102411495 | 0.078474692 | 2 |
| BP | GO:0002934 | 0.01735885 | 0.118618806 | 0.090893842 | 1 |
| BP | GO:0051541 | 0.01735885 | 0.118618806 | 0.090893842 | 1 |
| BP | GO:0071394 | 0.01735885 | 0.118618806 | 0.090893842 | 1 |
| BP | GO:0090673 | 0.01735885 | 0.118618806 | 0.090893842 | 1 |
| BP | GO:0030282 | 0.017762527 | 0.119840849 | 0.091830255 | 2 |
| BP | GO:0042447 | 0.019078576 | 0.127111012 | 0.097401151 | 1 |
| BP | GO:0070252 | 0.019512065 | 0.128394206 | 0.098384422 | 2 |
| BP | GO:0001867 | 0.020795383 | 0.131951659 | 0.101110386 | 1 |
| BP | GO:0031581 | 0.020795383 | 0.131951659 | 0.101110386 | 1 |
| BP | GO:0051549 | 0.020795383 | 0.131951659 | 0.101110386 | 1 |
| BP | GO:0016264 | 0.022509277 | 0.139505172 | 0.106898403 | 1 |
| BP | GO:0060340 | 0.022509277 | 0.139505172 | 0.106898403 | 1 |
| BP | GO:0001508 | 0.024187401 | 0.143437773 | 0.109911832 | 2 |
| BP | GO:0010838 | 0.024220262 | 0.143437773 | 0.109911832 | 1 |
| BP | GO:0048681 | 0.024220262 | 0.143437773 | 0.109911832 | 1 |
| BP | GO:0051547 | 0.024220262 | 0.143437773 | 0.109911832 | 1 |
| BP | GO:0060048 | 0.024842391 | 0.144740383 | 0.11090998 | 2 |
| BP | GO:0010631 | 0.025525251 | 0.144740383 | 0.11090998 | 3 |
| BP | GO:0051290 | 0.025928342 | 0.144740383 | 0.11090998 | 1 |
| BP | GO:0060339 | 0.025928342 | 0.144740383 | 0.11090998 | 1 |
| BP | GO:0070571 | 0.025928342 | 0.144740383 | 0.11090998 | 1 |
| BP | GO:0090132 | 0.026069562 | 0.144740383 | 0.11090998 | 3 |
| BP | GO:0090130 | 0.027177038 | 0.145828394 | 0.111743689 | 3 |
| BP | GO:0007043 | 0.027191668 | 0.145828394 | 0.111743689 | 2 |
| BP | GO:0061337 | 0.027534401 | 0.145828394 | 0.111743689 | 2 |
| BP | GO:0043508 | 0.027633523 | 0.145828394 | 0.111743689 | 1 |
| BP | GO:0090136 | 0.027633523 | 0.145828394 | 0.111743689 | 1 |
| BP | GO:0061041 | 0.028573134 | 0.148914156 | 0.11410821 | 2 |
| BP | GO:0030048 | 0.029274333 | 0.148914156 | 0.11410821 | 2 |
| BP | GO:0090185 | 0.029335809 | 0.148914156 | 0.11410821 | 1 |
| BP | GO:2001267 | 0.029335809 | 0.148914156 | 0.11410821 | 1 |
| BP | GO:0001890 | 0.03033906 | 0.152553954 | 0.116897273 | 2 |
| BP | GO:0035313 | 0.031035205 | 0.153164486 | 0.117365104 | 1 |
| BP | GO:0043011 | 0.031035205 | 0.153164486 | 0.117365104 | 1 |
| BP | GO:0002076 | 0.032731716 | 0.154389423 | 0.118303735 | 1 |
| BP | GO:0003417 | 0.032731716 | 0.154389423 | 0.118303735 | 1 |
| BP | GO:0045780 | 0.032731716 | 0.154389423 | 0.118303735 | 1 |
| BP | GO:0046852 | 0.032731716 | 0.154389423 | 0.118303735 | 1 |
| BP | GO:2000696 | 0.032731716 | 0.154389423 | 0.118303735 | 1 |
| BP | GO:0007620 | 0.034425347 | 0.159553999 | 0.122261186 | 1 |
| BP | GO:0048670 | 0.034425347 | 0.159553999 | 0.122261186 | 1 |
| BP | GO:0001503 | 0.03477311 | 0.159776447 | 0.122431641 | 3 |
| BP | GO:0030856 | 0.035889454 | 0.163134594 | 0.125004883 | 2 |
| BP | GO:0001502 | 0.036116102 | 0.163134594 | 0.125004883 | 1 |
| BP | GO:0006937 | 0.036657319 | 0.163891272 | 0.125584701 | 2 |
| BP | GO:0007398 | 0.037803985 | 0.163891272 | 0.125584701 | 1 |
| BP | GO:0046058 | 0.037803985 | 0.163891272 | 0.125584701 | 1 |
| BP | GO:0051291 | 0.037803985 | 0.163891272 | 0.125584701 | 1 |
| BP | GO:0010634 | 0.037821063 | 0.163891272 | 0.125584701 | 2 |
| BP | GO:0006941 | 0.038604783 | 0.165938299 | 0.127153274 | 2 |
| BP | GO:1903589 | 0.039489003 | 0.167628981 | 0.12844879 | 1 |
| BP | GO:0034329 | 0.039627114 | 0.167628981 | 0.12844879 | 3 |
| BP | GO:0032331 | 0.041171159 | 0.170110291 | 0.130350139 | 1 |
| BP | GO:0070841 | 0.041171159 | 0.170110291 | 0.130350139 | 1 |
| BP | GO:0098743 | 0.041171159 | 0.170110291 | 0.130350139 | 1 |
| BP | GO:0071377 | 0.044526904 | 0.179743566 | 0.137731813 | 1 |
| BP | GO:0006979 | 0.04529872 | 0.179743566 | 0.137731813 | 3 |
| BP | GO:0003416 | 0.046200504 | 0.179743566 | 0.137731813 | 1 |
| BP | GO:0006516 | 0.046200504 | 0.179743566 | 0.137731813 | 1 |
| BP | GO:0006706 | 0.046200504 | 0.179743566 | 0.137731813 | 1 |
| BP | GO:0034698 | 0.046200504 | 0.179743566 | 0.137731813 | 1 |
| BP | GO:0048668 | 0.046200504 | 0.179743566 | 0.137731813 | 1 |
| BP | GO:1900120 | 0.046200504 | 0.179743566 | 0.137731813 | 1 |
| BP | GO:1901654 | 0.047627468 | 0.182099188 | 0.139536852 | 2 |
| BP | GO:0090200 | 0.04787126 | 0.182099188 | 0.139536852 | 1 |
| BP | GO:0001773 | 0.049539179 | 0.182099188 | 0.139536852 | 1 |
| BP | GO:0001958 | 0.049539179 | 0.182099188 | 0.139536852 | 1 |
| BP | GO:0008209 | 0.049539179 | 0.182099188 | 0.139536852 | 1 |
| BP | GO:0036075 | 0.049539179 | 0.182099188 | 0.139536852 | 1 |
| BP | GO:0048679 | 0.049539179 | 0.182099188 | 0.139536852 | 1 |
| BP | GO:0060351 | 0.049539179 | 0.182099188 | 0.139536852 | 1 |
| CC | GO:0001533 | 1.51E-24 | 6.51E-23 | 3.67E-23 | 12 |
| CC | GO:0030057 | 5.16E-10 | 1.11E-08 | 6.25E-09 | 5 |
| CC | GO:0005882 | 5.89E-08 | 8.45E-07 | 4.76E-07 | 7 |
| CC | GO:0045111 | 1.69E-07 | 1.82E-06 | 1.03E-06 | 7 |
| CC | GO:0045095 | 1.92E-05 | 0.000164969 | 9.29E-05 | 4 |
| CC | GO:0005911 | 0.00015446 | 0.001106965 | 0.00062326 | 6 |
| CC | GO:0101002 | 0.001180939 | 0.006347545 | 0.003573893 | 3 |
| CC | GO:1904813 | 0.001180939 | 0.006347545 | 0.003573893 | 3 |
| CC | GO:0070820 | 0.002625403 | 0.012543592 | 0.007062488 | 3 |
| CC | GO:0014704 | 0.003342369 | 0.014372187 | 0.008092051 | 2 |
| CC | GO:0101003 | 0.004747084 | 0.018556782 | 0.010448127 | 2 |
| CC | GO:0044291 | 0.006911434 | 0.02476597 | 0.01394412 | 2 |
| CC | GO:0005916 | 0.016748328 | 0.051441293 | 0.028963274 | 1 |
| CC | GO:1990124 | 0.016748328 | 0.051441293 | 0.028963274 | 1 |
| CC | GO:0043256 | 0.020065198 | 0.057520235 | 0.032385934 | 1 |
| CC | GO:0097449 | 0.031589017 | 0.074943254 | 0.042195712 | 1 |
| CC | GO:0005912 | 0.031830422 | 0.074943254 | 0.042195712 | 2 |
| CC | GO:0005775 | 0.034329168 | 0.074943254 | 0.042195712 | 2 |
| CC | GO:0062023 | 0.034750525 | 0.074943254 | 0.042195712 | 3 |
| CC | GO:0005922 | 0.034857327 | 0.074943254 | 0.042195712 | 1 |
| MF | GO:0030280 | 7.53E-09 | 3.91E-07 | 1.82E-07 | 4 |
| MF | GO:0004867 | 2.45E-05 | 0.000622667 | 0.000289906 | 4 |
| MF | GO:0004222 | 3.59E-05 | 0.000622667 | 0.000289906 | 4 |
| MF | GO:0086080 | 0.000227016 | 0.001918066 | 0.000893027 | 2 |
| MF | GO:0005518 | 0.000243494 | 0.001918066 | 0.000893027 | 3 |
| MF | GO:0004866 | 0.000276563 | 0.001918066 | 0.000893027 | 4 |
| MF | GO:0008237 | 0.000312692 | 0.001918066 | 0.000893027 | 4 |
| MF | GO:0030414 | 0.000312692 | 0.001918066 | 0.000893027 | 4 |
| MF | GO:0061135 | 0.000331973 | 0.001918066 | 0.000893027 | 4 |
| MF | GO:0061134 | 0.000645445 | 0.003356313 | 0.001562656 | 4 |
| MF | GO:0005200 | 0.000778115 | 0.003678363 | 0.001712598 | 3 |
| MF | GO:0098632 | 0.003560622 | 0.015429361 | 0.007183711 | 2 |
| MF | GO:0004857 | 0.004231002 | 0.016924007 | 0.007879598 | 4 |
| MF | GO:0004869 | 0.004736921 | 0.016969165 | 0.007900624 | 2 |
| MF | GO:0098631 | 0.004894952 | 0.016969165 | 0.007900624 | 2 |
| MF | GO:0004175 | 0.006890155 | 0.022393005 | 0.010425893 | 4 |
| MF | GO:0045295 | 0.020730798 | 0.063411854 | 0.029523738 | 1 |
| MF | GO:0002020 | 0.023707243 | 0.068487592 | 0.031886935 | 2 |
| MF | GO:0019215 | 0.025848084 | 0.070742124 | 0.032936617 | 1 |
| MF | GO:0005521 | 0.027548082 | 0.071625013 | 0.033347678 | 1 |
| MF | GO:0070628 | 0.029245206 | 0.072010107 | 0.033526973 | 1 |
| MF | GO:0016755 | 0.03093946 | 0.072010107 | 0.033526973 | 1 |
| MF | GO:0004252 | 0.034905136 | 0.072010107 | 0.033526973 | 2 |
| MF | GO:0008201 | 0.034905136 | 0.072010107 | 0.033526973 | 2 |
| MF | GO:0005243 | 0.036005053 | 0.072010107 | 0.033526973 | 1 |
| MF | GO:0051428 | 0.036005053 | 0.072010107 | 0.033526973 | 1 |
| MF | GO:0017134 | 0.039367854 | 0.075819571 | 0.03530061 | 1 |
| MF | GO:0008236 | 0.0419443 | 0.077896558 | 0.036267628 | 2 |
| MF | GO:0017171 | 0.04357591 | 0.078136114 | 0.036379162 | 2 |
| ICI gene signature B | | | | | |
| ONTOLOGY | ID | pvalue | p.adjust | qvalue | Count |
| BP | GO:0097028 | 1.59E-05 | 0.004219942 | 0.000787834 | 2 |
| BP | GO:0002573 | 0.000367237 | 0.037394143 | 0.00698123 | 2 |
| BP | GO:0030099 | 0.001538493 | 0.037394143 | 0.00698123 | 2 |
| BP | GO:0045625 | 0.001589404 | 0.037394143 | 0.00698123 | 1 |
| BP | GO:0006979 | 0.001735843 | 0.037394143 | 0.00698123 | 2 |
| BP | GO:0002604 | 0.001748251 | 0.037394143 | 0.00698123 | 1 |
| BP | GO:0097048 | 0.001748251 | 0.037394143 | 0.00698123 | 1 |
| BP | GO:2000668 | 0.001748251 | 0.037394143 | 0.00698123 | 1 |
| BP | GO:0001768 | 0.001907082 | 0.037394143 | 0.00698123 | 1 |
| BP | GO:0002468 | 0.001907082 | 0.037394143 | 0.00698123 | 1 |
| BP | GO:0001767 | 0.002065896 | 0.037394143 | 0.00698123 | 1 |
| BP | GO:0001771 | 0.002224693 | 0.037394143 | 0.00698123 | 1 |
| BP | GO:0043011 | 0.002859713 | 0.037394143 | 0.00698123 | 1 |
| BP | GO:0045063 | 0.003018426 | 0.037394143 | 0.00698123 | 1 |
| BP | GO:0002577 | 0.003177122 | 0.037394143 | 0.00698123 | 1 |
| BP | GO:0002827 | 0.003177122 | 0.037394143 | 0.00698123 | 1 |
| BP | GO:0071731 | 0.003177122 | 0.037394143 | 0.00698123 | 1 |
| BP | GO:0010560 | 0.003494464 | 0.037394143 | 0.00698123 | 1 |
| BP | GO:0045624 | 0.003494464 | 0.037394143 | 0.00698123 | 1 |
| BP | GO:0002407 | 0.003811738 | 0.037394143 | 0.00698123 | 1 |
| BP | GO:0034695 | 0.003811738 | 0.037394143 | 0.00698123 | 1 |
| BP | GO:0090023 | 0.004128945 | 0.037394143 | 0.00698123 | 1 |
| BP | GO:1903020 | 0.004128945 | 0.037394143 | 0.00698123 | 1 |
| BP | GO:0002825 | 0.004287523 | 0.037394143 | 0.00698123 | 1 |
| BP | GO:0001773 | 0.004604629 | 0.037394143 | 0.00698123 | 1 |
| BP | GO:0036336 | 0.004604629 | 0.037394143 | 0.00698123 | 1 |
| BP | GO:0071624 | 0.004604629 | 0.037394143 | 0.00698123 | 1 |
| BP | GO:0043372 | 0.004763157 | 0.037394143 | 0.00698123 | 1 |
| BP | GO:1902624 | 0.004921668 | 0.037394143 | 0.00698123 | 1 |
| BP | GO:0034694 | 0.005080162 | 0.037394143 | 0.00698123 | 1 |
| BP | GO:0043552 | 0.005238639 | 0.037394143 | 0.00698123 | 1 |
| BP | GO:0090022 | 0.005238639 | 0.037394143 | 0.00698123 | 1 |
| BP | GO:0014046 | 0.005555543 | 0.037394143 | 0.00698123 | 1 |
| BP | GO:0014059 | 0.005555543 | 0.037394143 | 0.00698123 | 1 |
| BP | GO:0032735 | 0.005555543 | 0.037394143 | 0.00698123 | 1 |
| BP | GO:0045622 | 0.00571397 | 0.037394143 | 0.00698123 | 1 |
| BP | GO:2000516 | 0.00571397 | 0.037394143 | 0.00698123 | 1 |
| BP | GO:0090218 | 0.005872379 | 0.037394143 | 0.00698123 | 1 |
| BP | GO:0007257 | 0.006347508 | 0.037394143 | 0.00698123 | 1 |
| BP | GO:0008207 | 0.006347508 | 0.037394143 | 0.00698123 | 1 |
| BP | GO:1902622 | 0.006664176 | 0.037394143 | 0.00698123 | 1 |
| BP | GO:0042088 | 0.006980778 | 0.037394143 | 0.00698123 | 1 |
| BP | GO:0010559 | 0.007455553 | 0.037394143 | 0.00698123 | 1 |
| BP | GO:0046638 | 0.007455553 | 0.037394143 | 0.00698123 | 1 |
| BP | GO:0043370 | 0.007613778 | 0.037394143 | 0.00698123 | 1 |
| BP | GO:0015872 | 0.007771986 | 0.037394143 | 0.00698123 | 1 |
| BP | GO:2000107 | 0.007771986 | 0.037394143 | 0.00698123 | 1 |
| BP | GO:0032760 | 0.007930177 | 0.037394143 | 0.00698123 | 1 |
| BP | GO:1903727 | 0.007930177 | 0.037394143 | 0.00698123 | 1 |
| BP | GO:0048260 | 0.008246509 | 0.037394143 | 0.00698123 | 1 |
| BP | GO:0071622 | 0.008246509 | 0.037394143 | 0.00698123 | 1 |
| BP | GO:1903557 | 0.008246509 | 0.037394143 | 0.00698123 | 1 |
| BP | GO:0032731 | 0.00840465 | 0.037394143 | 0.00698123 | 1 |
| BP | GO:1903018 | 0.008562774 | 0.037394143 | 0.00698123 | 1 |
| BP | GO:0032655 | 0.008720881 | 0.037394143 | 0.00698123 | 1 |
| BP | GO:0050433 | 0.008720881 | 0.037394143 | 0.00698123 | 1 |
| BP | GO:0031295 | 0.008878971 | 0.037394143 | 0.00698123 | 1 |
| BP | GO:0032615 | 0.009037045 | 0.037394143 | 0.00698123 | 1 |
| BP | GO:0050432 | 0.009037045 | 0.037394143 | 0.00698123 | 1 |
| BP | GO:0031294 | 0.009195101 | 0.037394143 | 0.00698123 | 1 |
| BP | GO:0043551 | 0.009195101 | 0.037394143 | 0.00698123 | 1 |
| BP | GO:0032732 | 0.009511165 | 0.037394143 | 0.00698123 | 1 |
| BP | GO:0042093 | 0.009511165 | 0.037394143 | 0.00698123 | 1 |
| BP | GO:0002294 | 0.00982716 | 0.037394143 | 0.00698123 | 1 |
| BP | GO:0048488 | 0.00982716 | 0.037394143 | 0.00698123 | 1 |
| BP | GO:0140238 | 0.00982716 | 0.037394143 | 0.00698123 | 1 |
| BP | GO:0002287 | 0.009985133 | 0.037394143 | 0.00698123 | 1 |
| BP | GO:0002293 | 0.009985133 | 0.037394143 | 0.00698123 | 1 |
| BP | GO:0046635 | 0.009985133 | 0.037394143 | 0.00698123 | 1 |
| BP | GO:2000514 | 0.009985133 | 0.037394143 | 0.00698123 | 1 |
| BP | GO:0046637 | 0.010143089 | 0.037394143 | 0.00698123 | 1 |
| BP | GO:0048247 | 0.010301028 | 0.037394143 | 0.00698123 | 1 |
| BP | GO:0098586 | 0.010301028 | 0.037394143 | 0.00698123 | 1 |
| BP | GO:0002548 | 0.010616856 | 0.03751289 | 0.0070034 | 1 |
| BP | GO:0043550 | 0.010616856 | 0.03751289 | 0.0070034 | 1 |
| BP | GO:0002292 | 0.01124831 | 0.03922108 | 0.007322307 | 1 |
| BP | GO:0051937 | 0.011406131 | 0.039254867 | 0.007328615 | 1 |
| BP | GO:0036465 | 0.011721724 | 0.039319707 | 0.00734072 | 1 |
| BP | GO:0043507 | 0.011721724 | 0.039319707 | 0.00734072 | 1 |
| BP | GO:0008344 | 0.011879495 | 0.039350827 | 0.00734653 | 1 |
| BP | GO:0043367 | 0.012352708 | 0.040413179 | 0.007544864 | 1 |
| BP | GO:0009988 | 0.012668099 | 0.040446339 | 0.007551054 | 1 |
| BP | GO:1901224 | 0.012668099 | 0.040446339 | 0.007551054 | 1 |
| BP | GO:0015844 | 0.013298679 | 0.040751834 | 0.007608088 | 1 |
| BP | GO:2000106 | 0.013613869 | 0.040751834 | 0.007608088 | 1 |
| BP | GO:0070098 | 0.013928992 | 0.040751834 | 0.007608088 | 1 |
| BP | GO:0043506 | 0.014086528 | 0.040751834 | 0.007608088 | 1 |
| BP | GO:0070542 | 0.014244047 | 0.040751834 | 0.007608088 | 1 |
| BP | GO:0051952 | 0.01440155 | 0.040751834 | 0.007608088 | 1 |
| BP | GO:0045582 | 0.014559035 | 0.040751834 | 0.007608088 | 1 |
| BP | GO:1903725 | 0.015031392 | 0.040751834 | 0.007608088 | 1 |
| BP | GO:0002690 | 0.015188811 | 0.040751834 | 0.007608088 | 1 |
| BP | GO:0032651 | 0.015346213 | 0.040751834 | 0.007608088 | 1 |
| BP | GO:0035710 | 0.015346213 | 0.040751834 | 0.007608088 | 1 |
| BP | GO:0046634 | 0.015346213 | 0.040751834 | 0.007608088 | 1 |
| BP | GO:0071674 | 0.015346213 | 0.040751834 | 0.007608088 | 1 |
| BP | GO:1990868 | 0.015346213 | 0.040751834 | 0.007608088 | 1 |
| BP | GO:1990869 | 0.015346213 | 0.040751834 | 0.007608088 | 1 |
| BP | GO:0015837 | 0.015660966 | 0.040751834 | 0.007608088 | 1 |
| BP | GO:0045807 | 0.015818318 | 0.040751834 | 0.007608088 | 1 |
| BP | GO:0042102 | 0.015975653 | 0.040751834 | 0.007608088 | 1 |
| BP | GO:0030593 | 0.016290272 | 0.040751834 | 0.007608088 | 1 |
| BP | GO:0120034 | 0.016447556 | 0.040751834 | 0.007608088 | 1 |
| BP | GO:0002824 | 0.016604824 | 0.040751834 | 0.007608088 | 1 |
| BP | GO:0045621 | 0.016604824 | 0.040751834 | 0.007608088 | 1 |
| BP | GO:0048259 | 0.016604824 | 0.040751834 | 0.007608088 | 1 |
| BP | GO:0032611 | 0.016762075 | 0.040751834 | 0.007608088 | 1 |
| BP | GO:0046632 | 0.016762075 | 0.040751834 | 0.007608088 | 1 |
| BP | GO:0046928 | 0.016762075 | 0.040751834 | 0.007608088 | 1 |
| BP | GO:0071887 | 0.017076527 | 0.040783996 | 0.007614093 | 1 |
| BP | GO:0002286 | 0.017233727 | 0.040783996 | 0.007614093 | 1 |
| BP | GO:0002821 | 0.017390911 | 0.040783996 | 0.007614093 | 1 |
| BP | GO:0032680 | 0.017390911 | 0.040783996 | 0.007614093 | 1 |
| BP | GO:0032652 | 0.017548078 | 0.040791586 | 0.00761551 | 1 |
| BP | GO:1903555 | 0.017862363 | 0.040858787 | 0.007628055 | 1 |
| BP | GO:0032640 | 0.018333663 | 0.040858787 | 0.007628055 | 1 |
| BP | GO:0072676 | 0.01849073 | 0.040858787 | 0.007628055 | 1 |
| BP | GO:0051209 | 0.018647779 | 0.040858787 | 0.007628055 | 1 |
| BP | GO:0051283 | 0.018804812 | 0.040858787 | 0.007628055 | 1 |
| BP | GO:0071706 | 0.018804812 | 0.040858787 | 0.007628055 | 1 |
| BP | GO:1901222 | 0.018804812 | 0.040858787 | 0.007628055 | 1 |
| BP | GO:0051588 | 0.018961829 | 0.040858787 | 0.007628055 | 1 |
| BP | GO:0032612 | 0.019118828 | 0.040858787 | 0.007628055 | 1 |
| BP | GO:0051282 | 0.019118828 | 0.040858787 | 0.007628055 | 1 |
| BP | GO:1990266 | 0.019275811 | 0.04086472 | 0.007629163 | 1 |
| BP | GO:0002688 | 0.019589727 | 0.040876201 | 0.007631307 | 1 |
| BP | GO:0051208 | 0.019589727 | 0.040876201 | 0.007631307 | 1 |
| BP | GO:0071621 | 0.020060474 | 0.041531451 | 0.007753637 | 1 |
| BP | GO:0034754 | 0.021158299 | 0.04340672 | 0.008103737 | 1 |
| BP | GO:0050671 | 0.021471813 | 0.04340672 | 0.008103737 | 1 |
| BP | GO:0030534 | 0.021628545 | 0.04340672 | 0.008103737 | 1 |
| BP | GO:0032946 | 0.021628545 | 0.04340672 | 0.008103737 | 1 |
| BP | GO:0046330 | 0.021785259 | 0.04340672 | 0.008103737 | 1 |
| BP | GO:0097553 | 0.022098639 | 0.043670348 | 0.008152955 | 1 |
| BP | GO:0030010 | 0.022255304 | 0.043670348 | 0.008152955 | 1 |
| BP | GO:0002687 | 0.022411952 | 0.043670348 | 0.008152955 | 1 |
| BP | GO:0050921 | 0.022725198 | 0.043957499 | 0.008206564 | 1 |
| BP | GO:0046631 | 0.023038377 | 0.044240361 | 0.008259372 | 1 |
| BP | GO:0045580 | 0.023664534 | 0.044475897 | 0.008303345 | 1 |
| BP | GO:0070665 | 0.023664534 | 0.044475897 | 0.008303345 | 1 |
| BP | GO:0097530 | 0.023664534 | 0.044475897 | 0.008303345 | 1 |
| BP | GO:0002822 | 0.024133976 | 0.044723802 | 0.008349627 | 1 |
| BP | GO:0045834 | 0.024133976 | 0.044723802 | 0.008349627 | 1 |
| BP | GO:0000187 | 0.024603268 | 0.045276847 | 0.008452877 | 1 |
| BP | GO:0060402 | 0.024916045 | 0.045508139 | 0.008496058 | 1 |
| BP | GO:1902107 | 0.025072409 | 0.045508139 | 0.008496058 | 1 |
| BP | GO:0042129 | 0.026322717 | 0.047411597 | 0.00885142 | 1 |
| BP | GO:0002819 | 0.02647893 | 0.047411597 | 0.00885142 | 1 |
| BP | GO:0032874 | 0.026791306 | 0.047648968 | 0.008895736 | 1 |
| BP | GO:0070304 | 0.027103616 | 0.047654197 | 0.008896712 | 1 |
| BP | GO:0007269 | 0.027571955 | 0.047654197 | 0.008896712 | 1 |
| BP | GO:0099643 | 0.027571955 | 0.047654197 | 0.008896712 | 1 |
| BP | GO:0051897 | 0.028040144 | 0.047654197 | 0.008896712 | 1 |
| BP | GO:0060401 | 0.028040144 | 0.047654197 | 0.008896712 | 1 |
| BP | GO:0048469 | 0.028196173 | 0.047654197 | 0.008896712 | 1 |
| BP | GO:0045619 | 0.028508182 | 0.047654197 | 0.008896712 | 1 |
| BP | GO:0071346 | 0.028664162 | 0.047654197 | 0.008896712 | 1 |
| BP | GO:0071347 | 0.028820124 | 0.047654197 | 0.008896712 | 1 |
| BP | GO:0120032 | 0.028820124 | 0.047654197 | 0.008896712 | 1 |
| BP | GO:0038061 | 0.02897607 | 0.047654197 | 0.008896712 | 1 |
| BP | GO:0046328 | 0.02897607 | 0.047654197 | 0.008896712 | 1 |
| BP | GO:0060491 | 0.029132 | 0.047654197 | 0.008896712 | 1 |
| BP | GO:0002285 | 0.029443808 | 0.047828763 | 0.008929302 | 1 |
| BP | GO:0043123 | 0.029599687 | 0.047828763 | 0.008929302 | 1 |
| BP | GO:0042098 | 0.030690375 | 0.049290602 | 0.009202218 | 1 |
| BP | GO:0007626 | 0.031157562 | 0.049638502 | 0.009267168 | 1 |
| BP | GO:0099504 | 0.031313258 | 0.049638502 | 0.009267168 | 1 |
| BP | GO:1901654 | 0.031468937 | 0.049638502 | 0.009267168 | 1 |
| BP | GO:0034341 | 0.031780245 | 0.049832928 | 0.009303466 | 1 |
| BP | GO:1903708 | 0.032091486 | 0.050024964 | 0.009339318 | 1 |
| BP | GO:0030100 | 0.03302481 | 0.050414474 | 0.009412037 | 1 |
| BP | GO:0070555 | 0.033180305 | 0.050414474 | 0.009412037 | 1 |
| BP | GO:0002685 | 0.033335784 | 0.050414474 | 0.009412037 | 1 |
| BP | GO:0007254 | 0.033491247 | 0.050414474 | 0.009412037 | 1 |
| BP | GO:0050870 | 0.033646692 | 0.050414474 | 0.009412037 | 1 |
| BP | GO:0070374 | 0.033802121 | 0.050414474 | 0.009412037 | 1 |
| BP | GO:0051651 | 0.033957533 | 0.050414474 | 0.009412037 | 1 |
| BP | GO:0050670 | 0.03442367 | 0.050414474 | 0.009412037 | 1 |
| BP | GO:0007163 | 0.034579016 | 0.050414474 | 0.009412037 | 1 |
| BP | GO:0099003 | 0.034579016 | 0.050414474 | 0.009412037 | 1 |
| BP | GO:0032944 | 0.034734344 | 0.050414474 | 0.009412037 | 1 |
| BP | GO:0042445 | 0.034734344 | 0.050414474 | 0.009412037 | 1 |
| BP | GO:0097529 | 0.034889657 | 0.050414474 | 0.009412037 | 1 |
| BP | GO:0002699 | 0.035044952 | 0.050414474 | 0.009412037 | 1 |
| BP | GO:0008037 | 0.035510739 | 0.050414474 | 0.009412037 | 1 |
| BP | GO:0006836 | 0.035976376 | 0.050414474 | 0.009412037 | 1 |
| BP | GO:0050920 | 0.035976376 | 0.050414474 | 0.009412037 | 1 |
| BP | GO:0019882 | 0.036441863 | 0.050414474 | 0.009412037 | 1 |
| BP | GO:0030595 | 0.036441863 | 0.050414474 | 0.009412037 | 1 |
| BP | GO:0097305 | 0.036752104 | 0.050414474 | 0.009412037 | 1 |
| BP | GO:0000302 | 0.0369072 | 0.050414474 | 0.009412037 | 1 |
| BP | GO:0001505 | 0.0369072 | 0.050414474 | 0.009412037 | 1 |
| BP | GO:0071695 | 0.0369072 | 0.050414474 | 0.009412037 | 1 |
| BP | GO:1903039 | 0.0369072 | 0.050414474 | 0.009412037 | 1 |
| BP | GO:0032872 | 0.037217342 | 0.050577413 | 0.009442456 | 1 |
| BP | GO:0070302 | 0.037682429 | 0.050689562 | 0.009463394 | 1 |
| BP | GO:0070663 | 0.037682429 | 0.050689562 | 0.009463394 | 1 |
| BP | GO:0009636 | 0.039231639 | 0.052335624 | 0.009770702 | 1 |
| BP | GO:0043122 | 0.039541281 | 0.052335624 | 0.009770702 | 1 |
| BP | GO:0030217 | 0.039696077 | 0.052335624 | 0.009770702 | 1 |
| BP | GO:0051896 | 0.039696077 | 0.052335624 | 0.009770702 | 1 |
| BP | GO:0043406 | 0.041397738 | 0.054308913 | 0.010139102 | 1 |
| BP | GO:0015850 | 0.042016024 | 0.054848505 | 0.01023984 | 1 |
| BP | GO:0043491 | 0.043560578 | 0.05650956 | 0.010549948 | 1 |
| BP | GO:0022409 | 0.043714942 | 0.05650956 | 0.010549948 | 1 |
| BP | GO:0046651 | 0.044332232 | 0.056721681 | 0.010589549 | 1 |
| BP | GO:0007249 | 0.044795025 | 0.056721681 | 0.010589549 | 1 |
| BP | GO:0032943 | 0.044795025 | 0.056721681 | 0.010589549 | 1 |
| BP | GO:0051403 | 0.044795025 | 0.056721681 | 0.010589549 | 1 |
| BP | GO:0021700 | 0.044949257 | 0.056721681 | 0.010589549 | 1 |
| BP | GO:1902105 | 0.04541185 | 0.057033841 | 0.010647827 | 1 |
| BP | GO:0071356 | 0.046490655 | 0.058113319 | 0.010849358 | 1 |
| BP | GO:0031098 | 0.046952751 | 0.058415395 | 0.010905754 | 1 |
| BP | GO:0070372 | 0.047876495 | 0.059286314 | 0.011068349 | 1 |
| BP | GO:0034599 | 0.048491993 | 0.059507624 | 0.011109666 | 1 |
| BP | GO:0060326 | 0.048645826 | 0.059507624 | 0.011109666 | 1 |
| BP | GO:0070588 | 0.048799642 | 0.059507624 | 0.011109666 | 1 |
| BP | GO:0070661 | 0.048953442 | 0.059507624 | 0.011109666 | 1 |
| CC | GO:0043679 | 0.019808439 | 0.068044264 | 0.035812771 | 1 |
| CC | GO:0044306 | 0.022681421 | 0.068044264 | 0.035812771 | 1 |
| CC | GO:0150034 | 0.046652588 | 0.070812909 | 0.037269952 | 1 |
| MF | GO:0048020 | 0.007663843 | 0.031349786 | 0.005999959 | 1 |
| MF | GO:0008009 | 0.007989093 | 0.031349786 | 0.005999959 | 1 |
| MF | GO:0005507 | 0.009776694 | 0.031349786 | 0.005999959 | 1 |
| MF | GO:0042379 | 0.011399922 | 0.031349786 | 0.005999959 | 1 |
| MF | GO:0016616 | 0.019489458 | 0.038687093 | 0.007404228 | 1 |
| MF | GO:0016614 | 0.021102051 | 0.038687093 | 0.007404228 | 1 |
| MF | GO:0005125 | 0.037927656 | 0.057613828 | 0.01102657 | 1 |
| MF | GO:0005126 | 0.043651738 | 0.057613828 | 0.01102657 | 1 |
| MF | GO:0001664 | 0.047138587 | 0.057613828 | 0.01102657 | 1 |
