# Supplementary material for: The Immune Cell Infiltration Patterns and Characterization Score in Bladder Cancer to Identify Prognosis
Source: Front Genet. 2022 Jun 21;13:852708. doi: 10.3389/fgene.2022.852708 (PMC9255635; doi:10.3389/fgene.2022.852708)
Supplement: Supplementary file 8 [file Table2.DOC]

**Supplementary Table 3: Results of ICI clusters to classify BLCA patients.**

| **ID** | **ICI clusters** | **ID** | **ICI clusters** |
| --- | --- | --- | --- |
| TCGA_TCGA-ZF-A9R7 | A | TCGA_TCGA-BT-A42F | B |
| TCGA_TCGA-E7-A97P | A | TCGA_TCGA-ZF-A9RF | A |
| TCGA_TCGA-4Z-AA81 | A | TCGA_TCGA-BT-A20U | B |
| TCGA_TCGA-XF-A9SM | A | TCGA_TCGA-XF-A9SW | A |
| TCGA_TCGA-DK-A3IN | A | TCGA_TCGA-FD-A3B3 | A |
| TCGA_TCGA-DK-A2I4 | A | TCGA_TCGA-E7-A6MD | A |
| TCGA_TCGA-XF-AAMT | B | TCGA_TCGA-BT-A0YX | A |
| TCGA_TCGA-XF-A9SU | A | TCGA_TCGA-XF-A9T3 | B |
| TCGA_TCGA-FD-A6TK | A | TCGA_TCGA-PQ-A6FN | B |
| TCGA_TCGA-XF-A9T5 | B | TCGA_TCGA-E7-A3X6 | A |
| TCGA_TCGA-XF-A9SX | A | TCGA_TCGA-DK-A3WW | A |
| TCGA_TCGA-E7-A7DV | A | TCGA_TCGA-K4-A3WS | A |
| TCGA_TCGA-DK-AA6L | B | TCGA_TCGA-GC-A3YS | A |
| TCGA_TCGA-XF-A9T4 | B | TCGA_TCGA-DK-A1AF | B |
| TCGA_TCGA-UY-A8OB | A | TCGA_TCGA-DK-AA74 | A |
| TCGA_TCGA-DK-A1A5 | B | TCGA_TCGA-G2-A2EO | A |
| TCGA_TCGA-SY-A9G0 | A | TCGA_TCGA-UY-A78K | A |
| TCGA_TCGA-FD-A5BX | B | TCGA_TCGA-FD-A6TH | B |
| TCGA_TCGA-ZF-AA54 | B | TCGA_TCGA-DK-AA6W | B |
| TCGA_TCGA-GU-A766 | A | TCGA_TCGA-DK-A1A6 | A |
| TCGA_TCGA-C4-A0F1 | B | TCGA_TCGA-E7-A541 | A |
| TCGA_TCGA-G2-A2ES | B | TCGA_TCGA-XF-A8HE | A |
| TCGA_TCGA-XF-AAN4 | A | TCGA_TCGA-DK-AA6Q | A |
| TCGA_TCGA-4Z-AA7N | A | TCGA_TCGA-GC-A3WC | A |
| TCGA_TCGA-UY-A8OC | B | TCGA_TCGA-K4-A4AC | A |
| TCGA_TCGA-GV-A3JV | A | TCGA_TCGA-FD-A3SL | B |
| TCGA_TCGA-XF-A9T8 | A | TCGA_TCGA-K4-A5RH | A |
| TCGA_TCGA-E7-A7XN | A | TCGA_TCGA-FD-A5C1 | A |
| TCGA_TCGA-4Z-AA7W | A | TCGA_TCGA-2F-A9KO | A |
| TCGA_TCGA-FD-A43U | B | TCGA_TCGA-DK-A3IM | B |
| TCGA_TCGA-ZF-AA4V | A | TCGA_TCGA-XF-A9T6 | A |
| TCGA_TCGA-ZF-AA53 | A | TCGA_TCGA-FD-A43Y | B |
| TCGA_TCGA-BL-A13I | A | TCGA_TCGA-GV-A3QG | A |
| TCGA_TCGA-CU-A72E | B | TCGA_TCGA-DK-AA6M | A |
| TCGA_TCGA-GC-A3I6 | A | TCGA_TCGA-ZF-AA52 | B |
| TCGA_TCGA-FD-A6TA | A | TCGA_TCGA-K4-A5RJ | A |
| TCGA_TCGA-BL-A3JM | B | TCGA_TCGA-BT-A3PK | B |
| TCGA_TCGA-BL-A5ZZ | B | TCGA_TCGA-FD-A3N6 | B |
| TCGA_TCGA-FD-A62N | A | TCGA_TCGA-UY-A78P | B |
| TCGA_TCGA-BT-A20J | A | TCGA_TCGA-BT-A20Q | A |
| TCGA_TCGA-FT-A61P | B | TCGA_TCGA-CU-A0YR | B |
| TCGA_TCGA-BT-A20O | A | TCGA_TCGA-SY-A9G5 | A |
| TCGA_TCGA-4Z-AA82 | A | TCGA_TCGA-4Z-AA7Q | A |
| TCGA_TCGA-FD-A5BU | A | TCGA_TCGA-FD-A5BT | A |
| TCGA_TCGA-C4-A0F0 | A | TCGA_TCGA-FD-A6TF | A |
| TCGA_TCGA-FD-A3N5 | B | TCGA_TCGA-XF-A8HF | A |
| TCGA_TCGA-DK-AA6S | A | TCGA_TCGA-FD-A43S | A |
| TCGA_TCGA-BT-A42E | A | TCGA_TCGA-K4-A83P | A |
| TCGA_TCGA-ZF-AA4R | B | TCGA_TCGA-DK-A2I1 | A |
| TCGA_TCGA-DK-A3WY | A | TCGA_TCGA-KQ-A41P | B |
| TCGA_TCGA-G2-A2EF | A | TCGA_TCGA-UY-A9PH | A |
| TCGA_TCGA-GU-AATQ | B | TCGA_TCGA-FD-A5BY | B |
| TCGA_TCGA-YC-A8S6 | A | TCGA_TCGA-E7-A519 | A |
| TCGA_TCGA-XF-A9SJ | A | TCGA_TCGA-DK-A3IU | A |
| TCGA_TCGA-DK-AA6T | A | TCGA_TCGA-FD-A3SP | B |
| TCGA_TCGA-XF-AAMW | A | TCGA_TCGA-GC-A6I3 | A |
| TCGA_TCGA-BT-A3PJ | A | TCGA_TCGA-GC-A3RC | B |
| TCGA_TCGA-GC-A3OO | B | TCGA_TCGA-GU-AATO | A |
| TCGA_TCGA-XF-AAN5 | A | TCGA_TCGA-DK-A6AV | A |
| TCGA_TCGA-FD-A6TB | A | TCGA_TCGA-BT-A20R | B |
| TCGA_TCGA-XF-AAN3 | A | GSE13507_GSM340606 | A |
| TCGA_TCGA-FD-A3B8 | A | GSE13507_GSM340607 | A |
| TCGA_TCGA-XF-A9SY | B | GSE13507_GSM340610 | A |
| TCGA_TCGA-CU-A0YN | B | GSE13507_GSM340623 | B |
| TCGA_TCGA-FD-A5BZ | B | GSE13507_GSM340636 | A |
| TCGA_TCGA-XF-A8HD | A | GSE13507_GSM340637 | A |
| TCGA_TCGA-GV-A3JX | A | GSE13507_GSM340645 | B |
| TCGA_TCGA-FD-A3B6 | A | GSE13507_GSM340647 | A |
| TCGA_TCGA-GU-A764 | A | GSE13507_GSM340650 | B |
| TCGA_TCGA-5N-A9KM | A | GSE13507_GSM340654 | B |
| TCGA_TCGA-FD-A6TD | A | GSE13507_GSM340655 | B |
| TCGA_TCGA-ZF-AA56 | A | GSE13507_GSM340660 | A |
| TCGA_TCGA-ZF-AA58 | B | GSE13507_GSM340673 | B |
| TCGA_TCGA-FJ-A871 | A | GSE13507_GSM340674 | A |
| TCGA_TCGA-ZF-A9RN | A | GSE13507_GSM340675 | A |
| TCGA_TCGA-YC-A89H | B | GSE13507_GSM340676 | A |
| TCGA_TCGA-XF-AAMQ | A | GSE13507_GSM340677 | A |
| TCGA_TCGA-FD-A3B4 | B | GSE13507_GSM340679 | A |
| TCGA_TCGA-DK-A1AB | B | GSE13507_GSM340681 | A |
| TCGA_TCGA-BT-A20X | B | GSE13507_GSM340686 | A |
| TCGA_TCGA-ZF-AA5H | B | GSE13507_GSM340687 | B |
| TCGA_TCGA-XF-A9T2 | B | GSE13507_GSM340693 | B |
| TCGA_TCGA-DK-A3WX | B | GSE13507_GSM340694 | A |
| TCGA_TCGA-DK-A6B2 | A | GSE13507_GSM340696 | B |
| TCGA_TCGA-4Z-AA86 | B | GSE13507_GSM340697 | A |
| TCGA_TCGA-XF-A9SL | A | GSE13507_GSM340700 | A |
| TCGA_TCGA-XF-AAN2 | A | GSE13507_GSM340702 | A |
| TCGA_TCGA-ZF-A9RD | A | GSE13507_GSM340705 | B |
| TCGA_TCGA-DK-A2I2 | B | GSE13507_GSM340708 | A |
| TCGA_TCGA-FD-A62S | B | GSE13507_GSM340722 | B |
| TCGA_TCGA-GD-A76B | A | GSE13507_GSM340723 | A |
| TCGA_TCGA-GC-A6I1 | B | GSE13507_GSM340725 | B |
| TCGA_TCGA-XF-A9SK | A | GSE13507_GSM340727 | A |
| TCGA_TCGA-DK-A1AD | A | GSE13507_GSM340728 | B |
| TCGA_TCGA-K4-A5RI | A | GSE13507_GSM340730 | A |
| TCGA_TCGA-XF-AAN8 | A | GSE13507_GSM340732 | A |
| TCGA_TCGA-G2-A2EJ | B | GSE13507_GSM340741 | A |
| TCGA_TCGA-FD-A3B7 | A | GSE13507_GSM340742 | A |
| TCGA_TCGA-FD-A5BS | A | GSE13507_GSM340744 | A |
| TCGA_TCGA-LC-A66R | A | GSE13507_GSM340745 | A |
| TCGA_TCGA-UY-A9PB | A | GSE13507_GSM340746 | A |
| TCGA_TCGA-K4-A54R | A | GSE13507_GSM340751 | B |
| TCGA_TCGA-FD-A43P | A | GSE13507_GSM340752 | A |
| TCGA_TCGA-DK-A3IV | A | GSE13507_GSM340758 | A |
| TCGA_TCGA-GU-A762 | A | GSE13507_GSM340763 | A |
| TCGA_TCGA-XF-AAME | A | GSE13507_GSM340769 | A |
| TCGA_TCGA-FD-A62P | B |  |  |
